# Unraveling the structural landscape of intra-chain domain interfaces: Implication in the evolution of domain-domain interactions

**DOI:** 10.1371/journal.pone.0220336

**Published:** 2019-08-02

**Authors:** Rivi Verma, Shashi Bhushan Pandit

**Affiliations:** Department of Biological Sciences, Indian Institute of Science Education and Research, Mohali, India; University of Michigan, UNITED STATES

## Abstract

Intra-chain domain interactions are known to play a significant role in the function and stability of multidomain proteins. These interactions are mediated through a physical interaction at domain-domain interfaces (DDIs). With a motivation to understand evolution of interfaces, we have investigated similarities among DDIs. Even though interfaces of protein-protein interactions (PPIs) have been previously studied by structurally aligning interfaces, similar analyses have not yet been performed on DDIs of either multidomain proteins or PPIs. For studying the structural landscape of DDIs, we have used iAlign to structurally align intra-chain domain interfaces of domains. The interface alignment of spatially constrained domains (due to inter-domain linkers) showed that ~88% of these could identify a structural matching interface having similar C-alpha geometry and contact pattern despite that aligned domain pairs are not structurally related. Moreover, the mean interface similarity score (IS-score) is 0.307, which is higher compared to the average random IS-score (0.207) suggesting domain interfaces are not random. The structural space of DDIs is highly connected as ~84% of all possible directed edges among interfaces are found to have at most path length of 8 when 0.26 is IS-score threshold. At this threshold, ~83% of interfaces form the largest strongly connected component. Thus, suggesting that structural space of intra-chain domain interfaces is degenerate and highly connected, as has been found in PPI interfaces. Interestingly, searching for structural neighbors of inter-chain interfaces among intra-chain interfaces showed that ~86% could find a statistically significant match to intra-chain interface with a mean IS-score of 0.311. This implies that domain interfaces are degenerate whether formed within a protein or between proteins. The interface degeneracy is most likely due to limited possible ways of packing secondary structures. In principle, interface similarities can be exploited to accurately model domain interfaces in structure prediction of multidomain proteins.

## Introduction

Proteins domains can be defined as an evolutionarily conserved region of sequence or a compact region of protein structure that are usually considered as evolutionary and/or functional units of proteins. Traditionally, sequence domains are classified based on sequence conservation that are documented in databases such as Pfam [1] and SMART [2]. On the contrary, structural domains are identified in experimentally determined tertiary structures and are classified hierarchically in databases such as SCOP [3] and CATH [4] using manual and semi-automated methods respectively. The analyses of completely sequenced genomes have shown that many genes encoded in genomes consist of more than one domain [5,6]. In most genomes, nearly half of their proteome consists of multidomain proteins, and these are relatively abundant in eukaryotes than prokaryotes [5–7]. The multidomain proteins can facilitate complex biological functions such as acting as a scaffold in cellular signaling, assembly of protein complexes and enzymatic catalysis. Taking domains as an evolutionary unit of proteins, it has been suggested that multidomain proteins evolve through the process of domain duplication, fusion and shuffling followed by adaptive changes and/or function divergence [8], which facilitates generation of novel, complex and disparate functions from a limited set of domains [8–10]. Apart from providing functional divergence, multiple domains in a protein can be advantageous for their folding, stability, and cooperative complex functions [6,11].

Previous analyses on multidomain architectures have shown that only a limited repertoire of domain combinations are observed among all possible combinations of domains and some domains are known to associate with specific domains whereas others are versatile and combine with diverse domains [8,12–15]. Furthermore, the order of domains from N- to C–terminal tends to be conserved suggesting a strong evolutionary selection pressure in domain combination [8,9]. As the number of domain combinations is limited, previous studies have focused on tertiary structures analyses to understand the extent of conservation of inter-domain geometry and interacting domain-domain interfaces (DDIs) among homologous proteins [16–18]. The study on the relationship between the conservation of sequence and inter-domain geometry found that similar geometry is observed for conserved domains [16]. The analyses on domain orientation of eight catalytic superfamilies in combination with classical Rossmann superfamily showed that within a given superfamily-superfamily pair, the relative orientation of domains and domain interfaces are conserved. However, these are not conserved when the same superfamily combines with two different superfamilies [19]. Later on, studies using pairs of homologous two-domain proteins from 128 multidomain families, found that ~60% of pairs conserve their interface and geometry as assessed by degree of translation and rotation of second domain when homologous domains are superposed [20]. The remaining domain pairs showed variable inter-domain geometries and interfaces. Importantly, this study found that variable geometry and interface was observed even among homologous structures [20]. In a separate study on two continuous domain proteins have shown that relative orientation of domains is conserved in homologous multidomain proteins as evaluated using the difference in pseudo-torsion angles calculated from the center of masses of domains and Cα residues at domain boundaries [21]. Moreover, the conservation of inter-domain geometry suggested that it is probably constrained by domain interfaces wherein inter-domain linkers modulate DDI by varying their lengths, conformations and local structures [21].

The domain-domain interaction interfaces can play an important role in allosteric regulation, substrate recognition of enzymes [22–27], and can affect folding and/or stability of individual domains in multidomain proteins [6,11,24,28,29]. There have been limited analyses on the extent of structural conservation of domain-domain interfaces among non-homologous domains. Previously, similarities among domain-domain interfaces were assessed using Root Mean Square Deviation (RMSD) of topologically equivalent domain interface residues obtained by aligning individual domains [16,18]. Such a method of interface comparison assumes interaction sites on domains to be similar between two proteins as well as rely on reliable alignments of an individual domain. These could potentially limit the ability to detect similarities among DDIs. Hence, to explore structural relatedness of DDIs, it is more appropriate to structurally align only interfacial residues rather than aligning domains. Recently, a structural alignment method iAlign [30] has been developed that aligns only interface residues of protein-protein interaction (PPIs) interfaces. Moreover, iAlign computes a length independent Interface Similarity score (IS-score) subsequent to the optimal structural alignment. With the availability of appropriate interface alignment method and scoring method, it is possible to investigate structural relatedness of DDIs as well as to understand the evolutionary or functional constraints in the evolution of domain-domain interaction interfaces.

In past years, many analyses have focused on understanding the nature of protein structural space, which can be broadly viewed as a collection of known tertiary structures, mostly from the perspective of evolution as well as prediction and design of protein structures [31–33]. These studies have found that there are usually finite ways of arranging a set of secondary structures with similar topological connections (Fold). Thus, suggesting a limited number of folds would be sufficient to cover complete structural space [32–35]. Later studies on the library of single domain protein structures and their comparison to artificial poly-alanine predicted tertiary structure, have shown that structural space of single domain is likely complete due to the packing of compact, hydrogen-bonded secondary structural elements [36]. The completeness argument of structural space was subsequently extended to the structural space of protein-protein interaction interfaces [37–39]. Interestingly, PPI interfaces are found to be highly degenerate and close to complete, which are mainly due to functional requirements, relatively flat interacting surfaces, and limited ways of packing hydrogen-bonded secondary structural elements [39]. The similarity of PPI interfaces has been utilized to predict PPIs and their interaction surfaces [40].

As protein-protein interaction interfaces has been shown to be structurally degenerate [39], we asked whether the same properties are observed when proteins are discretized as domains, especially among intra-chain domain interaction interfaces in the multidomain proteins. In the present work, we have addressed whether a) domain-domain interfaces in multidomain protein are structurally degenerate, and b) this degeneracy can be extended between intra- and inter-chain (from PPIs) domain-domain interfaces. Through this analysis, we investigated the nature of the structural space of domain interaction interfaces in terms of their structural degeneracy and connectedness. This study provides insight into evolution of interface when proteins are discretized as domains. Eventually, the study could facilitate accurate modeling of domain-domain interaction interfaces by identifying native like domain interfaces before assembling the domains of multidomain protein in the process of their structure prediction.

Since interacting domains could possibly be spatially constrained depending on whether inter-domain linker region/s and domain/s linearly separate them, we evaluated structural degeneracy of DDIs on four non-redundant datasets, which represent a varying degree of constraints on DDIs. The linked domain pairs are: a) fully constrained system is when domains are separated by only one inter-domain linker as in consecutive continuous domains (single sequence segment forming a domain); b) consecutive and discontinuous domains (constituting of one or more sequence segments) pairs are taken as less constrained because at least two inter-domain linkers separate domains, and c) least constrained system when non-consecutive continuous domains, which are separated by more than one domain. Finally, inter-chain domain interfaces are taken as having no constraints for domain interactions. The comparison of intra-chain with inter-chain domain interfaces could also potentially give a basis to construct a combined interface template library to improve structure prediction of interfaces. As has been mentioned before, the similarity of interfaces is evaluated using IS-score after non-sequential structural alignment of the interfaces using iAlign [30]. Hence, this study provides geometrical similarity of interfaces across the structural space of domain-domain within a multidomain protein as well as their similarity with inter-chain domain interfaces.

## Materials and methods

### Intra-chain domain-domain interaction interface dataset

**Fig 1:**
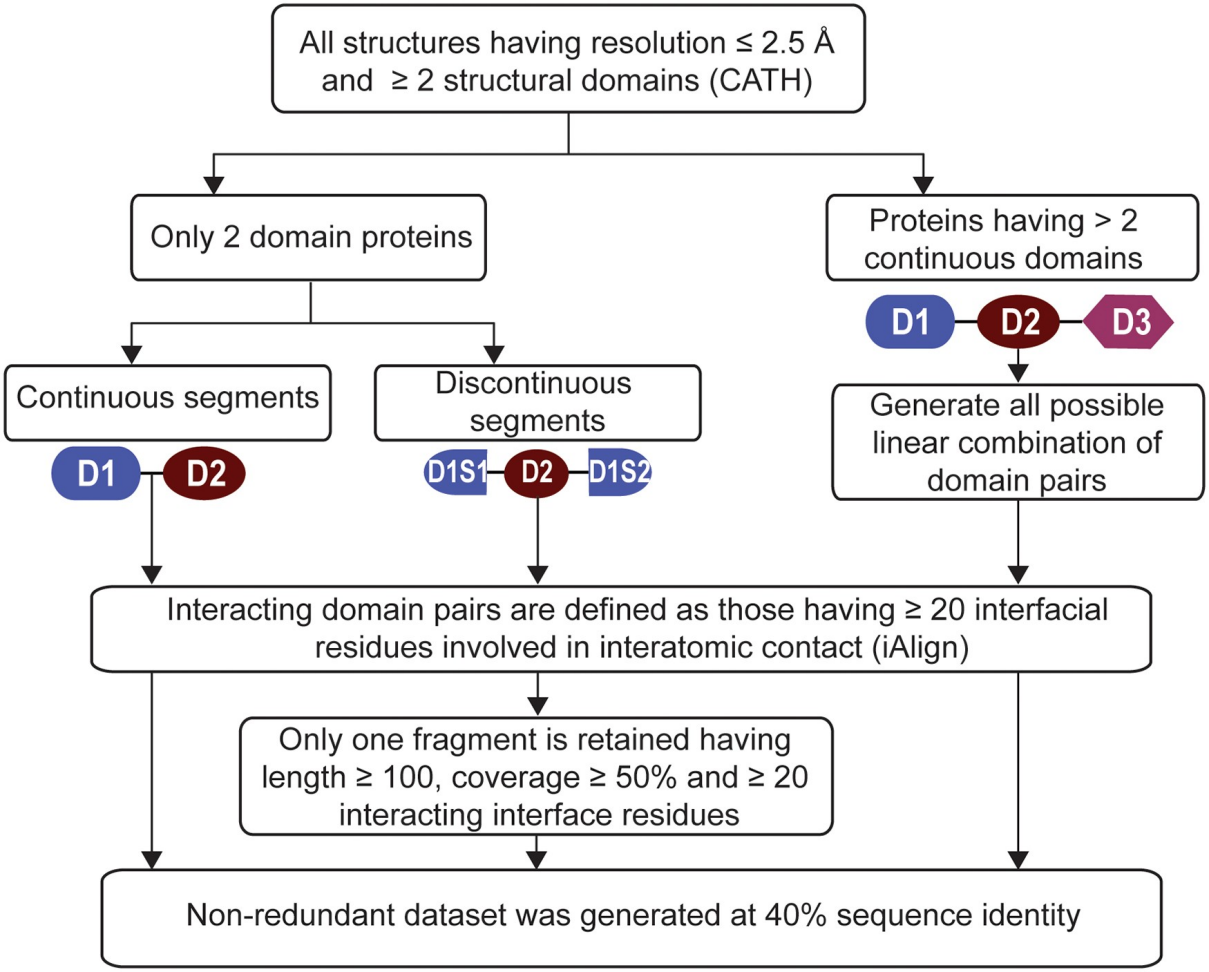
Overview of dataset construction. The flowchart showing steps in the construction of various datasets.

The structural domains and their classification were obtained from CATH (v 4.1.0) database [4]. Using all structural domains delineated and classified in CATH database, we constructed following four datasets: domain-CC-2 (consecutive continuous domains from structures having only two classified domain), domain-CC-M (consecutive continuous domains of structures having 3 or more classified domains), domain-CU-M (continuous and non-consecutive domains of structures consisting of 3 or more classified domains) and domain-CD-2 (consecutive and discontinuous domains of structures having only two classified domains). Fig 1 illustrates the procedure used for the construction of the dataset (Fig 1). Briefly, we took all x-ray crystal structures (PDBID with chain identifier) with resolution ≤ 2.5Å and having at least two classified structural domains from CATH database. We took only classified domains from CATH, because this also consists of domain delineated regions in protein yet unclassified in CATH. Thus, obtained list of structures were mapped to UniProt identifiers [41] from EBI-SIFTS database [42], which documents mapping between PDBIDs with chain identifier to UniProt protein sequence identifiers. In order to consider representative PDBID for each UniProt entry, first, we took structures defined to be interacting by using interaction definition from iAlign (v1.0b7), which considers two proteins (domains) as interacting if at least 20 residues are involved in interatomic contacts in interfacial region. Subsequently, a representative PDBID having the longest length and the best resolution was selected for each UniProt entry. This set of proteins was further divided into 2 datasets a) Only 2 domain structures (set A) and b) rest all structures (set B).

From set A (consists of 2 classified domain proteins), we selected PDB entries having only continuous domains *i*.*e*. domain consisting of only one single segment. The continuous domains separated only by inter-domain linker (not any structural domain) are referred to as consecutive continuous domains. Since it is possible to have structures with missing residues lying in the intervening domains, we imposed a criterion on the length of inter-domain linkers of such structures to consider them as consecutive domains. Based on the observed minimum CATH domain length, we set the length cut-off for inter-domain linker to a maximum of 13 residues for structures with missing residues between domains. This strict criterion would ensure that at least there is no intervening CATH domain in structures with missing residues. This process resulted in a redundant set of 2411 proteins. From this, we prepared a list of 1511 non-redundant (at 40% sequence identity at domain level) consecutive continuous domain dataset (domain-CC-2) using the procedure described later in the methodology section that ensures that at least one domain is non-redundant at 40% sequence identity.

Next, set A was used to obtain PDB entries where one or both domains are discontinuous *i*.*e*. a domain is composed of more than one segment. Since the discontinuous domain consists of non-contiguous sequences, it will pose a problem in the sequence alignment while constructing a non-redundant dataset. To address this issue, we analyzed coverage and length distribution of segments in discontinuous domains. This showed that many discontinuous domains (63%) have a major segment (more than 70%), which contributes to the domain structure. Thus, we chose to represent discontinuous domain with one major segment. For this, we empirically imposed following conditions to select the major segment of a domain: a) it should be the longest segment with a minimum length of 100 residues and ≥ 50% coverage of total discontinuous domain length, b) it should contribute at least 20 residues for domain interaction interface (Fig 1). Based on these, we obtained a total 851 structures, which were made non-redundant at 40% sequence identity for at least one domain using the procedure described later in the methods section. This dataset is referred to as domain-CD-2, which consists of 512 proteins.

To investigate the effect of the intervening domain/s on the domain interaction interface, we used proteins having at least three classified domains (set B). From this, we constructued two sub-datasets: a) consecutive continuous domain pairs and b) all combinations of consecutive and non-consecutive continuous domain pairs. The non-consecutive domain pairs have at least one intervening domain between two domains. We followed the same procedure to construct consecutive domain pairs, as described before, that resulted in a set of 1113 domain pairs. These were used to construct non-redundant dataset at 40% sequence identity at domain level that resulted in 759 domain pairs (domain-CC-M). For the second set, we made all possible combinations of interacting domain pairs for a given PDB entry that resulted in a total of 1553 domain pairs (Fig 1). These were made non-redundant at 40% sequence identity that resulted in 1046 list of domain pairs for consecutive/non-consecutive continuous (domain-CU-M) respectively. S1 Table lists PDB entries as well as their CATH classification for each dataset.

### Generation of non-redundant DDI

Full-length protein sequences are usually used for generating non-redundant DDI datasets. However, such non-redundant dataset does not have information of non-redundancy available at the level of domains because domain boundaries are not used as an input for alignment or extracting words as in case of CD-HIT [43]. Moreover, such approach of using full-length sequence cannot be used for non-consecutive domains, as these are two distinct regions of the protein sequence. In order to generate non-redundant dataset at the level of domains, we have designed a simple method, which considers non-redundancy at the level of domains and ensures that at least one domain has the minimum desired non-redundant level.

In this procedure, first CD-HIT [43] is used to cluster all domains in a given dataset at 40% sequence identity. This step results in clusters having domain entries, which are PDBID followed by chain identifier with their respective domain numbers from CATH. Each cluster is numbered from 1 to N, where N is the number of clusters. Next, we generate combinations of clusters *i = (1 to N)* and *j = (1 to N)* such that cluster numbers *i < j* (*i* and *j* are cluster numbers). For each such combination of clusters, first the common PDB entries having different domain numbers between two clusters are identified. Then, depending on the number of structures (zero, one or more) identified in previous step following is performed: a) if there are zero common cluster members (structures), then no domain pair structure is selected; b) if only one common entry exists, then it is taken as representative structure; and c) if there is more than one common PDB entry, then a representative non-redundant structure is selected that has the highest (best) resolution with the longest length. The overview of this procedure is shown in supporting Figure A in S1 File with additional details mentioned in Appendix A in S1 File. Importantly, the order of domains is maintained while selecting the representative in the dataset *i*.*e*. if the order of domains for two common PDB entries is in reversed order, both domain pairs are to be considered in the non-redundant dataset.

### Protein-protein interaction dataset

To prepare protein-protein interaction dataset or inter-chain domain interactions, we took 17659 heteromers protein-protein interaction dataset from previous work [44]. From this dataset, we considered interacting proteins with only a unique chain order and removed discontinuous protein domains. Further, this dataset was curated based on the criteria given below. First, we selected interacting protein structures with resolution ≤ 2.5Å and have CATH domains defined for both proteins (1366). Next, if a multidomain protein is involved in PPI, then we identify interacting domain pairs between two proteins using iAlign criteria, as has been given in the previous section. From this set, we only considered protein pairs in PPIs having valid interacting domains between two proteins. We performed this additional step because we need to compare the fold of respective domains in the process to find the best match of the query domain-domain interface. Thus, we obtained a total of 1464 interacting inter-chain domain pairs in 1233 PPIs.

### Interface alignment using iAlign

iAlign (interface alignment) program was developed for aligning protein-protein interfaces [30]. It essentially performs structural alignment of residues at the interfaces to detect their geometrical similarity. Since iAlign does not align individual proteins involved in PPI to detect similar interfaces, it can find structurally similar interfaces among all PPIs. We used iAlign version 1.0b7 for the structural alignment of domain-domain interfaces assuming each domain is equivalent to a protein in PPI. The similarity between interfaces is quantified using IS-score. Here, IS-score includes both geometric match score and conservation of the contact pattern between interfaces. IS-score is given by the equation:

$$\text{IS}-\text{score}=\left(\text{S}+{\text{s}}_{0}\right)/\left(1+{\text{s}}_{0}\right),$$

where

$$S=\frac{1}{L_{Q}}max\left[\sum_{i=1}^{Na}f_{i}/(1+\frac{d_{i}^{2}}{d_{0}^{2}})\right],$$

where, *L*_*Q*_ is the length of query interface; *N*_*a*_ is alignment length between query and template; d_i_ is distance (in Å) between Cα residue of aligned pairs; *f*_*i*_ is contact overlap defined by *f*_*i*_ ≡ (*c*_*i*_/*a*_*i*_ + *c*_*i*_/*b*_*i*_)/2; where *a*_*i*_ and *b*_*i*_ are number of interfacial contacts of template and query interfaces at *i*^*th*^ position in the alignment respectively, and *c*_*i*_ is number of overlapping interfacial contacts at the same *i*^*th*^ position; *d*_*0*_ is given by

d0≡{1.24(LQ-151/3-1.8Forsequentialalignment0.7(LQ-15)1/3-0.1Fornon-sequentialalignment

The length independent score *S* is obtained by normalizing it by *s*_*0*_, which is given by $s_{0}\equiv 0.18-0.35/{L_{Q}}^{0.3}$. The normalized *S* score is referred to as IS-score, which has the maximum score of one alignment between two identical structures [30]. The p-value for IS-score was calculated using distribution of interface scores from random protein-protein complexes [30]. This found that statistically significantly similar interfaces are those with IS-score having p-value < 0.05 and suggests that two aligned interfaces are similar and has some biological relevance [30].

We performed the non-sequential alignment of domain-domain interfaces using iAlign. Apart from IS-score, the fraction of aligned residues (f_res_) and the fraction of aligned contacts (f_con_) are also reported for each aligned domain interaction pair. Here, f_res_ is the ratio of the number of aligned residues over the number of interfacial residues of query DDI. Similarly, f_con_ is the ratio of the number of aligned contacts by total contacts of query interface.

## Results

In our study, we used CATH structural domains [4] and used interatomic contacts to define interfacial residues, which are those having at least one heavy atom of a domain within distance of 4.5 Å of a heavy atom from another domain [30,45]. All interfacial residues together constitute the domain-domain interface for a domain pair. The consecutive/non-consecutive domains interfacial residues are structurally aligned using non-sequential mode of iAlign [30]. Further, the best DDI is identified for each interface in the non-redundant dataset as the one with the maximum IS-score. Below, we describe similarity among intra-chain domain interfaces followed by the comparison of intra-chain and inter-chain domain interfaces.

### Similarity among intra-chain domain interfaces

We have constructed four datasets to study the affect of various spatial constraints between intra-chain domain interfaces. The results of these are discussed below:

#### a. Consecutive continuous domain-domain interfaces

As mentioned before, only one inter-domain linker separates consecutive continuous domains. In this analysis of consecutive continuous DDIs, we have used two non-redundant consecutive and continuous domains datasets *viz*. domain-CC-2 (1511 structures) and domain-CC-M (759 structures). The motive of using domain-CC-M dataset is to investigate whether interface properties of consecutive domains in proteins having more than two domains are affected by the presence of other domains.

Since our objective is to detect similar interfaces formed by domain pairs without any significant structural or sequence relationship, a list of structurally dissimilar proteins was generated for each member of domain-CC-2. Taking each member as query, it is searched against the rest other members of the dataset to identify structurally unrelated domain pairs based on the following conditions: a) has no domain within same CATH topology (fold), b) with no domain sequence having significant sequence similarity (PSI-BLAST [46], E-value > 1) and c) shares no significant structural similarity (structures were aligned using TM-align [47]) as assessed by TM-score [48], *i*.*e*. for all combinations of domains, TM-score < 0.4 [39,47,49]. Thus, a list of structurally dissimilar domain pairs was obtained that served as an individual template library for searching each query DDI. The interface similarity is measured using IS-score, which is normalized by the length of query DDI interfacial residues (*see* Methods).

**Fig 2:**
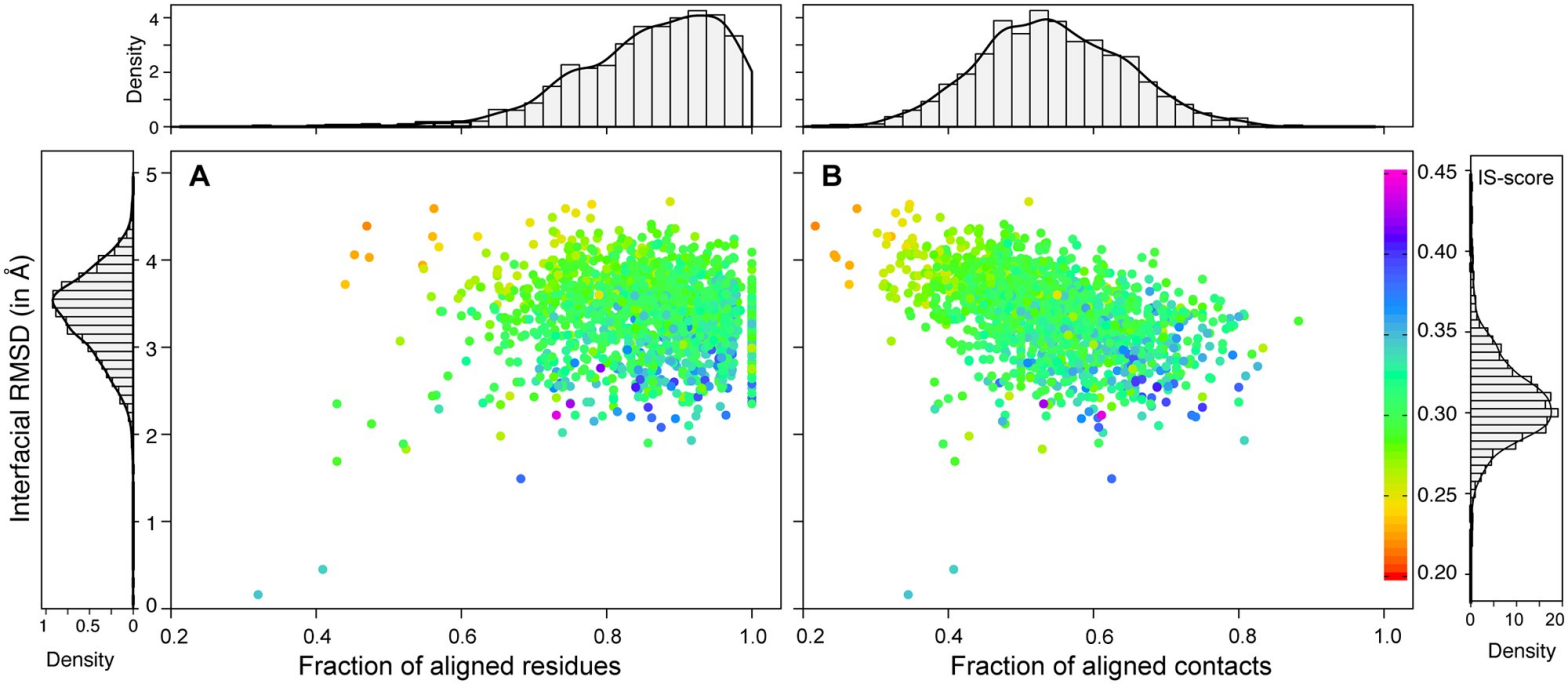
Plots of the best interface matches for Domain-CC-2 dataset. Scatter plot of the interfacial RMSD versus (A) fraction of aligned residues (f_res_) and (B) fraction of aligned contacts (f_con_) for the closest match of 1511 domain-domain interfaces extracted from proteins having only two CATH classified structural domains. Each point is represented using color gradient based on IS-score. Histogram and density plots of RMSD, f_res_, f_con_ and IS-score are shown surrounding main scatter plot.

The result of the closest interfacial match for each 1511 domain interfaces, as assessed by the best IS-score is shown in Fig 2 and the search statistics are summarized in Table 1. The mean (standard deviation (SD)) IS-score of the best interfacial similarity is 0.307 (0.026), which is higher compared to the mean (SD) IS-score of 0.207 (0.036) for the best matches among random PPI interfaces [30]. This indicates that the structural relationship between domain-domain interfaces is not random. Importantly, ~88% of these interfaces have the best structurally similar interface with a significant IS-score (p-value <0.05) suggesting these are probably biologically relevant. These have mean (SD) RMSD of 3.3 (0.5) Å, a mean (SD) residue coverage f_res_ of 86% (9%), and a mean (SD) contact coverage f_con_ of 55% (9%). The average residue and contact coverage were calculated with respect to the query DDI. Thus, suggesting that for most intra-chain domain interfaces one could find structurally similar interface even though interfaces are formed of structurally unrelated domains. This interface property is similar to the one observed previously in protein-protein interfaces [39].

**Table 1:** Summary statistics of the best similar interfaces for datasets.

Dataset	Mean (SD) of				Significant matches
	IS-score	RMSD	Residue coverage	Contact coverage	
Domain-CC-2 (1511)	0.307 (0.026)	3.3 (0.5)	86% (9)	55% (9)	88%
Domain-CC-M (759)	0.298 (0.029)	3.2 (0.5)	86% (10)	55% (10)	74%
Domain-CU-M (1046)	0.30 (0.027)	3.3 (0.5)	86% (10)	55% (10)	78%
Domain-DC-2 (512)	0.286 (0.02)	3.5 (0.3)	83% (10)	51% (8)	64%

Next, we searched for similar interfaces among structurally unrelated domain pairs for each of 759 intra-chain domain pairs (domain-CC-M), which are from multidomain proteins having more than 2 domains. Using similar criteria as described for domain-CC-2, we obtained structurally dissimilar domain pairs for each member of domain-CC-M and searched these for similar interfaces using iAlign. The results of the best match of similar interfaces for 759 proteins are summarized in Figure B in S1 File. As has been observed with domain-CC-2, similar domain interfaces could be identified among structures without having any structural similarity to the query DDI. The match statistics are summarized in Table 1. The mean (SD) IS-score is 0.298 (0.029) and the best match interfaces of ~74% have significant IS-score (p-value <0.05). The statistically significant interface pairs have a similar mean as in other datasets. These show that similar interfaces could be found among structurally unrelated domain pairs consisting of consecutive continuous domains suggesting structural degeneracy of interfaces. Importantly, these are observed for interfaces formed by consecutive domains, which can be constrained in their intra-chain domain interactions due to inter-domain linkers. Thus, implying towards a possibility that the interfaces are primarily important in intra-chain domain interactions and inter-domain linker lengths may vary to facilitate the formation of these interfaces.

**Fig 3:**
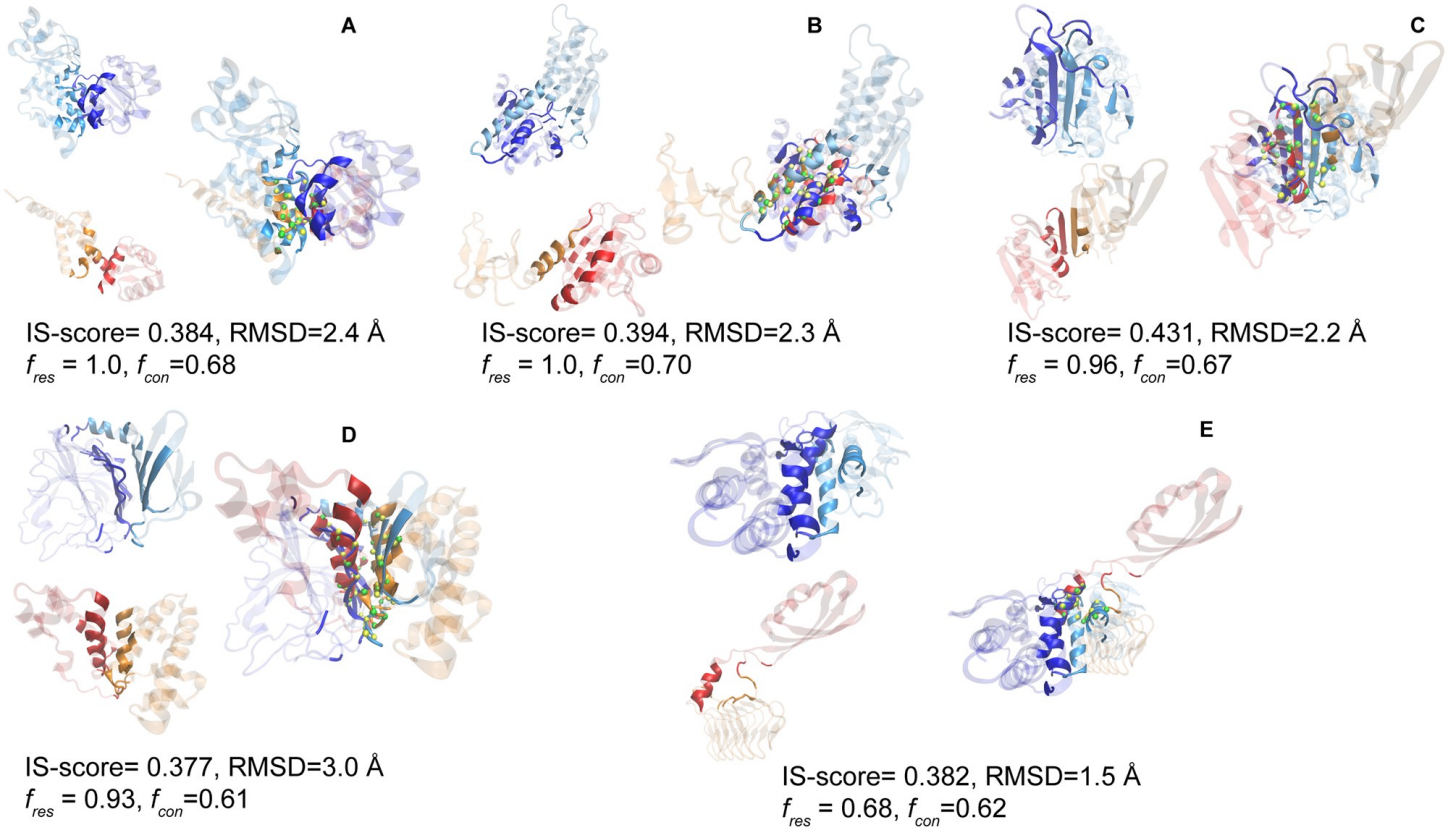
Examples of similar intra-chain domain-domain interface pairs. Two domains of template protein are shown in cyan and blue colors, while target protein domains are shown in orange and red colors. The aligned C-alpha residues are shown in green and yellow for target and template structures respectively. A) Periplasmic receptor CeuE (domains 1 and 2 of 4inoA) and manganese transport regulator (MNTR) protein (domains 1 and 2 of 2f5fB), PDB identifier is followed by the chain identifier. B) RhoA-dependent invasion protein (domains 1 and 2 of 4ldrB) and Peroxiredoxin protein (domains 1 and 2 of 2v2gA). C) Pullulanase enzyme (domains 3 and 4 of 2fh8A) and DNA polymerase sliding clamp (domains 1 and 2 of 4tr8B). D) Hyaluronate lyase enzyme (domains 1 and 3 of 1n7oA) and serum albumin (domains 5 and 6 of 4f5uA). E) Thermolysin (domains 1 and 2 of 4n4eE) and tetrahydropicolinate succinyltransferase (domains 1 and 2 of 3r8yB). The coordinates of structures were obtained from the PDB. In superposed structures, the interface and non-interface regions are shown in solid and transparent color respectively. Molecular images are generated using VMD [52].

It has been shown previously that protein-protein interaction interfaces are structurally degenerate mostly because of functional constraint, physical constraint due to the packing of compact hydrogen bonded secondary structure elements, and the interfaces are mostly flat [39]. Using these presumptions, similarities among DDIs could be explained due to packing of secondary structure and flat surfaces. However, we could not find similar domain interfaces due to functional constraints. The limited ways of packing secondary structures are found mostly to be the reason for degeneracy. Moreover, multidomain proteins are mostly globular and the packing of secondary structure elements from two domains may contribute to overall protein stability [11]. The examples of packing of α-helices and/or β-strands are shown in Fig 3A and 3C. The DNA polymerase III beta sliding clamp protein (4tr8B) consists of 3 topologically equivalent domains of α/β class that has anti-parallel helices bracketing the four-stranded anti-parallel β-sheet [50]. Two such anti-parallel β-sheets of consecutive domains interact to form an extended β-sheet and two parallelly oriented β-strands mainly constitute the domain interface of beta sliding camp proteins (Fig 3C). The pullulanase enzyme (2fh8A) has four domains. Of these, the first two and last domain belongs to Immunoglobulin-like fold (all β-class) and rest one domain adopts TIM barrel fold (α/β class) [51]. The β-sheets from first two domains come together in a parallel β-strand orientation to form the interface domain interface (Fig 3C). As shown in interface alignment of 4tr8B and 2fh8A (Fig 3C), the first two consecutive domain interfaces of DNA polymerase III beta sliding clamp and pullulanase are similar due to the packing of parallel β-strands, even though domains belonging to different CATH classes.

The flat interfaces have been observed in PPI that can easily show geometrical similarity and more so in the non-sequential alignment of interfaces. The investigation of domain interface alignments showed that these are also rather flat (Figure C in S1 File) and observed similar interfaces between domain pairs having different secondary structure elements at the interfaces (Fig 3D and 3E). The interface of domains 1 and 3 of hyaluronate lyase enzyme (1n7oA) that belongs to mainly beta class aligns with mainly helical domains (5 and 6) of serum albumin (4f5uA) as shown in Fig 3D. Most cases of interface alignment having non-significant IS-score are due to one of the domains enveloping other domain and in some case interaction interface is generally small comprising mostly of loops.

#### b. Consecutive/non-consecutive domain-domain interfaces

**Fig 4:**
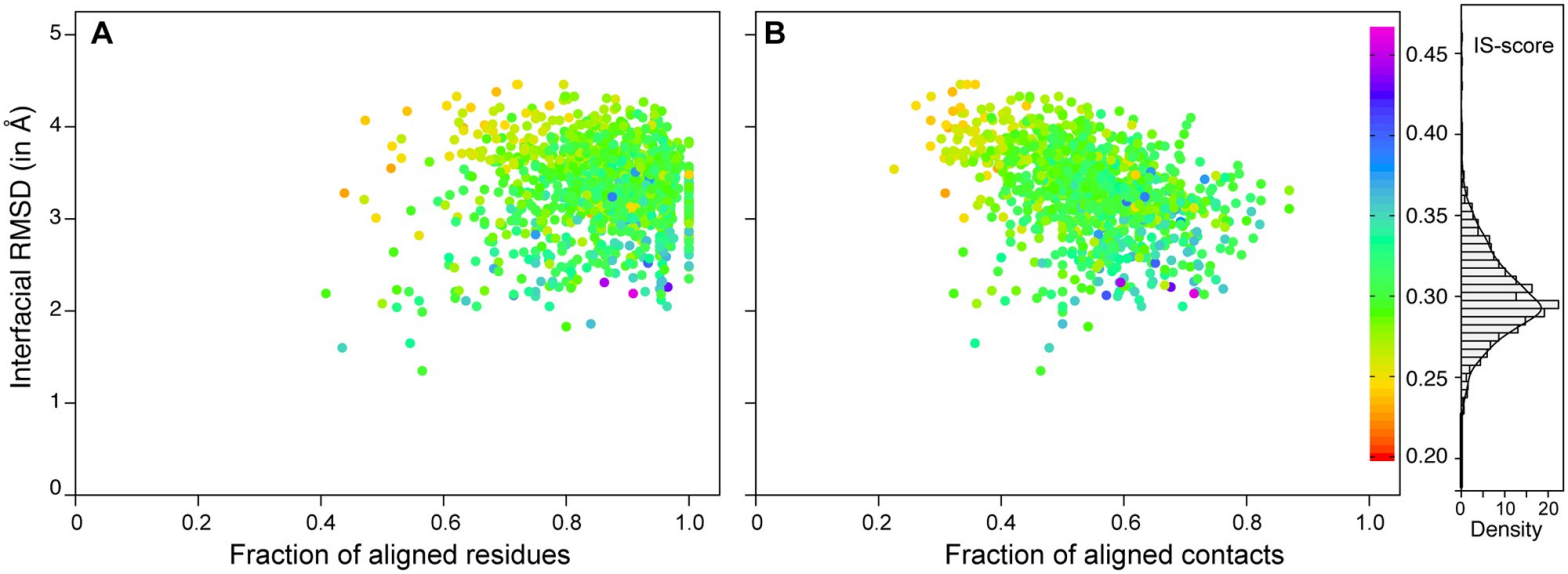
Scatter plot of the best interface matches for domain-CU-M dataset. Scatter plot of interfacial RMSD versus (A) fraction of aligned residues (f_res_) and (B) fraction of aligned contacts (f_con_) for the closest match of 1046 domain-domain interfaces extracted from proteins having > 2 CATH structural domains. Distribution of IS-score is shown as histogram.

Having shown degeneracy for consecutive domains in multidomain proteins, the same is extended to non-consecutive domains, which will have little or no restraints imposed by inter-domain linkers like consecutive domains. Here, we prepared a list of 1046 (domain-CU-M) non-redundant domain pairs, which consists of both consecutive /non-consecutive (282) domains. We used previously mentioned procedure to find the best domain interface match for each of 1046 domain pairs. The distribution of various parameters for the best match of interfaces is shown in Fig 4 and match statistics are summarized in Table 1. The mean IS-score of the best interface match is 0.30 and ~78% of interface match pairs have statistically significant IS-score. Of 282 non-consecutive domain pairs, ~76% of domains have significantly similar interfaces (p-value <0.05) with mean (SD) IS-score 0.30 (0.026). The visual inspection of non-significant alignments showed that some of these have one of the domains enveloping surface of other domains.

#### c. Discontinuous domain-domain interfaces

The structural domain can be composed of more than one segment (linear region of sequence) that is known as the discontinuous domain. Since two or more sequence segments form discontinuous domains, these have more than one inter-domain linker. Thus, we investigated whether discontinuous domains also show structural degeneracy as observed for interfaces of continuous domains. The issue with studying discontinuous domains is that it is not trivial to construct non-redundant dataset. Therefore, we exploited the observation that most discontinuous domains usually have one long segment, which contributes maximally to the structural domain and derived empirical criteria for representing discontinuous domains by only one segment (*see* Methods). Following this approach, we prepared a list of 512 non-redundant interacting domain pairs (domain-DC-2).

We followed the same procedure as has been described previously to find the best structural matches for discontinuous domain interfaces. The summary statistics of the best matches and their score distributions are shown Table 1 and supporting Figure D in S1 File respectively. The mean (SD) IS-score for best interface match for discontinuous domains is 0.286 (0.02), which is more than random IS-score of 0.207. Of these, ~64% of domain pairs have statistically significant interface similarity (p-value <0.05). This comparison for discontinuous domains suggests that degeneracy is a general feature of DDIs interfaces.

### Similarity between intra-chain and inter –chain domain-domain interfaces

In the previous sections, the intra-chain DDIs of consecutive/non-consecutive continuous or discontinuous domains were analysed and it was observed that DDIs show similar properties of degeneracy as seen in PPIs interfaces [39]. Since DDIs and PPIs also share physicochemical properties [53], we studied whether interface degeneracy is observed when inter-chain and intra-chain domain interfaces are compared to each other.

**Fig 5:**
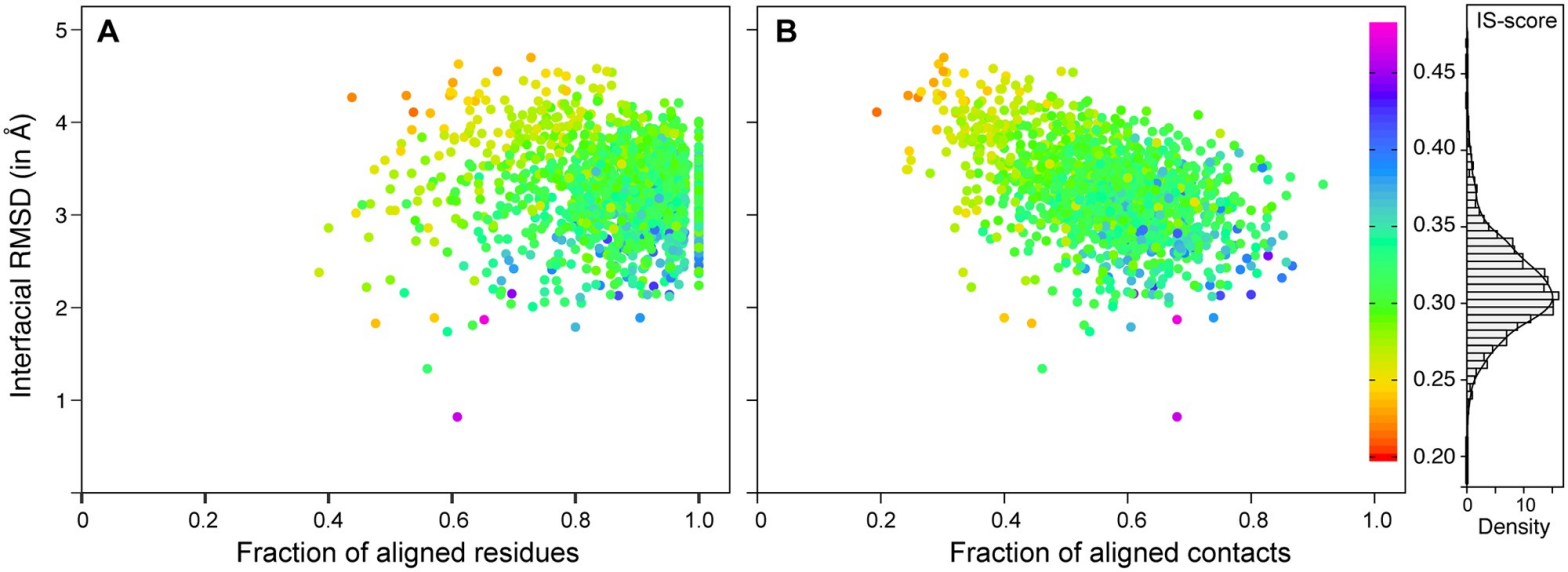
Structural comparison of inter-chain domain and intra-chain domain interfaces. Scatter plot of interfacial RMSD versus (A) fraction of aligned residues (f_res_) and (B) fraction of aligned contacts (f_con_) for the closest match of 1464 protein-protein interfaces with intra-chain domain interfaces. Distribution of IS-score is shown as histogram.

For this analysis, we took a non-redundant protein-protein interaction (PPI) dataset from previous work [44] and it was pruned to remove structures without CATH domain assignments. Further, PPIs having valid interfaces formed by interacting domains (inter-chain domains) from two monomers were taken to construct inter-chain domain interface from PPI dataset (*see* Methods). We followed procedure as has been mentioned before for searching the structural match of an inter-chain domain interface (target-PPI) in the template library of the DDIs (template-which consists of domain-CC-2, domain-CC-M, and domain-CU-M). The best structural match of a protein-protein interface (target PPI) is the one with the highest IS-score among DDIs. Fig 5 shows distribution of various parameters for the best match of interfaces. The mean (SD) IS-score of the closest match of inter-chain interface with intra-chain domain interface is 0.311 (0.031). Among these, ~86% of protein-protein interfaces have the best matches with statistically significant IS-score (p-value <0.05). These significant domain pairs have interface average RMSD (SD) of 3.2 Å (0.45), a mean (SD) residue coverage f_res_ of 88% (10%), and a mean (SD) contact coverage f_con_ of 58% (10%), respectively. Thus, showing that interfaces of inter-chain domains are similar to intra-chain domain interfaces despite having no similarity at the level of domains.

**Fig 6:**
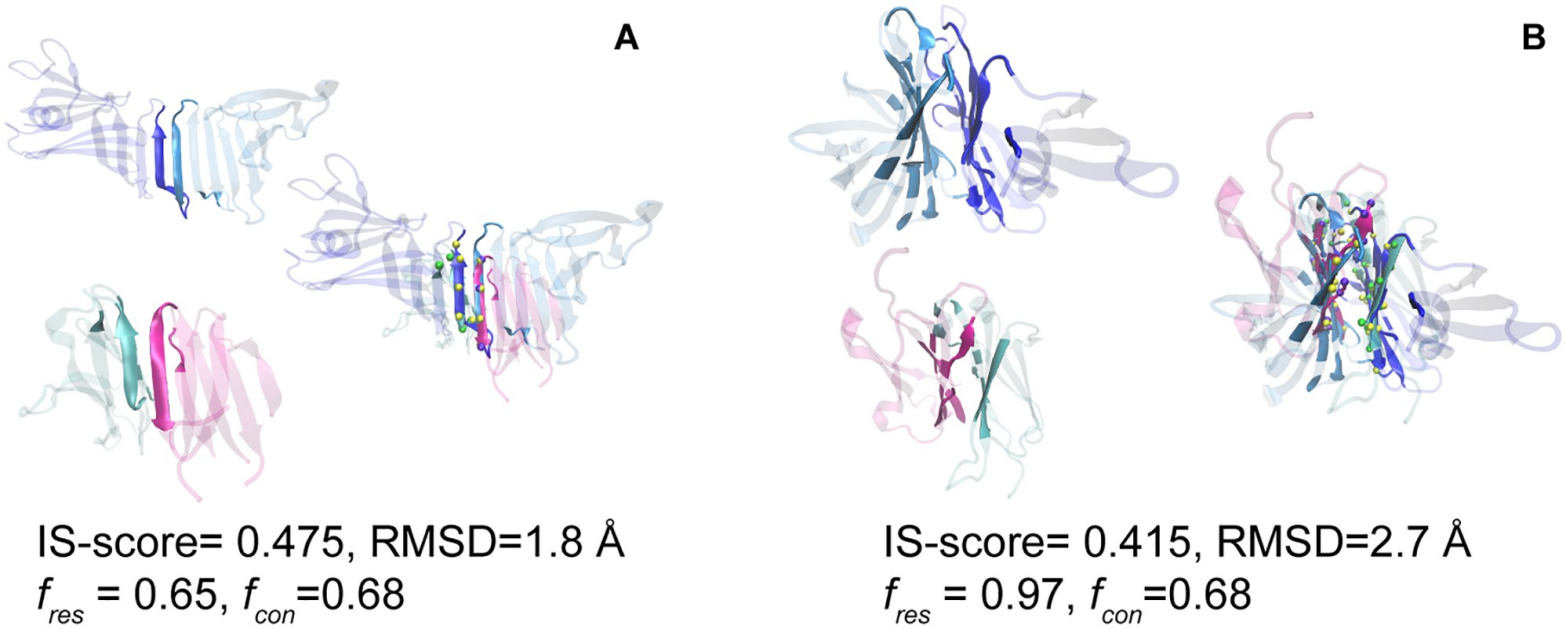
Examples of similar domain-domain and protein-protein interfaces. Two domains of template (domain-domain interface) protein are shown in blue and sky blue colors, while two interacting proteins are shown in pink and cyan colors. The aligned C-alpha residues are shown in green and yellow for target and template structures respectively. A) Two domains of outer surface protein A (domains 1 and 2 of 1ospO) is aligned with human major histocompatability complex with T-cell receptor (4pjeC (domain C02/ 4pjeE (domain E01). B) Structural interface alignment of protein of unknown function (domains 1 and 2 of 2o62A) is complexed with antibody fragments (3qnzB (domain B02)/3qnzA (domain A02). The coordinates of structures were obtained from the PDB. In superposed structures, the interface and non-interface regions are shown in solid and transparent color respectively. Molecular images are generated using VMD.

As has been reasoned before, it was found that intra-chain and inter-chain domain interfaces are structurally similar mostly because of similar packing of secondary structures as well as flat interfaces. To illustrate interface structural similarity we have shown two examples. First example shows a noticeable overlap between the PPI of 4pjeC/E [formed by major histocompatibility complex class I protein (domain C02) and domain 1 of T-cell receptor] and the DDI of ospA [outer surface protein A with two domains having antiparallel β-sheet topology], which consists of similar antiparallel β-strands, that is detected in the interface alignment (Fig 6A). Another similar structural interfaces, between packed anti-parallel β-sheets, which belong to the β-sandwich scaffold, have also been demonstrated between intra-chain domains of 2o62A and inter-chain domains of 3qnzB and 3qnzA (Fig 6B). These examples clearly manifest that interfaces of intra-chain domain interfaces share a definite structural similarity to inter-domain interfaces despite having no structural relatedness at the level of individual domains, which may be due to possible congruent secondary structure packing and presence of flat interfaces.

Since intra- and inter- chain domain interfaces are degenerate, we investigated whether merging inter-chain domain interfaces data with intra-chain domain interfaces in the template library could increase the number of significant structural matches for DDIs. For this, the closest structural neighbour search was conducted for consecutive continuous domain interfaces (1511) against three different template libraries. These three different template libraries were: a) inter-chain (1464 interfaces of PPI), b) intra-chain (1511 interfaces of DDI), and c) mixed set of both inter- and intra-chain domains (2975 interfaces of PPI and DDI). The closest match of 1511 interfaces against 1464 PPI resulted in mean (SD) IS-score of 0.304 (0.027) having ~85% of significant matches, where as, in a combined dataset of both intra-chain and inter-chain domain interfaces resulted in average (SD) IS-score of 0.314 (0.027). This is statistically significantly (p-value << 0.001 in the paired t-test) different than the mean IS-score of 0.304 and 0.307 obtained from searching against inter-chain and intra-chain domain interfaces respectively. Interestingly, the number of significant IS-score matched interfaces also increased from ~85 (88) for inter (intra)-chain to 93%. Therefore, it becomes quite evident that enriching the intra-chain template library with inter-chain domain interfaces can certainly assist in identifying more structurally similar interfaces for intra-chain domain interfaces that would not be possible while using only DDI template library derived from multidomain proteins.

### Connectivity of DDIs interface structural space

Next, we investigated the connectivity of domain-domain interface structural space using a directed graph, which is described by domain interfaces as vertices and a directed edge that points from template to query (target) interfaces drawn based on a predefined IS-score threshold with a path length of one. Here, the directed edge was considered because IS-score is not transitive and it is not same for two interfaces when target interface is changed. For instance, IS-score for A-B is not same as B-A, where B and A are target interfaces respectively. An interface I_A_ is said to be *k*^*th*^ neighbor of I_B_, if the minimum path length from node I_A_ to I_B_ is ≤ *k*. Since domain-CC-2 dataset is the largest among DDIs, we performed network analysis on digrah only for this dataset. The fraction of all possible directed pairs at a given *k*^*th*^ neighbor for varying IS-score is shown in supporting Figure E1 in S1 File. This shows that at a significant IS-score threshold of 0.26 about ~84% of all directed interface pairs are at most separated by the eighth neighbor. The largest strongly connected component (LSCC), where all nodes are connected bidirectionally to at most *k*^*th*^ neighbor consists of ~83% of interfaces at a threshold of 0.26 and k = 8 (Figure E2 in S1 File). The related size of LSCC drops drastically to ~3% at IS-score of 0.30, which probably is the critical threshold below which nodes are densely connected and structural space is continuous.

As we have found that including PPIs improve overall closely related matches for intra-chain domain interfaces, we examined whether connectivity of structural space can be improved by including inter-chain domain interfaces. For this, we utilized search results of domain-CC-2 against PPI and vice-versa to include only edges between interfaces (nodes) from PPI and DDI. The summary of all possible directed pairs at given *k* as a function of IS-score and LSCC at given IS-score as a function of k are shown in supporting Figures E3 and E4 respectively in S1 File. At IS-score threshold of 0.26, ~90% of all directed pairs are at most eighth neighbor and LSCC consists of ~89% of interfaces at k = 8. The LSCC increases by ~6% in comparison to graph without inter-chain interface connectivity. The LSCC for IS-score threshold of 0.30 is also increased to ~6%. This shows that structural space of domain-domain interface is continuous and connected, which improves by including inter-chain interfaces.

## Discussion

In the present study, we explored the structural landscape of domain-domain interfaces, by discretizing proteins as domains and structurally comparing domain-domain interfaces among structurally unrelated domains. For this, we compared intra-chain domain interfaces among consecutive/non-consecutive domains. In our analyses of domain interfaces, we found that domain-domain interfaces are structurally degenerate and are not random interfaces. The same property of interfaces has been shown for PPI interfaces. Importantly, a possible constraint imposed due to linker region between domains does not affect the general features of interfaces and during evolution of mutlidomain proteins linkers probably adapt to accommodate appropriate intra-chain domain interactions to either facilitate protein function and/or stability. The detailed investigation into understanding the basis of similar interfaces showed that similar packing of secondary structures and flat nature of domain interfaces are primarily responsible for similarity among domain interfaces. Essentially, flat interfaces can allow different interfacial secondary structural elements to be aligned in case of purely geometric matches. Thus, this study provides insight into evolution of domain interfaces.

Further, we searched for similar inter-chains interfaces within DDI interfaces and found the best structural matches between them with mean IS-score of 0.311. These show that degeneracy among domain interfaces are observed even when domains are from the same or different protein. This is most likely because of limited ways of packing secondary structure elements. The network analysis of interfaces at significant IS-score threshold showed that domain-domain interface is highly connected and continuous, which increases upon including PPI interfaces. Importantly, combining the inter-chain with intra-chain domain interfaces could enrich the interface template library that could be used for modeling either PPI or domain interfaces of multidomain proteins. The interface structural similarity among DDIs suggests toward a possibility that domain-domain interaction interface evolved from non-specific to specific interaction depending on the functional/structural significance of interfaces as it has been previously speculated for PPI interfaces [39].

## Supporting information

S1 Table List of all PDB entries from five datasets with their CATH classification. (PDF)

S1 File This contains supporting figures and appendix A. **Figure A: Overview of non-redundant dataset construction.** A Flow chart showing steps of non-redundant domain pairs dataset generation with an example. **Figure B**: **Distribution of scores for the best structural interface matches of domain-CC-M.** Scatter plot of interfacial RMSD versus (1) fraction of aligned residues (f_res_) and (2) fraction of aligned contacts (f_con_) for the closest match of 759 consecutive continuous domain-domain interfaces extracted from proteins with >2 CATH structural domains. Each point is colored based on IS-score. Distribution of IS-score is shown as histogram. The same scheme is used in Figure D. **Figure C: Comparison of interface planarity and IS-score.** Scatter plot showing relationship between planarity of domain-domain interface to the best IS-score of interface obtained for each representative 2270 consecutive domains. Planarity is measured using PRINCIP program in SURFNET [Laskowski, 1995] suit of programs that is a root-mean square deviation between interface Cα-atoms and the best fit of plane through the interface Cα-atoms. **Figure D: Distribution of scores for the best structural interface matches of domain-CU-2.** Scatter plot of interfacial RMSD versus (1) fraction of aligned residues (f_res_) and (2) fraction of aligned contacts (f_con_) for the closest match of 512 consecutive and non-continuous domains extracted from proteins with only two CATH structural domains. Each point is colored based on IS-score. Distribution of IS-score is shown as histogram. **Figure E**: **Network connectivity of DDI in structural space.** The fraction of directed pairs of nodes (interfaces), which are connected with at most *k*^*th*^ neighbor are plotted as a function of IS-score. This is shown for DDI and a combined DDI+PPI interfaces in panels (1) and (3) respectively. Here, fraction is computed as *n*_*k*_/(*N x (N-1))*, where *n*_*k*_ is the number of *k*^*th*^ neighbor pairs and *N* is total number of interfaces in a graph. The relative size of LSCC at various *k* for graphs generated at a given IS-score thresholds are shown in panels (2) and (4) for DDI and a combined DDI+PPI interfaces respectively.
(PDF)
