# Supplementary material for: Unraveling the structural landscape of intra-chain domain interfaces: Implication in the evolution of domain-domain interactions
Source: PLoS One. 2019 Aug 2;14(8):e0220336. doi: 10.1371/journal.pone.0220336 (PMC6677297; doi:10.1371/journal.pone.0220336)
Supplement: S1 Table — (PDF) [file pone.0220336.s001.pdf]

S1 Table: List of pdb entries from five dataset and their CATH classification

1511 consecutive continuous domain dataset (domain-CC-2)			
**Protein**	**Domain_pair**	**Domain1 CATH classification**	**Domain 2 CATH classification**
3mmlF	3mmlF01_3mmlF02	3.30.1360.40	2.40.100.10
3zvlA	3zvlA01_3zvlA02	3.40.50.1000	3.40.50.300
3h4lB	3h4lB01_3h4lB02	3.30.565.10	3.30.230.10
2i0mA	2i0mA01_2i0mA02	1.20.58.220	1.20.58.220
2o62A	2o62A01_2o62A02	2.40.128.20	2.40.128.20
3o08A	3o08A02_3o08A03	3.30.420.40	3.40.367.20
2id3A	2id3A01_2id3A02	1.10.10.60	1.10.357.10
4jaqA	4jaqA01_4jaqA02	3.40.47.10	3.40.47.10
3s97C	3s97C01_3s97C02	2.60.40.10	2.60.40.10
2fd5A	2fd5A01_2fd5A02	1.10.10.60	1.10.357.10
3utnX	3utnX01_3utnX02	3.40.250.10	3.40.250.10
4itxB	4itxB01_4itxB02	3.40.640.10	3.90.1150.10
2vqeE	2vqeE01_2vqeE02	3.30.160.20	3.30.230.10
4czuA	4czuA01_4czuA02	3.30.200.20	1.10.510.10
3c8dB	3c8dB01_3c8dB02	2.60.40.10	3.40.50.1820
1rzhM	1rzhM01_1rzhM02	1.20.85.10	1.20.85.10
3ik4B	3ik4B01_3ik4B02	3.30.390.10	3.20.20.120
2qq9A	2qq9A01_2qq9A02	1.10.10.10	1.10.60.10
2nruB	2nruB01_2nruB02	3.30.200.20	1.10.510.10
3sq7A	3sq7A01_3sq7A02	3.30.870.10	3.30.870.20
1ls1A	1ls1A01_1ls1A02	1.20.120.140	3.40.50.300
3d2yA	3d2yA01_3d2yA02	3.40.80.10	1.10.101.10
2wxuA	2wxuA01_2wxuA02	1.10.575.10	2.60.60.20
4aj2B	4aj2B01_4aj2B02	3.40.50.720	3.90.110.10
3pozA	3pozA01_3pozA02	3.30.200.20	1.10.510.10
2qzuA	2qzuA01_2qzuA02	3.40.720.10	3.30.1120.10
3qdkD	3qdkD01_3qdkD02	3.30.420.40	3.30.420.40
2yxnA	2yxnA01_2yxnA02	3.40.50.620	1.10.240.10
2j5cB	2j5cB01_2j5cB02	1.50.10.130	1.10.600.10
2w42A	2w42A01_2w42A02	3.40.50.2300	3.30.420.10
1mkfA	1mkfA01_1mkfA02	2.60.40.1330	2.60.40.1340
3ltoA	3ltoA01_3ltoA02	3.30.230.10	3.30.70.890
2i6lA	2i6lA01_2i6lA02	3.30.200.20	1.10.510.10
1ibjA	1ibjA01_1ibjA02	3.40.640.10	3.90.1150.10
3nwrA	3nwrA01_3nwrA02	3.30.70.150	3.20.20.110
1kq3A	1kq3A01_1kq3A02	3.40.50.1970	1.20.1090.10
1rmdA	1rmdA01_1rmdA02	3.30.40.10	3.30.160.60
1q15A	1q15A01_1q15A02	3.60.20.10	3.40.50.620
3gbxA	3gbxA01_3gbxA02	3.40.640.10	3.90.1150.10
2a19B	2a19B01_2a19B02	3.30.200.20	1.10.510.10
4bhbA	4bhbA01_4bhbA02	3.30.160.70	1.10.10.10
1kv9A	1kv9A01_1kv9A02	2.140.10.10	1.10.760.10
2hiyD	2hiyD01_2hiyD02	3.30.70.1280	3.30.70.1260
1jvnA	1jvnA01_1jvnA02	3.40.50.880	3.20.20.70
2prsB	2prsB01_2prsB02	3.40.50.1980	3.40.50.1980
4ix3B	4ix3B01_4ix3B02	3.30.200.20	1.10.510.10
4j2fA	4j2fA01_4j2fA02	3.40.30.10	1.20.1050.10
1sgvA	1sgvA01_1sgvA02	3.30.2350.10	2.30.130.10
3su6A	3su6A01_3su6A02	2.40.10.120	2.40.10.10

3cerC	3cerC01_3cerC02	3.30.420.40	3.30.420.150
1yqtA	1yqtA01_1yqtA02	3.40.50.300	3.40.50.300
4h05B	4h05B01_4h05B02	3.30.200.20	3.90.1200.10
3r5tA	3r5tA01_3r5tA02	3.40.50.1980	3.40.50.1980
3s9dD	3s9dD01_3s9dD02	2.60.40.10	2.60.40.10
3bpzA	3bpzA01_3bpzA02	1.10.287.630	2.60.120.10
3zrpA	3zrpA01_3zrpA02	3.40.640.10	3.90.1150.10
4nheB	4nheB01_4nheB02	3.40.50.720	3.30.360.10
4gqaC	4gqaC01_4gqaC02	3.40.50.720	3.30.360.10
2gu3A	2gu3A01_2gu3A02	3.10.450.40	3.10.450.40
1g8aA	1g8aA01_1g8aA02	3.30.200.20	3.40.50.150
3m84B	3m84B01_3m84B02	3.30.1330.10	3.90.650.10
4l6wB	4l6wB01_4l6wB02	3.90.226.10	3.90.226.10
2b3zD	2b3zD01_2b3zD02	3.40.140.10	3.40.430.10
3r2uA	3r2uA01_3r2uA02	3.60.15.10	3.40.250.10
4lejA	4lejA01_4lejA02	2.60.120.10	2.60.120.10
3ruxA	3ruxA01_3ruxA02	3.30.930.10	2.30.30.100
1efyA	1efyA01_1efyA02	1.20.142.10	3.90.228.10
3p86A	3p86A01_3p86A02	3.30.200.20	1.10.510.10
3pjxA	3pjxA01_3pjxA02	3.30.70.270	3.20.20.450
1ayeA	1ayeA01_1ayeA02	3.30.70.340	3.40.630.10
3k6mD	3k6mD01_3k6mD02	3.40.1080.10	3.40.1080.10
2weiA	2weiA01_2weiA02	3.30.200.20	1.10.510.10
1o69A	1o69A01_1o69A02	3.40.640.10	3.90.1150.10
1ogoX	1ogoX01_1ogoX02	2.60.350.10	2.160.20.10
3d3yA	3d3yA01_3d3yA02	3.30.830.10	3.30.830.10
2fnuB	2fnuB01_2fnuB02	3.40.640.10	3.90.1150.10
1z82A	1z82A01_1z82A02	3.40.50.720	1.10.1040.10
4lpqA	4lpqA01_4lpqA02	1.10.101.10	2.40.440.10
1fpoA	1fpoA01_1fpoA02	1.10.287.110	1.20.1280.20
3f65A	3f65A01_3f65A02	2.60.40.10	2.60.40.10
3kqfA	3kqfA01_3kqfA02	3.90.226.10	1.10.12.10
2ip2A	2ip2A01_2ip2A02	1.10.10.10	3.40.50.150
1hyoA	1hyoA01_1hyoA02	2.30.30.230	3.90.850.10
1wv3A	1wv3A01_1wv3A02	2.60.200.20	2.60.200.20
1xahB	1xahB01_1xahB02	3.40.50.1970	1.20.1090.10
3ec3A	3ec3A01_3ec3A02	3.40.30.10	3.40.30.10
3zbfA	3zbfA01_3zbfA02	3.30.200.20	1.10.510.10
1fwxA	1fwxA01_1fwxA02	2.130.10.10	2.60.40.420
1qf8A	1qf8A01_1qf8A02	1.10.1820.10	2.20.25.20
1i39A	1i39A01_1i39A02	3.30.420.10	1.10.10.460
2aplA	2aplA01_2aplA02	1.10.8.330	1.10.8.340
3unvA	3unvA01_3unvA02	1.10.275.10	1.20.200.10
1ox0A	1ox0A01_1ox0A02	3.40.47.10	3.40.47.10
1pj3A	1pj3A01_1pj3A02	3.40.50.10380	3.40.50.720
1llnA	1llnA01_1llnA02	3.40.420.10	4.10.470.10
1ia9B	1ia9B01_1ia9B02	3.30.200.20	3.20.200.10
1x9yA	1x9yA01_1x9yA02	3.10.500.10	3.90.70.10
3bfmA	3bfmA01_3bfmA02	3.30.930.10	2.30.30.100
1yqeA	1yqeA01_1yqeA02	3.40.630.50	3.40.50.10700
4acfA	4acfA01_4acfA02	3.10.20.70	3.30.590.10
4gklA	4gklA01_4gklA02	3.20.20.80	2.60.40.1180
3lrkA	3lrkA01_3lrkA02	3.20.20.70	2.60.40.1180

2hyxA	2hyxA01_2hyxA02	3.40.30.10	2.60.120.260
3nvoA	3nvoA01_3nvoA02	3.30.460.20	1.20.58.340
3ligA	3ligA01_3ligA02	2.115.10.20	2.60.120.560
1zu4A	1zu4A01_1zu4A02	1.20.120.140	3.40.50.300
2auwB	2auwB01_2auwB02	3.30.2020.10	1.10.260.40
3lm4B	3lm4B01_3lm4B02	3.10.180.10	3.10.180.10
1rzhH	1rzhH01_1rzhH02	4.10.540.10	3.90.50.10
3g17G	3g17G01_3g17G02	3.40.50.720	1.10.1040.10
2i5hA	2i5hA01_2i5hA02	2.40.50.140	1.10.150.280
4e5sC	4e5sC01_4e5sC02	3.40.50.10740	3.50.30.60
4k91A	4k91A01_4k91A02	3.40.710.10	2.60.410.10
2vosA	2vosA01_2vosA02	3.40.1190.10	3.90.190.20
2yboA	2yboA01_2yboA02	3.40.1010.10	3.30.950.10
3ubdA	3ubdA01_3ubdA02	3.30.200.20	1.10.510.10
3tlkA	3tlkA01_3tlkA02	3.40.50.1980	3.40.50.1980
3c3vA	3c3vA01_3c3vA02	2.60.120.10	2.60.120.10
2ivxA	2ivxA01_2ivxA02	1.10.472.10	1.10.472.10
4uwjA	4uwjA01_4uwjA02	3.40.630.30	3.40.630.30
2ehzA	2ehzA01_2ehzA02	3.10.180.10	3.10.180.10
2f21A	2f21A01_2f21A02	2.20.70.10	3.10.50.40
1nd4A	1nd4A01_1nd4A02	3.30.200.20	3.90.1200.10
3r0sA	3r0sA01_3r0sA02	2.160.10.10	1.20.1180.10
1ee8A	1ee8A01_1ee8A02	3.20.190.10	1.10.8.50
2pbpA	2pbpA01_2pbpA02	3.90.226.10	1.10.12.10
1n4kA	1n4kA01_1n4kA02	2.80.10.50	1.25.10.30
3w6gA	3w6gA01_3w6gA02	3.40.30.10	3.30.1020.10
4msoA	4msoA01_4msoA02	3.40.640.10	3.90.1150.10
3hhpA	3hhpA01_3hhpA02	3.40.50.720	3.90.110.10
1d8lA	1d8lA01_1d8lA02	1.10.150.20	2.40.50.140
1hr6B	1hr6B01_1hr6B02	3.30.830.10	3.30.830.10
1vcaA	1vcaA01_1vcaA02	2.60.40.10	2.60.40.10
3cymA	3cymA01_3cymA02	3.30.420.10	1.10.150.80
1gcyA	1gcyA01_1gcyA02	3.20.20.80	2.60.40.1180
4b9dB	4b9dB01_4b9dB02	3.30.200.20	1.10.510.10
4h2gA	4h2gA01_4h2gA02	3.60.21.10	3.90.780.10
3fmeA	3fmeA01_3fmeA02	3.30.200.20	1.10.510.10
4e01A	4e01A01_4e01A02	1.20.140.20	3.30.565.10
4jykA	4jykA01_4jykA02	1.10.10.60	1.10.357.10
4kemA	4kemA01_4kemA02	3.30.390.10	3.20.20.120
3e70C	3e70C01_3e70C02	1.20.120.140	3.40.50.300
3lulB	3lulB01_3lulB02	3.30.470.10	3.20.10.10
3rloA	3rloA01_3rloA02	2.40.50.140	2.40.50.140
3l41A	3l41A01_3l41A02	3.40.50.10190	3.40.50.10190
2nsmA	2nsmA01_2nsmA02	3.40.630.10	2.60.40.1120
1se8A	1se8A01_1se8A02	2.40.50.140	2.40.50.140
3gdcA	3gdcA01_3gdcA02	2.60.40.420	2.60.40.420
4mzuF	4mzuF01_4mzuF02	2.160.10.10	2.60.120.10
1jp4A	1jp4A01_1jp4A02	3.30.540.10	3.40.190.80
1vkwA	1vkwA01_1vkwA02	3.40.109.10	3.40.109.30
3dmgA	3dmgA01_3dmgA02	3.40.50.150	3.40.50.150
1kwmA	1kwmA01_1kwmA02	3.30.70.340	3.40.630.10
3hsqA	3hsqA01_3hsqA02	2.160.10.10	1.20.1180.10
3p0bA	3p0bA01_3p0bA02	3.20.110.10	1.20.1430.10

2yvkA	2yvkA01_2yvkA02	1.20.120.420	3.40.50.10470
3vprA	3vprA01_3vprA02	1.10.10.60	1.10.357.10
3rssA	3rssA01_3rssA02	3.40.50.10260	3.40.1190.20
2oasA	2oasA01_2oasA02	3.40.1080.10	3.30.750.70
2ycfA	2ycfA01_2ycfA02	3.30.200.20	1.10.510.10
3kvnX	3kvnX01_3kvnX02	3.40.50.1110	2.40.128.130
2h21B	2h21B01_2h21B02	3.90.1410.10	3.90.1420.10
1hyeA	1hyeA01_1hyeA02	3.40.50.720	3.90.110.10
3mcqA	3mcqA01_3mcqA02	3.30.1330.10	3.90.650.10
1s35A	1s35A01_1s35A02	1.20.58.60	1.20.58.60
2dfkA	2dfkA01_2dfkA02	1.20.900.10	2.30.29.30
3m0fA	3m0fA01_3m0fA02	3.40.30.10	1.20.1050.10
3fpqB	3fpqB01_3fpqB02	3.30.200.20	1.10.510.10
3bbyA	3bbyA01_3bbyA02	3.40.30.10	1.20.1050.10
3cjjA	3cjjA01_3cjjA02	2.60.40.10	2.60.40.10
4gipD	4gipD02_4gipD03	2.40.490.10	2.60.40.1690
2hevR	2hevR01_2hevR02	2.10.50.10	2.10.50.10
1vquA	1vquA01_1vquA02	1.20.970.10	3.40.1030.10
3gyrA	3gyrA01_3gyrA02	2.60.40.420	2.60.40.420
1woqA	1woqA01_1woqA02	3.30.420.40	3.30.420.40
1wlsA	1wlsA01_1wlsA02	3.40.50.1170	3.40.50.40
2yvlD	2yvlD01_2yvlD02	3.10.330.20	3.40.50.150
4jgiB	4jgiB01_4jgiB02	1.10.1240.10	3.40.50.280
1j2zA	1j2zA01_1j2zA02	2.160.10.10	1.20.1180.10
1fp2A	1fp2A01_1fp2A02	1.10.10.10	3.40.50.150
2j7pD	2j7pD01_2j7pD02	1.20.120.140	3.40.50.300
4cydB	4cydB01_4cydB02	2.60.120.10	1.10.10.10
2zxdA	2zxdA01_2zxdA02	3.20.20.80	2.60.40.1180
2a3nA	2a3nA01_2a3nA02	3.40.50.10490	3.40.50.10490
1h3xA	1h3xA01_1h3xA02	2.60.40.10	2.60.40.10
4itjB	4itjB01_4itjB02	3.30.200.20	1.10.510.10
3nvwB	3nvwB02_3nvwB03	3.30.465.10	3.30.390.50
3qfwB	3qfwB01_3qfwB02	3.30.70.150	3.20.20.110
1k8wA	1k8wA01_1k8wA02	3.30.2350.10	2.30.130.10
3vrdA	3vrdA01_3vrdA02	1.10.760.10	1.10.760.10
2evaA	2evaA01_2evaA02	3.30.200.20	1.10.510.10
3tqsA	3tqsA01_3tqsA02	3.40.50.150	1.10.8.100
3lszA	3lszA01_3lszA02	3.40.30.10	1.20.1050.10
1sznA	1sznA01_1sznA02	3.20.20.70	2.60.40.1180
3rgaA	3rgaA01_3rgaA02	3.10.450.50	3.10.450.50
2jksA	2jksA01_2jksA02	2.60.40.1320	2.60.40.1320
3nivA	3nivA01_3nivA02	3.40.30.10	1.20.1050.10
3g2fA	3g2fA01_3g2fA02	3.30.200.20	1.10.510.10
2oyoA	2oyoA01_2oyoA02	1.20.5.810	1.20.1290.10
3zylA	3zylA01_3zylA02	1.25.40.90	1.20.58.150
1q95H	1q95H01_1q95H02	3.30.70.140	2.30.30.20
2gpjA	2gpjA01_2gpjA02	2.40.30.10	3.40.50.80
4euuA	4euuA01_4euuA02	3.30.200.20	1.10.510.10
2h6dA	2h6dA01_2h6dA02	3.30.200.20	1.10.510.10
1h3iA	1h3iA01_1h3iA02	2.20.110.10	2.170.270.10
1sumB	1sumB01_1sumB02	1.20.58.220	1.20.58.220
4bguA	4bguA01_4bguA02	3.40.50.720	3.90.110.10
3efmA	3efmA01_3efmA02	2.170.130.10	2.40.170.20

3mybA	3mybA01_3mybA02	3.90.226.10	1.10.12.10
2dplA	2dplA01_2dplA02	3.40.50.620	3.30.300.10
1y14D	1y14D01_1y14D02	3.30.1490.120	2.40.50.140
2z1aA	2z1aA01_2z1aA02	3.60.21.10	3.90.780.10
2j5vB	2j5vB01_2j5vB02	3.40.1160.10	2.30.130.10
3tytA	3tytA01_3tytA02	3.30.70.330	3.30.70.330
1bcoA	1bcoA01_1bcoA02	3.30.420.10	2.30.30.130
3be6A	3be6A01_3be6A02	3.40.50.1980	3.40.50.1980
4h03A	4h03A01_4h03A02	3.90.176.10	3.90.176.10
1gkmA	1gkmA01_1gkmA02	1.10.275.10	1.20.200.10
1sauA	1sauA01_1sauA02	3.30.1420.10	1.10.10.370
2pgxA	2pgxA01_2pgxA02	3.40.1630.10	3.40.50.150
1bl0A	1bl0A01_1bl0A02	1.10.10.60	1.10.10.60
1seiA	1seiA01_1seiA02	3.30.1370.30	3.30.1490.10
1kw3B	1kw3B01_1kw3B02	3.10.180.10	3.10.180.10
2bc4C	2bc4C01_2bc4C02	3.10.320.10	2.60.40.10
2o1sB	2o1sB02_2o1sB03	3.40.50.970	3.40.50.920
4igkA	4igkA01_4igkA02	3.40.50.10190	3.40.50.10190
3lo8A	3lo8A01_3lo8A02	2.40.30.10	3.40.50.80
4mdyA	4mdyA01_4mdyA02	3.40.50.1980	3.40.50.1980
2rb9D	2rb9D01_2rb9D02	3.30.1330.10	3.90.650.10
3hx3A	3hx3A01_3hx3A02	1.10.8.20	3.40.525.10
1a8lA	1a8lA01_1a8lA02	3.40.30.10	3.40.30.10
2aprA	2aprA01_2aprA02	2.40.70.10	2.40.70.10
3e0mC	3e0mC01_3e0mC02	3.30.1060.10	2.170.150.20
3tl2A	3tl2A01_3tl2A02	3.40.50.720	3.90.110.10
2zgiD	2zgiD01_2zgiD02	3.30.470.10	3.20.10.10
4f80A	4f80A01_4f80A02	2.60.40.10	2.60.40.10
3plnA	3plnA01_3plnA02	3.40.50.720	1.20.5.100
3c24A	3c24A01_3c24A02	3.40.50.720	1.10.3640.10
4wsjC	4wsjC01_4wsjC02	3.20.20.80	2.60.40.1180
2i6hA	2i6hA01_2i6hA02	1.20.58.320	1.25.40.10
1tedA	1tedA01_1tedA02	3.40.47.10	3.40.47.10
3ohsX	3ohsX01_3ohsX02	3.40.50.720	3.30.360.10
2essA	2essA01_2essA02	3.10.129.10	3.10.129.10
2qytA	2qytA01_2qytA02	3.40.50.720	1.10.1040.10
1eerC	1eerC01_1eerC02	2.60.40.10	2.60.40.10
3k9oA	3k9oA01_3k9oA02	3.10.110.10	1.10.8.10
3pdiD	3pdiD01_3pdiD02	3.40.50.1980	3.40.50.1980
2izzB	2izzB01_2izzB02	3.40.50.720	1.10.3730.10
3ct4A	3ct4A01_3ct4A02	3.40.50.10440	3.30.1180.20
4q1tB	4q1tB01_4q1tB02	3.40.1160.10	2.30.130.10
1ihoA	1ihoA01_1ihoA02	3.40.50.620	3.30.1300.10
1oheA	1oheA01_1oheA02	3.90.190.10	3.90.190.10
4giuA	4giuA01_4giuA02	1.20.970.10	3.40.1030.10
3rk1B	3rk1B01_3rk1B02	3.40.50.620	3.90.1490.10
3sllB	3sllB01_3sllB02	3.90.226.10	1.10.12.10
2z16A	2z16A01_2z16A02	1.20.91.10	1.10.10.180
4hadA	4hadA01_4hadA02	3.40.50.720	3.30.360.10
2v3zA	2v3zA01_2v3zA02	3.40.350.10	3.90.230.10
1a79A	1a79A01_1a79A02	3.40.1350.10	3.40.1170.20
1fjrA	1fjrA01_1fjrA02	2.30.160.11	2.170.180.11
3edpA	3edpA01_3edpA02	1.10.10.10	3.40.1410.10

2fb5A	2fb5A01_2fb5A02	1.10.287.770	3.40.1700.10
3h9yB	3h9yB01_3h9yB02	2.60.40.10	2.60.40.10
3qxzA	3qxzA01_3qxzA02	3.90.226.10	1.10.12.10
2ppqA	2ppqA01_2ppqA02	3.30.200.20	3.90.1200.10
4egeA	4egeA01_4egeA02	3.40.350.10	3.90.230.10
2fboJ	2fboJ01_2fboJ02	2.60.40.10	2.60.40.10
3m70A	3m70A01_3m70A02	2.60.120.10	3.40.50.150
2rl0A	2rl0A01_2rl0A02	2.10.70.10	2.10.70.10
2w40B	2w40B01_2w40B02	3.30.420.40	3.30.420.40
1aw9A	1aw9A01_1aw9A02	3.40.30.10	1.20.1050.10
3dg6A	3dg6A01_3dg6A02	3.30.390.10	3.20.20.120
2bw0A	2bw0A01_2bw0A02	3.40.50.170	3.10.25.10
2ywmA	2ywmA01_2ywmA02	3.40.30.10	3.40.30.10
2o14A	2o14A01_2o14A02	2.60.120.430	3.40.50.1110
2npnA	2npnA01_2npnA02	3.40.1010.10	3.30.950.10
3lhsA	3lhsA01_3lhsA02	3.40.50.1980	3.40.50.1980
4ldrB	4ldrB01_4ldrB02	1.20.120.420	3.40.50.10470
3mwfA	3mwfA01_3mwfA02	3.40.50.1980	3.40.50.1980
3q6bA	3q6bA01_3q6bA02	3.10.20.310	3.10.20.310
3irpX	3irpX01_3irpX02	2.60.40.1280	2.60.40.1290
1zk8A	1zk8A01_1zk8A02	1.10.10.60	1.10.357.10
1a8dA	1a8dA01_1a8dA02	2.60.120.200	2.80.10.50
4bgqA	4bgqA01_4bgqA02	3.30.200.20	1.10.510.10
1kutA	1kutA01_1kutA02	3.30.200.20	3.30.470.20
4gbjC	4gbjC01_4gbjC02	3.40.50.720	1.10.1040.10
2xryA	2xryA01_2xryA02	3.40.50.620	1.25.40.80
1oi2A	1oi2A01_1oi2A02	3.40.50.10440	3.30.1180.20
3ezyA	3ezyA01_3ezyA02	3.40.50.720	3.30.360.10
1a6qA	1a6qA01_1a6qA02	3.60.40.10	1.10.10.430
2optA	2optA01_2optA02	1.10.10.60	1.10.357.10
3r0uB	3r0uB01_3r0uB02	3.30.390.10	3.20.20.120
2d69A	2d69A01_2d69A02	3.30.70.150	3.20.20.110
3h8gA	3h8gA01_3h8gA02	3.40.220.10	3.40.630.10
3c9uB	3c9uB01_3c9uB02	3.30.1330.10	3.90.650.10
4gkhF	4gkhF01_4gkhF02	3.30.200.20	3.90.1200.10
4m7oA	4m7oA01_4m7oA02	3.40.50.1980	3.40.50.1980
2blnA	2blnA01_2blnA02	3.40.50.170	3.10.25.10
2ia1B	2ia1B01_2ia1B02	3.30.500.20	1.10.10.10
3wxmB	3wxmB02_3wxmB03	3.30.420.60	3.30.1330.30
3cb6A	3cb6A01_3cb6A02	3.40.350.10	3.90.230.10
1k0dC	1k0dC01_1k0dC02	3.40.30.10	1.20.1050.10
1kb0A	1kb0A01_1kb0A02	2.140.10.10	1.10.760.10
1wlfA	1wlfA01_1wlfA02	2.40.40.20	3.10.330.10
3tjeF	3tjeF01_3tjeF02	2.10.50.10	2.10.50.10
1fepA	1fepA01_1fepA02	2.170.130.10	2.40.170.20
3fjyB	3fjyB01_3fjyB02	3.90.79.10	3.40.50.1240
3qavA	3qavA01_3qavA02	3.40.30.10	1.20.1050.10
2be3A	2be3A01_2be3A02	3.30.460.10	1.10.287.860
4e70A	4e70A01_4e70A02	1.10.10.10	3.40.50.150
2jhnA	2jhnA02_2jhnA03	1.10.340.30	1.10.1670.10
1zl0A	1zl0A01_1zl0A02	3.40.50.10740	3.50.30.60
1ywmA	1ywmA01_1ywmA02	2.60.500.10	1.20.1270.150
4ksiA	4ksiA01_4ksiA02	3.40.220.10	3.40.630.10

2izrA	2izrA01_2izrA02	3.30.200.20	1.10.510.10
1wdeA	1wdeA01_1wdeA02	3.40.1010.10	3.30.950.10
2pgeA	2pgeA01_2pgeA02	3.30.390.10	3.20.20.120
1txkA	1txkA01_1txkA02	2.70.98.10	2.60.40.10
1npyA	1npyA01_1npyA02	3.40.50.10860	3.40.50.720
2p3nA	2p3nA01_2p3nA02	3.30.540.10	3.40.190.80
1ms9B	1ms9B01_1ms9B02	2.120.10.10	2.60.120.200
2wr8A	2wr8A01_2wr8A02	3.40.50.10790	2.40.30.90
1iy9A	1iy9A01_1iy9A02	3.40.50.150	2.30.140.10
2cycA	2cycA01_2cycA02	3.40.50.620	1.10.240.10
1i7oA	1i7oA01_1i7oA02	3.90.850.10	3.90.850.10
2bmbA	2bmbA01_2bmbA02	3.30.70.560	3.20.20.20
3b9qA	3b9qA01_3b9qA02	1.20.120.140	3.40.50.300
2iw2A	2iw2A01_2iw2A02	3.40.350.10	3.90.230.10
4lcbA	4lcbA01_4lcbA02	3.40.50.300	1.10.8.60
2e5yA	2e5yA01_2e5yA02	2.60.15.10	1.20.5.440
3nsxA	3nsxA01_3nsxA02	2.60.40.1760	3.20.20.80
4i1aB	4i1aB01_4i1aB02	1.25.40.10	1.25.40.10
4eg2A	4eg2A01_4eg2A02	3.40.140.10	3.40.140.10
3hg3A	3hg3A01_3hg3A02	3.20.20.70	2.60.40.1180
1rhsA	1rhsA01_1rhsA02	3.40.250.10	3.40.250.10
4iiyB	4iiyB01_4iiyB02	3.40.50.10740	3.50.30.60
3mi9A	3mi9A01_3mi9A02	3.30.200.20	1.10.510.10
3whiA	3whiA01_3whiA02	3.30.70.80	3.40.50.200
3w5sA	3w5sA01_3w5sA02	3.40.50.1970	1.20.1090.10
3p5mB	3p5mB01_3p5mB02	3.90.226.10	1.10.12.10
1bifA	1bifA01_1bifA02	3.40.50.300	3.40.50.1240
2arhA	2arhA01_2arhA02	3.40.630.30	1.10.287.870
1l3lC	1l3lC01_1l3lC02	3.30.450.80	1.10.10.10
1uekA	1uekA01_1uekA02	3.30.230.10	3.30.70.890
1zowA	1zowA01_1zowA02	3.40.47.10	3.40.47.10
1tlvA	1tlvA01_1tlvA02	1.10.1790.10	1.10.1790.10
4dllA	4dllA01_4dllA02	3.40.50.720	1.10.1040.10
2xg5A	2xg5A01_2xg5A02	2.60.40.10	2.60.40.10
1va0B	1va0B01_1va0B02	3.40.1010.10	3.30.950.10
3o1lA	3o1lA01_3o1lA02	3.30.70.260	3.40.50.170
1r6xA	1r6xA01_1r6xA02	3.10.400.10	3.40.50.620
3wb0A	3wb0A01_3wb0A02	1.10.287.470	3.40.50.10150
2z1kA	2z1kA01_2z1kA02	3.20.20.80	2.60.40.1180
4fyoA	4fyoA01_4fyoA02	3.30.200.20	1.10.510.10
4gfiC	4gfiC01_4gfiC02	3.30.390.10	3.20.20.120
4do4B	4do4B01_4do4B02	3.20.20.70	2.60.40.1180
1kmoA	1kmoA01_1kmoA02	2.170.130.10	2.40.170.20
4tviA	4tviA01_4tviA02	3.30.470.10	3.20.10.10
1zxmB	1zxmB01_1zxmB02	3.30.565.10	3.30.230.10
4kamC	4kamC01_4kamC02	3.40.640.10	3.90.1150.10
3dlqR	3dlqR01_3dlqR02	2.60.40.10	2.60.40.10
2bgiA	2bgiA01_2bgiA02	2.40.30.10	3.40.50.80
1oxjA	1oxjA01_1oxjA02	1.10.150.50	1.25.40.170
2w4oA	2w4oA01_2w4oA02	3.30.200.20	1.10.510.10
1x7fA	1x7fA01_1x7fA02	3.20.20.70	2.40.100.10
1sqdA	1sqdA01_1sqdA02	3.10.180.10	3.10.180.10
3mdqA	3mdqA01_3mdqA02	3.30.420.40	3.30.420.150

4l00B	4l00B01_4l00B02	3.30.200.20	1.10.510.10
2bgcB	2bgcB01_2bgcB02	2.60.120.10	1.10.10.10
1wrjA	1wrjA01_1wrjA02	3.30.160.70	1.10.10.10
2ac3A	2ac3A01_2ac3A02	3.30.200.20	1.10.510.10
3vpbA	3vpbA01_3vpbA02	3.40.50.20	3.30.1490.20
3rf7A	3rf7A01_3rf7A02	3.40.50.1970	1.20.1090.10
1a5zA	1a5zA01_1a5zA02	3.40.50.720	3.90.110.10
1g4mB	1g4mB01_1g4mB02	2.60.40.840	2.60.40.640
2f68X	2f68X01_2f68X02	2.60.40.1280	2.60.40.740
4jxjA	4jxjA01_4jxjA02	3.40.50.150	1.10.8.100
3lv0A	3lv0A01_3lv0A02	3.30.540.10	3.40.190.80
1pn2A	1pn2A01_1pn2A02	3.10.129.10	3.10.129.10
2v8pA	2v8pA01_2v8pA02	3.30.230.10	3.30.70.890
3qt5A	3qt5A01_3qt5A02	3.30.230.10	3.30.70.890
1ct9A	1ct9A01_1ct9A02	3.60.20.10	3.40.50.620
1y9qA	1y9qA01_1y9qA02	1.10.260.40	2.60.120.10
4f6uA	4f6uA01_4f6uA02	3.30.200.20	1.10.510.10
2wi8A	2wi8A01_2wi8A02	3.40.50.1980	3.40.50.1980
2jexA	2jexA01_2jexA02	1.10.287.30	2.170.200.10
3sy8B	3sy8B01_3sy8B02	3.40.50.2300	3.20.20.450
1nijA	1nijA01_1nijA02	3.40.50.300	3.30.1220.10
3d6wA	3d6wA01_3d6wA02	2.40.50.40	2.20.25.10
3tk8A	3tk8A01_3tk8A02	1.10.166.10	2.160.10.10
3fysA	3fysA01_3fysA02	3.40.50.10170	3.30.1180.10
1mgpA	1mgpA01_1mgpA02	3.40.50.10170	3.30.1180.10
1mrzB	1mrzB01_1mrzB02	3.40.50.620	2.40.30.30
1uirB	1uirB01_1uirB02	2.30.140.10	3.40.50.150
4f0fA	4f0fA01_4f0fA02	3.30.200.20	1.10.510.10
4k89A	4k89A01_4k89A02	3.10.170.10	1.10.390.10
1f1uA	1f1uA01_1f1uA02	3.10.180.10	3.10.180.10
2y4rA	2y4rA01_2y4rA02	3.30.470.10	3.20.10.10
2z4uA	2z4uA01_2z4uA02	3.40.420.10	4.10.470.10
2pv7B	2pv7B01_2pv7B02	3.40.50.720	1.10.3660.10
2qcsB	2qcsB01_2qcsB02	2.60.120.10	2.60.120.10
2i4lB	2i4lB01_2i4lB02	3.30.930.10	3.40.50.800
3im1A	3im1A02_3im1A03	1.10.150.20	2.60.40.150
3gk7A	3gk7A01_3gk7A02	3.40.1080.10	3.30.750.70
4dnxA	4dnxA01_4dnxA02	3.10.400.10	3.40.50.620
1rl0A	1rl0A01_1rl0A02	3.40.420.10	4.10.470.10
2xotA	2xotA01_2xotA02	3.80.10.10	2.60.40.10
2hqsA	2hqsA01_2hqsA02	3.40.50.10070	2.120.10.30
3q0gA	3q0gA01_3q0gA02	3.90.226.10	1.10.12.10
3loqA	3loqA01_3loqA02	3.40.50.620	3.40.50.620
4hz4A	4hz4A01_4hz4A02	3.40.30.10	1.20.1050.10
3md9A	3md9A01_3md9A02	3.40.50.1980	3.40.50.1980
1egaA	1egaA01_1egaA02	3.40.50.300	3.30.300.20
3i83A	3i83A01_3i83A02	3.40.50.720	1.10.1040.10
2uvfA	2uvfA01_2uvfA02	2.60.40.10	2.160.20.10
4dikA	4dikA01_4dikA02	3.60.15.10	3.40.50.360
1rrmA	1rrmA01_1rrmA02	3.40.50.1970	1.20.1090.10
2r6zB	2r6zB01_2r6zB02	3.40.1630.10	3.40.50.150
2yhxA	2yhxA02_2yhxA03	3.30.420.40	3.40.367.20
3w0fA	3w0fA01_3w0fA02	3.20.190.10	1.10.8.50

4gwgA	4gwgA01_4gwgA02	3.40.50.720	1.10.1040.10
3rf3B	3rf3B01_3rf3B02	1.20.120.230	1.20.120.230
2gf2A	2gf2A01_2gf2A02	3.40.50.720	1.10.1040.10
2o0yC	2o0yC01_2o0yC02	1.10.10.10	3.30.450.40
1go3E	1go3E01_1go3E02	2.40.50.140	3.30.1490.120
3tm0A	3tm0A01_3tm0A02	3.30.200.20	3.90.1200.10
3smtA	3smtA01_3smtA02	3.90.1410.10	3.90.1420.10
2dxuA	2dxuA01_2dxuA02	3.30.930.10	2.30.30.100
4i93A	4i93A01_4i93A02	3.30.200.20	1.10.510.10
1dl5A	1dl5A01_1dl5A02	3.40.50.150	3.55.20.10
1ua7A	1ua7A01_1ua7A02	3.20.20.80	2.60.40.1180
3oc7A	3oc7A01_3oc7A02	3.90.226.10	1.10.12.10
3bqyA	3bqyA01_3bqyA02	1.10.10.60	1.10.357.10
1ik6A	1ik6A01_1ik6A02	3.40.50.970	3.40.50.920
2wsuB	2wsuB01_2wsuB02	2.60.120.200	2.60.120.200
2zp1A	2zp1A01_2zp1A02	3.40.50.620	1.10.240.10
1wkuB	1wkuB01_1wkuB02	1.10.418.10	1.10.418.10
1r3eA	1r3eA01_1r3eA02	3.30.2350.10	2.30.130.10
3t57A	3t57A01_3t57A02	2.160.10.10	1.20.1180.10
3hpyC	3hpyC01_3hpyC02	3.10.180.10	3.10.180.10
3louA	3louA01_3louA02	3.30.70.260	3.40.50.170
4b4dA	4b4dA01_4b4dA02	2.40.30.10	3.40.50.80
3tdhC	3tdhC01_3tdhC02	3.10.580.10	3.10.580.10
3bfpA	3bfpA01_3bfpA02	3.40.50.20	2.160.10.10
2ibdA	2ibdA01_2ibdA02	1.10.10.60	1.10.357.10
1j1bA	1j1bA01_1j1bA02	3.30.200.20	1.10.510.10
1sg6A	1sg6A01_1sg6A02	3.40.50.1970	1.20.1090.10
2r5wB	2r5wB01_2r5wB02	3.40.50.620	3.90.79.10
2uytA	2uytA01_2uytA02	3.30.420.40	3.30.420.40
2bjiB	2bjiB01_2bjiB02	3.30.540.10	3.40.190.80
2gtrA	2gtrA01_2gtrA02	3.90.226.10	1.10.12.10
2bo9B	2bo9B01_2bo9B02	3.10.450.10	3.10.450.10
2r79A	2r79A01_2r79A02	3.40.50.1980	3.40.50.1980
3ztvA	3ztvA01_3ztvA02	3.60.21.10	3.90.780.10
3pmeA	3pmeA01_3pmeA02	2.60.120.200	2.80.10.50
1topA	1topA01_1topA02	1.10.238.10	1.10.238.10
3al9A	3al9A01_3al9A02	2.130.10.10	3.30.1680.10
2ih2A	2ih2A01_2ih2A02	3.40.50.150	3.90.220.10
4b60A	4b60A01_4b60A02	2.60.40.1280	2.60.40.1290
2pocB	2pocB01_2pocB02	3.40.50.10490	3.40.50.10490
3ce9A	3ce9A01_3ce9A02	3.40.50.1970	1.20.1090.10
1hp1A	1hp1A01_1hp1A02	3.60.21.10	3.90.780.10
1e40A	1e40A01_1e40A02	3.20.20.80	2.60.40.1180
4f4hB	4f4hB01_4f4hB02	3.60.110.10	3.40.50.620
3mczB	3mczB01_3mczB02	1.10.10.10	3.40.50.150
2fefB	2fefB01_2fefB02	1.20.1440.70	1.10.3920.10
3fnbA	3fnbA01_3fnbA02	1.20.1440.110	3.40.50.1820
2nr4A	2nr4A01_2nr4A02	2.30.110.10	1.20.58.290
4e2oA	4e2oA01_4e2oA02	3.20.20.80	2.60.40.1180
2f4nB	2f4nB01_2f4nB02	3.40.50.10790	2.40.30.90
1wy2A	1wy2A01_1wy2A02	3.40.350.10	3.90.230.10
1udxA	1udxA01_1udxA02	2.70.210.12	3.40.50.300
4eozB	4eozB01_4eozB02	1.20.1310.10	1.20.1310.10

3fkqA	3fkqA01_3fkqA02	3.40.50.10850	3.40.50.300
1ka1A	1ka1A01_1ka1A02	3.30.540.10	3.40.190.80
1wydB	1wydB01_1wydB02	2.40.50.140	3.30.930.10
1ht6A	1ht6A01_1ht6A02	3.20.20.80	2.60.40.1180
4p10A	4p10A01_4p10A02	3.30.70.340	3.40.630.10
1mgtA	1mgtA01_1mgtA02	3.30.160.70	1.10.10.10
4jz6A	4jz6A01_4jz6A02	3.40.605.10	3.40.309.10
2q8pA	2q8pA01_2q8pA02	3.40.50.1980	3.40.50.1980
3jvaB	3jvaB01_3jvaB02	3.30.390.10	3.20.20.120
2elcA	2elcA01_2elcA02	1.20.970.10	3.40.1030.10
2r7aB	2r7aB01_2r7aB02	3.40.50.1980	3.40.50.1980
2nnuA	2nnuA01_2nnuA02	1.10.287.30	2.170.200.10
1yp2C	1yp2C01_1yp2C02	3.90.550.10	2.160.10.10
2rc5A	2rc5A01_2rc5A02	2.40.30.10	3.40.50.80
4plzA	4plzA01_4plzA02	3.40.50.720	3.90.110.10
1h1oA	1h1oA01_1h1oA02	1.10.760.10	1.10.760.10
4mboA	4mboA01_4mboA02	2.60.40.1280	2.60.40.1290
3ghyA	3ghyA01_3ghyA02	3.40.50.720	1.10.1040.10
3mwdB	3mwdB01_3mwdB02	3.40.50.720	3.40.50.261
4um9A	4um9A01_4um9A02	2.130.10.130	2.60.40.1460
2ichB	2ichB01_2ichB02	2.40.370.10	2.40.370.10
1a76A	1a76A01_1a76A02	3.40.50.1010	1.10.150.20
3p9cA	3p9cA01_3p9cA02	1.10.10.10	3.40.50.150
1s2mA	1s2mA01_1s2mA02	3.40.50.300	3.40.50.300
3ci0J	3ci0J01_3ci0J02	3.10.610.10	2.10.70.20
3ctaA	3ctaA01_3ctaA02	1.10.10.10	2.40.30.30
1g8pA	1g8pA01_1g8pA02	3.40.50.300	1.10.8.80
1jaeA	1jaeA01_1jaeA02	3.20.20.80	2.60.40.1180
1svzA	1svzA01_1svzA02	2.60.40.10	2.60.40.10
3cebA	3cebA01_3cebA02	3.30.470.10	3.20.10.10
1txdA	1txdA01_1txdA02	1.20.900.10	2.30.29.30
3ldaA	3ldaA01_3ldaA02	1.10.150.20	3.40.50.300
3nepX	3nepX01_3nepX02	3.40.50.720	3.90.110.10
3a7rA	3a7rA01_3a7rA02	3.30.930.10	3.30.390.50
3s3lA	3s3lA01_3s3lA02	3.40.47.10	3.40.47.10
2fomB	2fomB01_2fomB02	2.40.10.120	2.40.10.10
2gruA	2gruA01_2gruA02	3.40.50.1970	1.20.1090.10
3eqnA	3eqnA01_3eqnA02	2.160.20.10	2.160.20.10
2icyB	2icyB01_2icyB02	3.90.550.10	2.160.10.10
1nkrA	1nkrA01_1nkrA02	2.60.40.10	2.60.40.10
2qt5A	2qt5A01_2qt5A02	2.30.42.10	2.30.42.10
4kq9A	4kq9A01_4kq9A02	3.40.50.2300	3.40.50.2300
1rzhL	1rzhL01_1rzhL02	1.20.85.10	1.20.85.10
3hhsA	3hhsA02_3hhsA03	1.10.1280.10	2.60.40.1520
2ph7A	2ph7A01_2ph7A02	1.10.3400.10	3.40.50.10670
3bq9A	3bq9A01_3bq9A02	3.30.1850.10	3.40.50.450
1kz7A	1kz7A01_1kz7A02	1.20.900.10	2.30.29.30
4ikpA	4ikpA01_4ikpA02	3.40.50.150	2.70.160.11
1nv8A	1nv8A01_1nv8A02	1.10.8.10	3.40.50.150
4cbbA	4cbbA01_4cbbA02	3.40.605.10	3.40.309.10
1ccwB	1ccwB01_1ccwB02	3.20.20.240	3.90.970.10
4ay0B	4ay0B01_4ay0B02	2.60.40.10	2.60.40.10
3wfiA	3wfiA01_3wfiA02	3.40.50.720	1.10.1040.10

3c2bA	3c2bA01_3c2bA02	1.10.10.60	1.10.357.10
1ospO	1ospO01_1ospO02	2.40.128.160	3.90.930.1
4horA	4horA01_4horA02	1.25.40.10	1.25.40.10
4o5hD	4o5hD01_4o5hD02	3.40.605.10	3.40.309.10
1su3B	1su3B01_1su3B02	3.40.390.10	2.110.10.10
2hs5A	2hs5A01_2hs5A02	1.10.10.10	1.20.120.530
1cjxA	1cjxA01_1cjxA02	3.10.180.10	3.10.180.10
3pwfA	3pwfA01_3pwfA02	1.20.1260.10	2.20.28.10
1et9A	1et9A01_1et9A02	3.10.20.120	2.40.50.110
3sk3B	3sk3B01_3sk3B02	3.30.420.40	3.30.420.40
1xx6A	1xx6A01_1xx6A02	3.40.50.300	3.30.60.20
4fcwA	4fcwA01_4fcwA02	3.40.50.300	1.10.8.60
1tkiA	1tkiA01_1tkiA02	3.30.200.20	1.10.510.10
2ouxA	2ouxA01_2ouxA02	1.25.60.10	3.10.580.10
3bz6A	3bz6A01_3bz6A02	1.10.10.10	1.10.10.10
3hbaA	3hbaA01_3hbaA02	3.40.50.10490	3.40.50.10490
2i6dA	2i6dA01_2i6dA02	3.30.1330.30	3.40.1280.10
1e0cA	1e0cA01_1e0cA02	3.40.250.10	3.40.250.10
1hf2B	1hf2B01_1hf2B02	3.30.750.50	2.160.20.70
3u6pA	3u6pA01_3u6pA02	3.20.190.10	1.10.8.50
2cxcA	2cxcA01_2cxcA02	3.30.300.20	3.30.300.20
3nd1B	3nd1B01_3nd1B02	3.40.1010.10	3.30.950.10
2ab5A	2ab5A01_2ab5A02	3.10.28.10	3.10.28.10
1tqyA	1tqyA01_1tqyA02	3.40.47.10	3.40.47.10
4o7iA	4o7iA01_4o7iA02	3.30.540.10	3.40.190.80
1js8A	1js8A01_1js8A02	1.10.1280.10	2.60.310.10
4lc3A	4lc3A01_4lc3A02	3.40.640.10	3.90.1150.10
3cokA	3cokA01_3cokA02	3.30.200.20	1.10.510.10
3pvkA	3pvkA01_3pvkA02	2.40.70.10	2.40.70.10
4hktA	4hktA01_4hktA02	3.40.50.720	3.30.360.10
2wv0A	2wv0A01_2wv0A02	1.10.10.10	3.40.1410.10
1ku1A	1ku1A01_1ku1A02	1.10.220.20	1.10.1000.11
1jakA	1jakA01_1jakA02	3.20.20.80	3.30.379.10
1gytL	1gytL01_1gytL02	3.40.220.10	3.40.630.10
1t6eX	1t6eX01_1t6eX02	2.40.70.10	2.40.70.10
2id6A	2id6A01_2id6A02	1.10.10.60	1.10.357.10
2h88B	2h88B01_2h88B02	3.10.20.30	1.10.1060.10
2oemB	2oemB01_2oemB02	3.30.70.150	3.20.20.110
3cx3A	3cx3A01_3cx3A02	3.40.50.1980	3.40.50.1980
3rnqB	3rnqB01_3rnqB02	2.60.40.10	2.60.40.10
1knyA	1knyA01_1knyA02	3.30.460.10	1.20.120.330
2ycdA	2ycdA01_2ycdA02	3.40.30.10	1.20.1050.10
2x0kA	2x0kA01_2x0kA02	3.40.50.620	2.40.30.30
1lj8A	1lj8A01_1lj8A02	3.40.50.720	1.10.1040.10
2blfA	2blfA01_2blfA02	3.90.420.10	2.60.40.650
4q31C	4q31C01_4q31C02	3.40.640.10	3.90.1150.10
4cj0A	4cj0A01_4cj0A02	2.60.40.10	1.50.10.10
1njgA	1njgA01_1njgA02	3.40.50.300	1.10.8.60
2nteA	2nteA01_2nteA02	3.40.50.10190	3.40.50.10190
2jiiB	2jiiB01_2jiiB02	3.30.200.20	1.10.510.10
1xjvA	1xjvA01_1xjvA02	2.40.50.140	2.40.50.140
3tiwB	3tiwB01_3tiwB02	2.40.40.20	3.10.330.10
2r5tA	2r5tA01_2r5tA02	3.30.200.20	1.10.510.10

1evyA	1evyA01_1evyA02	3.40.50.720	1.10.1040.10
3dnhA	3dnhA01_3dnhA02	2.30.110.10	3.20.180.10
2eiyB	2eiyB01_2eiyB02	3.30.470.10	3.20.10.10
4iqfC	4iqfC01_4iqfC02	3.40.50.170	3.10.25.10
1yiqA	1yiqA01_1yiqA02	2.140.10.10	1.10.760.10
3cdlB	3cdlB01_3cdlB02	1.10.10.60	1.10.357.10
2raeA	2raeA01_2raeA02	1.10.10.60	1.10.357.10
1wkrA	1wkrA01_1wkrA02	2.40.70.10	2.40.70.10
3kjdB	3kjdB01_3kjdB02	1.20.142.10	3.90.228.10
3n4iB	3n4iB01_3n4iB02	3.30.1450.10	3.30.1450.10
4ke4A	4ke4A01_4ke4A02	3.30.559.10	3.30.559.10
1qhuA	1qhuA01_1qhuA02	2.110.10.10	2.110.10.10
2o07B	2o07B01_2o07B02	2.30.140.10	3.40.50.150
4js8A	4js8A01_4js8A02	3.30.200.20	1.10.510.10
2ohhA	2ohhA01_2ohhA02	3.60.15.10	3.40.50.360
1jqgA	1jqgA01_1jqgA02	3.30.70.340	3.40.630.10
3p3cA	3p3cA01_3p3cA02	3.30.230.20	3.30.1700.10
4h7pA	4h7pA01_4h7pA02	3.40.50.720	3.90.110.10
1ik9B	1ik9B01_1ik9B02	2.170.210.10	1.20.5.370
4cu4A	4cu4A01_4cu4A02	2.170.130.10	2.40.170.20
3v4gA	3v4gA01_3v4gA02	1.10.10.10	3.30.1360.40
2f1fA	2f1fA01_2f1fA02	3.30.70.260	3.30.70.1150
3mvgA	3mvgA01_3mvgA02	3.40.420.10	4.10.470.10
1rzmA	1rzmA01_1rzmA02	3.30.70.1140	3.20.20.70
4d0nB	4d0nB01_4d0nB02	1.20.900.10	2.30.29.30
3ayxB	3ayxB01_3ayxB02	3.40.50.700	4.10.480.10
1i2kA	1i2kA01_1i2kA02	3.30.470.10	3.20.10.10
4aaaA	4aaaA01_4aaaA02	3.30.200.20	1.10.510.10
2hxvA	2hxvA01_2hxvA02	3.40.140.10	3.40.430.10
2qolA	2qolA01_2qolA02	3.30.200.20	1.10.510.10
1mxgA	1mxgA01_1mxgA02	3.20.20.80	2.60.40.1180
2z3zA	2z3zA01_2z3zA02	2.140.10.30	3.40.50.1820
4ja7A	4ja7A01_4ja7A02	1.25.40.10	3.60.21.10
4kh7B	4kh7B01_4kh7B02	3.40.30.10	1.20.1050.10
4egwA	4egwA01_4egwA02	3.30.460.20	1.20.58.340
2wzbA	2wzbA01_2wzbA02	3.40.50.1260	3.40.50.1260
4ks7A	4ks7A01_4ks7A02	3.30.200.20	1.10.510.10
3clhA	3clhA01_3clhA02	3.40.50.1970	1.20.1090.10
4m1uA	4m1uA01_4m1uA02	3.40.420.10	4.10.470.10
4gtnA	4gtnA01_4gtnA02	1.20.970.10	3.40.1030.10
1qntA	1qntA01_1qntA02	3.30.160.70	1.10.10.10
3akhA	3akhA01_3akhA02	2.115.10.20	2.80.10.50
1mdxA	1mdxA01_1mdxA02	3.40.640.10	3.90.1150.10
2icwG	2icwG01_2icwG02	1.20.120.390	1.10.10.530
4eysA	4eysA01_4eysA02	3.40.50.10740	3.50.30.60
1rhyA	1rhyA01_1rhyA02	3.30.230.40	3.30.230.40
3sjnA	3sjnA01_3sjnA02	3.30.390.10	3.20.20.120
3fx3B	3fx3B01_3fx3B02	2.60.120.10	1.10.10.10
1z85B	1z85B01_1z85B02	2.40.240.20	3.40.1280.10
3a8gB	3a8gB01_3a8gB02	1.10.472.20	2.30.30.50
1o2dA	1o2dA01_1o2dA02	3.40.50.1970	1.20.1090.10
3ou5A	3ou5A01_3ou5A02	3.40.640.10	3.90.1150.10
3go9A	3go9A01_3go9A02	3.30.830.10	3.30.830.10

3vk9C	3vk9C01_3vk9C02	3.40.30.10	1.20.1050.10
1oj7A	1oj7A01_1oj7A02	3.40.50.1970	1.20.1090.10
1k3xA	1k3xA01_1k3xA02	3.20.190.10	1.10.8.50
3ckyA	3ckyA01_3ckyA02	3.40.50.720	1.10.1040.10
1ys7B	1ys7B01_1ys7B02	3.40.50.2300	1.10.10.10
2yv7A	2yv7A01_2yv7A02	3.40.30.10	1.20.1050.10
2petA	2petA01_2petA02	2.60.40.10	2.60.40.10
3frhA	3frhA01_3frhA02	1.10.8.10	3.40.50.150
4pgaA	4pgaA01_4pgaA02	3.40.50.1170	3.40.50.40
3d2uE	3d2uE01_3d2uE02	3.30.500.10	2.60.40.10
3u3zA	3u3zA01_3u3zA02	3.40.50.10190	3.40.50.10190
1w85B	1w85B01_1w85B02	3.40.50.970	3.40.50.920
1qh4A	1qh4A01_1qh4A02	1.10.135.10	3.30.590.10
3a99A	3a99A01_3a99A02	3.30.200.20	1.10.510.10
1n3lA	1n3lA01_1n3lA02	3.40.50.620	1.10.240.10
3cetB	3cetB01_3cetB02	3.30.420.40	3.30.420.190
4i9xD	4i9xD01_4i9xD02	2.10.50.10	2.10.50.10
3q8kA	3q8kA01_3q8kA02	3.40.50.1010	1.10.150.20
1x38A	1x38A01_1x38A02	3.20.20.300	3.40.50.1700
2xijA	2xijA01_2xijA02	3.20.20.240	3.40.50.280
4md5A	4md5A01_4md5A02	3.10.320.10	2.60.40.10
3isqA	3isqA01_3isqA02	3.10.180.10	3.10.180.10
3cnlA	3cnlA01_3cnlA02	3.40.50.300	1.10.1580.10
2gfqA	2gfqA01_2gfqA02	3.40.630.50	3.40.50.10700
4ea9A	4ea9A01_4ea9A02	3.40.50.20	2.160.10.10
3jqqC	3jqqC01_3jqqC02	2.40.30.10	3.40.50.80
3fj1A	3fj1A01_3fj1A02	3.40.50.10490	3.40.50.10490
4c08A	4c08A01_4c08A02	3.40.50.150	2.70.160.11
3umvA	3umvA01_3umvA02	3.40.50.620	1.25.40.80
3ur6B	3ur6B01_3ur6B02	2.40.10.10	2.40.10.10
1u5qA	1u5qA01_1u5qA02	3.30.200.20	1.10.510.10
3twlA	3twlA01_3twlA02	3.20.190.10	1.10.8.50
1d2nA	1d2nA01_1d2nA02	3.40.50.300	1.10.8.60
2cvzA	2cvzA01_2cvzA02	3.40.50.720	1.10.1040.10
2z6rB	2z6rB01_2z6rB02	3.40.1010.10	3.30.950.10
4ca1B	4ca1B01_4ca1B02	3.30.40.10	2.60.210.10
3ifrA	3ifrA01_3ifrA02	3.30.420.40	3.30.420.40
1omrA	1omrA01_1omrA02	1.10.238.10	1.10.238.10
3tacB	3tacB02_3tacB03	1.10.150.50	1.10.150.50
2hxtA	2hxtA01_2hxtA02	3.30.390.10	3.20.20.120
3ktzA	3ktzA01_3ktzA02	3.40.420.10	4.10.470.10
3wjpA	3wjpA01_3wjpA02	3.30.1330.10	3.90.650.10
1j8mF	1j8mF01_1j8mF02	1.20.120.140	3.40.50.300
1vqzA	1vqzA01_1vqzA02	3.30.930.10	3.30.390.50
1oktA	1oktA01_1oktA02	3.40.30.10	1.20.1050.10
3uc3A	3uc3A01_3uc3A02	3.30.200.20	1.10.510.10
2hftA	2hftA01_2hftA02	2.60.40.10	2.60.40.10
2obnD	2obnD01_2obnD02	3.40.50.720	3.40.50.300
2arzA	2arzA01_2arzA02	2.30.110.10	3.20.180.10
2rcyA	2rcyA01_2rcyA02	3.40.50.720	1.10.3730.10
1vcoA	1vcoA01_1vcoA02	3.40.50.300	3.40.50.880
3k05B	3k05B01_3k05B02	3.40.50.10190	3.40.50.10190
2wa7A	2wa7A01_2wa7A02	1.10.418.10	1.10.418.10

1qs1A	1qs1A01_1qs1A02	3.90.176.10	3.90.176.10
1vhyA	1vhyA01_1vhyA02	2.40.240.20	3.40.1280.10
2bb3B	2bb3B01_2bb3B02	3.40.1010.10	3.30.950.10
3d1lB	3d1lB01_3d1lB02	3.40.50.720	1.10.1040.20
3iv7A	3iv7A01_3iv7A02	3.40.50.1970	1.20.1090.10
3o36A	3o36A01_3o36A02	3.30.40.10	1.20.920.10
3mqdA	3mqdA01_3mqdA02	3.40.47.10	3.40.47.10
2zyqA	2zyqA01_2zyqA02	3.10.180.10	3.10.180.10
2i9aB	2i9aB01_2i9aB02	2.10.25.10	2.40.20.10
1kyfA	1kyfA01_1kyfA02	2.60.40.1230	3.30.310.10
1nzyA	1nzyA01_1nzyA02	3.90.226.10	1.10.12.10
3u0oB	3u0oB01_3u0oB02	3.30.1330.10	3.90.650.10
2vu9A	2vu9A01_2vu9A02	2.60.120.200	2.80.10.50
3cbuB	3cbuB01_3cbuB02	3.40.30.10	1.20.1050.10
1sesA	1sesA01_1sesA02	1.10.287.40	3.30.930.10
3qbeA	3qbeA01_3qbeA02	3.40.50.1970	1.20.1090.10
3u7vA	3u7vA01_3u7vA02	3.20.20.80	2.60.220.20
3afgB	3afgB02_3afgB03	3.40.50.200	2.60.120.380
1jg8A	1jg8A01_1jg8A02	3.40.640.10	3.90.1150.10
1a62A	1a62A01_1a62A02	1.10.720.10	2.40.50.140
2eifA	2eifA01_2eifA02	2.30.30.30	2.40.50.140
2pidB	2pidB01_2pidB02	3.40.50.620	1.10.240.10
3kk8A	3kk8A01_3kk8A02	3.30.200.20	1.10.510.10
2bfdB	2bfdB01_2bfdB02	3.40.50.970	3.40.50.920
3q0iA	3q0iA01_3q0iA02	3.40.50.170	3.10.25.10
3kh8A	3kh8A01_3kh8A02	3.10.129.10	3.10.129.10
2eayB	2eayB01_2eayB02	3.30.930.10	2.30.30.100
3w6zA	3w6zA01_3w6zA02	3.40.50.720	1.10.1040.10
3uenA	3uenA01_3uenA02	3.40.50.10190	3.40.50.10190
2ea7A	2ea7A01_2ea7A02	2.60.120.10	2.60.120.10
1g87B	1g87B01_1g87B02	1.50.10.10	2.60.40.710
3lqsA	3lqsA01_3lqsA02	3.30.470.10	3.20.10.10
1mjfA	1mjfA01_1mjfA02	2.30.140.10	3.40.50.150
1vliA	1vliA01_1vliA02	3.20.20.70	3.90.1210.10
1orvA	1orvA01_1orvA02	2.140.10.30	3.40.50.1820
1ks9A	1ks9A01_1ks9A02	3.40.50.720	1.10.1040.10
2xtsA	2xtsA01_2xtsA02	3.90.420.10	2.60.40.650
1a8rA	1a8rA01_1a8rA02	1.10.286.10	3.30.1130.10
3zgjB	3zgjB01_3zgjB02	3.10.180.10	3.10.180.10
3m3mA	3m3mA01_3m3mA02	3.40.30.10	1.20.1050.10
3c9zA	3c9zA01_3c9zA02	2.80.10.50	2.80.10.50
1n67A	1n67A01_1n67A02	2.60.40.1280	2.60.40.1290
3e04C	3e04C01_3e04C02	1.10.275.10	1.20.200.10
4e04B	4e04B01_4e04B02	3.30.450.20	3.30.450.40
1wf3A	1wf3A01_1wf3A02	3.40.50.300	3.30.300.20
3tlfD	3tlfD01_3tlfD02	3.90.226.10	1.10.12.10
4m8iA	4m8iA01_4m8iA02	3.40.50.1440	3.30.1330.20
3nt1A	3nt1A01_3nt1A02	2.10.25.10	1.10.640.10
3my9A	3my9A01_3my9A02	3.30.390.10	3.20.20.120
2qyzA	2qyzA01_2qyzA02	3.30.1490.160	2.30.30.290
4hbqB	4hbqB01_4hbqB02	2.60.40.10	2.60.40.10
1uarA	1uarA01_1uarA02	3.40.250.10	3.40.250.10
2vs7D	2vs7D01_2vs7D02	3.10.28.10	3.10.28.10

1ulyA	1ulyA01_1ulyA02	1.10.10.10	1.10.20.10
1yqgA	1yqgA01_1yqgA02	3.40.50.720	1.10.3730.10
1vyuA	1vyuA01_1vyuA02	2.30.30.40	3.40.50.300
3vx0A	3vx0A01_3vx0A02	3.20.20.80	2.60.40.1180
4gyoB	4gyoB01_4gyoB02	1.25.40.10	1.25.40.10
2oi2A	2oi2A01_2oi2A02	3.30.230.10	3.30.70.890
3pohA	3pohA01_3pohA02	2.60.40.1740	3.20.20.80
3uhmA	3uhmA01_3uhmA02	3.30.230.20	3.30.1700.10
2v6kA	2v6kA01_2v6kA02	3.40.30.10	1.20.1050.10
4knuA	4knuA01_4knuA02	2.60.40.420	2.60.40.420
1cjyA	1cjyA01_1cjyA02	2.60.40.150	3.40.1090.10
3iavA	3iavA01_3iavA02	3.90.226.10	3.90.226.10
4b3hA	4b3hA01_4b3hA02	3.90.226.10	3.40.50.720
4e72A	4e72A01_4e72A02	3.30.565.40	3.90.640.20
2g3rA	2g3rA01_2g3rA02	2.30.30.140	2.30.30.30
4i4tA	4i4tA02_4i4tA03	3.30.1330.20	1.10.287.600
3ir9A	3ir9A01_3ir9A02	1.20.5.170	3.30.1330.30
2qgqA	2qgqA01_2qgqA02	3.80.30.20	2.40.50.140
4x9yA	4x9yA01_4x9yA02	3.20.20.80	2.60.40.1180
4h3vA	4h3vA01_4h3vA02	3.40.50.720	3.30.360.10
3cwvA	3cwvA01_3cwvA02	3.30.565.10	3.30.230.10
3egoA	3egoA01_3egoA02	3.40.50.720	1.10.1040.10
4hkmB	4hkmB01_4hkmB02	1.20.970.10	3.40.1030.10
2w37A	2w37A01_2w37A02	3.40.50.1370	3.40.50.1370
2z0qA	2z0qA01_2z0qA02	1.20.900.10	2.30.29.30
3rc1A	3rc1A01_3rc1A02	3.40.50.720	3.30.360.10
3ugcA	3ugcA01_3ugcA02	3.30.200.20	1.10.510.10
3p1aA	3p1aA01_3p1aA02	3.30.200.20	1.10.510.10
2qqiA	2qqiA01_2qqiA02	2.60.120.260	2.60.120.260
3triA	3triA01_3triA02	3.40.50.720	1.10.3730.10
1bg6A	1bg6A01_1bg6A02	3.40.50.720	1.10.1040.10
3qqmA	3qqmA01_3qqmA02	3.30.470.10	3.20.10.10
4ffhA	4ffhA01_4ffhA02	2.115.10.20	2.60.120.560
3kh5A	3kh5A01_3kh5A02	3.10.580.10	3.10.580.10
2a9dA	2a9dA01_2a9dA02	3.90.420.10	2.60.40.650
3sc7X	3sc7X01_3sc7X02	2.115.10.20	2.60.120.560
4c72A	4c72A01_4c72A02	3.40.47.10	3.40.47.10
1fd9A	1fd9A01_1fd9A02	3.10.50.40	1.10.287.460
2hekA	2hekA01_2hekA02	1.10.3210.10	3.30.70.1370
1ogaE	1ogaE01_1ogaE02	2.60.40.10	2.60.40.10
3nqhA	3nqhA01_3nqhA02	2.115.10.20	2.60.120.260
3o74B	3o74B01_3o74B02	3.40.50.2300	3.40.50.2300
4m98A	4m98A01_4m98A02	3.40.50.20	2.160.10.10
4d02A	4d02A01_4d02A02	3.60.15.10	3.40.50.360
1tzbA	1tzbA01_1tzbA02	3.40.50.10490	3.40.50.10490
1wzoB	1wzoB01_1wzoB02	2.30.30.370	3.90.850.10
3vpgA	3vpgA01_3vpgA02	3.40.50.720	3.90.110.10
1rktA	1rktA01_1rktA02	1.10.10.60	1.10.357.10
3mzfA	3mzfA01_3mzfA02	3.40.710.10	2.60.410.10
2r751	2r75101_2r75102	3.40.50.1440	3.30.1330.20
3hhtB	3hhtB01_3hhtB02	1.10.472.20	2.30.30.50
2a06B	2a06B01_2a06B02	3.30.830.10	3.30.830.10
4igjA	4igjA01_4igjA02	3.40.30.10	1.20.1050.10

3vb0A	3vb0A01_3vb0A02	3.10.180.10	3.10.180.10
3f3zA	3f3zA01_3f3zA02	3.30.200.20	1.10.510.10
3moyA	3moyA01_3moyA02	3.90.226.10	1.10.12.10
1feuA	1feuA01_1feuA02	2.40.240.10	2.170.120.20
4m9cA	4m9cA01_4m9cA02	3.40.50.20	2.160.10.10
4hwtB	4hwtB01_4hwtB02	3.30.930.10	3.40.50.800
1f5nA	1f5nA01_1f5nA02	1.20.1000.10	3.40.50.300
3brcB	3brcB01_3brcB02	1.10.287.470	3.40.50.10150
2xsaA	2xsaA01_2xsaA02	3.20.20.80	1.20.58.240
3kw2A	3kw2A01_3kw2A02	2.40.240.20	3.40.1280.10
3hksA	3hksA01_3hksA02	2.30.30.30	2.40.50.140
1wkyA	1wkyA01_1wkyA02	3.20.20.80	2.60.120.260
1iuqA	1iuqA01_1iuqA02	1.10.1200.50	3.40.1130.10
1kkoA	1kkoA01_1kkoA02	3.30.390.10	3.20.20.120
3s21A	3s21A01_3s21A02	3.40.47.10	3.40.47.10
3kf3A	3kf3A01_3kf3A02	2.115.10.20	2.60.120.560
2yl6A	2yl6A01_2yl6A02	3.20.20.80	1.20.1270.90
2gauA	2gauA01_2gauA02	2.60.120.10	1.10.10.10
2f23A	2f23A01_2f23A02	1.10.287.180	3.10.50.30
4l7gA	4l7gA01_4l7gA02	2.40.70.10	2.40.70.10
3irmA	3irmA01_3irmA02	3.40.430.10	3.30.572.10
1yb4A	1yb4A01_1yb4A02	3.40.50.720	1.10.1040.10
4lrtC	4lrtC01_4lrtC02	3.20.20.70	1.10.8.60
1u94A	1u94A01_1u94A02	3.40.50.300	3.30.250.10
1xeuA	1xeuA01_1xeuA02	3.80.10.10	2.60.40.1220
4bu0A	4bu0A01_4bu0A02	3.40.50.10190	3.40.50.10190
1reqB	1reqB01_1reqB02	3.20.20.240	3.40.50.280
3zokA	3zokA01_3zokA02	3.40.50.1970	1.20.1090.10
2hyjA	2hyjA01_2hyjA02	1.10.10.60	1.10.357.10
1h4aX	1h4aX01_1h4aX02	2.60.20.10	2.60.20.10
4fwwA	4fwwA01_4fwwA02	2.130.10.10	3.30.1680.10
1v54B	1v54B01_1v54B02	1.10.287.90	2.60.40.420
1bu8A	1bu8A01_1bu8A02	3.40.50.1820	2.60.60.20
4fklA	4fklA01_4fklA02	3.30.200.20	1.10.510.10
1hnjA	1hnjA01_1hnjA02	3.40.47.10	3.40.47.10
1gnwA	1gnwA01_1gnwA02	3.40.30.10	1.20.1050.10
3a32A	3a32A01_3a32A02	3.30.930.10	3.40.50.800
3ihuA	3ihuA01_3ihuA02	1.10.10.10	1.20.120.530
3q7eA	3q7eA01_3q7eA02	3.40.50.150	2.70.160.11
3ccyA	3ccyA01_3ccyA02	1.10.10.60	1.10.357.10
3orkA	3orkA01_3orkA02	3.30.200.20	1.10.510.10
3ox4A	3ox4A01_3ox4A02	3.40.50.1970	1.20.1090.10
3evnA	3evnA01_3evnA02	3.40.50.720	3.30.360.10
2i2xH	2i2xH01_2i2xH02	1.10.1240.10	3.40.50.280
1i88A	1i88A01_1i88A02	3.40.47.10	3.40.47.10
2ggoA	2ggoA01_2ggoA02	3.90.550.10	2.160.10.10
1v47A	1v47A01_1v47A02	3.10.400.10	3.40.50.620
4h8wC	4h8wC01_4h8wC02	2.60.40.10	2.60.40.10
3ag6A	3ag6A01_3ag6A02	3.40.50.620	3.30.1300.10
1ncwL	1ncwL01_1ncwL02	2.60.40.10	2.60.40.10
1hm9A	1hm9A01_1hm9A02	3.90.550.10	2.160.10.10
1c8uA	1c8uA01_1c8uA02	3.10.129.10	3.10.129.10
1rqpA	1rqpA01_1rqpA02	3.40.50.10790	2.40.30.90

3covA	3covA01_3covA02	3.40.50.620	3.30.1300.10
3swxB	3swxB01_3swxB02	3.90.226.10	1.10.12.10
3vm7A	3vm7A01_3vm7A02	3.20.20.80	2.60.40.1180
3biqA	3biqA01_3biqA02	3.40.350.10	3.90.230.10
1dzfA	1dzfA01_1dzfA02	3.40.1340.10	3.90.940.20
2hrvA	2hrvA01_2hrvA02	2.40.10.10	2.40.10.10
3mxtA	3mxtA01_3mxtA02	3.40.50.620	3.30.1300.10
2ip1A	2ip1A01_2ip1A02	3.40.50.620	1.10.240.10
4fj6D	4fj6D01_4fj6D02	2.60.40.1290	2.120.10.10
3zpgA	3zpgA01_3zpgA02	3.40.140.10	3.40.430.10
4a6dA	4a6dA01_4a6dA02	1.10.10.10	3.40.50.150
3wgcB	3wgcB01_3wgcB02	3.40.640.10	3.90.1150.10
4c2vA	4c2vA01_4c2vA02	3.30.200.20	1.10.510.10
1w4rA	1w4rA01_1w4rA02	3.40.50.300	3.30.60.20
1v8cA	1v8cA01_1v8cA02	3.10.20.30	3.30.1370.80
4qiiB	4qiiB01_4qiiB02	3.90.226.10	1.10.12.10
3tpzA	3tpzA01_3tpzA02	3.40.50.150	1.10.8.100
3kfaA	3kfaA01_3kfaA02	3.30.200.20	1.10.510.10
3b02A	3b02A01_3b02A02	2.60.120.10	1.10.10.10
1yi9A	1yi9A01_1yi9A02	2.60.120.310	2.60.120.230
2g7zA	2g7zA01_2g7zA02	3.40.50.10170	3.30.1180.10
3bwuC	3bwuC01_3bwuC02	2.60.40.10	2.60.40.10
1mppA	1mppA01_1mppA02	2.40.70.10	2.40.70.10
1ntyA	1ntyA01_1ntyA02	1.20.900.10	2.30.29.30
4chsA	4chsA01_4chsA02	3.40.30.10	1.20.1050.10
3cx5B	3cx5B01_3cx5B02	3.30.830.10	3.30.830.10
3pduA	3pduA01_3pduA02	3.40.50.720	1.10.1040.10
2x7jA	2x7jA01_2x7jA02	3.40.50.970	3.40.50.1220
3dl2A	3dl2A01_3dl2A02	3.40.50.720	3.90.110.10
1earA	1earA01_1earA02	2.60.260.20	3.30.70.790
2wjnM	2wjnM01_2wjnM02	1.20.85.10	1.20.85.10
2zcwA	2zcwA01_2zcwA02	2.60.120.10	1.10.10.10
3eh7A	3eh7A01_3eh7A02	3.40.1080.10	3.30.750.70
4qosA	4qosA01_4qosA02	3.40.50.300	1.10.8.60
1qd1B	1qd1B01_1qd1B02	3.30.990.10	3.30.70.670
4ir8B	4ir8B01_4ir8B02	3.30.540.10	3.40.190.80
2wb9A	2wb9A01_2wb9A02	3.40.30.10	1.20.1050.10
1lf6A	1lf6A01_1lf6A02	2.70.98.10	1.50.10.10
1k82B	1k82B01_1k82B02	3.20.190.10	1.10.8.50
4ew6A	4ew6A01_4ew6A02	3.40.50.720	3.30.360.10
1d5rA	1d5rA01_1d5rA02	3.90.190.10	2.60.40.1110
3ll3A	3ll3A01_3ll3A02	3.30.420.40	3.30.420.40
2bi0A	2bi0A01_2bi0A02	3.10.129.10	3.10.129.10
2qz4A	2qz4A01_2qz4A02	3.40.50.300	1.10.8.60
1bygA	1bygA01_1bygA02	3.30.200.20	1.10.510.10
1h7sA	1h7sA01_1h7sA02	3.30.565.10	3.30.230.10
2qiaA	2qiaA01_2qiaA02	2.160.10.10	1.20.1180.10
2c81A	2c81A01_2c81A02	3.40.640.10	3.90.1150.10
2egvA	2egvA01_2egvA02	2.40.240.20	3.40.1280.10
1jgtB	1jgtB01_1jgtB02	3.60.20.10	3.40.50.620
3sdeA	3sdeA01_3sdeA02	3.30.70.330	3.30.70.330
4oc9D	4oc9D01_4oc9D02	3.40.640.10	3.90.1150.10
1u1qA	1u1qA01_1u1qA02	3.30.70.330	3.30.70.330

4f01B	4f01B01_4f01B02	2.60.34.10	1.20.1270.10
3fmsA	3fmsA01_3fmsA02	1.10.10.10	1.20.120.530
1q6hA	1q6hA01_1q6hA02	1.10.287.460	3.10.50.40
1okiB	1okiB01_1okiB02	2.60.20.10	2.60.20.10
1s6yA	1s6yA01_1s6yA02	3.40.50.720	3.90.110.10
1wpbD	1wpbD01_1wpbD02	1.10.287.680	1.10.3190.10
4oujA	4oujA01_4oujA02	2.80.10.50	2.80.10.50
1w23A	1w23A01_1w23A02	3.40.640.10	3.90.1150.10
1dbhA	1dbhA01_1dbhA02	1.20.900.10	2.30.29.30
4rfzA	4rfzA01_4rfzA02	3.30.200.20	1.10.510.10
3nk6A	3nk6A01_3nk6A02	3.30.1330.30	3.40.1280.10
3agkA	3agkA02_3agkA03	3.30.420.60	3.30.1330.30
2fq4A	2fq4A01_2fq4A02	1.10.10.60	1.10.357.10
1l0qA	1l0qA01_1l0qA02	2.130.10.10	2.60.40.10
1g94A	1g94A01_1g94A02	3.20.20.80	2.60.40.1180
3i4tA	3i4tA01_3i4tA02	3.40.1010.10	3.30.950.10
2zvbA	2zvbA01_2zvbA02	3.40.1010.10	3.30.950.10
3uesB	3uesB01_3uesB02	3.20.20.80	2.60.120.260
1efdN	1efdN01_1efdN02	3.40.50.1980	3.40.50.1980
3hi0A	3hi0A01_3hi0A02	3.30.420.40	3.30.420.150
1fo8A	1fo8A01_1fo8A02	3.90.550.10	3.10.180.20
3zi1A	3zi1A01_3zi1A02	3.10.180.10	3.10.180.10
1qwrA	1qwrA01_1qwrA02	2.60.120.10	2.60.120.10
3q0wA	3q0wA01_3q0wA02	1.10.10.60	1.10.357.10
3p4eA	3p4eA01_3p4eA02	3.30.1330.10	3.90.650.10
1ux6A	1ux6A01_1ux6A02	4.10.1080.10	2.60.120.200
1h72C	1h72C01_1h72C02	3.30.230.10	3.30.70.890
2pfxA	2pfxA01_2pfxA02	1.20.5.810	1.20.1290.10
3c0wA	3c0wA01_3c0wA02	3.10.28.10	3.10.28.10
3uqcC	3uqcC01_3uqcC02	3.30.200.20	1.10.510.10
3zclA	3zclA01_3zclA02	3.30.200.20	1.10.510.10
3uo3A	3uo3A01_3uo3A02	1.10.287.110	1.20.1280.20
1t7vA	1t7vA01_1t7vA02	3.30.500.10	2.60.40.10
2cw5C	2cw5C01_2cw5C02	3.40.50.10790	2.40.30.90
3n7lA	3n7lA01_3n7lA02	2.60.120.200	2.80.10.50
4hpnA	4hpnA01_4hpnA02	3.30.390.10	3.20.20.120
3obiA	3obiA01_3obiA02	3.30.70.260	3.40.50.170
1toaA	1toaA01_1toaA02	3.40.50.1980	3.40.50.1980
1wurA	1wurA01_1wurA02	1.10.286.10	3.30.1130.10
1qgiA	1qgiA01_1qgiA02	3.30.386.10	1.20.141.10
1e5dA	1e5dA01_1e5dA02	3.40.50.360	3.60.15.10
2j3xA	2j3xA01_2j3xA02	3.90.176.10	3.90.176.10
4lcyF	4lcyF01_4lcyF02	3.30.500.10	2.60.40.10
1uctA	1uctA01_1uctA02	2.60.40.10	2.60.40.10
1v2aB	1v2aB01_1v2aB02	3.40.30.10	1.20.1050.10
1h6tA	1h6tA01_1h6tA02	3.80.10.10	2.60.40.1220
2hkxA	2hkxA01_2hkxA02	2.60.120.10	1.10.10.10
4hzrA	4hzrA01_4hzrA02	3.30.200.20	1.10.510.10
1y4wA	1y4wA01_1y4wA02	2.115.10.20	2.60.120.560
4n6aA	4n6aA01_4n6aA02	1.10.3130.10	2.160.10.10
3ddjA	3ddjA01_3ddjA02	3.10.580.10	3.10.580.10
1rz4A	1rz4A01_1rz4A02	1.25.40.250	1.10.10.10
1ut9A	1ut9A01_1ut9A02	2.60.40.10	1.50.10.10

3bb0A	3bb0A01_3bb0A02	1.20.144.10	1.10.606.10
3sovA	3sovA01_3sovA02	2.120.10.30	2.10.25.10
3ze9A	3ze9A01_3ze9A02	3.40.50.700	4.10.480.10
3cmqA	3cmqA01_3cmqA02	3.30.930.10	3.30.70.380
4loxA	4loxA01_4loxA02	3.10.28.10	3.10.28.10
4l5rC	4l5rC01_4l5rC02	2.40.50.140	2.40.50.140
2cb1A	2cb1A01_2cb1A02	3.40.640.10	3.90.1150.10
4ojmX	4ojmX01_4ojmX02	3.40.50.620	1.10.240.10
2b5iC	2b5iC01_2b5iC02	2.60.40.10	2.60.40.10
3ljuX	3ljuX02_3ljuX03	2.30.29.30	2.30.29.30
1t33A	1t33A01_1t33A02	1.10.10.60	1.10.357.10
2gq1A	2gq1A01_2gq1A02	3.30.540.10	3.40.190.80
4u7jA	4u7jA01_4u7jA02	3.40.50.620	3.90.1260.10
3i6eA	3i6eA01_3i6eA02	3.30.390.10	3.20.20.120
3lgiB	3lgiB01_3lgiB02	2.40.10.10	2.40.10.10
2ibgA	2ibgA01_2ibgA02	2.60.40.10	2.60.40.10
3k1dA	3k1dA02_3k1dA03	3.20.20.80	2.60.40.1180
2v2gA	2v2gA01_2v2gA02	3.40.30.10	3.30.1020.10
2p84A	2p84A01_2p84A02	2.60.430.10	2.30.30.290
1kskA	1kskA01_1kskA02	3.10.290.10	3.30.2350.10
3i0zA	3i0zA01_3i0zA02	3.40.50.10490	3.40.50.10490
3gdiA	3gdiA01_3gdiA02	3.30.450.20	3.30.450.20
3eqcA	3eqcA01_3eqcA02	3.30.200.20	1.10.510.10
4hfiA	4hfiA01_4hfiA02	2.70.170.10	1.20.58.390
3socB	3socB01_3socB02	3.30.200.20	1.10.510.10
2gdqA	2gdqA01_2gdqA02	3.30.390.10	3.20.20.120
1o57A	1o57A01_1o57A02	1.10.10.10	3.40.50.2020
1k8kD	1k8kD01_1k8kD02	3.30.1460.20	3.30.1460.20
1m15A	1m15A01_1m15A02	1.10.135.10	3.30.590.10
3vlaA	3vlaA01_3vlaA02	2.40.70.10	2.40.70.10
3o4hA	3o4hA01_3o4hA02	2.130.10.150	3.40.50.1820
2r7gC	2r7gC01_2r7gC02	1.10.472.10	1.10.472.10
3c2qB	3c2qB01_3c2qB02	2.40.420.10	3.40.50.10690
1qfjA	1qfjA01_1qfjA02	2.40.30.10	3.40.50.80
4j3cB	4j3cB01_4j3cB02	2.40.240.20	3.40.1280.10
3bjdC	3bjdC01_3bjdC02	1.10.1240.20	1.20.910.10
2ra9A	2ra9A01_2ra9A02	3.10.540.10	2.30.270.10
1hyhA	1hyhA01_1hyhA02	3.40.50.720	3.90.110.10
3obbA	3obbA01_3obbA02	3.40.50.720	1.10.1040.10
1lbvA	1lbvA01_1lbvA02	3.30.540.10	3.40.190.80
3uimA	3uimA01_3uimA02	3.30.200.20	1.10.510.10
1pswA	1pswA01_1pswA02	3.40.50.2000	3.40.50.2000
1q7hA	1q7hA01_1q7hA02	3.10.450.120	2.30.130.10
3ajdA	3ajdA01_3ajdA02	3.30.70.1170	3.40.50.150
3ijlA	3ijlA01_3ijlA02	3.30.390.10	3.20.20.120
1jy1A	1jy1A01_1jy1A02	3.30.870.10	3.30.870.20
1vpdA	1vpdA01_1vpdA02	3.40.50.720	1.10.1040.10
3seiA	3seiA01_3seiA02	1.10.150.50	1.10.150.50
3or1C	3or1C01_3or1C02	3.30.1420.10	1.10.10.370
4prxA	4prxA01_4prxA02	3.30.565.10	3.30.230.10
1yuzB	1yuzB01_1yuzB02	1.20.1260.10	2.20.28.10
1vctA	1vctA01_1vctA02	1.20.58.220	3.30.70.1450
4finA	4finA01_4finA02	3.40.50.300	3.40.50.300

4fasA	4fasA01_4fasA02	1.10.780.10	1.20.850.10
4nnoA	4nnoA01_4nnoA02	3.40.50.1980	3.40.50.1980
2g30A	2g30A01_2g30A02	2.60.40.1150	3.30.310.10
1xp8A	1xp8A01_1xp8A02	3.40.50.300	3.30.250.10
4mocA	4mocA01_4mocA02	3.10.129.10	3.10.129.10
3eoqB	3eoqB01_3eoqB02	3.30.830.10	3.30.830.10
1w55A	1w55A01_1w55A02	3.90.550.10	3.30.1330.50
4mpcA	4mpcA01_4mpcA02	1.20.140.20	3.30.565.10
1xr4A	1xr4A01_1xr4A02	3.40.1080.10	3.40.1080.10
3n06B	3n06B01_3n06B02	2.60.40.10	2.60.40.10
3i07B	3i07B01_3i07B02	2.20.25.10	3.30.300.20
1dciA	1dciA01_1dciA02	3.90.226.10	1.10.12.10
3ohrA	3ohrA01_3ohrA02	3.30.420.40	3.30.420.40
3c8zB	3c8zB01_3c8zB02	3.40.50.620	1.20.120.640
2jj7B	2jj7B01_2jj7B02	1.10.10.60	1.10.357.10
3k5rA	3k5rA01_3k5rA02	2.60.40.60	2.60.40.60
3ereD	3ereD01_3ereD02	1.10.10.10	3.30.1360.40
1ujnA	1ujnA01_1ujnA02	3.40.50.1970	1.20.1090.10
2hxiB	2hxiB01_2hxiB02	1.10.10.60	1.10.357.10
2ej9A	2ej9A01_2ej9A02	3.30.930.10	2.30.30.100
3p7nA	3p7nA01_3p7nA02	3.30.450.20	1.10.10.10
1wmdA	1wmdA01_1wmdA02	3.40.50.200	2.60.120.380
1jmcA	1jmcA01_1jmcA02	2.40.50.140	2.40.50.140
3flgA	3flgA01_3flgA02	2.30.30.140	1.10.720.50
2fl4A	2fl4A01_2fl4A02	1.10.287.900	3.40.630.30
2ae8B	2ae8B01_2ae8B02	3.30.230.40	3.30.230.40
4hzfB	4hzfB01_4hzfB02	2.60.120.10	1.10.10.10
2f5fB	2f5fB01_2f5fB02	1.10.10.10	1.10.60.10
1n62A	1n62A01_1n62A02	3.10.20.30	1.10.150.120
1vi0A	1vi0A01_1vi0A02	1.10.10.60	1.10.357.10
4jcwA	4jcwA01_4jcwA02	2.40.40.10	2.60.40.760
2e1vA	2e1vA01_2e1vA02	3.30.559.10	3.30.559.10
1rl2A	1rl2A01_1rl2A02	2.40.50.140	2.30.30.30
1jbwA	1jbwA01_1jbwA02	3.40.1190.10	3.90.190.20
4omcA	4omcA01_4omcA02	3.40.50.200	2.60.120.260
1ekeA	1ekeA01_1ekeA02	3.30.420.10	1.10.10.460
4gt4A	4gt4A01_4gt4A02	3.30.200.20	1.10.510.10
1pujA	1pujA01_1pujA02	3.40.50.300	1.10.1580.10
1fyhB	1fyhB01_1fyhB02	2.60.40.10	2.60.40.10
1hdhA	1hdhA01_1hdhA02	3.40.720.10	3.30.1120.10
3ojoB	3ojoB01_3ojoB02	3.40.50.720	3.40.50.720
3bkhA	3bkhA01_3bkhA02	1.10.101.10	1.10.530.10
4n4eE	4n4eE01_4n4eE02	3.10.170.10	1.10.390.10
2gvhB	2gvhB01_2gvhB02	3.10.129.10	3.10.129.10
4i2oA	4i2oA01_4i2oA02	2.60.120.10	1.10.10.10
3gwzA	3gwzA01_3gwzA02	1.10.10.10	3.40.50.150
2hc9A	2hc9A01_2hc9A02	3.40.50.10590	3.40.630.10
3uh0A	3uh0A01_3uh0A02	3.30.930.10	3.40.50.800
2vvnA	2vvnA01_2vvnA02	3.30.379.10	3.20.20.80
2i53A	2i53A01_2i53A02	1.10.472.10	1.10.472.10
3cswC	3cswC01_3cswC02	3.30.470.10	3.20.10.10
4m0jA	4m0jA01_4m0jA02	3.30.470.10	3.20.10.10
3afcA	3afcA01_3afcA02	2.130.10.10	3.30.1680.10

3dlsA	3dlsA01_3dlsA02	3.30.200.20	1.10.510.10
2ahuA	2ahuA01_2ahuA02	3.40.1080.10	3.40.1080.10
4kdxA	4kdxA01_4kdxA02	3.40.30.10	1.20.1050.10
2rfvA	2rfvA01_2rfvA02	3.40.640.10	3.90.1150.10
2xirA	2xirA01_2xirA02	3.30.200.20	1.10.510.10
1r4xA	1r4xA01_1r4xA02	2.60.40.1480	3.30.310.10
4ajyV	4ajyV01_4ajyV02	2.60.40.780	1.10.750.10
2znxA	2znxA01_2znxA02	2.60.40.10	2.60.40.10
1px5B	1px5B01_1px5B02	3.30.460.10	1.10.1410.20
2hfsA	2hfsA01_2hfsA02	3.30.230.10	3.30.70.890
4bf2A	4bf2A01_4bf2A02	3.30.200.20	1.10.510.10
3knzA	3knzA01_3knzA02	3.40.50.10490	3.40.50.10490
2gwrA	2gwrA01_2gwrA02	3.40.50.2300	1.10.10.10
3q48A	3q48A01_3q48A02	2.60.40.10	2.60.40.10
3k1wB	3k1wB01_3k1wB02	2.40.70.10	2.40.70.10
4avaA	4avaA01_4avaA02	2.60.120.10	3.40.630.30
4qmaA	4qmaA01_4qmaA02	1.20.5.440	2.60.120.10
1a04A	1a04A01_1a04A02	3.40.50.2300	1.10.10.10
4m2aA	4m2aA01_4m2aA02	3.90.550.10	2.160.10.10
1oi7A	1oi7A01_1oi7A02	3.40.50.720	3.40.50.261
3oxhA	3oxhA01_3oxhA02	3.10.180.10	3.10.180.10
1x7oA	1x7oA01_1x7oA02	3.30.1330.30	3.40.1280.10
2qfpA	2qfpA01_2qfpA02	2.60.40.380	3.60.21.10
2bhgA	2bhgA01_2bhgA02	2.40.10.10	2.40.10.10
3b8bA	3b8bA01_3b8bA02	3.30.540.10	3.40.190.80
1hx6B	1hx6B01_1hx6B02	2.70.9.30	2.60.120.20
4m4zA	4m4zA01_4m4zA02	2.30.30.40	3.30.505.10
4hppA	4hppA01_4hppA02	3.10.20.70	3.30.590.10
4eq3A	4eq3A01_4eq3A02	2.60.40.10	2.60.40.10
3a7iA	3a7iA01_3a7iA02	3.30.200.20	1.10.510.10
4im7A	4im7A01_4im7A02	3.40.50.720	1.10.1040.10
3e18B	3e18B01_3e18B02	3.40.50.720	3.30.360.10
2yexA	2yexA01_2yexA02	3.30.200.20	1.10.510.10
2b9eA	2b9eA01_2b9eA02	3.30.70.1170	3.40.50.150
3b77A	3b77A01_3b77A02	2.30.29.50	1.10.287.210
3e6cC	3e6cC01_3e6cC02	2.60.120.10	1.10.10.10
1b9wA	1b9wA01_1b9wA02	2.10.25.10	2.10.25.10
2ahrA	2ahrA01_2ahrA02	3.40.50.720	1.10.3730.10
1d3yA	1d3yA01_1d3yA02	1.10.10.10	3.40.1360.10
4f03A	4f03A01_4f03A02	3.40.30.10	1.20.1050.10
1gjwA	1gjwA01_1gjwA02	3.20.20.80	2.60.40.1180
4d25A	4d25A01_4d25A02	3.40.50.300	3.40.50.300
4h59A	4h59A01_4h59A02	3.40.50.1980	3.40.50.1980
3repA	3repA01_3repA02	3.30.200.20	1.10.510.10
1ycsB	1ycsB01_1ycsB02	1.25.40.20	2.30.30.40
2o3iA	2o3iA01_2o3iA02	3.40.1610.10	2.40.390.10
2r2nA	2r2nA02_2r2nA03	3.40.640.10	3.90.1150.10
1kbvA	1kbvA01_1kbvA02	2.60.40.420	2.60.40.420
1kbiA	1kbiA01_1kbiA02	3.10.120.10	3.20.20.70
1j5xA	1j5xA01_1j5xA02	3.40.50.10490	3.40.50.10490
3pyfA	3pyfA01_3pyfA02	3.30.230.10	3.30.70.890
3eypA	3eypA01_3eypA02	3.20.20.80	2.60.120.260
3of1A	3of1A01_3of1A02	2.60.120.10	2.60.120.10

1cs1A	1cs1A01_1cs1A02	3.40.640.10	3.90.1150.10
4inoA	4inoA01_4inoA02	3.40.50.1980	3.40.50.1980
1n2zA	1n2zA01_1n2zA02	3.40.50.1980	3.40.50.1980
1tuaA	1tuaA01_1tuaA02	3.30.1370.10	3.30.1370.10
4ob0B	4ob0B01_4ob0B02	1.10.472.20	2.30.30.50
3bofA	3bofA01_3bofA02	3.20.20.330	3.20.20.20
1t77A	1t77A01_1t77A02	2.30.29.40	1.10.1540.10
4f27A	4f27A01_4f27A02	2.60.40.1280	2.60.40.1290
4cqeA	4cqeA01_4cqeA02	3.30.200.20	1.10.510.10
1n93X	1n93X01_1n93X02	1.10.3040.10	1.10.3050.10
4eh1A	4eh1A01_4eh1A02	2.40.30.10	3.40.50.80
3qqaA	3qqaA01_3qqaA02	1.10.10.60	1.10.357.10
2v62B	2v62B01_2v62B02	3.30.200.20	1.10.510.10
1jq5A	1jq5A01_1jq5A02	3.40.50.1970	1.20.1090.10
1htrB	1htrB01_1htrB02	2.40.70.10	2.40.70.10
3aj6B	3aj6B01_3aj6B02	2.80.10.50	2.80.10.50
1a0pA	1a0pA01_1a0pA02	1.10.150.130	1.10.443.10
2p0oA	2p0oA01_2p0oA02	3.20.20.70	2.40.100.10
1u0mA	1u0mA01_1u0mA02	3.40.47.10	3.40.47.10
1s8nA	1s8nA01_1s8nA02	3.40.50.2300	1.10.10.10
1phkA	1phkA01_1phkA02	3.30.200.20	1.10.510.10
1grjA	1grjA01_1grjA02	1.10.287.180	3.10.50.30
3csvA	3csvA01_3csvA02	3.30.200.20	3.90.1200.10
3hz6A	3hz6A01_3hz6A02	3.30.420.40	3.30.420.40
1wd3A	1wd3A01_1wd3A02	2.60.120.200	2.80.10.50
1t6cA	1t6cA01_1t6cA02	3.30.420.40	3.30.420.150
3tqoA	3tqoA01_3tqoA02	3.40.50.620	1.20.120.640
4koaA	4koaA01_4koaA02	3.40.50.720	3.30.360.10
3sqdA	3sqdA01_3sqdA02	3.40.50.10190	3.40.50.10190
2vx2A	2vx2A01_2vx2A02	3.90.226.10	1.10.12.10
2xrwA	2xrwA01_2xrwA02	3.30.200.20	1.10.510.10
3n0vA	3n0vA01_3n0vA02	3.30.70.260	3.40.50.170
2gt1A	2gt1A01_2gt1A02	3.40.50.2000	3.40.50.2000
2zv2A	2zv2A01_2zv2A02	3.30.200.20	1.10.510.10
1mtyG	1mtyG01_1mtyG02	1.20.1280.10	1.20.1280.30
1u8xX	1u8xX01_1u8xX02	3.40.50.720	3.90.110.10
2orwB	2orwB01_2orwB02	3.40.50.300	3.30.60.20
3ip3B	3ip3B01_3ip3B02	3.40.50.720	3.30.360.10
4mu3A	4mu3A01_4mu3A02	3.30.230.40	3.30.230.40
4bq2A	4bq2A01_4bq2A02	2.60.120.430	3.20.20.80
2b6cB	2b6cB01_2b6cB02	1.20.1660.10	1.25.40.290
3qupA	3qupA01_3qupA02	3.30.200.20	1.10.510.10
3gzkA	3gzkA01_3gzkA02	2.60.40.10	1.50.10.10
3uuwA	3uuwA01_3uuwA02	3.40.50.720	3.30.360.10
4dqnA	4dqnA01_4dqnA02	3.30.470.10	3.20.10.10
4ewgA	4ewgA01_4ewgA02	3.40.47.10	3.40.47.10
3a35A	3a35A01_3a35A02	2.40.30.20	2.40.30.20
3t9wA	3t9wA01_3t9wA02	2.60.40.420	2.60.40.420
3uzuA	3uzuA01_3uzuA02	3.40.50.150	1.10.8.100
2ww4A	2ww4A01_2ww4A02	3.30.230.10	3.30.70.890
2qsvA	2qsvA01_2qsvA02	2.60.40.10	2.60.40.10
4mi2B	4mi2B01_4mi2B02	3.90.226.10	1.10.12.10
1tdhA	1tdhA01_1tdhA02	3.20.190.10	1.10.8.50

2cmgA	2cmgA01_2cmgA02	2.30.140.10	3.40.50.150
4a1kA	4a1kA01_4a1kA02	3.10.350.10	2.40.440.10
1g5hD	1g5hD01_1g5hD02	3.30.930.10	3.40.50.800
1lldA	1lldA01_1lldA02	3.40.50.720	3.90.110.10
1cbfA	1cbfA01_1cbfA02	3.40.1010.10	3.30.950.10
3q8gA	3q8gA01_3q8gA02	1.10.8.20	3.40.525.10
3lcvB	3lcvB01_3lcvB02	1.10.8.10	3.40.50.150
3mg1A	3mg1A01_3mg1A02	1.10.2090.10	3.10.450.50
2r01A	2r01A01_2r01A02	3.40.109.10	2.20.180.10
2o6yA	2o6yA01_2o6yA02	1.10.275.10	1.20.200.10
4jwvA	4jwvA01_4jwvA02	3.90.226.10	1.10.12.10
3fhrA	3fhrA01_3fhrA02	3.30.200.20	1.10.510.10
1khiA	1khiA01_1khiA02	2.30.30.30	2.40.50.140
4bxjB	4bxjB01_4bxjB02	3.40.80.10	1.10.101.10
4ivnA	4ivnA01_4ivnA02	1.10.10.10	3.40.50.10490
2d20B	2d20B01_2d20B02	3.20.20.80	2.80.10.50
3fanA	3fanA01_3fanA02	2.40.10.10	2.40.10.10
2bbrA	2bbrA01_2bbrA02	1.10.533.10	1.10.533.10
4fxqA	4fxqA01_4fxqA02	3.40.390.10	3.90.176.10
3vgjB	3vgjB01_3vgjB02	3.40.50.620	1.10.240.10
3op1A	3op1A01_3op1A02	3.40.50.620	2.40.30.30
1rkxC	1rkxC01_1rkxC02	3.40.50.720	3.90.25.10
1txgA	1txgA01_1txgA02	3.40.50.720	1.10.1040.10
4o38B	4o38B01_4o38B02	3.30.200.20	1.10.510.10
4mmwA	4mmwA01_4mmwA02	3.30.390.10	3.20.20.120
2a0uA	2a0uA01_2a0uA02	1.20.120.420	3.40.50.10470
3ndcA	3ndcA01_3ndcA02	3.40.1010.10	3.30.950.10
4htlA	4htlA01_4htlA02	3.30.420.40	3.30.420.40
3k96A	3k96A01_3k96A02	3.40.50.720	1.10.1040.10
1go3F	1go3F01_1go3F02	1.10.150.80	1.10.287.420
1t0tV	1t0tV01_1t0tV02	3.30.70.1030	3.30.70.1100
4m1qA	4m1qA01_4m1qA02	3.40.50.720	3.90.110.10
2i1oA	2i1oA01_2i1oA02	3.90.1170.20	3.20.20.70
3i8bA	3i8bA01_3i8bA02	3.30.420.40	3.30.420.40
3b4nA	3b4nA01_3b4nA02	2.60.40.10	2.160.20.10
3fb2B	3fb2B01_3fb2B02	1.20.58.60	1.20.58.60
4izbB	4izbB01_4izbB02	3.90.226.10	1.10.12.10
4g0aA	4g0aA01_4g0aA02	3.90.1400.10	3.30.428.20
3lufB	3lufB01_3lufB02	3.40.50.2300	3.40.50.2300
2jbwB	2jbwB01_2jbwB02	1.20.1440.110	3.40.50.1820
1b9hA	1b9hA01_1b9hA02	3.40.640.10	3.90.1150.10
2pjpA	2pjpA01_2pjpA02	1.10.10.10	1.10.10.10
1qupA	1qupA01_1qupA02	3.30.70.100	2.60.40.200
2j5bA	2j5bA01_2j5bA02	3.40.50.620	1.10.240.10
2gizA	2gizA01_2gizA02	3.40.33.10	1.10.10.740
1vl2D	1vl2D01_1vl2D02	3.40.50.620	3.90.1260.10
2pn0A	2pn0A01_2pn0A02	1.10.286.20	3.10.50.30
2vxrA	2vxrA01_2vxrA02	2.60.120.200	2.80.10.50
4k2nA	4k2nA01_4k2nA02	3.90.226.10	1.10.12.10
1a41A	1a41A01_1a41A02	3.90.15.10	1.20.120.380
1r8sE	1r8sE01_1r8sE02	1.10.220.20	1.10.1000.11
3rleA	3rleA01_3rleA02	2.30.42.10	2.30.42.10
2chgA	2chgA01_2chgA02	3.40.50.300	1.10.8.60

1ef8A	1ef8A01_1ef8A02	1.10.12.10	3.90.226.10
4j15A	4j15A01_4j15A02	2.40.50.140	3.30.930.10
2x3eA	2x3eA01_2x3eA02	3.40.47.10	3.40.47.10
2zadA	2zadA01_2zadA02	3.30.390.10	3.20.20.120
1xkwA	1xkwA01_1xkwA02	2.170.130.10	2.40.170.20
4x0mA	4x0mA01_4x0mA02	3.30.200.20	1.10.510.10
1g0xA	1g0xA01_1g0xA02	2.60.40.10	2.60.40.10
1vs3A	1vs3A01_1vs3A02	3.30.70.580	3.30.70.660
3dhuA	3dhuA01_3dhuA02	3.20.20.80	2.60.40.1180
1t0fA	1t0fA01_1t0fA02	3.40.1350.10	1.10.10.10
4f0cA	4f0cA01_4f0cA02	3.40.30.10	1.20.1050.10
3ro6C	3ro6C01_3ro6C02	3.30.390.10	3.20.20.120
1j5wB	1j5wB01_1j5wB02	3.30.930.10	1.20.58.180
3ctkA	3ctkA01_3ctkA02	3.40.420.10	4.10.470.10
3hisA	3hisA01_3hisA02	3.40.420.10	4.10.470.10
3ftdA	3ftdA01_3ftdA02	3.40.50.150	1.10.8.100
1tkkA	1tkkA01_1tkkA02	3.30.390.10	3.20.20.120
1zx5A	1zx5A01_1zx5A02	2.60.120.10	2.60.120.10
2nrhB	2nrhB01_2nrhB02	3.30.420.40	3.30.420.40
2q9uA	2q9uA01_2q9uA02	3.60.15.10	3.40.50.360
3kptA	3kptA01_3kptA02	2.60.40.10	2.60.40.740
2bjuA	2bjuA01_2bjuA02	2.40.70.10	2.40.70.10
3lm5A	3lm5A01_3lm5A02	3.30.200.20	1.10.510.10
3gwbA	3gwbA01_3gwbA02	3.30.830.10	3.30.830.10
1ve2A	1ve2A01_1ve2A02	3.40.1010.10	3.30.950.10
3pmsA	3pmsA01_3pmsA02	2.60.120.230	2.60.120.230
1lbuA	1lbuA01_1lbuA02	1.10.101.10	3.30.1380.10
1axiB	1axiB01_1axiB02	2.60.40.10	2.60.40.10
1j1jA	1j1jA01_1j1jA02	1.20.58.190	1.20.58.200
2yd6A	2yd6A01_2yd6A02	2.60.40.10	2.60.40.10
3ry4A	3ry4A01_3ry4A02	2.60.40.10	2.60.40.10
4bxoA	4bxoA01_4bxoA02	3.40.50.10130	1.10.150.20
2y2zA	2y2zA01_2y2zA02	1.10.10.60	1.10.357.10
1aoaA	1aoaA01_1aoaA02	1.10.418.10	1.10.418.10
4lc1A	4lc1A01_4lc1A02	1.20.5.170	3.40.50.300
3cc1A	3cc1A01_3cc1A02	3.20.20.70	2.60.40.1180
3ndnA	3ndnA01_3ndnA02	3.40.640.10	3.90.1150.10
1vhvA	1vhvA01_1vhvA02	3.40.1010.10	3.30.950.10
3lstA	3lstA01_3lstA02	1.10.10.10	3.40.50.150
1gwcA	1gwcA01_1gwcA02	3.40.30.10	1.20.1050.10
2bw4A	2bw4A01_2bw4A02	2.60.40.420	2.60.40.420
1vhnA	1vhnA01_1vhnA02	3.20.20.70	1.10.1200.80
1a9xB	1a9xB01_1a9xB02	3.50.30.20	3.40.50.880
2qfjB	2qfjB01_2qfjB02	3.30.70.330	3.30.70.330
3g64A	3g64A01_3g64A02	3.90.226.10	1.10.12.10
3gwaA	3gwaA01_3gwaA02	3.40.47.10	3.40.47.10
3futA	3futA01_3futA02	3.40.50.150	1.10.8.100
5rubA	5rubA01_5rubA02	3.30.70.150	3.20.20.110
2j7tA	2j7tA01_2j7tA02	3.30.200.20	1.10.510.10
4k3wA	4k3wA01_4k3wA02	3.90.226.10	1.10.12.10
3htvA	3htvA01_3htvA02	3.30.420.40	3.30.420.40
1ykwA	1ykwA01_1ykwA02	3.30.70.150	3.20.20.110
3px8X	3px8X01_3px8X02	3.40.420.10	4.10.470.10

3fr7A	3fr7A01_3fr7A02	3.40.50.720	1.10.1040.10
3q9sA	3q9sA01_3q9sA02	3.40.50.2300	1.10.10.10
1oizA	1oizA01_1oizA02	1.10.8.20	3.40.525.10
2ej5B	2ej5B01_2ej5B02	3.90.226.10	1.10.12.10
2ppyA	2ppyA01_2ppyA02	3.90.226.10	1.10.12.10
4hdoA	4hdoA02_4hdoA03	1.20.80.10	2.30.29.30
1z0xA	1z0xA01_1z0xA02	1.10.10.60	1.10.357.10
4ct2A	4ct2A01_4ct2A02	3.30.200.20	1.10.510.10
3axjB	3axjB01_3axjB02	1.20.58.190	1.20.58.200
1ipaA	1ipaA01_1ipaA02	3.30.1330.30	3.40.1280.10
2iu4A	2iu4A01_2iu4A02	3.40.50.10440	3.30.1180.20
3hunA	3hunA01_3hunA02	3.40.710.10	2.30.140.20
3bb8A	3bb8A01_3bb8A02	3.40.640.10	3.90.1150.10
3nt8A	3nt8A01_3nt8A02	3.40.33.10	3.40.33.10
2vwiD	2vwiD01_2vwiD02	3.30.200.20	1.10.510.10
4ijnA	4ijnA01_4ijnA02	3.30.420.40	3.30.420.40
2iksA	2iksA01_2iksA02	3.40.50.2300	3.40.50.2300
3ir4A	3ir4A01_3ir4A02	3.40.30.10	1.20.1050.10
4jcsA	4jcsA01_4jcsA02	3.90.226.10	1.10.12.10
3g0oA	3g0oA01_3g0oA02	3.40.50.720	1.10.1040.10
2v3cD	2v3cD01_2v3cD02	1.20.120.140	3.40.50.300
2hsiB	2hsiB01_2hsiB02	2.60.40.1590	2.70.70.10
1af7A	1af7A01_1af7A02	1.10.155.10	3.40.50.150
2xpwA	2xpwA01_2xpwA02	1.10.10.60	1.10.357.10
3qk8A	3qk8A01_3qk8A02	3.90.226.10	1.10.12.10
1b63A	1b63A01_1b63A02	3.30.565.10	3.30.230.10
3qhaA	3qhaA01_3qhaA02	3.40.50.720	1.10.1040.10
3k6zA	3k6zA01_3k6zA02	2.40.10.10	2.40.10.10
3getA	3getA01_3getA02	3.40.640.10	3.90.1150.10
1p4xA	1p4xA01_1p4xA02	1.10.10.10	1.10.10.10
1bquB	1bquB01_1bquB02	2.60.40.10	2.60.40.10
3hp7A	3hp7A01_3hp7A02	3.10.290.10	3.40.50.150
3khpA	3khpA01_3khpA02	3.10.129.10	3.10.129.10
4nhoA	4nhoA01_4nhoA02	3.40.50.300	3.40.50.300
1mixA	1mixA01_1mixA02	1.20.80.10	2.30.29.30
3ulkA	3ulkA01_3ulkA02	3.40.50.720	1.10.1040.10
4o42A	4o42A01_4o42A02	2.40.50.40	2.40.50.40
2x9zA	2x9zA01_2x9zA02	2.60.40.740	2.60.40.1140
2qikA	2qikA01_2qikA02	3.10.490.10	3.10.490.10
3rioA	3rioA01_3rioA02	2.30.24.10	1.10.1790.10
1ev7A	1ev7A01_1ev7A02	3.40.600.10	1.10.10.10
3gonA	3gonA01_3gonA02	3.30.230.10	3.30.70.890
1x0cA	1x0cA01_1x0cA02	2.60.350.10	2.160.20.10
1tqyB	1tqyB01_1tqyB02	3.40.47.10	3.40.47.10
1vb5B	1vb5B01_1vb5B02	1.20.120.420	3.40.50.10470
4hzdA	4hzdA01_4hzdA02	1.10.3130.10	2.160.10.10
1nu5A	1nu5A01_1nu5A02	3.30.390.10	3.20.20.120
1zxqA	1zxqA01_1zxqA02	2.60.40.10	2.60.40.10
2prrA	2prrA01_2prrA02	1.20.5.810	1.20.1290.10
2c3nA	2c3nA01_2c3nA02	3.40.30.10	1.20.1050.10
3lxzA	3lxzA01_3lxzA02	3.40.30.10	1.20.1050.10
2qjfB	2qjfB01_2qjfB02	3.10.400.10	3.40.50.620
2x8sA	2x8sA01_2x8sA02	2.115.10.20	2.40.128.10

3iveA	3iveA01_3iveA02	3.60.21.10	3.90.780.10
3hwrA	3hwrA01_3hwrA02	3.40.50.720	1.10.1040.10
2j4dB	2j4dB02_2j4dB03	1.25.40.80	1.10.579.10
3ajvB	3ajvB01_3ajvB02	3.40.1170.20	3.40.1350.10
3h78B	3h78B01_3h78B02	3.40.47.10	3.40.47.10
4edgA	4edgA01_4edgA02	3.90.980.10	3.40.1360.10
3wiuA	3wiuA01_3wiuA02	3.30.70.80	3.40.50.200
4i5pA	4i5pA01_4i5pA02	3.30.200.20	1.10.510.10
4qhsA	4qhsA01_4qhsA02	3.40.50.300	1.10.8.60
3oryA	3oryA01_3oryA02	3.40.50.1010	1.10.150.20
2nlkA	2nlkA01_2nlkA02	3.90.190.10	3.90.190.10
2y8yA	2y8yA01_2y8yA02	3.30.70.1200	3.30.70.1210
2hxoA	2hxoA01_2hxoA02	1.10.10.60	1.10.357.10
3cloA	3cloA01_3cloA02	3.30.450.20	1.10.10.10
1vemA	1vemA01_1vemA02	3.20.20.80	2.60.40.10
3o9zA	3o9zA01_3o9zA02	3.40.50.720	3.30.360.10
2zbvA	2zbvA01_2zbvA02	3.40.50.10790	2.40.30.90
3og6B	3og6B01_3og6B02	2.60.40.10	2.60.40.10
4u19A	4u19A01_4u19A02	3.90.226.10	1.10.12.10
2gufA	2gufA01_2gufA02	2.170.130.10	2.40.170.20
2po3A	2po3A01_2po3A02	3.40.640.10	3.90.1150.10
3n5oA	3n5oA01_3n5oA02	3.40.30.10	1.20.1050.10
3v7dB	3v7dB01_3v7dB02	1.20.1280.50	2.130.10.10
1x0vA	1x0vA01_1x0vA02	3.40.50.720	1.10.1040.10
1t5oA	1t5oA01_1t5oA02	1.20.120.420	3.40.50.10470
1sfeA	1sfeA01_1sfeA02	3.30.160.70	1.10.10.10
2ozlB	2ozlB01_2ozlB02	3.40.50.970	3.40.50.920
2ynmD	2ynmD01_2ynmD02	3.40.50.1980	3.40.50.1980
3h38A	3h38A01_3h38A02	3.30.460.10	1.10.3090.10
2cz9A	2cz9A01_2cz9A02	3.30.230.10	3.30.70.890
2gupA	2gupA01_2gupA02	3.30.420.40	3.30.420.40
3lqcB	3lqcB01_3lqcB02	3.30.460.10	3.30.210.10
1fdrA	1fdrA01_1fdrA02	2.40.30.10	3.40.50.80
4gyiA	4gyiA01_4gyiA02	1.10.10.10	3.30.200.20
3ch8A	3ch8A01_3ch8A02	2.30.42.10	2.60.40.10
1uasA	1uasA01_1uasA02	3.20.20.70	2.60.40.1180
3gasA	3gasA01_3gasA02	3.20.180.10	2.30.110.10
3apqA	3apqA01_3apqA02	1.10.287.110	3.40.30.10
1evlA	1evlA01_1evlA02	3.30.930.10	3.40.50.800
2itmA	2itmA01_2itmA02	3.30.420.40	3.30.420.40
3tjoA	3tjoA01_3tjoA02	2.40.10.10	2.40.10.10
2zb9A	2zb9A01_2zb9A02	1.10.10.60	1.10.357.10
3uxjA	3uxjA01_3uxjA02	3.30.1130.10	3.30.1130.10
1vdwA	1vdwA01_1vdwA02	3.30.540.10	3.40.190.80
3r9rA	3r9rA01_3r9rA02	3.30.200.20	3.30.470.20
3bqzA	3bqzA01_3bqzA02	1.10.10.60	1.10.357.10
2q8uA	2q8uA01_2q8uA02	3.60.21.10	3.30.160.210
2jamA	2jamA01_2jamA02	3.30.200.20	1.10.510.10
2rioA	2rioA01_2rioA02	3.30.200.20	1.10.510.10
4k0nA	4k0nA01_4k0nA02	3.40.30.10	1.20.1050.10
2h8lA	2h8lA01_2h8lA02	3.40.30.10	3.40.30.10
2cnqA	2cnqA01_2cnqA02	3.30.200.20	3.30.470.20
3r8yB	3r8yB01_3r8yB02	3.30.70.250	2.160.10.10

1svvB	1svvB01_1svvB02	3.40.640.10	3.90.1150.10
2id4A	2id4A01_2id4A02	3.40.50.200	2.60.120.260
1o5zA	1o5zA01_1o5zA02	3.40.1190.10	3.90.190.20
1hr6A	1hr6A01_1hr6A02	3.30.830.10	3.30.830.10
2hdiA	2hdiA01_2hdiA02	2.170.130.10	2.40.170.20
2fytA	2fytA01_2fytA02	3.40.50.150	2.70.160.11
1vmaA	1vmaA01_1vmaA02	1.20.120.140	3.40.50.300
2h09A	2h09A01_2h09A02	1.10.10.10	1.10.60.10
1ihuA	1ihuA01_1ihuA02	3.40.50.300	3.40.50.300
4hymA	4hymA01_4hymA02	3.30.565.10	3.30.230.10
4cs4A	4cs4A01_4cs4A02	1.10.287.540	3.30.930.10
3shrA	3shrA01_3shrA02	2.60.120.10	2.60.120.10
1oewA	1oewA01_1oewA02	2.40.70.10	2.40.70.10
3fiwA	3fiwA01_3fiwA02	1.10.10.60	1.10.357.10
1ollA	1ollA01_1ollA02	2.60.40.10	2.60.40.10
4aa9A	4aa9A01_4aa9A02	2.40.70.10	2.40.70.10
3czpA	3czpA01_3czpA02	3.40.50.300	3.40.50.300
2v9vA	2v9vA01_2v9vA02	1.10.10.10	1.10.10.10
2je6I	2je6I02_2je6I03	2.40.50.140	3.30.1370.10
3w5hA	3w5hA01_3w5hA02	2.40.30.10	3.40.50.80
1gupA	1gupA01_1gupA02	3.30.428.10	3.30.428.10
1ijqA	1ijqA01_1ijqA02	2.120.10.30	2.10.25.10
2zsgA	2zsgA01_2zsgA02	3.40.350.10	3.90.230.10
3nyiB	3nyiB01_3nyiB02	3.40.50.10170	3.30.1180.10
3gidB	3gidB02_3gidB03	3.30.1490.20	3.30.470.20
1dpgA	1dpgA01_1dpgA02	3.40.50.720	3.30.360.10
1ydwB	1ydwB01_1ydwB02	3.40.50.720	3.30.360.10
1xt0B	1xt0B01_1xt0B02	1.10.220.20	1.10.1000.11
1gtmA	1gtmA01_1gtmA02	3.40.50.10860	3.40.50.720
4hi7A	4hi7A01_4hi7A02	3.40.30.10	1.20.1050.10
2fyfA	2fyfA01_2fyfA02	3.40.640.10	3.90.1150.10
1wuiS	1wuiS01_1wuiS02	3.40.50.700	4.10.480.10
2qggA	2qggA01_2qggA02	2.40.30.60	2.30.30.240
1e5rA	1e5rA01_1e5rA02	2.60.120.330	1.10.1720.10
2yxzA	2yxzA01_2yxzA02	3.30.1330.10	3.90.650.10
3bwhA	3bwhA01_3bwhA02	3.40.420.10	4.10.470.10
2nynA	2nynA01_2nynA02	1.10.275.10	1.20.200.10
1nowA	1nowA01_1nowA02	3.30.379.10	3.20.20.80
3nrsA	3nrsA01_3nrsA02	3.40.1190.10	3.90.190.20
1iarB	1iarB01_1iarB02	2.60.40.10	2.60.40.10
1chmA	1chmA01_1chmA02	3.40.350.10	3.90.230.10
1cr5A	1cr5A01_1cr5A02	2.40.40.20	3.10.330.10
3b8xB	3b8xB01_3b8xB02	3.40.640.10	3.90.1150.10
1kkhA	1kkhA01_1kkhA02	3.30.230.10	3.30.70.890
2bj7B	2bj7B01_2bj7B02	1.10.1220.10	3.30.70.1150
1inlC	1inlC01_1inlC02	3.40.50.150	2.30.140.10
3focA	3focA01_3focA02	3.40.50.620	1.10.240.10
2q83A	2q83A01_2q83A02	3.30.200.20	3.90.1200.10
1kl9A	1kl9A01_1kl9A02	2.40.50.140	1.10.150.190
1yisA	1yisA01_1yisA02	1.10.275.10	1.20.200.10
2qveA	2qveA01_2qveA02	1.10.275.10	1.20.200.10
3vtfA	3vtfA01_3vtfA02	3.40.50.720	3.40.50.720
3f4lA	3f4lA01_3f4lA02	3.40.50.720	3.30.360.10

4hl0A	4hl0A01_4hl0A02	2.60.120.200	2.60.120.200
3a04A	3a04A01_3a04A02	3.40.50.620	1.10.240.10
2akzA	2akzA01_2akzA02	3.30.390.10	3.20.20.120
1vljA	1vljA01_1vljA02	3.40.50.1970	1.20.1090.10
3lqmA	3lqmA01_3lqmA02	2.60.40.10	2.60.40.10
1p2fA	1p2fA01_1p2fA02	3.40.50.2300	1.10.10.10
4agkA	4agkA01_4agkA02	2.40.10.10	2.40.10.10
3zw8B	3zw8B01_3zw8B02	3.90.226.10	3.40.50.720
2qtwB	2qtwB01_2qtwB02	3.40.50.200	2.60.120.690
3axjA	3axjA01_3axjA02	1.20.58.190	1.20.58.200
2dt8A	2dt8A01_2dt8A02	3.40.50.10170	3.30.1180.10
2himA	2himA01_2himA02	3.40.50.1170	3.40.50.40
3kheB	3kheB01_3kheB02	1.10.238.10	1.10.238.10
1w2tA	1w2tA01_1w2tA02	2.115.10.20	2.60.120.560
1dj0A	1dj0A01_1dj0A02	3.30.70.660	3.30.70.580
1a2oA	1a2oA01_1a2oA02	3.40.50.2300	3.40.50.180
4f9zA	4f9zA01_4f9zA02	3.40.30.10	3.40.30.10
1y88A	1y88A01_1y88A02	3.40.1350.10	1.10.150.20
4kbfA	4kbfA01_4kbfA02	3.40.50.300	3.40.50.300
2zodB	2zodB01_2zodB02	3.30.1330.10	3.90.650.10
3c2uA	3c2uA01_3c2uA02	2.115.10.20	2.60.120.200
3fseB	3fseB01_3fseB02	3.40.50.880	1.20.1260.10
1wddA	1wddA01_1wddA02	3.30.70.150	3.20.20.110
3dewA	3dewA01_3dewA02	1.10.10.60	1.10.357.10
3gz5B	3gz5B01_3gz5B02	3.90.79.10	1.10.10.10
1pq4B	1pq4B01_1pq4B02	3.40.50.1980	3.40.50.1980
1qksA	1qksA01_1qksA02	1.10.760.10	2.140.10.20
2wc7A	2wc7A01_2wc7A02	3.20.20.80	2.60.40.1180
3ubkA	3ubkA01_3ubkA02	3.40.30.10	1.20.1050.10
1wleB	1wleB01_1wleB02	1.10.287.40	3.30.930.10
4dwdB	4dwdB01_4dwdB02	3.30.390.10	3.20.20.120
2qm4A	2qm4A01_2qm4A02	2.170.210.10	1.10.287.450
4u9uA	4u9uA01_4u9uA02	2.40.30.10	3.40.50.80
3cp7B	3cp7B01_3cp7B02	2.40.10.10	2.40.10.10
1o6vA	1o6vA01_1o6vA02	3.80.10.10	2.60.40.1220
2vapA	2vapA01_2vapA02	3.40.50.1440	3.30.1330.20
1zy4A	1zy4A01_1zy4A02	3.30.200.20	1.10.510.10
3cx5A	3cx5A01_3cx5A02	3.30.830.10	3.30.830.10
2hbwA	2hbwA01_2hbwA02	2.30.30.40	3.90.1720.10
1i8dA	1i8dA01_1i8dA02	2.40.30.20	2.40.30.20
1nuwA	1nuwA01_1nuwA02	3.30.540.10	3.40.190.80
2dvmA	2dvmA01_2dvmA02	3.40.50.10380	3.40.50.720
1fnlA	1fnlA01_1fnlA02	2.60.40.10	2.60.40.10
4q7fA	4q7fA01_4q7fA02	3.60.21.10	3.90.780.10
2i7cA	2i7cA01_2i7cA02	2.30.140.10	3.40.50.150
3sqlA	3sqlA01_3sqlA02	3.20.20.300	3.40.50.10870
3la7A	3la7A01_3la7A02	2.60.120.10	1.10.10.10
2r9qB	2r9qB01_2r9qB02	2.70.40.10	2.70.40.10
3vpdA	3vpdA01_3vpdA02	3.40.50.20	3.30.1490.20
1kzlA	1kzlA01_1kzlA02	2.40.30.20	2.40.30.20
4gv0A	4gv0A01_4gv0A02	1.20.142.10	3.90.228.10
4mlzA	4mlzA01_4mlzA02	3.40.50.1980	3.40.50.1980
2gsmB	2gsmB01_2gsmB02	1.10.287.90	2.60.40.420

3hn2A	3hn2A01_3hn2A02	3.40.50.720	1.10.1040.10
3amiA	3amiA01_3amiA02	3.30.830.10	3.30.830.10
4bolA	4bolA01_4bolA02	3.40.80.10	1.10.101.10
3zl8A	3zl8A02_3zl8A03	3.40.1190.10	3.90.190.20
3ledA	3ledA01_3ledA02	3.40.47.10	3.40.47.10
3zdbA	3zdbA01_3zdbA02	3.40.50.1010	1.10.150.20
3besR	3besR01_3besR02	2.60.40.10	2.60.40.10
4iqzA	4iqzA01_4iqzA02	2.40.50.100	2.40.50.100
3s95A	3s95A01_3s95A02	3.30.200.20	1.10.510.10
2a06A	2a06A01_2a06A02	3.30.830.10	3.30.830.10
3e97A	3e97A01_3e97A02	2.60.120.10	1.10.10.10
1m70A	1m70A01_1m70A02	1.10.760.10	1.10.760.10
4eeiA	4eeiA01_4eeiA02	1.10.275.10	1.20.200.10
3fi9A	3fi9A01_3fi9A02	3.40.50.720	3.90.110.10
2e8yA	2e8yA02_2e8yA03	2.60.40.10	3.20.20.80
3tj8B	3tj8B01_3tj8B02	2.60.260.20	3.30.70.790
1o59A	1o59A01_1o59A02	2.60.120.260	2.60.120.260
1lamA	1lamA01_1lamA02	3.40.220.10	3.40.630.10
2w5fB	2w5fB01_2w5fB02	2.60.120.260	3.20.20.80
3euoA	3euoA01_3euoA02	3.40.47.10	3.40.47.10
1s7hA	1s7hA01_1s7hA02	3.30.70.930	3.30.70.930
2wtbA	2wtbA01_2wtbA02	3.90.226.10	3.40.50.720
1u00A	1u00A01_1u00A02	2.60.34.10	1.20.1270.10
2r0yA	2r0yA01_2r0yA02	1.20.920.10	1.20.920.10
3einA	3einA01_3einA02	3.40.30.10	1.20.1050.10
3dcfA	3dcfA01_3dcfA02	1.10.10.60	1.10.357.10
3v0dA	3v0dA01_3v0dA02	3.90.190.10	2.60.40.1110
2of7A	2of7A01_2of7A02	1.10.10.60	1.10.357.10
2hlsA	2hlsA01_2hlsA02	3.40.30.10	3.40.30.10
4iibA	4iibA01_4iibA02	3.20.20.300	3.40.50.1700
2e5fA	2e5fA01_2e5fA02	3.40.50.10490	3.40.50.10490

759 consecutive continuous domain (domain-CC-M)			
**PDBID**	**Domain pair**	**Domain 1 CATH classification**	**Domain 2 CATH classification**
1a5tA	1a5tA02_1a5tA03	1.10.8.10	1.20.272.10
2pw9B	2pw9B02_2pw9B03	3.10.20.10	3.40.140.10
3hwcA	3hwcA01_3hwcA02	1.10.3140.10	2.40.110.10
1ba3A	1ba3A01_1ba3A02	3.40.50.980	3.40.50.980
1ba3A	1ba3A05_1ba3A01	4.10.8.10	3.40.50.980
1jmxA	1jmxA02_1jmxA03	2.40.128.120	2.60.40.10
1gvhA	1gvhA02_1gvhA03	2.40.30.10	3.40.50.80
3hi7A	3hi7A01_3hi7A02	3.10.450.40	3.10.450.40
3hn7A	3hn7A02_3hn7A03	3.40.1190.10	3.90.190.20
3hn7A	3hn7A01_3hn7A02	3.40.50.720	3.40.1190.10
2vrwB	2vrwB02_2vrwB03	2.30.29.30	3.30.60.20
1r8eA	1r8eA03_1r8eA01	1.20.5.490	3.20.80.10
1in6A	1in6A03_1in6A02	1.10.8.60	1.10.10.10
1js3A	1js3A02_1js3A03	3.40.640.10	3.90.1150.10
4e1oA	4e1oA01_4e1oA02	1.20.1340.10	3.40.640.10
4e1oA	4e1oA02_4e1oA03	3.40.640.10	3.90.1150.10
2wlrA	2wlrA01_2wlrA02	3.40.250.10	3.40.250.10
2wlrA	2wlrA02_2wlrA03	3.40.250.10	3.40.250.10
1vclA	1vclA01_1vclA02	2.80.10.50	2.80.10.50
1qhoA	1qhoA01_1qhoA02	3.20.20.80	2.60.40.1180
4tr8B	4tr8B02_4tr8B03	3.10.150.10	3.10.150.10
4tr8B	4tr8B01_4tr8B02	3.10.150.10	3.10.150.10
2o7sA	2o7sA01_2o7sA02	3.20.20.70	3.40.50.10860
4dmgA	4dmgA02_4dmgA03	3.30.750.80	3.40.50.150
1wxxA	1wxxA02_1wxxA03	3.30.750.80	3.40.50.150
4aefA	4aefA03_4aefA04	3.20.20.80	2.60.40.1180
4aefA	4aefA02_4aefA03	2.60.40.10	3.20.20.80
2q9oA	2q9oA02_2q9oA03	2.60.40.420	2.60.40.420
4fnqA	4fnqA01_4fnqA02	2.70.98.60	3.20.20.70
4fnqA	4fnqA02_4fnqA03	3.20.20.70	2.60.40.1180
2yfoA	2yfoA02_2yfoA03	3.20.20.70	2.60.40.1180
2yfoA	2yfoA01_2yfoA02	2.70.98.60	3.20.20.70
4k9qA	4k9qA01_4k9qA02	3.40.50.970	3.40.50.1220
1q6zA	1q6zA01_1q6zA02	3.40.50.970	3.40.50.1220
1lxmA	1lxmA01_1lxmA03	2.70.98.10	2.60.220.10
1n7oA	1n7oA01_1n7oA03	2.70.98.10	2.60.220.10
1ei5A	1ei5A02_1ei5A03	2.40.128.50	2.40.128.50
1olzA	1olzA02_1olzA03	3.30.1680.10	2.60.40.10
4bucA	4bucA01_4bucA02	3.40.50.720	3.40.1190.10
1e8cA	1e8cA01_1e8cA02	3.40.1390.10	3.40.1190.10
1e8cA	1e8cA02_1e8cA03	3.40.1190.10	3.90.190.20
2fnaA	2fnaA01_2fnaA02	3.40.50.300	1.10.8.60
1gntA	1gntA03_1gntA04	3.40.50.2030	3.40.50.2030

2fh1A	2fh1A02_2fh1A03	3.40.20.10	3.40.20.10
3cb2A	3cb2A01_3cb2A02	3.40.50.1440	3.30.1330.20
2fh8A	2fh8A03_2fh8A04	3.20.20.80	2.60.40.1180
2fh8A	2fh8A02_2fh8A03	2.60.40.10	3.20.20.80
1l5hA	1l5hA01_1l5hA03	3.40.50.1980	3.40.50.1980
2dy1A	2dy1A02_2dy1A03	2.40.30.10	3.30.70.870
4cokA	4cokA02_4cokA03	3.40.50.1220	3.40.50.970
1xszA	1xszA01_1xszA02	1.10.220.20	1.10.1000.11
2r09A	2r09A01_2r09A02	1.10.220.20	1.10.1000.11
2ywcC	2ywcC01_2ywcC02	3.40.50.880	3.40.50.620
1iq8A	1iq8A01_1iq8A02	3.20.20.105	3.90.1020.10
3erbA	3erbA02_3erbA03	2.10.70.10	2.10.70.10
3zyzA	3zyzA02_3zyzA03	3.40.50.1700	2.60.40.10
1bg1A	1bg1A01_1bg1A02	1.20.1050.20	2.60.40.630
2w91A	2w91A02_2w91A03	2.60.120.260	2.60.40.10
4b7lA	4b7lA01_4b7lA02	1.10.418.10	1.10.418.10
3lpfA	3lpfA01_3lpfA02	2.60.120.260	2.60.40.10
4jkmA	4jkmA01_4jkmA02	2.60.120.260	2.60.40.10
3iecA	3iecA01_3iecA02	3.30.200.20	1.10.510.10
1musA	1musA02_1musA03	3.90.350.10	1.10.740.10
1musA	1musA01_1musA02	1.10.246.40	3.90.350.10
2fuvA	2fuvA01_2fuvA02	3.40.120.10	3.40.120.10
1c97A	1c97A02_1c97A03	3.40.1060.10	3.30.499.10
3lk7A	3lk7A01_3lk7A02	3.40.50.720	3.40.1190.10
2qtcA	2qtcA02_2qtcA03	3.40.50.970	3.40.50.920
2hmaA	2hmaA02_2hmaA03	2.30.30.280	2.40.30.10
2ck3A	2ck3A01_2ck3A02	2.40.30.20	3.40.50.300
2ck3A	2ck3A02_2ck3A03	3.40.50.300	1.20.150.20
1ybyB	1ybyB02_1ybyB03	2.40.50.140	2.40.50.140
1f7uA	1f7uA01_1f7uA02	3.40.50.620	1.10.730.10
3nvqA	3nvqA01_3nvqA02	2.130.10.10	3.30.1680.10
2dwcA	2dwcA02_2dwcA03	3.30.1490.20	3.30.470.20
1kj8A	1kj8A02_1kj8A03	3.30.1490.20	3.30.470.20
1amuA	1amuA02_1amuA03	3.40.50.980	2.30.38.10
1tuoA	1tuoA01_1tuoA02	3.40.120.10	3.40.120.10
1rwhA	1rwhA01_1rwhA02	1.50.10.100	2.70.98.10
3js8A	3js8A03_3js8A04	3.40.462.10	1.10.45.10
1kfiA	1kfiA01_1kfiA02	3.40.120.10	3.40.120.10
3s98A	3s98A02_3s98A03	2.60.40.10	2.60.40.10
1hn0A	1hn0A01_1hn0A02	2.60.120.430	1.50.10.100
1hn0A	1hn0A02_1hn0A03	1.50.10.100	2.70.98.10
1f8nA	1f8nA03_1f8nA04	4.10.372.10	3.10.450.60
1olzA	1olzA01_1olzA02	2.130.10.10	3.30.1680.10
2yv5A	2yv5A01_2yv5A02	2.40.50.140	3.40.50.300
2rcnA	2rcnA01_2rcnA02	2.40.50.140	3.40.50.300
2rcnA	2rcnA02_2rcnA03	3.40.50.300	1.10.40.50

1o7fA	1o7fA02_1o7fA03	1.10.10.10	2.60.120.10
1t5hX	1t5hX01_1t5hX02	3.40.50.980	3.40.50.980
1sqgA	1sqgA03_1sqgA04	3.30.70.1170	3.40.50.150
2uz1A	2uz1A02_2uz1A03	3.40.50.1220	3.40.50.970
3rh4A	3rh4A02_3rh4A03	1.10.150.20	3.30.460.10
3rh4A	3rh4A03_3rh4A04	3.30.460.10	3.30.210.10
2i0kA	2i0kA03_2i0kA04	3.40.462.10	1.10.45.10
3js8A	3js8A02_3js8A03	3.30.465.10	3.40.462.10
1kxpD	1kxpD04_1kxpD05	1.10.246.10	1.10.246.10
3c1dA	3c1dA02_3c1dA03	1.10.10.10	1.10.10.10
2c12A	2c12A02_2c12A03	2.40.110.10	1.20.140.10
3gm8A	3gm8A03_3gm8A04	3.20.20.80	2.60.40.10
3gm8A	3gm8A02_3gm8A03	2.60.40.10	3.20.20.80
3tewA	3tewA03_3tewA04	3.10.20.110	2.60.40.810
1kfiA	1kfiA03_1kfiA04	3.40.120.10	3.30.310.50
4tr7A	4tr7A01_4tr7A02	3.10.150.10	3.10.150.10
1pbyA	1pbyA02_1pbyA03	2.40.128.120	2.60.40.10
2wskA	2wskA01_2wskA02	2.60.40.10	3.20.20.80
2wskA	2wskA02_2wskA03	3.20.20.80	2.60.40.1180
1bxrC	1bxrC02_1bxrC03	3.30.470.20	1.10.1030.10
1bxrC	1bxrC01_1bxrC02	3.40.50.20	3.30.470.20
4amwA	4amwA03_4amwA04	2.60.40.1180	2.60.40.1180
4cvuA	4cvuA04_4cvuA05	2.60.40.10	2.60.40.10
2q6fA	2q6fA01_2q6fA02	2.40.10.10	2.40.10.10
4f49A	4f49A01_4f49A02	2.40.10.10	2.40.10.10
3mjgX	3mjgX01_3mjgX02	2.60.40.10	2.60.40.10
2fmlA	2fmlA02_2fmlA03	3.90.79.10	1.10.10.10
3apoA	3apoA02_3apoA03	3.40.30.10	3.40.30.10
4gafB	4gafB01_4gafB02	2.60.40.10	2.60.40.10
4ogzA	4ogzA01_4ogzA02	2.60.40.10	3.20.20.70
4ogzA	4ogzA02_4ogzA03	3.20.20.70	2.60.40.1180
4i4tB	4i4tB02_4i4tB03	3.30.1330.20	1.10.287.600
3b0xA	3b0xA04_3b0xA05	3.30.210.10	3.20.20.140
4e9sA	4e9sA01_4e9sA02	2.60.40.420	2.60.40.420
1h2vC	1h2vC02_1h2vC03	1.25.40.180	1.25.40.180
1jb7A	1jb7A02_1jb7A03	2.40.50.140	2.40.50.140
1ffvC	1ffvC03_1ffvC02	3.30.465.10	3.30.390.50
1ffvC	1ffvC01_1ffvC03	3.30.43.10	3.30.465.10
1soxA	1soxA01_1soxA02	3.10.120.10	3.90.420.10
1soxA	1soxA02_1soxA03	3.90.420.10	2.60.40.650
2ff4A	2ff4A01_2ff4A02	1.10.10.10	1.25.40.10
2ff4A	2ff4A02_2ff4A03	1.25.40.10	2.60.200.20
1g7sA	1g7sA01_1g7sA02	3.40.50.300	2.40.30.10
1hfuA	1hfuA02_1hfuA03	2.60.40.420	2.60.40.420
1mv8A	1mv8A02_1mv8A03	1.20.5.170	3.40.50.720
2qn6A	2qn6A01_2qn6A02	3.40.50.300	2.40.30.10

1t3qC	1t3qC01_1t3qC02	3.30.43.10	3.30.465.10
1t3qC	1t3qC02_1t3qC03	3.30.465.10	3.30.390.50
1w96A	1w96A02_1w96A03	3.90.1770.10	3.30.1490.20
4f5uA	4f5uA05_4f5uA06	1.10.246.10	1.10.246.10
4f5uA	4f5uA04_4f5uA05	1.10.246.10	1.10.246.10
1pixA	1pixA01_1pixA02	1.20.5.680	3.90.226.10
1r6tA	1r6tA02_1r6tA03	3.40.50.620	1.10.240.10
1ozhB	1ozhB02_1ozhB03	3.40.50.1220	3.40.50.970
2pg0A	2pg0A02_2pg0A03	2.40.110.10	1.20.140.10
2q07A	2q07A01_2q07A02	3.40.50.10630	3.10.450.90
2f2hC	2f2hC03_2f2hC04	2.60.40.1180	2.60.40.1180
2gqtA	2gqtA01_2gqtA02	3.30.43.10	3.30.465.10
3p7lA	3p7lA03_3p7lA04	3.30.830.10	3.30.830.10
2zxqA	2zxqA05_2zxqA06	2.60.120.260	1.20.1270.70
2zxqA	2zxqA04_2zxqA05	2.60.120.260	2.60.120.260
4e4tA	4e4tA02_4e4tA03	3.30.1490.20	3.30.470.20
4gwmA	4gwmA02_4gwmA03	2.60.120.200	2.60.210.10
3a9sA	3a9sA01_3a9sA02	3.40.50.1070	3.40.275.10
2eplX	2eplX01_2eplX02	3.30.160.230	3.20.20.80
2eplX	2eplX02_2eplX03	3.20.20.80	1.20.120.670
1t9hA	1t9hA02_1t9hA03	3.40.50.300	1.10.40.50
1t9hA	1t9hA01_1t9hA02	2.40.50.140	3.40.50.300
1a5tA	1a5tA01_1a5tA02	3.40.50.300	1.10.8.10
1j6uA	1j6uA01_1j6uA02	3.40.50.720	3.40.1190.10
1j6uA	1j6uA02_1j6uA03	3.40.1190.10	3.90.190.20
3ejjX	3ejjX01_3ejjX02	2.60.40.10	2.60.40.10
2x5oA	2x5oA01_2x5oA02	3.40.50.720	3.40.1190.10
2x5oA	2x5oA02_2x5oA03	3.40.1190.10	3.90.190.20
3cb2A	3cb2A02_3cb2A03	3.30.1330.20	1.10.287.600
3r7kA	3r7kA02_3r7kA03	2.40.110.10	1.20.140.10
1in6A	1in6A01_1in6A03	3.40.50.300	1.10.8.60
1d4vA	1d4vA03_1d4vA01	2.10.50.10	2.10.50.10
3fl7A	3fl7A02_3fl7A03	2.60.40.1770	2.10.50.10
1ofeB	1ofeB01_1ofeB02	3.60.20.10	3.20.20.70
1ofcX	1ofcX02_1ofcX03	1.10.10.60	1.20.5.1190
1lmlA	1lmlA01_1lmlA02	3.10.170.20	3.90.132.10
1pxyB	1pxyB03_1pxyB04	1.10.418.10	1.10.418.10
1rt8A	1rt8A03_1rt8A04	1.10.418.10	1.10.418.10
1ofcX	1ofcX03_1ofcX04	1.20.5.1190	1.10.10.60
4i2aA	4i2aA03_4i2aA04	3.30.460.10	3.30.210.10
4i2aA	4i2aA02_4i2aA03	1.10.150.20	3.30.460.10
3abqA	3abqA02_3abqA03	1.10.220.70	3.20.20.70
2c42A	2c42A06_2c42A07	3.40.50.970	4.10.790.10
2c42A	2c42A05_2c42A06	3.30.70.20	3.40.50.970
1pxyB	1pxyB01_1pxyB02	1.10.418.10	1.10.418.10
3nf4A	3nf4A01_3nf4A02	1.10.540.10	2.40.110.10

2or0A	2or0A01_2or0A02	1.10.540.10	2.40.110.10
4m9aA	4m9aA01_4m9aA02	1.10.540.10	2.40.110.10
2dvlA	2dvlA01_2dvlA02	1.10.540.10	2.40.110.10
3p26A	3p26A02_3p26A03	2.40.30.10	2.40.30.10
3nb2C	3nb2C03_3nb2C04	3.40.1850.10	1.10.4140.10
4e4tA	4e4tA01_4e4tA02	3.40.50.20	3.30.1490.20
1bxrC	1bxrC05_1bxrC06	3.30.470.20	3.40.50.1380
1bxrC	1bxrC04_1bxrC05	3.40.50.20	3.30.470.20
2ya0A	2ya0A01_2ya0A02	2.60.40.10	3.20.20.80
2ya0A	2ya0A02_2ya0A03	3.20.20.80	2.60.40.1180
3rimA	3rimA02_3rimA03	3.40.50.970	3.40.50.920
1vbkA	1vbkA01_1vbkA02	3.30.70.1510	3.30.2300.10
3sqrA	3sqrA01_3sqrA02	2.60.40.420	2.60.40.420
3sf6A	3sf6A01_3sf6A02	1.10.540.10	2.40.110.10
2wcoA	2wcoA01_2wcoA02	1.50.10.100	2.70.98.10
1t5hX	1t5hX02_1t5hX03	3.40.50.980	2.30.38.10
1h3fA	1h3fA01_1h3fA02	3.40.50.620	1.10.240.10
2piaA	2piaA01_2piaA02	2.40.30.10	3.40.50.80
1uzkA	1uzkA01_1uzkA02	2.10.25.10	3.90.290.10
1rrkA	1rrkA01_1rrkA02	3.40.50.410	2.40.10.10
4f5uA	4f5uA02_4f5uA03	1.10.246.10	1.10.246.10
4f5uA	4f5uA03_4f5uA04	1.10.246.10	1.10.246.10
1hh2P	1hh2P02_1hh2P03	2.40.50.140	3.30.300.20
2asbA	2asbA01_2asbA02	2.40.50.140	3.30.300.20
1hh2P	1hh2P03_1hh2P04	3.30.300.20	3.30.300.20
4agsA	4agsA03_4agsA04	3.40.30.10	1.20.1050.10
3b0xA	3b0xA01_3b0xA02	1.10.150.110	1.10.150.20
1u8vA	1u8vA02_1u8vA03	2.40.110.10	1.20.140.10
2y0cA	2y0cA02_2y0cA03	1.20.5.100	3.40.50.720
4f3lA	4f3lA01_4f3lA02	4.10.280.10	3.30.450.20
2c31A	2c31A02_2c31A03	3.40.50.1220	3.40.50.970
1bf2A	1bf2A01_1bf2A02	2.60.40.10	3.20.20.80
1bf2A	1bf2A02_1bf2A03	3.20.20.80	2.60.40.1180
2c42A	2c42A02_2c42A03	3.40.50.920	3.40.920.10
1opkA	1opkA03_1opkA04	3.30.200.20	1.10.510.10
3vmfA	3vmfA01_3vmfA02	3.40.50.300	2.40.30.10
2b78A	2b78A02_2b78A03	3.30.750.80	3.40.50.150
3vseA	3vseA02_3vseA03	3.30.750.80	3.40.50.150
1j72A	1j72A02_1j72A03	3.40.20.10	3.40.20.10
1np7B	1np7B02_1np7B03	1.25.40.80	1.10.579.10
4iv6A	4iv6A02_4iv6A03	2.40.110.10	1.20.140.10
3mw9A	3mw9A01_3mw9A02	1.10.287.140	3.40.50.10860
2c42A	2c42A01_2c42A02	3.40.50.970	3.40.50.920
4f3lB	4f3lB02_4f3lB03	3.30.450.20	3.30.450.20
3oojB	3oojB02_3oojB03	3.40.50.10490	3.40.50.10490
1f8nA	1f8nA02_1f8nA03	4.10.375.10	4.10.372.10

1x6vA	1x6vA02_1x6vA03	3.10.400.10	3.40.50.620
1krhA	1krhA02_1krhA03	2.40.30.10	3.40.50.80
2vtfA	2vtfA01_2vtfA02	3.20.20.80	2.60.120.260
3asiA	3asiA02_3asiA03	2.10.25.10	2.60.120.200
4nwgB	4nwgB02_4nwgB03	3.30.505.10	3.90.190.10
1g0dA	1g0dA03_1g0dA04	2.60.40.10	2.60.40.10
1t9bA	1t9bA02_1t9bA03	3.40.50.1220	3.40.50.970
2csuA	2csuA01_2csuA02	3.40.50.720	3.40.50.261
1w07A	1w07A03_1w07A04	1.20.140.10	1.20.140.10
1aozA	1aozA02_1aozA03	2.60.40.420	2.60.40.420
1oaoC	1oaoC02_1oaoC03	3.40.50.2030	3.30.1650.10
1oaoC	1oaoC01_1oaoC02	1.10.8.190	3.40.50.2030
1ex0A	1ex0A02_1ex0A03	3.90.260.10	2.60.40.10
1g0dA	1g0dA01_1g0dA02	2.60.40.10	3.90.260.10
1g0dA	1g0dA02_1g0dA03	3.90.260.10	2.60.40.10
1ex0A	1ex0A01_1ex0A02	2.60.40.10	3.90.260.10
1l9mA	1l9mA01_1l9mA02	2.60.40.10	3.90.260.10
1l9mA	1l9mA02_1l9mA03	3.90.260.10	2.60.40.10
1lxmA	1lxmA04_1lxmA02	2.60.40.1380	1.50.10.100
1lxmA	1lxmA02_1lxmA01	1.50.10.100	2.70.98.10
4ko8A	4ko8A01_4ko8A02	2.40.40.20	3.10.330.10
2qyuA	2qyuA02_2qyuA03	1.25.40.300	3.40.1850.10
2qyuA	2qyuA01_2qyuA02	2.160.20.80	1.25.40.300
4jklA	4jklA01_4jklA02	2.60.120.260	2.60.40.10
1vq8A	1vq8A01_1vq8A02	2.40.50.140	2.30.30.30
3zyzA	3zyzA01_3zyzA02	3.20.20.300	3.40.50.1700
2j8gA	2j8gA02_2j8gA03	2.10.270.10	2.20.120.10
1e3pA	1e3pA02_1e3pA03	1.10.10.400	3.30.230.70
3gcmA	3gcmA02_3gcmA03	1.10.10.400	3.30.230.70
1dofA	1dofA01_1dofA03	1.20.200.10	1.10.40.30
1dofA	1dofA02_1dofA01	1.10.275.10	1.20.200.10
2c12A	2c12A01_2c12A02	1.10.540.10	2.40.110.10
3cmgA	3cmgA03_3cmgA04	3.20.20.80	2.60.40.10
3cmgA	3cmgA02_3cmgA03	2.60.40.10	3.20.20.80
2hmaA	2hmaA01_2hmaA02	3.40.50.620	2.30.30.280
1iq0A	1iq0A01_1iq0A03	3.40.50.620	1.10.730.10
1furA	1furA01_1furA02	1.10.275.10	1.20.200.10
3ocfA	3ocfA02_3ocfA03	1.20.200.10	1.10.40.30
1furA	1furA02_1furA03	1.20.200.10	1.10.40.30
4admC	4admC02_4admC03	1.20.200.10	1.10.40.30
3r6qA	3r6qA02_3r6qA03	1.20.200.10	1.10.40.30
1kxpD	1kxpD01_1kxpD02	1.10.246.10	1.10.246.10
1kxpD	1kxpD02_1kxpD03	1.10.246.10	1.10.246.10
2x8xX	2x8xX02_2x8xX03	3.10.20.310	3.10.20.310
4i4tB	4i4tB01_4i4tB02	3.40.50.1440	3.30.1330.20
2wzkA	2wzkA01_2wzkA02	1.20.1310.10	1.20.1310.10

2v5cA	2v5cA02_2v5cA03	3.20.20.80	1.20.58.460
2chnA	2chnA01_2chnA02	3.30.379.10	3.20.20.80
2chnA	2chnA02_2chnA03	3.20.20.80	1.20.58.460
2v5cA	2v5cA01_2v5cA02	3.30.379.10	3.20.20.80
1kfuL	1kfuL03_1kfuL04	2.60.120.380	1.10.238.10
2w91A	2w91A01_2w91A02	3.20.20.80	2.60.120.260
2je8B	2je8B04_2je8B05	2.60.40.10	2.60.40.10
4f5uA	4f5uA01_4f5uA02	1.10.246.10	1.10.246.10
2c31A	2c31A01_2c31A02	3.40.50.970	3.40.50.1220
1mbmA	1mbmA02_1mbmA03	2.40.10.10	3.30.40.20
2jifA	2jifA02_2jifA03	2.40.110.10	1.20.140.10
3mpiA	3mpiA02_3mpiA03	2.40.110.10	1.20.140.10
3wdhA	3wdhA03_3wdhA04	3.20.20.80	2.60.40.1180
3wdhA	3wdhA02_3wdhA03	2.60.40.10	3.20.20.80
1dljA	1dljA01_1dljA03	3.40.50.720	1.10.1040.10
1n8yC	1n8yC01_1n8yC02	3.80.20.20	2.10.220.10
3edfA	3edfA02_3edfA03	3.20.20.80	2.60.40.1180
3edfA	3edfA01_3edfA02	2.60.40.10	3.20.20.80
3zh9B	3zh9B02_3zh9B03	1.10.8.60	1.20.272.10
1vkzA	1vkzA03_1vkzA04	3.30.470.20	3.90.600.10
3p7lA	3p7lA01_3p7lA02	3.30.830.10	3.30.830.10
2wqrA	2wqrA02_2wqrA03	2.60.40.10	2.60.40.10
4hr3A	4hr3A02_4hr3A03	2.40.110.10	1.20.140.10
4ba0A	4ba0A03_4ba0A04	2.60.40.1180	2.60.40.1180
1jz7A	1jz7A01_1jz7A02	2.60.120.260	2.60.40.10
1yq2A	1yq2A01_1yq2A02	2.60.120.260	2.60.40.10
3a21A	3a21A01_3a21A02	3.20.20.70	2.60.40.1180
3fl7A	3fl7A01_3fl7A02	2.60.120.260	2.60.40.1770
4iv6A	4iv6A01_4iv6A02	1.10.540.10	2.40.110.10
4m9aA	4m9aA02_4m9aA03	2.40.110.10	1.20.140.10
2dvlA	2dvlA02_2dvlA03	2.40.110.10	1.20.140.10
3owaA	3owaA03_3owaA04	1.20.140.10	1.20.140.10
2z1qB	2z1qB02_2z1qB03	2.40.110.10	1.20.140.10
2z1qB	2z1qB03_2z1qB04	1.20.140.10	1.20.140.10
2jbrA	2jbrA01_2jbrA02	1.10.540.10	2.40.110.10
2yweA	2yweA02_2yweA03	2.40.30.10	3.30.70.870
1qsaA	1qsaA02_1qsaA03	1.10.1240.20	1.10.530.10
2fokA	2fokA01_2fokA02	3.90.241.10	1.10.10.10
1k3iA	1k3iA02_1k3iA03	2.130.10.80	2.60.40.10
1k3iA	1k3iA01_1k3iA02	2.60.120.260	2.130.10.80
2vk8A	2vk8A02_2vk8A03	3.40.50.1220	3.40.50.970
2vbfA	2vbfA02_2vbfA03	3.40.50.1220	3.40.50.970
1biaA	1biaA01_1biaA02	1.10.10.10	3.30.930.10
1biaA	1biaA02_1biaA03	3.30.930.10	2.30.30.100
3uw2A	3uw2A01_3uw2A02	3.40.120.10	3.40.120.10
1ozhB	1ozhB01_1ozhB02	3.40.50.970	3.40.50.1220

2e6kA	2e6kA01_2e6kA02	3.40.50.970	3.40.50.970
2yweA	2yweA01_2yweA02	3.40.50.300	2.40.30.10
4psrA	4psrA01_4psrA02	3.20.20.80	2.60.40.1180
3fl7A	3fl7A03_3fl7A04	2.10.50.10	2.60.40.10
1gg4A	1gg4A02_1gg4A01	3.40.1190.10	3.90.190.20
1gg4A	1gg4A03_1gg4A02	3.40.1390.10	3.40.1190.10
1dd9A	1dd9A02_1dd9A03	3.40.1360.10	1.20.50.20
1dd9A	1dd9A01_1dd9A02	3.90.980.10	3.40.1360.10
2hr7A	2hr7A01_2hr7A02	3.80.20.20	2.10.220.10
1cs6A	1cs6A01_1cs6A02	2.60.40.10	2.60.40.10
2yykA	2yykA02_2yykA03	2.40.110.10	1.20.140.10
2csuA	2csuA02_2csuA03	3.40.50.261	3.40.50.261
1iq8A	1iq8A02_1iq8A03	3.90.1020.10	3.10.450.90
2re3A	2re3A01_2re3A02	3.10.540.10	2.30.270.10
4jekA	4jekA01_4jekA02	1.10.540.10	2.40.110.10
1mbmA	1mbmA01_1mbmA02	2.40.10.10	2.40.10.10
2w9mA	2w9mA02_2w9mA03	1.10.150.20	3.30.460.10
3hwcA	3hwcA02_3hwcA03	2.40.110.10	1.20.140.10
3uw2A	3uw2A02_3uw2A03	3.40.120.10	3.40.120.10
3uw2A	3uw2A03_3uw2A04	3.40.120.10	3.30.310.50
4i2xE	4i2xE02_4i2xE03	2.60.40.10	2.60.40.10
1q25A	1q25A02_1q25A03	2.70.130.10	2.70.130.10
1q25A	1q25A01_1q25A02	2.70.130.10	2.70.130.10
2v1xB	2v1xB02_2v1xB03	3.40.50.300	1.10.10.10
1oywA	1oywA02_1oywA03	3.40.50.300	1.10.10.10
1hm6A	1hm6A02_1hm6A03	1.10.220.10	1.10.220.10
4mduA	4mduA02_4mduA03	1.10.220.10	1.10.220.10
2wzkA	2wzkA02_2wzkA03	1.20.1310.10	1.20.1310.10
1h2vC	1h2vC01_1h2vC02	1.25.40.180	1.25.40.180
2vseA	2vseA04_2vseA05	2.80.10.50	2.80.10.50
2je8B	2je8B01_2je8B02	2.60.120.260	2.60.40.10
3n75A	3n75A03_3n75A04	3.90.1150.10	3.90.100.10
2as0A	2as0A02_2as0A03	3.30.750.80	3.40.50.150
1bg1A	1bg1A02_1bg1A03	2.60.40.630	1.10.238.10
2ahxA	2ahxA03_2ahxA04	3.80.20.20	2.10.220.10
1mdtA	1mdtA01_1mdtA02	3.90.175.10	1.10.490.40
4mamA	4mamA02_4mamA03	3.30.1490.20	3.30.470.20
3k5iA	3k5iA02_3k5iA03	3.30.1490.20	3.30.470.20
1cukA	1cukA01_1cukA02	2.40.50.140	1.10.150.20
1amuA	1amuA01_1amuA02	3.40.50.980	3.40.50.980
3lidA	3lidA01_3lidA02	1.20.5.170	3.30.450.20
3lidA	3lidA02_3lidA03	3.30.450.20	3.30.450.20
1zarA	1zarA01_1zarA02	1.10.10.10	3.30.200.20
2vycA	2vycA03_2vycA04	3.90.1150.10	3.90.100.10
4g1vA	4g1vA02_4g1vA03	2.40.30.10	3.40.50.80
3cmgA	3cmgA01_3cmgA02	2.60.120.260	2.60.40.10

3sxxA	3sxxA02_3sxxA03	3.10.450.40	2.70.98.20
2je8B	2je8B03_2je8B04	3.20.20.80	2.60.40.10
2je8B	2je8B02_2je8B03	2.60.40.10	3.20.20.80
2pz1A	2pz1A02_2pz1A03	1.20.900.10	2.30.29.30
2pz1A	2pz1A01_2pz1A02	2.30.30.40	1.20.900.10
3hi7A	3hi7A02_3hi7A03	3.10.450.40	2.70.98.20
2c10A	2c10A02_2c10A03	3.10.450.40	2.70.98.20
1pjqB	1pjqB03_1pjqB04	1.10.8.210	3.40.1010.10
1hc7A	1hc7A01_1hc7A02	3.30.930.10	3.40.50.800
2vzsA	2vzsA01_2vzsA02	2.60.120.260	2.60.40.10
2jbrA	2jbrA02_2jbrA03	2.40.110.10	1.20.140.10
1iq8A	1iq8A03_1iq8A04	3.10.450.90	2.30.130.10
4fl3A	4fl3A02_4fl3A03	1.10.930.10	3.30.505.10
4kmqA	4kmqA01_4kmqA02	2.60.40.10	2.60.40.1760
4ic6A	4ic6A02_4ic6A03	2.40.10.10	2.30.42.10
1y8tB	1y8tB02_1y8tB03	2.40.10.10	2.30.42.10
1sotB	1sotB01_1sotB02	2.40.10.10	2.40.10.10
1y8tB	1y8tB01_1y8tB02	2.40.10.10	2.40.10.10
4ic6A	4ic6A01_4ic6A02	2.40.10.10	2.40.10.10
4jkmA	4jkmA02_4jkmA03	2.60.40.10	3.20.20.80
4jklA	4jklA02_4jklA03	2.60.40.10	3.20.20.80
3lpfA	3lpfA02_3lpfA03	2.60.40.10	3.20.20.80
2bcqA	2bcqA03_2bcqA04	3.30.460.10	3.30.210.10
2bcqA	2bcqA02_2bcqA03	1.10.150.20	3.30.460.10
2f2hC	2f2hC02_2f2hC03	3.20.20.80	2.60.40.1180
2f2hC	2f2hC01_2f2hC02	2.60.40.1760	3.20.20.80
1l5hA	1l5hA03_1l5hA02	3.40.50.1980	3.40.50.1980
3m9vA	3m9vA01_3m9vA02	1.10.540.10	2.40.110.10
1aozA	1aozA01_1aozA02	2.60.40.420	2.60.40.420
2ebaA	2ebaA02_2ebaA03	2.40.110.10	1.20.140.10
1opkA	1opkA01_1opkA02	2.30.30.40	3.30.505.10
1k9aA	1k9aA02_1k9aA03	3.30.505.10	3.30.200.20
3bgaA	3bgaA03_3bgaA04	3.20.20.80	2.60.40.10
3bgaA	3bgaA02_3bgaA03	2.60.40.10	3.20.20.80
2or0A	2or0A02_2or0A03	2.40.110.10	1.20.140.10
2bm0A	2bm0A01_2bm0A02	3.40.50.300	2.40.30.10
1mv8A	1mv8A01_1mv8A02	3.40.50.720	1.20.5.170
1r6bX	1r6bX04_1r6bX05	3.40.50.300	1.10.8.60
3js8A	3js8A01_3js8A02	3.30.43.10	3.30.465.10
2vseA	2vseA02_2vseA03	2.80.10.50	2.80.10.50
2ztdA	2ztdA01_2ztdA02	2.40.50.140	1.10.150.20
3rimA	3rimA01_3rimA02	3.40.50.970	3.40.50.970
2o26U	2o26U01_2o26U02	2.60.40.10	2.60.40.10
1r2jA	1r2jA01_1r2jA02	1.10.540.10	2.40.110.10
2o3jA	2o3jA02_2o3jA03	1.20.5.100	3.40.50.720
3vmnA	3vmnA01_3vmnA02	2.60.40.10	3.20.20.80

3vmnA	3vmnA02_3vmnA03	3.20.20.80	2.60.40.1180
1xszA	1xszA02_1xszA03	1.10.1000.11	3.30.310.140
4aioA	4aioA01_4aioA02	2.60.40.1130	2.60.40.10
4c12A	4c12A01_4c12A02	3.40.1390.10	3.40.1190.10
4c12A	4c12A02_4c12A03	3.40.1190.10	3.90.190.20
1np7B	1np7B01_1np7B02	3.40.50.620	1.25.40.80
1z6tA	1z6tA01_1z6tA02	1.10.533.10	3.40.50.300
4o5mB	4o5mB01_4o5mB02	1.10.540.10	2.40.110.10
3utoA	3utoA03_3utoA04	1.10.510.10	2.60.40.10
3utoA	3utoA02_3utoA03	3.30.200.20	1.10.510.10
1ofeB	1ofeB02_1ofeB03	3.20.20.70	3.20.20.70
1ofeB	1ofeB03_1ofeB04	3.20.20.70	2.160.20.60
1yiiA	1yiiA02_1yiiA03	1.10.220.10	1.10.220.10
2nxwA	2nxwA01_2nxwA02	3.40.50.970	3.40.50.1220
2qenA	2qenA01_2qenA02	3.40.50.300	1.10.8.60
2r09A	2r09A02_2r09A03	1.10.1000.11	2.30.29.30
1ni2A	1ni2A01_1ni2A02	3.10.20.90	1.20.80.10
1ni2A	1ni2A02_1ni2A03	1.20.80.10	2.30.29.30
4ejnA	4ejnA02_4ejnA03	3.30.200.20	1.10.510.10
4jb1A	4jb1A01_4jb1A02	3.30.43.10	3.30.465.10
1x1iA	1x1iA01_1x1iA02	1.50.10.100	2.70.98.10
2vbfA	2vbfA01_2vbfA02	3.40.50.970	3.40.50.1220
2vk8A	2vk8A01_2vk8A02	3.40.50.970	3.40.50.1220
3l4yA	3l4yA04_3l4yA05	2.60.40.1180	2.60.40.1180
1djxA	1djxA02_1djxA03	3.20.20.190	2.60.40.150
1hn0A	1hn0A03_1hn0A04	2.70.98.10	2.60.220.10
2au3A	2au3A01_2au3A02	3.90.580.10	3.90.980.10
2au3A	2au3A02_2au3A03	3.90.980.10	3.40.1360.10
3au4A	3au4A03_3au4A04	1.20.80.10	2.30.29.30
3au4A	3au4A02_3au4A03	3.10.20.90	1.20.80.10
2yrxA	2yrxA03_2yrxA04	3.30.470.20	3.90.600.10
2c10A	2c10A01_2c10A02	3.10.450.40	3.10.450.40
1llaA	1llaA03_1llaA02	1.10.1280.10	2.60.40.1520
1llaA	1llaA01_1llaA03	1.20.1370.10	1.10.1280.10
1mkyA	1mkyA02_1mkyA03	3.40.50.300	3.30.300.20
4dcuA	4dcuA02_4dcuA03	3.40.50.300	3.30.300.20
1r0vA	1r0vA02_1r0vA03	3.40.1170.20	3.40.1350.10
4j7rA	4j7rA01_4j7rA02	2.60.40.10	3.20.20.80
4j7rA	4j7rA02_4j7rA03	3.20.20.80	2.60.40.1180
3utoA	3utoA01_3utoA02	2.60.40.10	3.30.200.20
1jz7A	1jz7A02_1jz7A03	2.60.40.10	3.20.20.80
1yq2A	1yq2A03_1yq2A04	3.20.20.80	2.60.40.10
1jz7A	1jz7A03_1jz7A04	3.20.20.80	2.60.40.10
1yq2A	1yq2A02_1yq2A03	2.60.40.10	3.20.20.80
4amwA	4amwA02_4amwA03	3.20.20.80	2.60.40.1180
4amwA	4amwA01_4amwA02	2.60.40.1760	3.20.20.80

1e8yA	1e8yA02_1e8yA03	2.60.40.150	1.25.40.70
1e8yA	1e8yA03_1e8yA04	1.25.40.70	3.30.1010.10
1kwgA	1kwgA01_1kwgA02	3.20.20.80	3.40.50.880
1r6bX	1r6bX02_1r6bX03	3.40.50.300	1.10.8.60
2y0cA	2y0cA01_2y0cA02	3.40.50.720	1.20.5.100
3p7lA	3p7lA02_3p7lA03	3.30.830.10	3.30.830.10
2qyuA	2qyuA03_2qyuA04	3.40.1850.10	1.10.4140.10
1okgA	1okgA02_1okgA03	3.40.250.10	3.30.1670.10
1ep3B	1ep3B01_1ep3B02	2.40.30.10	3.40.50.80
3weoA	3weoA03_3weoA04	2.60.40.1180	2.60.40.1180
1n00A	1n00A02_1n00A04	1.10.220.10	1.10.220.10
4k9qA	4k9qA02_4k9qA03	3.40.50.1220	3.40.50.970
1wxxA	1wxxA01_1wxxA02	2.30.130.10	3.30.750.80
1nkgA	1nkgA01_1nkgA02	2.70.98.10	2.60.40.1120
2odpA	2odpA01_2odpA02	3.40.50.410	2.40.10.10
4a4aA	4a4aA03_4a4aA04	3.20.20.80	1.20.120.670
4a4aA	4a4aA02_4a4aA03	3.30.379.10	3.20.20.80
3zx1A	3zx1A01_3zx1A02	2.60.40.420	2.60.40.420
3zx1A	3zx1A02_3zx1A03	2.60.40.420	2.60.40.420
1lvaA	1lvaA01_1lvaA02	1.10.10.10	1.10.10.10
2vzsA	2vzsA02_2vzsA03	2.60.40.10	3.20.20.80
2vzsA	2vzsA03_2vzsA04	3.20.20.80	2.60.40.10
3lafA	3lafA02_3lafA03	2.60.40.10	2.60.40.10
1cb8A	1cb8A02_1cb8A03	2.70.98.10	2.60.220.10
2zxqA	2zxqA01_2zxqA02	2.70.98.10	3.20.20.80
2zxqA	2zxqA02_2zxqA03	3.20.20.80	2.60.40.1180
3mpiA	3mpiA01_3mpiA02	1.10.540.10	2.40.110.10
1yq2A	1yq2A04_1yq2A05	2.60.40.10	2.70.98.10
3ziaD	3ziaD01_3ziaD02	2.40.10.170	3.40.50.300
3ziaD	3ziaD02_3ziaD03	3.40.50.300	1.10.1140.10
1ba3A	1ba3A02_1ba3A03	3.40.50.980	2.30.38.10
1lmlA	1lmlA03_1lmlA04	2.10.55.10	2.30.34.10
2o3jA	2o3jA01_2o3jA02	3.40.50.720	1.20.5.100
1gntA	1gntA01_1gntA02	1.20.1270.20	1.20.1270.20
2ddhA	2ddhA01_2ddhA02	1.10.540.10	2.40.110.10
1w07A	1w07A01_1w07A02	1.10.540.10	2.40.110.10
4tr7A	4tr7A02_4tr7A03	3.10.150.10	3.10.150.10
2z1qB	2z1qB01_2z1qB02	1.10.540.10	2.40.110.10
1pixA	1pixA02_1pixA03	3.90.226.10	3.90.226.10
1npeB	1npeB02_1npeB03	2.10.25.10	2.10.25.10
1npeB	1npeB01_1npeB02	2.10.25.10	2.10.25.10
2bzdB	2bzdB02_2bzdB03	2.60.40.10	2.60.120.260
4o5mB	4o5mB02_4o5mB03	2.40.110.10	1.20.140.10
1e6yA	1e6yA02_1e6yA03	3.30.70.470	1.20.840.10
2qtcA	2qtcA01_2qtcA02	3.40.50.970	3.40.50.970
3llpA	3llpA01_3llpA02	2.80.10.50	2.80.10.50

1c7sA	1c7sA02_1c7sA03	3.30.379.10	3.20.20.80
1c7sA	1c7sA03_1c7sA04	3.20.20.80	2.60.40.10
4aioA	4aioA02_4aioA03	2.60.40.10	3.20.20.80
4aioA	4aioA03_4aioA04	3.20.20.80	2.60.40.1180
1k9fA	1k9fA02_1k9fA03	3.20.20.80	3.90.1330.10
1k9fA	1k9fA01_1k9fA02	3.30.379.10	3.20.20.80
1gqiA	1gqiA02_1gqiA03	3.20.20.80	3.90.1330.10
1gqiA	1gqiA01_1gqiA02	3.30.379.10	3.20.20.80
1v4aA	1v4aA02_1v4aA03	3.30.460.10	1.20.120.330
1v4aA	1v4aA01_1v4aA02	1.10.4050.10	3.30.460.10
3sqrA	3sqrA02_3sqrA03	2.60.40.420	2.60.40.420
1vpkA	1vpkA02_1vpkA03	3.10.150.10	3.10.150.10
1vpkA	1vpkA01_1vpkA02	3.10.150.10	3.10.150.10
4ba0A	4ba0A02_4ba0A03	3.20.20.80	2.60.40.1180
4ba0A	4ba0A01_4ba0A02	2.60.40.1760	3.20.20.80
1td6A	1td6A01_1td6A02	1.20.1480.10	3.30.1790.10
3pfdA	3pfdA01_3pfdA02	1.10.540.10	2.40.110.10
2uxtA	2uxtA02_2uxtA03	2.60.40.420	2.60.40.420
2uxtA	2uxtA01_2uxtA02	2.60.40.420	2.60.40.420
1k32A	1k32A04_1k32A05	2.30.42.10	3.90.226.10
2yykA	2yykA01_2yykA02	1.10.3140.10	2.40.110.10
2nxwA	2nxwA02_2nxwA03	3.40.50.1220	3.40.50.970
1oaoC	1oaoC03_1oaoC04	3.30.1650.10	3.40.970.20
4dmgA	4dmgA01_4dmgA02	2.30.130.10	3.30.750.80
3c0kA	3c0kA01_3c0kA02	2.30.130.10	3.30.750.80
1sr8A	1sr8A01_1sr8A02	3.30.1990.10	3.30.2110.10
3a24A	3a24A02_3a24A03	3.20.20.70	2.60.40.1180
3a24A	3a24A01_3a24A02	2.70.98.10	3.20.20.70
1hfuA	1hfuA01_1hfuA02	2.60.40.420	2.60.40.420
1lmlA	1lmlA02_1lmlA03	3.90.132.10	2.10.55.10
1pjqB	1pjqB04_1pjqB05	3.40.1010.10	3.30.950.10
3c0kA	3c0kA02_3c0kA03	3.30.750.80	3.40.50.150
1u4qA	1u4qA02_1u4qA03	1.20.58.60	1.20.58.60
2b78A	2b78A01_2b78A02	2.30.130.10	3.30.750.80
1rx0A	1rx0A02_1rx0A03	2.40.110.10	1.20.140.10
1fx7A	1fx7A02_1fx7A03	1.10.60.10	2.30.30.90
1q5nA	1q5nA01_1q5nA02	1.10.275.10	1.20.200.10
1q5nA	1q5nA02_1q5nA03	1.20.200.10	1.10.40.30
2gksA	2gksA01_2gksA02	3.10.400.10	3.40.50.620
1g8fA	1g8fA02_1g8fA01	3.10.400.10	3.40.50.620
1td6A	1td6A02_1td6A03	3.30.1790.10	1.10.472.40
4f3lA	4f3lA02_4f3lA03	3.30.450.20	3.30.450.20
3affA	3affA02_3affA03	2.40.110.10	1.20.140.10
2bzdB	2bzdB01_2bzdB02	2.120.10.10	2.60.40.10
3a9sA	3a9sA02_3a9sA03	3.40.275.10	3.20.14.10
4e9sA	4e9sA02_4e9sA03	2.60.40.420	2.60.40.420

1ehiA	1ehiA03_1ehiA02	3.30.1490.20	3.30.470.20
2q9oA	2q9oA01_2q9oA02	2.60.40.420	2.60.40.420
3q9oA	3q9oA02_3q9oA03	3.90.1350.10	3.90.175.10
1ikpA	1ikpA02_1ikpA03	3.90.1350.10	3.90.175.10
4ghgA	4ghgA01_4ghgA02	3.10.180.10	3.10.180.10
1kxpD	1kxpD03_1kxpD04	1.10.246.10	1.10.246.10
2he7A	2he7A01_2he7A02	3.10.20.90	1.20.80.10
2he7A	2he7A02_2he7A03	1.20.80.10	2.30.29.30
1mdbA	1mdbA02_1mdbA03	3.40.50.980	2.30.38.10
1mdbA	1mdbA01_1mdbA02	3.40.50.980	3.40.50.980
2btoA	2btoA02_2btoA03	3.30.1330.20	1.10.287.600
2wsdA	2wsdA02_2wsdA03	2.60.40.420	2.60.40.420
2wsdA	2wsdA01_2wsdA02	2.60.40.420	2.60.40.420
3k5iA	3k5iA01_3k5iA02	3.40.50.20	3.30.1490.20
1r5bA	1r5bA02_1r5bA03	2.40.30.10	2.40.30.10
2d73A	2d73A02_2d73A03	3.20.20.70	2.60.40.1180
2d73A	2d73A01_2d73A02	2.70.98.10	3.20.20.70
4hr3A	4hr3A01_4hr3A02	1.10.540.10	2.40.110.10
2as0A	2as0A01_2as0A02	2.30.130.10	3.30.750.80
2odpA	2odpA02_2odpA03	2.40.10.10	2.40.10.10
1rrkA	1rrkA02_1rrkA03	2.40.10.10	2.40.10.10
1oaoA	1oaoA03_1oaoA02	1.20.1270.30	3.40.50.2030
3c18C	3c18C01_3c18C02	3.30.460.10	1.20.120.330
3c18C	3c18C02_3c18C03	1.20.120.330	1.10.10.10
1kfiA	1kfiA02_1kfiA03	3.40.120.10	3.40.120.10
3nvwK	3nvwK01_3nvwK02	3.30.43.10	3.30.465.10
3nvwK	3nvwK02_3nvwK03	3.30.465.10	3.30.390.50
2o7sA	2o7sA02_2o7sA03	3.40.50.10860	3.40.50.720
1f00I	1f00I02_1f00I03	2.60.40.1080	3.10.100.10
1vl2A	1vl2A01_1vl2A02	3.40.50.620	3.90.1260.10
4jekA	4jekA02_4jekA03	2.40.110.10	1.20.140.10
1f8nA	1f8nA01_1f8nA02	2.60.60.20	4.10.375.10
1mdtA	1mdtA02_1mdtA03	1.10.490.40	2.60.40.700
3bgaA	3bgaA01_3bgaA02	2.60.120.260	2.60.40.10
1pbyA	1pbyA03_1pbyA04	2.60.40.10	2.60.40.10
1jmxA	1jmxA03_1jmxA04	2.60.40.10	2.60.40.10
1oacA	1oacA04_1oacA03	3.10.450.40	2.70.98.20
3rh4A	3rh4A01_3rh4A02	1.10.150.110	1.10.150.20
3llpA	3llpA03_3llpA04	2.80.10.50	2.80.10.50
3llpA	3llpA02_3llpA03	2.80.10.50	2.80.10.50
2btoA	2btoA01_2btoA02	3.40.50.1440	3.30.1330.20
2ihtA	2ihtA02_2ihtA03	3.40.50.1220	3.40.50.970
1c97A	1c97A01_1c97A02	3.30.499.10	3.40.1060.10
1u8vA	1u8vA01_1u8vA02	1.10.3140.10	2.40.110.10
3eyaA	3eyaA02_3eyaA03	3.40.50.1220	3.40.50.970
4evfA	4evfA02_4evfA03	1.10.220.10	1.10.220.10

1cwvA	1cwvA04_1cwvA05	2.60.40.1080	3.10.100.10
2f00A	2f00A01_2f00A02	3.40.50.720	3.40.1190.10
2f00A	2f00A02_2f00A03	3.40.1190.10	3.90.190.20
3n75A	3n75A02_3n75A03	3.40.640.10	3.90.1150.10
2vycA	2vycA02_2vycA03	3.40.640.10	3.90.1150.10
3zniA	3zniA02_3zniA03	1.10.238.10	3.30.505.10
1exmA	1exmA02_1exmA03	2.40.30.10	2.40.30.10
4kmqA	4kmqA05_4kmqA06	2.60.40.1180	2.60.120.260
4kmqA	4kmqA04_4kmqA05	2.60.40.1180	2.60.40.1180
1cqxA	1cqxA02_1cqxA03	2.40.30.10	3.40.50.80
1mpgA	1mpgA01_1mpgA02	3.30.310.20	1.10.340.30
1mpgA	1mpgA02_1mpgA03	1.10.340.30	1.10.1670.10
1bucA	1bucA01_1bucA02	1.10.540.10	2.40.110.10
3weoA	3weoA02_3weoA03	3.20.20.80	2.60.40.1180
3l4yA	3l4yA03_3l4yA04	3.20.20.80	2.60.40.1180
3weoA	3weoA01_3weoA02	2.60.40.1760	3.20.20.80
3l4yA	3l4yA02_3l4yA03	2.60.40.1760	3.20.20.80
1hskA	1hskA02_1hskA01	3.30.43.10	3.30.465.10
1kj8A	1kj8A01_1kj8A02	3.40.50.20	3.30.1490.20
2dwcA	2dwcA01_2dwcA02	3.40.50.20	3.30.1490.20
3ialA	3ialA01_3ialA02	3.30.930.10	3.40.50.800
1okgA	1okgA01_1okgA02	3.40.250.10	3.40.250.10
2wcoA	2wcoA02_2wcoA03	2.70.98.10	2.60.220.10
1x1iA	1x1iA02_1x1iA03	2.70.98.10	2.60.220.10
2fd6U	2fd6U02_2fd6U03	2.10.60.10	2.10.60.10
2fd6U	2fd6U01_2fd6U02	2.10.60.10	2.10.60.10
3zm6A	3zm6A02_3zm6A03	3.40.1190.10	3.90.190.20
3zm6A	3zm6A01_3zm6A02	3.40.1390.10	3.40.1190.10
3i99A	3i99A02_3i99A03	3.30.465.10	3.90.78.10
3i99A	3i99A01_3i99A02	3.30.43.10	3.30.465.10
1uxyA	1uxyA02_1uxyA03	3.30.43.10	3.30.465.10
4c5iA	4c5iA03_4c5iA04	2.30.30.140	2.30.30.140
4c5iA	4c5iA02_4c5iA03	2.30.30.140	2.30.30.140
4kxvA	4kxvA01_4kxvA02	3.40.50.970	3.40.50.970
2zygA	2zygA01_2zygA02	3.40.50.720	1.10.1040.10
1exmA	1exmA01_1exmA02	3.40.50.300	2.40.30.10
1rwhA	1rwhA02_1rwhA03	2.70.98.10	2.60.220.10
2fh8A	2fh8A01_2fh8A02	2.60.40.1130	2.60.40.10
2re3A	2re3A02_2re3A03	2.30.270.10	2.20.70.10
2fuvA	2fuvA03_2fuvA04	3.40.120.10	3.30.310.50
2fuvA	2fuvA02_2fuvA03	3.40.120.10	3.40.120.10
1ksiA	1ksiA02_1ksiA03	3.10.450.40	2.70.98.20
3oyyA	3oyyA02_3oyyA03	2.40.50.140	2.40.50.140
2v5mA	2v5mA02_2v5mA03	2.60.40.10	2.60.40.10
1lshA	1lshA01_1lshA02	2.30.230.10	1.25.10.20
1lshA	1lshA02_1lshA03	1.25.10.20	2.20.50.20

1wvfA	1wvfA02_1wvfA03	3.30.465.10	3.40.462.10
1e8yA	1e8yA04_1e8yA05	3.30.1010.10	1.10.1070.11
1lvaA	1lvaA03_1lvaA04	1.10.10.10	1.10.10.10
4im0A	4im0A01_4im0A02	3.30.200.20	1.10.510.10
1kfuL	1kfuL02_1kfuL03	3.90.70.10	2.60.120.380
3vrpA	3vrpA01_3vrpA02	1.20.930.20	1.10.238.10
1r5bA	1r5bA01_1r5bA02	3.40.50.300	2.40.30.10
2ebaA	2ebaA01_2ebaA02	1.10.540.10	2.40.110.10
1c97A	1c97A03_1c97A04	3.30.499.10	3.20.19.10
1f8nA	1f8nA04_1f8nA05	3.10.450.60	1.20.245.10
1kwgA	1kwgA02_1kwgA03	3.40.50.880	2.60.40.1180
2vzsA	2vzsA04_2vzsA05	2.60.40.10	2.60.40.10
1oaoA	1oaoA02_1oaoA01	3.40.50.2030	3.40.50.2030
1uebA	1uebA02_1uebA03	2.40.50.140	2.40.50.140
2qn6A	2qn6A02_2qn6A03	2.40.30.10	2.40.30.10
1ji1A	1ji1A01_1ji1A02	2.60.40.10	3.20.20.80
1ji1A	1ji1A02_1ji1A03	3.20.20.80	2.60.40.1180
2qqoA	2qqoA02_2qqoA03	2.60.120.260	2.60.120.260
2qqoA	2qqoA01_2qqoA02	2.60.120.290	2.60.120.260
3is0X	3is0X01_3is0X02	2.60.40.1280	2.60.40.1290
3wa2X	3wa2X02_3wa2X03	3.10.450.40	2.70.98.20
1gntA	1gntA02_1gntA03	1.20.1270.20	3.40.50.2030
4f7kA	4f7kA01_4f7kA02	2.60.40.420	2.60.40.420
4f7kA	4f7kA02_4f7kA03	2.60.40.420	2.60.40.420
1vclA	1vclA02_1vclA03	2.80.10.50	3.30.1750.10
3l5iA	3l5iA01_3l5iA02	2.60.40.10	2.60.40.10
2fh1A	2fh1A01_2fh1A02	3.40.20.10	3.40.20.10
2hoxA	2hoxA02_2hoxA03	3.40.640.10	3.90.1150.10
2hoxA	2hoxA01_2hoxA02	2.10.25.30	3.40.640.10
3q9oA	3q9oA01_3q9oA02	2.60.120.200	3.90.1350.10
1yt8A	1yt8A02_1yt8A03	3.40.250.10	3.40.250.10
3uomA	3uomA02_3uomA03	3.40.30.10	3.40.30.10
2pg0A	2pg0A01_2pg0A02	1.10.540.10	2.40.110.10
3r7kA	3r7kA01_3r7kA02	1.10.540.10	2.40.110.10
2d29A	2d29A01_2d29A02	1.10.540.10	2.40.110.10
1jqiA	1jqiA01_1jqiA02	1.10.540.10	2.40.110.10
2jifA	2jifA01_2jifA02	1.10.540.10	2.40.110.10
1egdA	1egdA01_1egdA02	1.10.540.10	2.40.110.10
2zueA	2zueA02_2zueA03	3.40.50.620	1.10.730.10
2qenA	2qenA02_2qenA03	1.10.8.60	1.10.10.10
2fnaA	2fnaA02_2fnaA03	1.10.8.60	1.10.10.10
1v5eA	1v5eA02_1v5eA03	3.40.50.1220	3.40.50.970
1n8yC	1n8yC03_1n8yC04	3.80.20.20	2.10.220.10
1n8yC	1n8yC02_1n8yC03	2.10.220.10	3.80.20.20
1cvrA	1cvrA01_1cvrA02	3.40.50.10390	3.40.50.1460
1cvrA	1cvrA02_1cvrA03	3.40.50.1460	2.60.40.10

3nb2C	3nb2C02_3nb2C03	1.25.40.300	3.40.1850.10
4kmqA	4kmqA02_4kmqA03	2.60.40.1760	3.20.20.80
4kmqA	4kmqA03_4kmqA04	3.20.20.80	2.60.40.1180
4aefA	4aefA01_4aefA02	2.60.40.10	2.60.40.10
3lp8A	3lp8A03_3lp8A04	3.30.470.20	3.90.600.10
3dzdA	3dzdA02_3dzdA03	3.40.50.300	1.10.8.60
2vtfA	2vtfA02_2vtfA03	2.60.120.260	2.60.40.10
3vseA	3vseA01_3vseA02	2.30.130.10	3.30.750.80
1wvfA	1wvfA01_1wvfA02	3.30.43.10	3.30.465.10
4g1vA	4g1vA01_4g1vA02	1.10.490.10	2.40.30.10
2v1xB	2v1xB01_2v1xB02	3.40.50.300	3.40.50.300
4mb4A	4mb4A01_4mb4A02	3.20.20.80	2.60.40.10
3uomA	3uomA01_3uomA02	3.40.30.10	3.40.30.10
2gnoA	2gnoA02_2gnoA03	1.10.8.250	1.20.272.10
1m7xA	1m7xA01_1m7xA02	2.60.40.10	3.20.20.80
1m7xA	1m7xA02_1m7xA03	3.20.20.80	2.60.40.1180
3aw5A	3aw5A02_3aw5A03	2.60.40.420	2.60.40.420
3aw5A	3aw5A01_3aw5A02	2.60.40.420	2.60.40.420
3tewA	3tewA02_3tewA03	2.60.120.240	3.10.20.110
1cb8A	1cb8A01_1cb8A02	1.50.10.100	2.70.98.10
1zarA	1zarA02_1zarA03	3.30.200.20	1.10.510.10
1fx7A	1fx7A01_1fx7A02	1.10.10.10	1.10.60.10
3tewA	3tewA01_3tewA02	3.90.182.10	2.60.120.240
3l4yA	3l4yA01_3l4yA02	4.10.110.10	2.60.40.1760
2p67A	2p67A02_2p67A03	3.40.50.300	1.10.287.130
2qm8A	2qm8A02_2qm8A03	3.40.50.300	1.10.287.130
3nxsA	3nxsA02_3nxsA03	3.40.50.300	1.10.287.130
1dljA	1dljA03_1dljA02	1.10.1040.10	3.40.50.720
2dy1A	2dy1A01_2dy1A02	3.40.50.300	2.40.30.10
4cvuA	4cvuA03_4cvuA04	3.20.20.80	2.60.40.10
4cvuA	4cvuA02_4cvuA03	2.60.40.10	3.20.20.80
2yevA	2yevA01_2yevA02	1.20.210.10	1.10.287.70
2uz1A	2uz1A01_2uz1A02	3.40.50.970	3.40.50.1220
3p26A	3p26A01_3p26A02	3.40.50.300	2.40.30.10
2ywcC	2ywcC02_2ywcC03	3.40.50.620	3.30.300.10
1tuoA	1tuoA03_1tuoA04	3.40.120.10	3.30.310.50
1tuoA	1tuoA02_1tuoA03	3.40.120.10	3.40.120.10
4ghgA	4ghgA02_4ghgA03	3.10.180.10	4.10.1270.10
1c3cA	1c3cA01_1c3cA02	1.10.275.10	1.20.200.10
1c3cA	1c3cA02_1c3cA03	1.20.200.10	1.10.40.30
3zniA	3zniA01_3zniA02	1.20.930.20	1.10.238.10
3abqA	3abqA01_3abqA02	2.30.170.30	1.10.220.70
5reqB	5reqB01_5reqB02	3.20.20.240	3.40.50.280
3dfgA	3dfgA02_3dfgA03	1.10.10.10	1.10.10.10
1jb7A	1jb7A01_1jb7A02	2.40.50.140	2.40.50.140
4kxvA	4kxvA02_4kxvA03	3.40.50.970	3.40.50.920

4agsA	4agsA02_4agsA03	1.20.1050.10	3.40.30.10
4agsA	4agsA01_4agsA02	3.40.30.10	1.20.1050.10
3f1sB	3f1sB02_3f1sB03	2.40.10.10	2.40.10.10
1ikpA	1ikpA01_1ikpA02	2.60.120.200	3.90.1350.10
3mw9A	3mw9A02_3mw9A03	3.40.50.10860	3.40.50.720
1qsaA	1qsaA01_1qsaA02	1.25.20.10	1.10.1240.20
3vmfA	3vmfA02_3vmfA03	2.40.30.10	2.40.30.10
1bg1A	1bg1A03_1bg1A04	1.10.238.10	3.30.505.10
1rx0A	1rx0A01_1rx0A02	1.10.540.10	2.40.110.10
3apoA	3apoA04_3apoA05	3.40.30.10	3.40.30.10
4h04A	4h04A01_4h04A02	3.30.379.10	3.20.20.80
4h04A	4h04A02_4h04A03	3.20.20.80	2.80.10.50
1d0nA	1d0nA01_1d0nA02	3.40.20.10	3.40.20.10
2chnA	2chnA03_2chnA04	1.20.58.460	2.60.40.1180
3abgA	3abgA02_3abgA03	2.60.40.420	2.60.40.420
3abgA	3abgA01_3abgA02	2.60.40.420	2.60.40.420
2q07A	2q07A02_2q07A03	3.10.450.90	2.30.130.10
1r2jA	1r2jA02_1r2jA03	2.40.110.10	1.20.140.10
3uemA	3uemA02_3uemA03	3.40.30.10	3.40.30.10
1q6zA	1q6zA02_1q6zA03	3.40.50.1220	3.40.50.970
1oaoC	1oaoC04_1oaoC05	3.40.970.20	3.40.1470.10
2be4A	2be4A01_2be4A02	1.10.238.10	1.10.238.10
2be4A	2be4A02_2be4A03	1.10.238.10	1.10.238.10
3ziyA	3ziyA02_3ziyA03	2.60.40.420	1.10.760.10
3ziyA	3ziyA01_3ziyA02	2.60.40.420	2.60.40.420
3c6kA	3c6kA02_3c6kA03	2.30.140.10	3.40.50.150

1046 consecutive/non-consecutive domain dataset (domain-CU-M)			
**PDB ID**	**Domain pair**	**Domain1 CATH classification**	**Domain 2 CATH classification**
1a5tA	1a5tA01_1a5tA02	3.40.50.300	1.10.8.10
1a5tA	1a5tA02_1a5tA03	1.10.8.10	1.20.272.10
1amuA	1amuA01_1amuA02	3.40.50.980	3.40.50.980
1amuA	1amuA01_1amuA03	3.40.50.980	2.30.38.10
1amuA	1amuA02_1amuA03	3.40.50.980	2.30.38.10
1anxB	1anxB01_1anxB04	1.10.220.10	1.10.220.10
1anxB	1anxB02_1anxB04	1.10.220.10	1.10.220.10
1anxB	1anxB03_1anxB02	1.10.220.10	1.10.220.10
1aozA	1aozA02_1aozA01	2.60.40.420	2.60.40.420
1aozA	1aozA03_1aozA01	2.60.40.420	2.60.40.420
1aozA	1aozA03_1aozA02	2.60.40.420	2.60.40.420
1axnA	1axnA01_1axnA04	1.10.220.10	1.10.220.10
1ba3A	1ba3A01_1ba3A02	3.40.50.980	3.40.50.980
1ba3A	1ba3A01_1ba3A05	3.40.50.980	4.10.8.10
1ba3A	1ba3A02_1ba3A03	3.40.50.980	2.30.38.10
1ba3A	1ba3A02_1ba3A05	3.40.50.980	4.10.8.10
1ba3A	1ba3A03_1ba3A05	2.30.38.10	4.10.8.10
1bf2A	1bf2A02_1bf2A01	3.20.20.80	2.60.40.10
1bf2A	1bf2A02_1bf2A03	3.20.20.80	2.60.40.1180
1bg1A	1bg1A01_1bg1A02	1.20.1050.20	2.60.40.630
1bg1A	1bg1A02_1bg1A03	2.60.40.630	1.10.238.10
1bg1A	1bg1A03_1bg1A04	1.10.238.10	3.30.505.10
1biaA	1biaA02_1biaA01	3.30.930.10	1.10.10.10
1biaA	1biaA02_1biaA03	3.30.930.10	2.30.30.100
1bxrC	1bxrC02_1bxrC01	3.30.470.20	3.40.50.20
1bxrC	1bxrC02_1bxrC03	3.30.470.20	1.10.1030.10
1bxrC	1bxrC02_1bxrC05	3.30.470.20	3.30.470.20
1bxrC	1bxrC05_1bxrC01	3.30.470.20	3.40.50.20
1bxrC	1bxrC05_1bxrC06	3.30.470.20	3.40.50.1380
1c7sA	1c7sA03_1c7sA01	3.20.20.80	2.60.40.290
1c7sA	1c7sA03_1c7sA02	3.20.20.80	3.30.379.10
1c7sA	1c7sA03_1c7sA04	3.20.20.80	2.60.40.10
1c97A	1c97A01_1c97A02	3.30.499.10	3.40.1060.10
1c97A	1c97A01_1c97A03	3.30.499.10	3.30.499.10
1c97A	1c97A03_1c97A02	3.30.499.10	3.40.1060.10
1c97A	1c97A04_1c97A01	3.20.19.10	3.30.499.10
1c97A	1c97A04_1c97A03	3.20.19.10	3.30.499.10
1cb8A	1cb8A01_1cb8A02	1.50.10.100	2.70.98.10
1cb8A	1cb8A02_1cb8A03	2.70.98.10	2.60.220.10
1cqxA	1cqxA01_1cqxA02	1.10.490.10	2.40.30.10
1cqxA	1cqxA03_1cqxA02	3.40.50.80	2.40.30.10
1cs6A	1cs6A01_1cs6A02	2.60.40.10	2.60.40.10
1cs6A	1cs6A03_1cs6A04	2.60.40.10	2.60.40.10
1cukA	1cukA02_1cukA01	1.10.150.20	2.40.50.140
1cvrA	1cvrA01_1cvrA03	3.40.50.10390	2.60.40.10
1cvrA	1cvrA02_1cvrA01	3.40.50.1460	3.40.50.10390
1cvrA	1cvrA02_1cvrA03	3.40.50.1460	2.60.40.10
1cwvA	1cwvA05_1cwvA04	3.10.100.10	2.60.40.1080
1cygA	1cygA01_1cygA02	3.20.20.80	2.60.40.1180
1cygA	1cygA01_1cygA03	3.20.20.80	2.60.40.10

1cygA	1cygA01_1cygA04	3.20.20.80	2.60.40.10
1d0nA	1d0nA01_1d0nA02	3.40.20.10	3.40.20.10
1d0nA	1d0nA01_1d0nA03	3.40.20.10	3.40.20.10
1d0nA	1d0nA04_1d0nA05	3.40.20.10	3.40.20.10
1d0nA	1d0nA04_1d0nA06	3.40.20.10	3.40.20.10
1d0nA	1d0nA06_1d0nA02	3.40.20.10	3.40.20.10
1d0nA	1d0nA06_1d0nA03	3.40.20.10	3.40.20.10
1dd9A	1dd9A02_1dd9A01	3.40.1360.10	3.90.980.10
1dd9A	1dd9A02_1dd9A03	3.40.1360.10	1.20.50.20
1djxA	1djxA02_1djxA03	3.20.20.190	2.60.40.150
1djxA	1djxA03_1djxA01	2.60.40.150	1.10.238.10
1dljA	1dljA01_1dljA03	3.40.50.720	1.10.1040.10
1dljA	1dljA02_1dljA03	3.40.50.720	1.10.1040.10
1dm5A	1dm5A03_1dm5A02	1.10.220.10	1.10.220.10
1dofB	1dofB01_1dofB02	1.20.200.10	1.10.275.10
1dofB	1dofB01_1dofB03	1.20.200.10	1.10.40.30
1du3G	1du3G01_1du3G03	2.10.50.10	2.10.50.10
1e3pA	1e3pA01_1e3pA03	3.30.230.70	3.30.230.70
1e3pA	1e3pA03_1e3pA02	3.30.230.70	1.10.10.400
1e6yA	1e6yA03_1e6yA02	1.20.840.10	3.30.70.470
1e7uA	1e7uA01_1e7uA04	3.10.20.90	3.30.1010.10
1e7uA	1e7uA03_1e7uA01	1.25.40.70	3.10.20.90
1e7uA	1e7uA03_1e7uA02	1.25.40.70	2.60.40.150
1e7uA	1e7uA03_1e7uA04	1.25.40.70	3.30.1010.10
1e7uA	1e7uA05_1e7uA03	1.10.1070.11	1.25.40.70
1e8cA	1e8cA02_1e8cA01	3.40.1190.10	3.40.1390.10
1e8cA	1e8cA02_1e8cA03	3.40.1190.10	3.90.190.20
1egdA	1egdA02_1egdA01	2.40.110.10	1.10.540.10
1egdA	1egdA03_1egdA01	1.20.140.10	1.10.540.10
1egdA	1egdA03_1egdA02	1.20.140.10	2.40.110.10
1ehiA	1ehiA02_1ehiA01	3.30.470.20	3.40.50.20
1ehiA	1ehiA02_1ehiA03	3.30.470.20	3.30.1490.20
1ei5A	1ei5A01_1ei5A03	3.40.710.10	2.40.128.50
1ei5A	1ei5A03_1ei5A02	2.40.128.50	2.40.128.50
1ep3B	1ep3B02_1ep3B01	3.40.50.80	2.40.30.10
1ex0A	1ex0A02_1ex0A01	3.90.260.10	2.60.40.10
1ex0A	1ex0A02_1ex0A03	3.90.260.10	2.60.40.10
1ex0A	1ex0A02_1ex0A04	3.90.260.10	2.60.40.10
1exmA	1exmA01_1exmA02	3.40.50.300	2.40.30.10
1exmA	1exmA01_1exmA03	3.40.50.300	2.40.30.10
1exmA	1exmA02_1exmA03	2.40.30.10	2.40.30.10
1f00I	1f00I03_1f00I02	3.10.100.10	2.60.40.1080
1f0lA	1f0lA01_1f0lA02	3.90.175.10	1.10.490.40
1f0lA	1f0lA01_1f0lA03	3.90.175.10	2.60.40.700
1f0lA	1f0lA02_1f0lA03	1.10.490.40	2.60.40.700
1f7uA	1f7uA01_1f7uA02	3.40.50.620	1.10.730.10
1f7uA	1f7uA03_1f7uA02	3.30.1360.70	1.10.730.10
1fx7B	1fx7B01_1fx7B02	1.10.10.10	1.10.60.10
1fx7B	1fx7B03_1fx7B02	2.30.30.90	1.10.60.10
1g0dA	1g0dA02_1g0dA01	3.90.260.10	2.60.40.10
1g0dA	1g0dA02_1g0dA03	3.90.260.10	2.60.40.10
1g0dA	1g0dA02_1g0dA04	3.90.260.10	2.60.40.10
1g0dA	1g0dA03_1g0dA04	2.60.40.10	2.60.40.10

1g7tA	1g7tA01_1g7tA02	3.40.50.300	2.40.30.10
1g8fA	1g8fA01_1g8fA02	3.40.50.620	3.10.400.10
1gg4A	1gg4A02_1gg4A01	3.40.1190.10	3.90.190.20
1gg4A	1gg4A02_1gg4A03	3.40.1190.10	3.40.1390.10
1gntA	1gntA01_1gntA02	1.20.1270.20	1.20.1270.20
1gntA	1gntA03_1gntA01	3.40.50.2030	1.20.1270.20
1gntA	1gntA03_1gntA02	3.40.50.2030	1.20.1270.20
1gntA	1gntA04_1gntA01	3.40.50.2030	1.20.1270.20
1gntA	1gntA04_1gntA02	3.40.50.2030	1.20.1270.20
1gntA	1gntA04_1gntA03	3.40.50.2030	3.40.50.2030
1gqiA	1gqiA02_1gqiA01	3.20.20.80	3.30.379.10
1gqiA	1gqiA02_1gqiA03	3.20.20.80	3.90.1330.10
1gsoA	1gsoA03_1gsoA01	3.30.470.20	3.40.50.20
1gsoA	1gsoA03_1gsoA04	3.30.470.20	3.90.600.10
1gsoA	1gsoA04_1gsoA01	3.90.600.10	3.40.50.20
1gvhA	1gvhA03_1gvhA02	3.40.50.80	2.40.30.10
1h3fB	1h3fB01_1h3fB02	3.40.50.620	1.10.240.10
1hc7B	1hc7B01_1hc7B02	3.30.930.10	3.40.50.800
1hc7B	1hc7B01_1hc7B03	3.30.930.10	3.30.110.30
1hh2P	1hh2P03_1hh2P02	3.30.300.20	2.40.50.140
1hn0A	1hn0A02_1hn0A01	1.50.10.100	2.60.120.430
1hn0A	1hn0A02_1hn0A03	1.50.10.100	2.70.98.10
1hn0A	1hn0A03_1hn0A04	2.70.98.10	2.60.220.10
1hskA	1hskA01_1hskA02	3.30.465.10	3.30.43.10
1hskA	1hskA02_1hskA03	3.30.43.10	3.90.78.10
1ikpA	1ikpA01_1ikpA02	2.60.120.200	3.90.1350.10
1ikpA	1ikpA03_1ikpA02	3.90.175.10	3.90.1350.10
1in6A	1in6A01_1in6A03	3.40.50.300	1.10.8.60
1in6A	1in6A03_1in6A02	1.10.8.60	1.10.10.10
1iq0A	1iq0A01_1iq0A03	3.40.50.620	1.10.730.10
1iq0A	1iq0A03_1iq0A02	1.10.730.10	3.30.1360.70
1iq8B	1iq8B01_1iq8B02	3.20.20.105	3.90.1020.10
1iq8B	1iq8B01_1iq8B04	3.20.20.105	2.30.130.10
1iq8B	1iq8B03_1iq8B02	3.10.450.90	3.90.1020.10
1iq8B	1iq8B04_1iq8B03	2.30.130.10	3.10.450.90
1itzB	1itzB02_1itzB03	3.40.50.970	3.40.50.920
1j6uA	1j6uA02_1j6uA01	3.40.1190.10	3.40.50.720
1j6uA	1j6uA02_1j6uA03	3.40.1190.10	3.90.190.20
1j72A	1j72A03_1j72A02	3.40.20.10	3.40.20.10
1jb7A	1jb7A01_1jb7A02	2.40.50.140	2.40.50.140
1jb7A	1jb7A02_1jb7A03	2.40.50.140	2.40.50.140
1jeyB	1jeyB01_1jeyB03	3.40.50.410	1.10.1600.10
1ji1A	1ji1A01_1ji1A03	2.60.40.10	2.60.40.1180
1ji1A	1ji1A02_1ji1A01	3.20.20.80	2.60.40.10
1ji1A	1ji1A02_1ji1A03	3.20.20.80	2.60.40.1180
1jmzA	1jmzA02_1jmzA03	2.40.128.120	2.60.40.10
1jmzA	1jmzA04_1jmzA02	2.60.40.10	2.40.128.120
1jmzA	1jmzA04_1jmzA03	2.60.40.10	2.60.40.10
1js3A	1js3A02_1js3A03	3.40.640.10	3.90.1150.10
1js3A	1js3A03_1js3A01	3.90.1150.10	1.20.1340.10
1jz8D	1jz8D01_1jz8D02	2.60.120.260	2.60.40.10
1jz8D	1jz8D03_1jz8D02	3.20.20.80	2.60.40.10
1jz8D	1jz8D03_1jz8D04	3.20.20.80	2.60.40.10

1jz8D	1jz8D03_1jz8D05	3.20.20.80	2.70.98.10
1k32E	1k32E01_1k32E03	2.120.10.60	3.30.750.44
1k32E	1k32E01_1k32E04	2.120.10.60	2.30.42.10
1k32E	1k32E01_1k32E05	2.120.10.60	3.90.226.10
1k32E	1k32E02_1k32E05	2.130.10.10	3.90.226.10
1k32E	1k32E05_1k32E03	3.90.226.10	3.30.750.44
1k32E	1k32E05_1k32E04	3.90.226.10	2.30.42.10
1k3iA	1k3iA02_1k3iA01	2.130.10.80	2.60.120.260
1k3iA	1k3iA02_1k3iA03	2.130.10.80	2.60.40.10
1k9aA	1k9aA03_1k9aA02	3.30.200.20	3.30.505.10
1k9fA	1k9fA02_1k9fA01	3.20.20.80	3.30.379.10
1k9fA	1k9fA02_1k9fA03	3.20.20.80	3.90.1330.10
1kfiB	1kfiB01_1kfiB02	3.40.120.10	3.40.120.10
1kfiB	1kfiB01_1kfiB03	3.40.120.10	3.40.120.10
1kfiB	1kfiB03_1kfiB02	3.40.120.10	3.40.120.10
1kfiB	1kfiB04_1kfiB03	3.30.310.50	3.40.120.10
1kfuL	1kfuL03_1kfuL02	2.60.120.380	3.90.70.10
1kfuL	1kfuL04_1kfuL03	1.10.238.10	2.60.120.380
1kj9A	1kj9A01_1kj9A02	3.40.50.20	3.30.1490.20
1kj9A	1kj9A03_1kj9A01	3.30.470.20	3.40.50.20
1kj9A	1kj9A03_1kj9A02	3.30.470.20	3.30.1490.20
1krhA	1krhA03_1krhA01	3.40.50.80	3.10.20.30
1krhA	1krhA03_1krhA02	3.40.50.80	2.40.30.10
1ksiB	1ksiB03_1ksiB01	2.70.98.20	3.10.450.40
1ksiB	1ksiB03_1ksiB02	2.70.98.20	3.10.450.40
1kwgA	1kwgA01_1kwgA02	3.20.20.80	3.40.50.880
1kwgA	1kwgA01_1kwgA03	3.20.20.80	2.60.40.1180
1kwgA	1kwgA02_1kwgA03	3.40.50.880	2.60.40.1180
1kxpD	1kxpD02_1kxpD01	1.10.246.10	1.10.246.10
1kxpD	1kxpD02_1kxpD03	1.10.246.10	1.10.246.10
1kxpD	1kxpD03_1kxpD04	1.10.246.10	1.10.246.10
1kxpD	1kxpD04_1kxpD05	1.10.246.10	1.10.246.10
1l5hA	1l5hA01_1l5hA03	3.40.50.1980	3.40.50.1980
1l5hA	1l5hA03_1l5hA02	3.40.50.1980	3.40.50.1980
1l9nB	1l9nB02_1l9nB01	3.90.260.10	2.60.40.10
1l9nB	1l9nB02_1l9nB03	3.90.260.10	2.60.40.10
1l9nB	1l9nB02_1l9nB04	3.90.260.10	2.60.40.10
1llaA	1llaA02_1llaA01	2.60.40.1520	1.20.1370.10
1llaA	1llaA03_1llaA01	1.10.1280.10	1.20.1370.10
1llaA	1llaA03_1llaA02	1.10.1280.10	2.60.40.1520
1lmlA	1lmlA01_1lmlA02	3.10.170.20	3.90.132.10
1lmlA	1lmlA02_1lmlA03	3.90.132.10	2.10.55.10
1lmlA	1lmlA04_1lmlA03	2.30.34.10	2.10.55.10
1lshA	1lshA01_1lshA03	2.30.230.10	2.20.50.20
1lshA	1lshA01_1lshA04	2.30.230.10	2.20.80.10
1lshA	1lshA02_1lshA01	1.25.10.20	2.30.230.10
1lshA	1lshA02_1lshA03	1.25.10.20	2.20.50.20
1lshA	1lshA02_1lshA04	1.25.10.20	2.20.80.10
1lvaA	1lvaA02_1lvaA01	1.10.10.10	1.10.10.10
1lvaA	1lvaA03_1lvaA04	1.10.10.10	1.10.10.10
1lvoE	1lvoE02_1lvoE01	2.40.10.10	2.40.10.10
1lxmA	1lxmA01_1lxmA03	2.70.98.10	2.60.220.10
1lxmA	1lxmA02_1lxmA01	1.50.10.100	2.70.98.10

1lxmA	1lxmA02_1lxmA04	1.50.10.100	2.60.40.1380
1m7xD	1m7xD02_1m7xD01	3.20.20.80	2.60.40.10
1m7xD	1m7xD02_1m7xD03	3.20.20.80	2.60.40.1180
1mbmB	1mbmB01_1mbmB02	2.40.10.10	2.40.10.10
1mbmB	1mbmB02_1mbmB03	2.40.10.10	3.30.40.20
1mdbA	1mdbA02_1mdbA01	3.40.50.980	3.40.50.980
1mdbA	1mdbA02_1mdbA03	3.40.50.980	2.30.38.10
1mdbA	1mdbA02_1mdbA04	3.40.50.980	3.30.300.30
1mkyA	1mkyA01_1mkyA03	3.40.50.300	3.30.300.20
1mkyA	1mkyA02_1mkyA03	3.40.50.300	3.30.300.20
1mpgA	1mpgA01_1mpgA03	3.30.310.20	1.10.1670.10
1mpgA	1mpgA02_1mpgA01	1.10.340.30	3.30.310.20
1mpgA	1mpgA02_1mpgA03	1.10.340.30	1.10.1670.10
1musA	1musA02_1musA01	3.90.350.10	1.10.246.40
1musA	1musA02_1musA03	3.90.350.10	1.10.740.10
1musA	1musA03_1musA01	1.10.740.10	1.10.246.40
1mv8C	1mv8C01_1mv8C02	3.40.50.720	1.20.5.170
1mv8C	1mv8C03_1mv8C02	3.40.50.720	1.20.5.170
1n00A	1n00A01_1n00A03	1.10.220.10	1.10.220.10
1n00A	1n00A02_1n00A04	1.10.220.10	1.10.220.10
1n52A	1n52A01_1n52A02	1.25.40.180	1.25.40.180
1n52A	1n52A01_1n52A03	1.25.40.180	1.25.40.180
1n52A	1n52A03_1n52A02	1.25.40.180	1.25.40.180
1n62C	1n62C03_1n62C01	3.30.465.10	3.30.43.10
1n62C	1n62C03_1n62C02	3.30.465.10	3.30.390.50
1n7qA	1n7qA01_1n7qA03	2.70.98.10	2.60.220.10
1n8yC	1n8yC01_1n8yC02	3.80.20.20	2.10.220.10
1n8yC	1n8yC03_1n8yC01	3.80.20.20	3.80.20.20
1n8yC	1n8yC03_1n8yC02	3.80.20.20	2.10.220.10
1n8yC	1n8yC03_1n8yC04	3.80.20.20	2.10.220.10
1nkgA	1nkgA01_1nkgA02	2.70.98.10	2.60.40.1120
1nkgA	1nkgA01_1nkgA03	2.70.98.10	2.60.120.260
1np7B	1np7B02_1np7B01	1.25.40.80	3.40.50.620
1np7B	1np7B03_1np7B01	1.10.579.10	3.40.50.620
1np7B	1np7B03_1np7B02	1.10.579.10	1.25.40.80
1npeB	1npeB02_1npeB01	2.10.25.10	2.10.25.10
1npeB	1npeB02_1npeB03	2.10.25.10	2.10.25.10
1o7fA	1o7fA01_1o7fA03	2.60.120.10	2.60.120.10
1o7fA	1o7fA02_1o7fA03	1.10.10.10	2.60.120.10
1oaoC	1oaoC02_1oaoC01	3.40.50.2030	1.10.8.190
1oaoC	1oaoC02_1oaoC03	3.40.50.2030	3.30.1650.10
1oaoC	1oaoC03_1oaoC04	3.30.1650.10	3.40.970.20
1oaoC	1oaoC05_1oaoC04	3.40.1470.10	3.40.970.20
1ofcX	1ofcX02_1ofcX03	1.10.10.60	1.20.5.1190
1ofcX	1ofcX04_1ofcX03	1.10.10.60	1.20.5.1190
1ofeB	1ofeB01_1ofeB02	3.60.20.10	3.20.20.70
1ofeB	1ofeB01_1ofeB04	3.60.20.10	2.160.20.60
1ofeB	1ofeB03_1ofeB01	3.20.20.70	3.60.20.10
1ofeB	1ofeB03_1ofeB02	3.20.20.70	3.20.20.70
1ofeB	1ofeB03_1ofeB04	3.20.20.70	2.160.20.60
1okgA	1okgA01_1okgA02	3.40.250.10	3.40.250.10
1okgA	1okgA02_1okgA03	3.40.250.10	3.30.1670.10
1olzA	1olzA01_1olzA02	2.130.10.10	3.30.1680.10

1olzA	1olzA03_1olzA02	2.60.40.10	3.30.1680.10
1opkA	1opkA02_1opkA01	3.30.505.10	2.30.30.40
1opkA	1opkA04_1opkA02	1.10.510.10	3.30.505.10
1oywA	1oywA01_1oywA03	3.40.50.300	1.10.10.10
1oywA	1oywA02_1oywA03	3.40.50.300	1.10.10.10
1ozhA	1ozhA01_1ozhA02	3.40.50.970	3.40.50.1220
1ozhA	1ozhA04_1ozhA01	3.40.50.970	3.40.50.970
1ozhA	1ozhA04_1ozhA02	3.40.50.970	3.40.50.1220
1pbyA	1pbyA02_1pbyA03	2.40.128.120	2.60.40.10
1pbyA	1pbyA04_1pbyA03	2.60.40.10	2.60.40.10
1pixA	1pixA02_1pixA01	3.90.226.10	1.20.5.680
1pixA	1pixA03_1pixA01	3.90.226.10	1.20.5.680
1pixA	1pixA03_1pixA02	3.90.226.10	3.90.226.10
1pjqB	1pjqB04_1pjqB03	3.40.1010.10	1.10.8.210
1pjqB	1pjqB05_1pjqB04	3.30.950.10	3.40.1010.10
1pxyB	1pxyB01_1pxyB02	1.10.418.10	1.10.418.10
1pxyB	1pxyB01_1pxyB04	1.10.418.10	1.10.418.10
1pxyB	1pxyB03_1pxyB04	1.10.418.10	1.10.418.10
1q25A	1q25A02_1q25A01	2.70.130.10	2.70.130.10
1q25A	1q25A02_1q25A03	2.70.130.10	2.70.130.10
1q25A	1q25A03_1q25A01	2.70.130.10	2.70.130.10
1q6zA	1q6zA01_1q6zA02	3.40.50.970	3.40.50.1220
1q6zA	1q6zA03_1q6zA01	3.40.50.970	3.40.50.970
1q6zA	1q6zA03_1q6zA02	3.40.50.970	3.40.50.1220
1qsaA	1qsaA01_1qsaA02	1.25.20.10	1.10.1240.20
1qsaA	1qsaA01_1qsaA03	1.25.20.10	1.10.530.10
1qsaA	1qsaA03_1qsaA02	1.10.530.10	1.10.1240.20
1r0vD	1r0vD03_1r0vD01	3.40.1350.10	3.40.1350.10
1r0vD	1r0vD03_1r0vD02	3.40.1350.10	3.40.1170.20
1r2jA	1r2jA02_1r2jA01	2.40.110.10	1.10.540.10
1r2jA	1r2jA03_1r2jA01	1.20.140.10	1.10.540.10
1r2jA	1r2jA03_1r2jA02	1.20.140.10	2.40.110.10
1r5bA	1r5bA01_1r5bA02	3.40.50.300	2.40.30.10
1r5bA	1r5bA01_1r5bA03	3.40.50.300	2.40.30.10
1r5bA	1r5bA03_1r5bA02	2.40.30.10	2.40.30.10
1r6bX	1r6bX02_1r6bX03	3.40.50.300	1.10.8.60
1r6bX	1r6bX04_1r6bX05	3.40.50.300	1.10.8.60
1r6tA	1r6tA02_1r6tA03	3.40.50.620	1.10.240.10
1r8eA	1r8eA01_1r8eA03	3.20.80.10	1.20.5.490
1r9jA	1r9jA01_1r9jA02	3.40.50.970	3.40.50.970
1rrkA	1rrkA01_1rrkA02	3.40.50.410	2.40.10.10
1rrkA	1rrkA03_1rrkA02	2.40.10.10	2.40.10.10
1rt8A	1rt8A01_1rt8A04	1.10.418.10	1.10.418.10
1rt8A	1rt8A03_1rt8A04	1.10.418.10	1.10.418.10
1rwhA	1rwhA01_1rwhA02	1.50.10.100	2.70.98.10
1rwhA	1rwhA02_1rwhA03	2.70.98.10	2.60.220.10
1rx0B	1rx0B01_1rx0B02	1.10.540.10	2.40.110.10
1rx0B	1rx0B03_1rx0B01	1.20.140.10	1.10.540.10
1rx0B	1rx0B03_1rx0B02	1.20.140.10	2.40.110.10
1sjiA	1sjiA01_1sjiA02	3.40.30.10	3.40.30.10
1sjiA	1sjiA03_1sjiA02	3.40.30.10	3.40.30.10
1sotB	1sotB02_1sotB01	2.40.10.10	2.40.10.10
1soxA	1soxA02_1soxA01	3.90.420.10	3.10.120.10

1soxA	1soxA02_1soxA03	3.90.420.10	2.60.40.650
1spuA	1spuA03_1spuA02	2.70.98.20	3.10.450.40
1spuA	1spuA03_1spuA04	2.70.98.20	3.10.450.40
1sqgA	1sqgA02_1sqgA04	1.10.287.730	3.40.50.150
1sqgA	1sqgA04_1sqgA01	3.40.50.150	1.10.940.10
1sqgA	1sqgA04_1sqgA03	3.40.50.150	3.30.70.1170
1sr8A	1sr8A02_1sr8A01	3.30.2110.10	3.30.1990.10
1sr8A	1sr8A03_1sr8A01	3.40.50.10720	3.30.1990.10
1su8A	1su8A01_1su8A02	1.20.1270.30	3.40.50.2030
1su8A	1su8A03_1su8A01	3.40.50.2030	1.20.1270.30
1su8A	1su8A03_1su8A02	3.40.50.2030	3.40.50.2030
1t3qF	1t3qF02_1t3qF01	3.30.465.10	3.30.43.10
1t3qF	1t3qF02_1t3qF03	3.30.465.10	3.30.390.50
1t9bB	1t9bB03_1t9bB01	3.40.50.970	3.40.50.970
1t9bB	1t9bB03_1t9bB02	3.40.50.970	3.40.50.1220
1t9hA	1t9hA02_1t9hA01	3.40.50.300	2.40.50.140
1t9hA	1t9hA02_1t9hA03	3.40.50.300	1.10.40.50
1td6A	1td6A01_1td6A02	1.20.1480.10	3.30.1790.10
1td6A	1td6A01_1td6A03	1.20.1480.10	1.10.472.40
1td6A	1td6A02_1td6A03	3.30.1790.10	1.10.472.40
1tuoA	1tuoA01_1tuoA02	3.40.120.10	3.40.120.10
1tuoA	1tuoA01_1tuoA03	3.40.120.10	3.40.120.10
1tuoA	1tuoA03_1tuoA02	3.40.120.10	3.40.120.10
1tuoA	1tuoA03_1tuoA04	3.40.120.10	3.30.310.50
1u4qB	1u4qB02_1u4qB03	1.20.58.60	1.20.58.60
1u8vC	1u8vC01_1u8vC02	1.10.3140.10	2.40.110.10
1u8vC	1u8vC03_1u8vC01	1.20.140.10	1.10.3140.10
1u8vC	1u8vC03_1u8vC02	1.20.140.10	2.40.110.10
1uebA	1uebA02_1uebA03	2.40.50.140	2.40.50.140
1ulzA	1ulzA03_1ulzA01	3.30.470.20	3.40.50.20
1uzkA	1uzkA02_1uzkA01	3.90.290.10	2.10.25.10
1v10A	1v10A02_1v10A01	2.60.40.420	2.60.40.420
1v10A	1v10A03_1v10A01	2.60.40.420	2.60.40.420
1v10A	1v10A03_1v10A02	2.60.40.420	2.60.40.420
1v4aA	1v4aA02_1v4aA01	3.30.460.10	1.10.4050.10
1v4aA	1v4aA02_1v4aA03	3.30.460.10	1.20.120.330
1v4aA	1v4aA03_1v4aA01	1.20.120.330	1.10.4050.10
1vbkA	1vbkA01_1vbkA02	3.30.70.1510	3.30.2300.10
1vbkA	1vbkA03_1vbkA01	3.40.50.620	3.30.70.1510
1vclB	1vclB01_1vclB02	2.80.10.50	2.80.10.50
1vclB	1vclB01_1vclB03	2.80.10.50	3.30.1750.10
1vclB	1vclB03_1vclB02	3.30.1750.10	2.80.10.50
1vkzA	1vkzA03_1vkzA01	3.30.470.20	3.40.50.20
1vkzA	1vkzA03_1vkzA04	3.30.470.20	3.90.600.10
1vpkA	1vpkA02_1vpkA01	3.10.150.10	3.10.150.10
1vpkA	1vpkA02_1vpkA03	3.10.150.10	3.10.150.10
1vqoA	1vqoA01_1vqoA02	2.40.50.140	2.30.30.30
1vqoA	1vqoA01_1vqoA03	2.40.50.140	4.10.950.10
1w07A	1w07A02_1w07A01	2.40.110.10	1.10.540.10
1w07A	1w07A03_1w07A01	1.20.140.10	1.10.540.10
1w07A	1w07A03_1w07A04	1.20.140.10	1.20.140.10
1w07A	1w07A04_1w07A01	1.20.140.10	1.10.540.10
1w7bA	1w7bA01_1w7bA04	1.10.220.10	1.10.220.10

1w7bA	1w7bA02_1w7bA03	1.10.220.10	1.10.220.10
1w7bA	1w7bA02_1w7bA04	1.10.220.10	1.10.220.10
1w96B	1w96B03_1w96B02	3.30.1490.20	3.90.1770.10
1w96B	1w96B04_1w96B02	3.30.470.20	3.90.1770.10
1wvfA	1wvfA01_1wvfA04	3.30.43.10	1.10.45.10
1wvfA	1wvfA02_1wvfA01	3.30.465.10	3.30.43.10
1wvfA	1wvfA02_1wvfA04	3.30.465.10	1.10.45.10
1wvfA	1wvfA03_1wvfA01	3.40.462.10	3.30.43.10
1wvfA	1wvfA03_1wvfA02	3.40.462.10	3.30.465.10
1wxxD	1wxxD02_1wxxD01	3.30.750.80	2.30.130.10
1wxxD	1wxxD03_1wxxD02	3.40.50.150	3.30.750.80
1x1iA	1x1iA01_1x1iA02	1.50.10.100	2.70.98.10
1x1iA	1x1iA02_1x1iA03	2.70.98.10	2.60.220.10
1x6vA	1x6vA03_1x6vA01	3.40.50.620	3.40.50.300
1x6vA	1x6vA03_1x6vA02	3.40.50.620	3.10.400.10
1xszA	1xszA02_1xszA01	1.10.1000.11	1.10.220.20
1xszA	1xszA03_1xszA02	3.30.310.140	1.10.1000.11
1ybyB	1ybyB02_1ybyB03	2.40.50.140	2.40.50.140
1yq2E	1yq2E01_1yq2E02	2.60.120.260	2.60.40.10
1yq2E	1yq2E03_1yq2E01	3.20.20.80	2.60.120.260
1yq2E	1yq2E03_1yq2E02	3.20.20.80	2.60.40.10
1yq2E	1yq2E03_1yq2E04	3.20.20.80	2.60.40.10
1yq2E	1yq2E05_1yq2E03	2.70.98.10	3.20.20.80
1yq2E	1yq2E05_1yq2E04	2.70.98.10	2.60.40.10
1yt8A	1yt8A01_1yt8A03	3.40.250.10	3.40.250.10
1yt8A	1yt8A02_1yt8A03	3.40.250.10	3.40.250.10
1z6tC	1z6tC01_1z6tC04	1.10.533.10	1.10.10.10
1z6tC	1z6tC02_1z6tC01	3.40.50.300	1.10.533.10
1z6tC	1z6tC02_1z6tC04	3.40.50.300	1.10.10.10
1zarA	1zarA01_1zarA02	1.10.10.10	3.30.200.20
1zarA	1zarA03_1zarA02	1.10.510.10	3.30.200.20
2ahxA	2ahxA03_2ahxA04	3.80.20.20	2.10.220.10
2as0A	2as0A02_2as0A01	3.30.750.80	2.30.130.10
2as0A	2as0A03_2as0A02	3.40.50.150	3.30.750.80
2asbA	2asbA02_2asbA01	3.30.300.20	2.40.50.140
2asbA	2asbA02_2asbA03	3.30.300.20	3.30.300.20
2au3A	2au3A02_2au3A01	3.90.980.10	3.90.580.10
2au3A	2au3A02_2au3A03	3.90.980.10	3.40.1360.10
2b78A	2b78A02_2b78A01	3.30.750.80	2.30.130.10
2b78A	2b78A03_2b78A02	3.40.50.150	3.30.750.80
2bcqA	2bcqA03_2bcqA02	3.30.460.10	1.10.150.20
2bcqA	2bcqA03_2bcqA04	3.30.460.10	3.30.210.10
2be4A	2be4A02_2be4A01	1.10.238.10	1.10.238.10
2be4A	2be4A02_2be4A03	1.10.238.10	1.10.238.10
2bm0A	2bm0A01_2bm0A02	3.40.50.300	2.40.30.10
2bm0A	2bm0A01_2bm0A05	3.40.50.300	3.30.70.240
2btoA	2btoA01_2btoA02	3.40.50.1440	3.30.1330.20
2btoA	2btoA01_2btoA03	3.40.50.1440	1.10.287.600
2btoA	2btoA02_2btoA03	3.30.1330.20	1.10.287.600
2bzdB	2bzdB01_2bzdB02	2.120.10.10	2.60.40.10
2bzdB	2bzdB03_2bzdB02	2.60.120.260	2.60.40.10
2c10C	2c10C02_2c10C01	3.10.450.40	3.10.450.40
2c10C	2c10C03_2c10C02	2.70.98.20	3.10.450.40

2c12E	2c12E02_2c12E01	2.40.110.10	1.10.540.10
2c12E	2c12E03_2c12E01	1.20.140.10	1.10.540.10
2c12E	2c12E03_2c12E02	1.20.140.10	2.40.110.10
2c42A	2c42A01_2c42A02	3.40.50.970	3.40.50.920
2c42A	2c42A01_2c42A03	3.40.50.970	3.40.920.10
2c42A	2c42A03_2c42A02	3.40.920.10	3.40.50.920
2c42A	2c42A06_2c42A01	3.40.50.970	3.40.50.970
2c42A	2c42A06_2c42A05	3.40.50.970	3.30.70.20
2c42A	2c42A06_2c42A07	3.40.50.970	4.10.790.10
2chnA	2chnA02_2chnA01	3.20.20.80	3.30.379.10
2chnA	2chnA02_2chnA03	3.20.20.80	1.20.58.460
2chnA	2chnA03_2chnA04	1.20.58.460	2.60.40.1180
2ck3A	2ck3A02_2ck3A01	3.40.50.300	2.40.30.20
2ck3A	2ck3A02_2ck3A03	3.40.50.300	1.20.150.20
2csuA	2csuA02_2csuA01	3.40.50.261	3.40.50.720
2csuA	2csuA03_2csuA02	3.40.50.261	3.40.50.261
2d29A	2d29A02_2d29A01	2.40.110.10	1.10.540.10
2d29A	2d29A03_2d29A01	1.20.140.10	1.10.540.10
2d73A	2d73A01_2d73A03	2.70.98.10	2.60.40.1180
2d73A	2d73A02_2d73A01	3.20.20.70	2.70.98.10
2d73A	2d73A02_2d73A03	3.20.20.70	2.60.40.1180
2ddhA	2ddhA02_2ddhA01	2.40.110.10	1.10.540.10
2djiA	2djiA03_2djiA01	3.40.50.970	3.40.50.970
2djiA	2djiA03_2djiA02	3.40.50.970	3.40.50.1220
2dvlA	2dvlA01_2dvlA02	1.10.540.10	2.40.110.10
2dvlA	2dvlA03_2dvlA01	1.20.140.10	1.10.540.10
2dvlA	2dvlA03_2dvlA02	1.20.140.10	2.40.110.10
2dwcA	2dwcA01_2dwcA02	3.40.50.20	3.30.1490.20
2dwcA	2dwcA03_2dwcA02	3.30.470.20	3.30.1490.20
2dy1A	2dy1A01_2dy1A02	3.40.50.300	2.40.30.10
2dy1A	2dy1A01_2dy1A03	3.40.50.300	3.30.70.870
2dy1A	2dy1A02_2dy1A03	2.40.30.10	3.30.70.870
2ebaI	2ebaI01_2ebaI02	1.10.540.10	2.40.110.10
2ebaI	2ebaI03_2ebaI01	1.20.140.10	1.10.540.10
2ebaI	2ebaI03_2ebaI02	1.20.140.10	2.40.110.10
2eplX	2eplX02_2eplX01	3.20.20.80	3.30.160.230
2eplX	2eplX02_2eplX03	3.20.20.80	1.20.120.670
2f2hC	2f2hC02_2f2hC01	3.20.20.80	2.60.40.1760
2f2hC	2f2hC02_2f2hC03	3.20.20.80	2.60.40.1180
2f2hC	2f2hC02_2f2hC04	3.20.20.80	2.60.40.1180
2f2hC	2f2hC04_2f2hC03	2.60.40.1180	2.60.40.1180
2fd6U	2fd6U02_2fd6U01	2.10.60.10	2.10.60.10
2fd6U	2fd6U02_2fd6U03	2.10.60.10	2.10.60.10
2ff4B	2ff4B02_2ff4B01	1.25.40.10	1.10.10.10
2ff4B	2ff4B02_2ff4B03	1.25.40.10	2.60.200.20
2fh1A	2fh1A03_2fh1A02	3.40.20.10	3.40.20.10
2fh8A	2fh8A01_2fh8A02	2.60.40.1130	2.60.40.10
2fh8A	2fh8A03_2fh8A01	3.20.20.80	2.60.40.1130
2fh8A	2fh8A03_2fh8A02	3.20.20.80	2.60.40.10
2fh8A	2fh8A03_2fh8A04	3.20.20.80	2.60.40.1180
2fmlA	2fmlA02_2fmlA03	3.90.79.10	1.10.10.10
2fnaA	2fnaA01_2fnaA02	3.40.50.300	1.10.8.60
2fnaA	2fnaA02_2fnaA03	1.10.8.60	1.10.10.10

2fokA	2fokA01_2fokA02	3.90.241.10	1.10.10.10
2fokA	2fokA01_2fokA03	3.90.241.10	3.40.91.30
2fuvB	2fuvB01_2fuvB02	3.40.120.10	3.40.120.10
2fuvB	2fuvB01_2fuvB03	3.40.120.10	3.40.120.10
2fuvB	2fuvB03_2fuvB02	3.40.120.10	3.40.120.10
2fuvB	2fuvB03_2fuvB04	3.40.120.10	3.30.310.50
2gksA	2gksA02_2gksA01	3.40.50.620	3.10.400.10
2gnoA	2gnoA03_2gnoA02	1.20.272.10	1.10.8.250
2gqtA	2gqtA02_2gqtA01	3.30.465.10	3.30.43.10
2gqtA	2gqtA03_2gqtA01	3.90.78.10	3.30.43.10
2he7A	2he7A02_2he7A01	1.20.80.10	3.10.20.90
2he7A	2he7A02_2he7A03	1.20.80.10	2.30.29.30
2hkjA	2hkjA01_2hkjA03	3.30.565.10	3.30.230.10
2hmaA	2hmaA01_2hmaA02	3.40.50.620	2.30.30.280
2hmaA	2hmaA01_2hmaA03	3.40.50.620	2.40.30.10
2hmaA	2hmaA03_2hmaA02	2.40.30.10	2.30.30.280
2hoxB	2hoxB02_2hoxB01	3.40.640.10	2.10.25.30
2hoxB	2hoxB02_2hoxB03	3.40.640.10	3.90.1150.10
2hoxB	2hoxB03_2hoxB01	3.90.1150.10	2.10.25.30
2hr7A	2hr7A01_2hr7A02	3.80.20.20	2.10.220.10
2i0kA	2i0kA01_2i0kA04	3.30.43.10	1.10.45.10
2i0kA	2i0kA02_2i0kA04	3.30.465.10	1.10.45.10
2i0kA	2i0kA03_2i0kA04	3.40.462.10	1.10.45.10
2ihtD	2ihtD03_2ihtD01	3.40.50.970	3.40.50.970
2ihtD	2ihtD03_2ihtD02	3.40.50.970	3.40.50.1220
2j8gA	2j8gA02_2j8gA03	2.10.270.10	2.20.120.10
2jbrD	2jbrD01_2jbrD02	1.10.540.10	2.40.110.10
2jbrD	2jbrD03_2jbrD01	1.20.140.10	1.10.540.10
2jbrD	2jbrD03_2jbrD02	1.20.140.10	2.40.110.10
2je8B	2je8B01_2je8B02	2.60.120.260	2.60.40.10
2je8B	2je8B03_2je8B01	3.20.20.80	2.60.120.260
2je8B	2je8B03_2je8B02	3.20.20.80	2.60.40.10
2je8B	2je8B03_2je8B04	3.20.20.80	2.60.40.10
2je8B	2je8B03_2je8B05	3.20.20.80	2.60.40.10
2je8B	2je8B04_2je8B05	2.60.40.10	2.60.40.10
2jifB	2jifB02_2jifB01	2.40.110.10	1.10.540.10
2jifB	2jifB03_2jifB01	1.20.140.10	1.10.540.10
2mbrA	2mbrA01_2mbrA02	3.90.78.10	3.30.43.10
2mbrA	2mbrA03_2mbrA01	3.30.465.10	3.90.78.10
2mbrA	2mbrA03_2mbrA02	3.30.465.10	3.30.43.10
2nxwB	2nxwB01_2nxwB02	3.40.50.970	3.40.50.1220
2nxwB	2nxwB03_2nxwB01	3.40.50.970	3.40.50.970
2nxwB	2nxwB03_2nxwB02	3.40.50.970	3.40.50.1220
2nz2A	2nz2A02_2nz2A01	3.90.1260.10	3.40.50.620
2o26U	2o26U02_2o26U01	2.60.40.10	2.60.40.10
2o7sA	2o7sA01_2o7sA02	3.20.20.70	3.40.50.10860
2o7sA	2o7sA03_2o7sA02	3.40.50.720	3.40.50.10860
2odqA	2odqA01_2odqA02	3.40.50.410	2.40.10.10
2odqA	2odqA03_2odqA02	2.40.10.10	2.40.10.10
2or0B	2or0B03_2or0B01	1.20.140.10	1.10.540.10
2or0B	2or0B03_2or0B02	1.20.140.10	2.40.110.10
2p67A	2p67A02_2p67A03	3.40.50.300	1.10.287.130
2pfmB	2pfmB02_2pfmB01	1.20.200.10	1.10.275.10

2pfmB	2pfmB02_2pfmB03	1.20.200.10	1.10.40.30
2pg0A	2pg0A02_2pg0A01	2.40.110.10	1.10.540.10
2pg0A	2pg0A03_2pg0A01	1.20.140.10	1.10.540.10
2pg0A	2pg0A03_2pg0A02	1.20.140.10	2.40.110.10
2piaA	2piaA01_2piaA03	2.40.30.10	3.10.20.30
2piaA	2piaA02_2piaA01	3.40.50.80	2.40.30.10
2pjuD	2pjuD01_2pjuD03	3.40.50.2300	1.20.5.170
2pw9B	2pw9B03_2pw9B02	3.40.140.10	3.10.20.10
2pz1A	2pz1A02_2pz1A01	1.20.900.10	2.30.30.40
2pz1A	2pz1A02_2pz1A03	1.20.900.10	2.30.29.30
2q07A	2q07A01_2q07A02	3.40.50.10630	3.10.450.90
2q07A	2q07A03_2q07A02	2.30.130.10	3.10.450.90
2q0zX	2q0zX01_2q0zX03	1.10.3380.10	2.60.40.150
2q28B	2q28B01_2q28B02	3.40.50.970	3.40.50.1220
2q28B	2q28B03_2q28B01	3.40.50.970	3.40.50.970
2q28B	2q28B03_2q28B02	3.40.50.970	3.40.50.1220
2q3eL	2q3eL01_2q3eL02	3.40.50.720	1.20.5.100
2q3eL	2q3eL03_2q3eL01	3.40.50.720	3.40.50.720
2q3eL	2q3eL03_2q3eL02	3.40.50.720	1.20.5.100
2q6dA	2q6dA02_2q6dA01	2.40.10.10	2.40.10.10
2q9oB	2q9oB02_2q9oB01	2.60.40.420	2.60.40.420
2q9oB	2q9oB03_2q9oB01	2.60.40.420	2.60.40.420
2q9oB	2q9oB03_2q9oB02	2.60.40.420	2.60.40.420
2qenA	2qenA01_2qenA02	3.40.50.300	1.10.8.60
2qenA	2qenA02_2qenA03	1.10.8.60	1.10.10.10
2qm8B	2qm8B02_2qm8B03	3.40.50.300	1.10.287.130
2qn6A	2qn6A01_2qn6A02	3.40.50.300	2.40.30.10
2qn6A	2qn6A01_2qn6A03	3.40.50.300	2.40.30.10
2qn6A	2qn6A02_2qn6A03	2.40.30.10	2.40.30.10
2qqmA	2qqmA03_2qqmA01	2.60.120.260	2.60.120.290
2qqoA	2qqoA02_2qqoA01	2.60.120.260	2.60.120.290
2qqoA	2qqoA02_2qqoA03	2.60.120.260	2.60.120.260
2qyuA	2qyuA02_2qyuA01	1.25.40.300	2.160.20.80
2qyuA	2qyuA02_2qyuA03	1.25.40.300	3.40.1850.10
2qyuA	2qyuA04_2qyuA03	1.10.4140.10	3.40.1850.10
2r09B	2r09B02_2r09B01	1.10.1000.11	1.10.220.20
2r09B	2r09B03_2r09B02	2.30.29.30	1.10.1000.11
2r44A	2r44A02_2r44A03	3.40.50.300	1.10.8.300
2rcnA	2rcnA02_2rcnA01	3.40.50.300	2.40.50.140
2re3B	2re3B01_2re3B02	3.10.540.10	2.30.270.10
2re3B	2re3B03_2re3B02	2.20.70.10	2.30.270.10
2rfqB	2rfqB01_2rfqB02	1.10.540.10	2.40.110.10
2rfqB	2rfqB03_2rfqB01	1.20.140.10	1.10.540.10
2rfqB	2rfqB03_2rfqB02	1.20.140.10	2.40.110.10
2uxtB	2uxtB01_2uxtB03	2.60.40.420	2.60.40.420
2uxtB	2uxtB02_2uxtB01	2.60.40.420	2.60.40.420
2uxtB	2uxtB02_2uxtB03	2.60.40.420	2.60.40.420
2uz1C	2uz1C01_2uz1C02	3.40.50.970	3.40.50.1220
2uz1C	2uz1C03_2uz1C01	3.40.50.970	3.40.50.970
2uz1C	2uz1C03_2uz1C02	3.40.50.970	3.40.50.1220
2v1xB	2v1xB01_2v1xB02	3.40.50.300	3.40.50.300
2v1xB	2v1xB02_2v1xB03	3.40.50.300	1.10.10.10
2v5cA	2v5cA02_2v5cA01	3.20.20.80	3.30.379.10

2v5cA	2v5cA02_2v5cA03	3.20.20.80	1.20.58.460
2v5mA	2v5mA01_2v5mA04	2.60.40.10	2.60.40.10
2v5mA	2v5mA02_2v5mA03	2.60.40.10	2.60.40.10
2vbfB	2vbfB01_2vbfB02	3.40.50.970	3.40.50.1220
2vbfB	2vbfB03_2vbfB02	3.40.50.970	3.40.50.1220
2vigB	2vigB02_2vigB01	2.40.110.10	1.10.540.10
2vk8D	2vk8D01_2vk8D02	3.40.50.970	3.40.50.1220
2vk8D	2vk8D03_2vk8D01	3.40.50.970	3.40.50.970
2vk8D	2vk8D03_2vk8D02	3.40.50.970	3.40.50.1220
2vrwB	2vrwB01_2vrwB03	1.20.900.10	3.30.60.20
2vrwB	2vrwB02_2vrwB03	2.30.29.30	3.30.60.20
2vseA	2vseA02_2vseA03	2.80.10.50	2.80.10.50
2vseA	2vseA04_2vseA05	2.80.10.50	2.80.10.50
2vtfB	2vtfB01_2vtfB02	3.20.20.80	2.60.120.260
2vtfB	2vtfB01_2vtfB03	3.20.20.80	2.60.40.10
2vtfB	2vtfB02_2vtfB03	2.60.120.260	2.60.40.10
2vycJ	2vycJ02_2vycJ03	3.40.640.10	3.90.1150.10
2vycJ	2vycJ02_2vycJ04	3.40.640.10	3.90.100.10
2vycJ	2vycJ03_2vycJ04	3.90.1150.10	3.90.100.10
2vzsA	2vzsA01_2vzsA02	2.60.120.260	2.60.40.10
2vzsA	2vzsA03_2vzsA01	3.20.20.80	2.60.120.260
2vzsA	2vzsA03_2vzsA02	3.20.20.80	2.60.40.10
2vzsA	2vzsA03_2vzsA04	3.20.20.80	2.60.40.10
2vzsA	2vzsA03_2vzsA05	3.20.20.80	2.60.40.10
2vzsA	2vzsA04_2vzsA05	2.60.40.10	2.60.40.10
2w9mA	2w9mA03_2w9mA02	3.30.460.10	1.10.150.20
2w9mA	2w9mA05_2w9mA01	3.20.20.140	1.10.150.110
2w9mA	2w9mA05_2w9mA02	3.20.20.140	1.10.150.20
2w9mA	2w9mA05_2w9mA03	3.20.20.140	3.30.460.10
2wcoA	2wcoA01_2wcoA02	1.50.10.100	2.70.98.10
2wcoA	2wcoA02_2wcoA03	2.70.98.10	2.60.220.10
2wlrA	2wlrA02_2wlrA01	3.40.250.10	3.40.250.10
2wlrA	2wlrA02_2wlrA03	3.40.250.10	3.40.250.10
2wqrB	2wqrB03_2wqrB02	2.60.40.10	2.60.40.10
2wsdA	2wsdA01_2wsdA03	2.60.40.420	2.60.40.420
2wsdA	2wsdA02_2wsdA01	2.60.40.420	2.60.40.420
2wsdA	2wsdA02_2wsdA03	2.60.40.420	2.60.40.420
2wskA	2wskA02_2wskA01	3.20.20.80	2.60.40.10
2wskA	2wskA02_2wskA03	3.20.20.80	2.60.40.1180
2wzkA	2wzkA01_2wzkA02	1.20.1310.10	1.20.1310.10
2wzkA	2wzkA02_2wzkA03	1.20.1310.10	1.20.1310.10
2x5oA	2x5oA02_2x5oA01	3.40.1190.10	3.40.50.720
2x5oA	2x5oA02_2x5oA03	3.40.1190.10	3.90.190.20
2x8xX	2x8xX03_2x8xX02	3.10.20.310	3.10.20.310
2xu9A	2xu9A02_2xu9A01	2.60.40.420	2.60.40.420
2xu9A	2xu9A02_2xu9A03	2.60.40.420	2.60.40.420
2xu9A	2xu9A03_2xu9A01	2.60.40.420	2.60.40.420
2y1nA	2y1nA01_2y1nA02	1.20.930.20	1.10.238.10
2y1nA	2y1nA01_2y1nA03	1.20.930.20	3.30.505.10
2y1nA	2y1nA01_2y1nA04	1.20.930.20	3.30.40.10
2y1nA	2y1nA02_2y1nA03	1.10.238.10	3.30.505.10
2y1nA	2y1nA02_2y1nA04	1.10.238.10	3.30.40.10
2ya0A	2ya0A02_2ya0A01	3.20.20.80	2.60.40.10

2ya0A	2ya0A02_2ya0A03	3.20.20.80	2.60.40.1180
2yevD	2yevD01_2yevD02	1.20.210.10	1.10.287.70
2yevD	2yevD01_2yevD03	1.20.210.10	1.20.120.80
2yfoA	2yfoA02_2yfoA01	3.20.20.70	2.70.98.60
2yfoA	2yfoA02_2yfoA03	3.20.20.70	2.60.40.1180
2yv5A	2yv5A02_2yv5A01	3.40.50.300	2.40.50.140
2yv5A	2yv5A02_2yv5A03	3.40.50.300	1.10.40.50
2ywcC	2ywcC01_2ywcC02	3.40.50.880	3.40.50.620
2ywcC	2ywcC02_2ywcC03	3.40.50.620	3.30.300.10
2yweA	2yweA01_2yweA02	3.40.50.300	2.40.30.10
2yweA	2yweA02_2yweA03	2.40.30.10	3.30.70.870
2yykA	2yykA01_2yykA02	1.10.3140.10	2.40.110.10
2yykA	2yykA03_2yykA01	1.20.140.10	1.10.3140.10
2yykA	2yykA03_2yykA02	1.20.140.10	2.40.110.10
2z1qA	2z1qA01_2z1qA04	1.10.540.10	1.20.140.10
2z1qA	2z1qA03_2z1qA04	1.20.140.10	1.20.140.10
2z9iA	2z9iA02_2z9iA01	2.40.10.10	2.40.10.10
2z9iA	2z9iA02_2z9iA03	2.40.10.10	2.30.42.10
2zpyA	2zpyA02_2zpyA01	1.20.80.10	3.10.20.90
2zpyA	2zpyA02_2zpyA03	1.20.80.10	2.30.29.30
2ztdA	2ztdA01_2ztdA02	2.40.50.140	1.10.150.20
2zueA	2zueA02_2zueA03	3.40.50.620	1.10.730.10
2zueA	2zueA03_2zueA01	1.10.730.10	3.30.1360.70
2zxqA	2zxqA01_2zxqA03	2.70.98.10	2.60.40.1180
2zxqA	2zxqA02_2zxqA01	3.20.20.80	2.70.98.10
2zxqA	2zxqA02_2zxqA03	3.20.20.80	2.60.40.1180
2zxqA	2zxqA02_2zxqA04	3.20.20.80	2.60.120.260
2zxqA	2zxqA02_2zxqA05	3.20.20.80	2.60.120.260
2zxqA	2zxqA05_2zxqA04	2.60.120.260	2.60.120.260
2zxqA	2zxqA05_2zxqA06	2.60.120.260	1.20.1270.70
2zydA	2zydA02_2zydA01	1.10.1040.10	3.40.50.720
3a21A	3a21A01_3a21A02	3.20.20.70	2.60.40.1180
3a24A	3a24A01_3a24A03	2.70.98.10	2.60.40.1180
3a24A	3a24A02_3a24A01	3.20.20.70	2.70.98.10
3a24A	3a24A02_3a24A03	3.20.20.70	2.60.40.1180
3a9sA	3a9sA01_3a9sA02	3.40.50.1070	3.40.275.10
3a9sA	3a9sA03_3a9sA01	3.20.14.10	3.40.50.1070
3a9sA	3a9sA03_3a9sA02	3.20.14.10	3.40.275.10
3abgA	3abgA01_3abgA03	2.60.40.420	2.60.40.420
3abgA	3abgA02_3abgA01	2.60.40.420	2.60.40.420
3abgA	3abgA02_3abgA03	2.60.40.420	2.60.40.420
3abqC	3abqC02_3abqC01	1.10.220.70	2.30.170.30
3abqC	3abqC03_3abqC01	3.20.20.70	2.30.170.30
3abqC	3abqC03_3abqC02	3.20.20.70	1.10.220.70
3apoA	3apoA02_3apoA03	3.40.30.10	3.40.30.10
3apoA	3apoA05_3apoA04	3.40.30.10	3.40.30.10
3asiA	3asiA03_3asiA02	2.60.120.200	2.10.25.10
3au4A	3au4A03_3au4A02	1.20.80.10	3.10.20.90
3au4A	3au4A03_3au4A04	1.20.80.10	2.30.29.30
3b0xA	3b0xA01_3b0xA02	1.10.150.110	1.10.150.20
3b0xA	3b0xA05_3b0xA04	3.20.20.140	3.30.210.10
3b34A	3b34A05_3b34A03	1.25.50.10	1.10.390.10
3bgaB	3bgaB01_3bgaB02	2.60.120.260	2.60.40.10

3bgaB	3bgaB03_3bgaB01	3.20.20.80	2.60.120.260
3bgaB	3bgaB03_3bgaB02	3.20.20.80	2.60.40.10
3bgaB	3bgaB03_3bgaB04	3.20.20.80	2.60.40.10
3bgaB	3bgaB03_3bgaB05	3.20.20.80	2.70.98.10
3c0kA	3c0kA02_3c0kA01	3.30.750.80	2.30.130.10
3c0kA	3c0kA03_3c0kA02	3.40.50.150	3.30.750.80
3c18C	3c18C02_3c18C01	1.20.120.330	3.30.460.10
3c18C	3c18C02_3c18C03	1.20.120.330	1.10.10.10
3c1dA	3c1dA02_3c1dA03	1.10.10.10	1.10.10.10
3c6kC	3c6kC03_3c6kC02	3.40.50.150	2.30.140.10
3c8tA	3c8tA02_3c8tA01	1.20.200.10	1.10.275.10
3c8tA	3c8tA02_3c8tA03	1.20.200.10	1.10.40.30
3cb2A	3cb2A01_3cb2A02	3.40.50.1440	3.30.1330.20
3cb2A	3cb2A01_3cb2A03	3.40.50.1440	1.10.287.600
3cb2A	3cb2A02_3cb2A03	3.30.1330.20	1.10.287.600
3cblA	3cblA03_3cblA02	1.10.510.10	3.30.200.20
3cmgA	3cmgA01_3cmgA02	2.60.120.260	2.60.40.10
3cmgA	3cmgA03_3cmgA01	3.20.20.80	2.60.120.260
3cmgA	3cmgA03_3cmgA02	3.20.20.80	2.60.40.10
3cmgA	3cmgA03_3cmgA04	3.20.20.80	2.60.40.10
3cw9B	3cw9B01_3cw9B02	3.40.50.980	3.40.50.980
3cw9B	3cw9B01_3cw9B03	3.40.50.980	2.30.38.10
3cw9B	3cw9B01_3cw9B04	3.40.50.980	3.30.300.30
3cw9B	3cw9B02_3cw9B03	3.40.50.980	2.30.38.10
3dfgA	3dfgA03_3dfgA02	1.10.10.10	1.10.10.10
3dzdB	3dzdB02_3dzdB03	3.40.50.300	1.10.8.60
3ebhA	3ebhA01_3ebhA03	2.60.40.1730	1.10.390.10
3ebhA	3ebhA05_3ebhA03	1.25.50.10	1.10.390.10
3edfB	3edfB02_3edfB01	3.20.20.80	2.60.40.10
3edfB	3edfB02_3edfB03	3.20.20.80	2.60.40.1180
3ejjX	3ejjX02_3ejjX01	2.60.40.10	2.60.40.10
3erbA	3erbA02_3erbA03	2.10.70.10	2.10.70.10
3eyaG	3eyaG03_3eyaG01	3.40.50.970	3.40.50.970
3eyaG	3eyaG03_3eyaG02	3.40.50.970	3.40.50.1220
3f1sB	3f1sB03_3f1sB01	2.40.10.10	2.10.25.10
3f1sB	3f1sB03_3f1sB02	2.40.10.10	2.40.10.10
3f70A	3f70A02_3f70A04	2.30.30.140	2.30.30.140
3f70A	3f70A03_3f70A02	2.30.30.140	2.30.30.140
3fl7A	3fl7A01_3fl7A02	2.60.120.260	2.60.40.1770
3fl7A	3fl7A03_3fl7A02	2.10.50.10	2.60.40.1770
3fl7A	3fl7A04_3fl7A03	2.60.40.10	2.10.50.10
3gcmB	3gcmB03_3gcmB02	3.30.230.70	1.10.10.400
3gdbA	3gdbA01_3gdbA02	3.20.20.80	2.60.120.260
3gdbA	3gdbA01_3gdbA03	3.20.20.80	2.60.40.10
3gdbA	3gdbA02_3gdbA03	2.60.120.260	2.60.40.10
3gg2A	3gg2A01_3gg2A02	3.40.50.720	1.20.5.100
3gg2A	3gg2A03_3gg2A01	3.40.50.720	3.40.50.720
3gg2A	3gg2A03_3gg2A02	3.40.50.720	1.20.5.100
3gm8A	3gm8A03_3gm8A01	3.20.20.80	2.60.120.260
3gm8A	3gm8A03_3gm8A02	3.20.20.80	2.60.40.10
3gm8A	3gm8A03_3gm8A04	3.20.20.80	2.60.40.10
3hi7A	3hi7A02_3hi7A01	3.10.450.40	3.10.450.40
3hi7A	3hi7A03_3hi7A01	2.70.98.20	3.10.450.40

3hi7A	3hi7A03_3hi7A02	2.70.98.20	3.10.450.40
3hn7A	3hn7A02_3hn7A01	3.40.1190.10	3.40.50.720
3hn7A	3hn7A02_3hn7A03	3.40.1190.10	3.90.190.20
3hn7A	3hn7A03_3hn7A01	3.90.190.20	3.40.50.720
3hwcD	3hwcD01_3hwcD02	1.10.3140.10	2.40.110.10
3hwcD	3hwcD03_3hwcD01	1.20.140.10	1.10.3140.10
3hwcD	3hwcD03_3hwcD02	1.20.140.10	2.40.110.10
3i99A	3i99A02_3i99A01	3.30.465.10	3.30.43.10
3i99A	3i99A03_3i99A01	3.90.78.10	3.30.43.10
3ialA	3ialA01_3ialA02	3.30.930.10	3.40.50.800
3ialA	3ialA01_3ialA03	3.30.930.10	3.30.110.30
3iecD	3iecD01_3iecD03	3.30.200.20	1.10.8.10
3iecD	3iecD02_3iecD01	1.10.510.10	3.30.200.20
3is0X	3is0X01_3is0X02	2.60.40.1280	2.60.40.1290
3is0X	3is0X01_3is0X03	2.60.40.1280	2.60.40.10
3js8A	3js8A02_3js8A01	3.30.465.10	3.30.43.10
3js8A	3js8A03_3js8A01	3.40.462.10	3.30.43.10
3js8A	3js8A03_3js8A02	3.40.462.10	3.30.465.10
3js8A	3js8A04_3js8A01	1.10.45.10	3.30.43.10
3js8A	3js8A04_3js8A02	1.10.45.10	3.30.465.10
3js8A	3js8A04_3js8A03	1.10.45.10	3.40.462.10
3k5iA	3k5iA01_3k5iA02	3.40.50.20	3.30.1490.20
3k5iA	3k5iA03_3k5iA01	3.30.470.20	3.40.50.20
3k5iA	3k5iA03_3k5iA02	3.30.470.20	3.30.1490.20
3komA	3komA01_3komA02	3.40.50.970	3.40.50.970
3l4yA	3l4yA02_3l4yA01	2.60.40.1760	4.10.110.10
3l4yA	3l4yA02_3l4yA04	2.60.40.1760	2.60.40.1180
3l4yA	3l4yA03_3l4yA02	3.20.20.80	2.60.40.1760
3l4yA	3l4yA03_3l4yA04	3.20.20.80	2.60.40.1180
3l4yA	3l4yA03_3l4yA05	3.20.20.80	2.60.40.1180
3l4yA	3l4yA05_3l4yA04	2.60.40.1180	2.60.40.1180
3l5iA	3l5iA01_3l5iA02	2.60.40.10	2.60.40.10
3l84A	3l84A02_3l84A03	3.40.50.970	3.40.50.920
3lafA	3lafA01_3lafA04	2.60.40.10	2.60.40.10
3lafA	3lafA02_3lafA03	2.60.40.10	2.60.40.10
3lidA	3lidA02_3lidA01	3.30.450.20	1.20.5.170
3lidA	3lidA02_3lidA03	3.30.450.20	3.30.450.20
3lidA	3lidA03_3lidA01	3.30.450.20	1.20.5.170
3lk7A	3lk7A02_3lk7A01	3.40.1190.10	3.40.50.720
3llpA	3llpA01_3llpA02	2.80.10.50	2.80.10.50
3llpA	3llpA02_3llpA04	2.80.10.50	2.80.10.50
3llpA	3llpA03_3llpA02	2.80.10.50	2.80.10.50
3llpA	3llpA03_3llpA04	2.80.10.50	2.80.10.50
3lp8A	3lp8A03_3lp8A01	3.30.470.20	3.40.50.20
3lp8A	3lp8A03_3lp8A04	3.30.470.20	3.90.600.10
3lp8A	3lp8A04_3lp8A01	3.90.600.10	3.40.50.20
3lpfA	3lpfA01_3lpfA02	2.60.120.260	2.60.40.10
3lpfA	3lpfA03_3lpfA01	3.20.20.80	2.60.120.260
3lpfA	3lpfA03_3lpfA02	3.20.20.80	2.60.40.10
3lq2A	3lq2A01_3lq2A02	3.40.50.970	3.40.50.970
3lq2A	3lq2A02_3lq2A03	3.40.50.970	3.40.50.920
3m9vA	3m9vA01_3m9vA02	1.10.540.10	2.40.110.10
3m9vA	3m9vA03_3m9vA01	1.20.140.10	1.10.540.10

3mjgX	3mjgX02_3mjgX01	2.60.40.10	2.60.40.10
3mpiB	3mpiB01_3mpiB02	1.10.540.10	2.40.110.10
3mpiB	3mpiB03_3mpiB01	1.20.140.10	1.10.540.10
3mw9F	3mw9F02_3mw9F01	3.40.50.10860	1.10.287.140
3mw9F	3mw9F03_3mw9F01	3.40.50.720	1.10.287.140
3mw9F	3mw9F03_3mw9F02	3.40.50.720	3.40.50.10860
3n75E	3n75E02_3n75E03	3.40.640.10	3.90.1150.10
3n75E	3n75E02_3n75E04	3.40.640.10	3.90.100.10
3n75E	3n75E03_3n75E04	3.90.1150.10	3.90.100.10
3nb2C	3nb2C02_3nb2C03	1.25.40.300	3.40.1850.10
3nb2C	3nb2C04_3nb2C03	1.10.4140.10	3.40.1850.10
3nf4A	3nf4A01_3nf4A02	1.10.540.10	2.40.110.10
3nf4A	3nf4A03_3nf4A01	1.20.140.10	1.10.540.10
3nvqE	3nvqE01_3nvqE02	2.130.10.10	3.30.1680.10
3nvzB	3nvzB02_3nvzB01	3.30.465.10	3.30.43.10
3nvzB	3nvzB02_3nvzB03	3.30.465.10	3.30.390.50
3ocfA	3ocfA02_3ocfA03	1.20.200.10	1.10.40.30
3oojF	3oojF02_3oojF03	3.40.50.10490	3.40.50.10490
3owaD	3owaD01_3owaD02	1.10.540.10	2.40.110.10
3owaD	3owaD03_3owaD01	1.20.140.10	1.10.540.10
3owaD	3owaD03_3owaD02	1.20.140.10	2.40.110.10
3owaD	3owaD03_3owaD04	1.20.140.10	1.20.140.10
3owaD	3owaD04_3owaD01	1.20.140.10	1.10.540.10
3oyyA	3oyyA02_3oyyA03	2.40.50.140	2.40.50.140
3p26B	3p26B01_3p26B02	3.40.50.300	2.40.30.10
3p26B	3p26B01_3p26B03	3.40.50.300	2.40.30.10
3p26B	3p26B03_3p26B02	2.40.30.10	2.40.30.10
3p4tB	3p4tB02_3p4tB01	2.40.110.10	1.10.540.10
3p4tB	3p4tB03_3p4tB01	1.20.140.10	1.10.540.10
3p4tB	3p4tB03_3p4tB02	1.20.140.10	2.40.110.10
3p7lA	3p7lA01_3p7lA02	3.30.830.10	3.30.830.10
3p7lA	3p7lA01_3p7lA04	3.30.830.10	3.30.830.10
3p7lA	3p7lA02_3p7lA03	3.30.830.10	3.30.830.10
3p7lA	3p7lA03_3p7lA04	3.30.830.10	3.30.830.10
3pfdD	3pfdD02_3pfdD01	2.40.110.10	1.10.540.10
3pfdD	3pfdD03_3pfdD01	1.20.140.10	1.10.540.10
3pzwA	3pzwA01_3pzwA02	2.60.60.20	4.10.375.10
3pzwA	3pzwA02_3pzwA03	4.10.375.10	4.10.372.10
3pzwA	3pzwA04_3pzwA03	3.10.450.60	4.10.372.10
3pzwA	3pzwA05_3pzwA01	1.20.245.10	2.60.60.20
3pzwA	3pzwA05_3pzwA02	1.20.245.10	4.10.375.10
3pzwA	3pzwA05_3pzwA03	1.20.245.10	4.10.372.10
3pzwA	3pzwA05_3pzwA04	1.20.245.10	3.10.450.60
3q9oA	3q9oA01_3q9oA02	2.60.120.200	3.90.1350.10
3q9oA	3q9oA03_3q9oA02	3.90.175.10	3.90.1350.10
3r6qG	3r6qG02_3r6qG03	1.20.200.10	1.10.40.30
3rh4A	3rh4A01_3rh4A02	1.10.150.110	1.10.150.20
3rh4A	3rh4A03_3rh4A02	3.30.460.10	1.10.150.20
3rh4A	3rh4A03_3rh4A04	3.30.460.10	3.30.210.10
3s98A	3s98A02_3s98A03	2.60.40.10	2.60.40.10
3sqgA	3sqgA03_3sqgA01	1.20.840.10	3.90.390.10
3sqrA	3sqrA02_3sqrA01	2.60.40.420	2.60.40.420
3sqrA	3sqrA03_3sqrA01	2.60.40.420	2.60.40.420

3sqrA	3sqrA03_3sqrA02	2.60.40.420	2.60.40.420
3swoD	3swoD01_3swoD02	1.10.540.10	2.40.110.10
3swoD	3swoD03_3swoD01	1.20.140.10	1.10.540.10
3sxxB	3sxxB03_3sxxB01	2.70.98.20	3.10.450.40
3sxxB	3sxxB03_3sxxB02	2.70.98.20	3.10.450.40
3teyA	3teyA01_3teyA02	3.90.182.10	2.60.120.240
3teyA	3teyA01_3teyA03	3.90.182.10	3.10.20.110
3teyA	3teyA02_3teyA03	2.60.120.240	3.10.20.110
3teyA	3teyA02_3teyA04	2.60.120.240	2.60.40.810
3teyA	3teyA04_3teyA03	2.60.40.810	3.10.20.110
3tk1A	3tk1A02_3tk1A01	3.40.50.300	1.20.5.170
3tk1A	3tk1A02_3tk1A03	3.40.50.300	1.10.287.130
3tv2A	3tv2A02_3tv2A01	1.20.200.10	1.10.275.10
3tv2A	3tv2A02_3tv2A03	1.20.200.10	1.10.40.30
3uemA	3uemA02_3uemA03	3.40.30.10	3.40.30.10
3utoB	3utoB02_3utoB01	3.30.200.20	2.60.40.10
3utoB	3utoB03_3utoB02	1.10.510.10	3.30.200.20
3utoB	3utoB03_3utoB04	1.10.510.10	2.60.40.10
3uw2A	3uw2A01_3uw2A02	3.40.120.10	3.40.120.10
3uw2A	3uw2A01_3uw2A03	3.40.120.10	3.40.120.10
3uw2A	3uw2A03_3uw2A02	3.40.120.10	3.40.120.10
3uw2A	3uw2A03_3uw2A04	3.40.120.10	3.30.310.50
3vmfA	3vmfA01_3vmfA02	3.40.50.300	2.40.30.10
3vmfA	3vmfA01_3vmfA03	3.40.50.300	2.40.30.10
3vmfA	3vmfA03_3vmfA02	2.40.30.10	2.40.30.10
3vmnA	3vmnA02_3vmnA01	3.20.20.80	2.60.40.10
3vmnA	3vmnA02_3vmnA03	3.20.20.80	2.60.40.1180
3vroA	3vroA01_3vroA02	1.20.930.20	1.10.238.10
3vroA	3vroA01_3vroA03	1.20.930.20	3.30.505.10
3vseA	3vseA02_3vseA01	3.30.750.80	2.30.130.10
3vseA	3vseA03_3vseA02	3.40.50.150	3.30.750.80
3wa2X	3wa2X03_3wa2X01	2.70.98.20	3.10.450.40
3wa2X	3wa2X03_3wa2X02	2.70.98.20	3.10.450.40
3wdhA	3wdhA03_3wdhA02	3.20.20.80	2.60.40.10
3wdhA	3wdhA03_3wdhA04	3.20.20.80	2.60.40.1180
3weoA	3weoA01_3weoA03	2.60.40.1760	2.60.40.1180
3weoA	3weoA02_3weoA01	3.20.20.80	2.60.40.1760
3weoA	3weoA02_3weoA03	3.20.20.80	2.60.40.1180
3weoA	3weoA02_3weoA04	3.20.20.80	2.60.40.1180
3weoA	3weoA04_3weoA03	2.60.40.1180	2.60.40.1180
3zh9B	3zh9B03_3zh9B02	1.20.272.10	1.10.8.60
3ziaO	3ziaO02_3ziaO01	3.40.50.300	2.40.10.170
3ziaO	3ziaO02_3ziaO03	3.40.50.300	1.10.1140.10
3ziyA	3ziyA02_3ziyA01	2.60.40.420	2.60.40.420
3ziyA	3ziyA02_3ziyA03	2.60.40.420	1.10.760.10
3zm6A	3zm6A02_3zm6A01	3.40.1190.10	3.40.1390.10
3zm6A	3zm6A02_3zm6A03	3.40.1190.10	3.90.190.20
3zx1A	3zx1A01_3zx1A03	2.60.40.420	2.60.40.420
3zx1A	3zx1A02_3zx1A01	2.60.40.420	2.60.40.420
3zx1A	3zx1A02_3zx1A03	2.60.40.420	2.60.40.420
3zyzA	3zyzA01_3zyzA03	3.20.20.300	2.60.40.10
3zyzA	3zyzA02_3zyzA03	3.40.50.1700	2.60.40.10
4a4aA	4a4aA03_4a4aA02	3.20.20.80	3.30.379.10

4a4aA	4a4aA03_4a4aA04	3.20.20.80	1.20.120.670
4a4aA	4a4aA04_4a4aA01	1.20.120.670	2.60.120.260
4admD	4admD02_4admD03	1.20.200.10	1.10.40.30
4aefB	4aefB01_4aefB02	2.60.40.10	2.60.40.10
4aefB	4aefB03_4aefB01	3.20.20.80	2.60.40.10
4aefB	4aefB03_4aefB02	3.20.20.80	2.60.40.10
4aefB	4aefB03_4aefB04	3.20.20.80	2.60.40.1180
4agsA	4agsA02_4agsA01	1.20.1050.10	3.40.30.10
4agsA	4agsA02_4agsA03	1.20.1050.10	3.40.30.10
4agsA	4agsA04_4agsA03	1.20.1050.10	3.40.30.10
4aioA	4aioA01_4aioA02	2.60.40.1130	2.60.40.10
4aioA	4aioA03_4aioA01	3.20.20.80	2.60.40.1130
4aioA	4aioA03_4aioA02	3.20.20.80	2.60.40.10
4aioA	4aioA03_4aioA04	3.20.20.80	2.60.40.1180
4amwA	4amwA02_4amwA01	3.20.20.80	2.60.40.1760
4amwA	4amwA02_4amwA03	3.20.20.80	2.60.40.1180
4amwA	4amwA02_4amwA04	3.20.20.80	2.60.40.1180
4amwA	4amwA04_4amwA03	2.60.40.1180	2.60.40.1180
4b7lA	4b7lA01_4b7lA02	1.10.418.10	1.10.418.10
4ba0A	4ba0A02_4ba0A01	3.20.20.80	2.60.40.1760
4ba0A	4ba0A02_4ba0A03	3.20.20.80	2.60.40.1180
4ba0A	4ba0A02_4ba0A04	3.20.20.80	2.60.40.1180
4ba0A	4ba0A04_4ba0A03	2.60.40.1180	2.60.40.1180
4bucB	4bucB02_4bucB01	3.40.1190.10	3.40.50.720
4c12A	4c12A02_4c12A01	3.40.1190.10	3.40.1390.10
4c12A	4c12A02_4c12A03	3.40.1190.10	3.90.190.20
4c5eA	4c5eA01_4c5eA04	2.30.30.140	2.30.30.140
4c5eA	4c5eA02_4c5eA04	2.30.30.140	2.30.30.140
4c5eA	4c5eA03_4c5eA02	2.30.30.140	2.30.30.140
4c5eA	4c5eA03_4c5eA04	2.30.30.140	2.30.30.140
4cokA	4cokA03_4cokA01	3.40.50.970	3.40.50.970
4cokA	4cokA03_4cokA02	3.40.50.970	3.40.50.1220
4cvuA	4cvuA03_4cvuA01	3.20.20.80	2.60.120.260
4cvuA	4cvuA03_4cvuA02	3.20.20.80	2.60.40.10
4cvuA	4cvuA03_4cvuA04	3.20.20.80	2.60.40.10
4cvuA	4cvuA03_4cvuA05	3.20.20.80	2.60.40.10
4cvuA	4cvuA04_4cvuA05	2.60.40.10	2.60.40.10
4dcsA	4dcsA01_4dcsA03	3.40.50.300	3.30.300.20
4dcsA	4dcsA02_4dcsA03	3.40.50.300	3.30.300.20
4dmgA	4dmgA02_4dmgA01	3.30.750.80	2.30.130.10
4dmgA	4dmgA03_4dmgA02	3.40.50.150	3.30.750.80
4doyC	4doyC01_4doyC02	1.10.540.10	2.40.110.10
4doyC	4doyC03_4doyC01	1.20.140.10	1.10.540.10
4doyC	4doyC03_4doyC02	1.20.140.10	2.40.110.10
4e1oE	4e1oE02_4e1oE01	3.40.640.10	1.20.1340.10
4e1oE	4e1oE02_4e1oE03	3.40.640.10	3.90.1150.10
4e1oE	4e1oE03_4e1oE01	3.90.1150.10	1.20.1340.10
4e4tB	4e4tB01_4e4tB02	3.40.50.20	3.30.1490.20
4e4tB	4e4tB03_4e4tB01	3.30.470.20	3.40.50.20
4e4tB	4e4tB03_4e4tB02	3.30.470.20	3.30.1490.20
4e9sA	4e9sA02_4e9sA01	2.60.40.420	2.60.40.420
4e9sA	4e9sA03_4e9sA01	2.60.40.420	2.60.40.420
4e9sA	4e9sA03_4e9sA02	2.60.40.420	2.60.40.420

4ejnA	4ejnA01_4ejnA02	2.30.29.30	3.30.200.20
4ejnA	4ejnA03_4ejnA01	1.10.510.10	2.30.29.30
4ejnA	4ejnA03_4ejnA02	1.10.510.10	3.30.200.20
4evfA	4evfA03_4evfA02	1.10.220.10	1.10.220.10
4f3lA	4f3lA02_4f3lA01	3.30.450.20	4.10.280.10
4f3lA	4f3lA03_4f3lA02	3.30.450.20	3.30.450.20
4f3lB	4f3lB02_4f3lB03	3.30.450.20	3.30.450.20
4f5uA	4f5uA01_4f5uA02	1.10.246.10	1.10.246.10
4f5uA	4f5uA01_4f5uA03	1.10.246.10	1.10.246.10
4f5uA	4f5uA03_4f5uA02	1.10.246.10	1.10.246.10
4f5uA	4f5uA03_4f5uA04	1.10.246.10	1.10.246.10
4f5uA	4f5uA05_4f5uA04	1.10.246.10	1.10.246.10
4f5uA	4f5uA05_4f5uA06	1.10.246.10	1.10.246.10
4f7kA	4f7kA01_4f7kA03	2.60.40.420	2.60.40.420
4f7kA	4f7kA02_4f7kA01	2.60.40.420	2.60.40.420
4f7kA	4f7kA02_4f7kA03	2.60.40.420	2.60.40.420
4fl2A	4fl2A03_4fl2A02	3.30.505.10	1.10.930.10
4fl2A	4fl2A05_4fl2A01	1.10.510.10	3.30.505.10
4fnqA	4fnqA02_4fnqA01	3.20.20.70	2.70.98.60
4fnqA	4fnqA02_4fnqA03	3.20.20.70	2.60.40.1180
4g1vA	4g1vA01_4g1vA03	1.10.490.10	3.40.50.80
4g1vA	4g1vA03_4g1vA02	3.40.50.80	2.40.30.10
4gafB	4gafB02_4gafB01	2.60.40.10	2.60.40.10
4ghgA	4ghgA01_4ghgA02	3.10.180.10	3.10.180.10
4ghgA	4ghgA01_4ghgA03	3.10.180.10	4.10.1270.10
4ghgA	4ghgA02_4ghgA03	3.10.180.10	4.10.1270.10
4gwmA	4gwmA01_4gwmA03	3.40.390.10	2.60.210.10
4gwmA	4gwmA03_4gwmA02	2.60.210.10	2.60.120.200
4hr3A	4hr3A01_4hr3A02	1.10.540.10	2.40.110.10
4hr3A	4hr3A03_4hr3A01	1.20.140.10	1.10.540.10
4hr3A	4hr3A03_4hr3A02	1.20.140.10	2.40.110.10
4hv4A	4hv4A02_4hv4A01	3.40.1190.10	3.40.50.720
4hv4A	4hv4A02_4hv4A03	3.40.1190.10	3.90.190.20
4hvcA	4hvcA01_4hvcA03	3.30.930.10	3.30.110.30
4i2aA	4i2aA01_4i2aA04	1.10.150.110	3.30.210.10
4i2aA	4i2aA03_4i2aA02	3.30.460.10	1.10.150.20
4i2aA	4i2aA03_4i2aA04	3.30.460.10	3.30.210.10
4i2xE	4i2xE02_4i2xE03	2.60.40.10	2.60.40.10
4i4tB	4i4tB01_4i4tB02	3.40.50.1440	3.30.1330.20
4i4tB	4i4tB01_4i4tB03	3.40.50.1440	1.10.287.600
4i4tB	4i4tB02_4i4tB03	3.30.1330.20	1.10.287.600
4ic6C	4ic6C02_4ic6C01	2.40.10.10	2.40.10.10
4ic6C	4ic6C02_4ic6C03	2.40.10.10	2.30.42.10
4iihB	4iihB01_4iihB02	3.20.20.300	3.40.50.1700
4iihB	4iihB01_4iihB04	3.20.20.300	2.60.40.10
4iihB	4iihB02_4iihB04	3.40.50.1700	2.60.40.10
4im0A	4im0A02_4im0A01	1.10.510.10	3.30.200.20
4iv6B	4iv6B01_4iv6B02	1.10.540.10	2.40.110.10
4iv6B	4iv6B03_4iv6B01	1.20.140.10	1.10.540.10
4iv6B	4iv6B03_4iv6B02	1.20.140.10	2.40.110.10
4j7rA	4j7rA02_4j7rA01	3.20.20.80	2.60.40.10
4j7rA	4j7rA02_4j7rA03	3.20.20.80	2.60.40.1180
4jawA	4jawA02_4jawA01	3.20.20.80	3.30.379.10

4jawA	4jawA02_4jawA03	3.20.20.80	2.80.10.50
4jb1A	4jb1A02_4jb1A01	3.30.465.10	3.30.43.10
4jb1A	4jb1A03_4jb1A01	3.90.78.10	3.30.43.10
4jklA	4jklA01_4jklA02	2.60.120.260	2.60.40.10
4jklA	4jklA03_4jklA02	3.20.20.80	2.60.40.10
4jkmB	4jkmB01_4jkmB02	2.60.120.260	2.60.40.10
4jkmB	4jkmB03_4jkmB02	3.20.20.80	2.60.40.10
4jpsA	4jpsA05_4jpsA04	1.10.1070.11	3.30.1010.10
4k9qB	4k9qB01_4k9qB02	3.40.50.970	3.40.50.1220
4k9qB	4k9qB03_4k9qB01	3.40.50.970	3.40.50.970
4k9qB	4k9qB03_4k9qB02	3.40.50.970	3.40.50.1220
4kmqA	4kmqA02_4kmqA01	2.60.40.1760	2.60.40.10
4kmqA	4kmqA03_4kmqA02	3.20.20.80	2.60.40.1760
4kmqA	4kmqA03_4kmqA04	3.20.20.80	2.60.40.1180
4kmqA	4kmqA03_4kmqA05	3.20.20.80	2.60.40.1180
4kmqA	4kmqA05_4kmqA04	2.60.40.1180	2.60.40.1180
4kmqA	4kmqA05_4kmqA06	2.60.40.1180	2.60.120.260
4ko8A	4ko8A01_4ko8A02	2.40.40.20	3.10.330.10
4kxwA	4kxwA01_4kxwA02	3.40.50.970	3.40.50.970
4kxwA	4kxwA02_4kxwA03	3.40.50.970	3.40.50.920
4l1fB	4l1fB01_4l1fB02	1.10.540.10	2.40.110.10
4l1fB	4l1fB03_4l1fB01	1.20.140.10	1.10.540.10
4m9aA	4m9aA01_4m9aA02	1.10.540.10	2.40.110.10
4m9aA	4m9aA03_4m9aA02	1.20.140.10	2.40.110.10
4mamA	4mamA03_4mamA01	3.30.470.20	3.40.50.20
4mamA	4mamA03_4mamA02	3.30.470.20	3.30.1490.20
4mb4A	4mb4A01_4mb4A02	3.20.20.80	2.60.40.10
4mduA	4mduA03_4mduA02	1.10.220.10	1.10.220.10
4nwgB	4nwgB03_4nwgB01	3.90.190.10	3.30.505.10
4o5mC	4o5mC01_4o5mC02	1.10.540.10	2.40.110.10
4o5mC	4o5mC03_4o5mC01	1.20.140.10	1.10.540.10
4o5mC	4o5mC03_4o5mC02	1.20.140.10	2.40.110.10
4ogzA	4ogzA02_4ogzA01	3.20.20.70	2.60.40.10
4ogzA	4ogzA02_4ogzA03	3.20.20.70	2.60.40.1180
4psrB	4psrB01_4psrB02	3.20.20.80	2.60.40.1180
4qhpA	4qhpA01_4qhpA03	2.60.40.1730	1.10.390.10
4qhpA	4qhpA05_4qhpA03	1.25.50.10	1.10.390.10
4tr7A	4tr7A02_4tr7A01	3.10.150.10	3.10.150.10
4tr7A	4tr7A03_4tr7A02	3.10.150.10	3.10.150.10
4tr8B	4tr8B02_4tr8B01	3.10.150.10	3.10.150.10
4tr8B	4tr8B02_4tr8B03	3.10.150.10	3.10.150.10
5reqD	5reqD01_5reqD02	3.20.20.240	3.40.50.280

512 consecutive discontinuous domain dataset (domain-CD-2)			
**PDB ID**	**Domain_pair**	**Domain 1 CATH classifcation**	**Domain 2 CATH classification**
3qvsA	3qvsA01_3qvsA02	3.40.50.720	3.30.360.10
3sqzA	3sqzA01_3sqzA02	3.40.47.10	3.40.47.10
2j91B	2j91B01_2j91B02	1.10.275.10	1.20.200.10
2olgA	2olgA01_2olgA02	2.40.10.10	2.40.10.10
1n9gD	1n9gD01_1n9gD02	3.90.180.10	3.40.50.720
2p8bA	2p8bA01_2p8bA02	3.30.390.10	3.20.20.120
2zyjB	2zyjB01_2zyjB02	3.90.1150.10	3.40.640.10
1enfA	1enfA01_1enfA02	2.40.50.110	3.10.20.120
4hwgA	4hwgA01_4hwgA02	3.40.50.2000	3.40.50.2000
2yhwA	2yhwA01_2yhwA02	3.30.420.40	3.30.420.40
3dr3A	3dr3A01_3dr3A02	3.40.50.720	3.30.360.10
1zodA	1zodA01_1zodA02	3.90.1150.10	3.40.640.10
2j6lD	2j6lD01_2j6lD02	3.40.605.10	3.40.309.10
3ke3A	3ke3A01_3ke3A02	3.90.1150.10	3.40.640.10
1sefA	1sefA01_1sefA02	2.60.120.10	2.60.120.10
1mkiA	1mkiA01_1mkiA02	3.40.710.10	1.10.1500.10
4jn7A	4jn7A01_4jn7A02	3.30.390.10	3.20.20.120
2c43A	2c43A01_2c43A02	3.90.470.20	3.90.470.20
3qldA	3qldA01_3qldA02	3.30.390.10	3.20.20.120
2r42A	2r42A01_2r42A02	3.30.230.10	3.30.70.890
1ty0B	1ty0B01_1ty0B02	3.10.20.120	2.40.50.110
4gaxA	4gaxA01_4gaxA02	1.10.600.10	1.50.10.130
2arrA	2arrA01_2arrA02	3.30.497.10	2.30.39.10
3i44A	3i44A01_3i44A02	3.40.605.10	3.40.309.10
2y1vB	2y1vB02_2y1vB03	2.60.40.740	2.60.40.1140
2bmoA	2bmoA01_2bmoA02	3.90.380.10	2.102.10.10
2cdcD	2cdcD01_2cdcD02	3.90.180.10	3.40.50.720
3b6zA	3b6zA01_3b6zA02	3.90.180.10	3.40.50.720
1bjwA	1bjwA01_1bjwA02	3.90.1150.10	3.40.640.10
1cshA	1cshA01_1cshA02	1.10.580.10	1.10.230.10
3vc5A	3vc5A01_3vc5A02	3.30.390.10	3.20.20.120
1x87B	1x87B01_1x87B02	3.40.1770.10	3.40.50.10730
3nclA	3nclA01_3nclA02	2.40.10.10	2.40.10.10
1exnA	1exnA01_1exnA02	1.10.150.20	3.40.50.1010
3jz6B	3jz6B01_3jz6B02	3.30.470.10	3.20.10.10
3nvsA	3nvsA01_3nvsA02	3.65.10.10	3.65.10.10
1y8zA	1y8zA01_1y8zA02	3.40.50.2000	3.40.50.2000
1te2A	1te2A01_1te2A02	3.40.50.1000	1.10.150.240
3t7dA	3t7dA01_3t7dA02	3.40.50.2000	3.40.50.2000
3g68B	3g68B01_3g68B02	3.40.50.10490	3.40.50.10490
3ziuA	3ziuA01_3ziuA02	1.10.730.10	3.40.50.620
3l8aB	3l8aB01_3l8aB02	3.90.1150.10	3.40.640.10
1iyeA	1iyeA01_1iyeA02	3.30.470.10	3.20.10.10
4bvaA	4bvaA01_4bvaA02	3.30.1780.10	3.40.50.720
1yr2A	1yr2A01_1yr2A02	3.40.50.1820	2.130.10.120
3vvfB	3vvfB01_3vvfB02	3.40.190.10	3.40.190.10
3widB	3widB01_3widB02	3.90.180.10	3.40.50.720
2nttA	2nttA01_2nttA02	3.10.20.120	2.40.50.110
3lq6B	3lq6B01_3lq6B02	2.40.510.10	2.40.30.120
4n07B	4n07B01_4n07B02	3.40.190.10	3.40.190.10
4eclC	4eclC01_4eclC02	2.40.37.10	3.20.20.10
3h7fB	3h7fB01_3h7fB02	3.90.1150.10	3.40.640.10
4e9xB	4e9xB01_4e9xB02	2.60.40.420	2.60.40.420
3stpA	3stpA01_3stpA02	3.30.390.10	3.20.20.120

4d9bB	4d9bB01_4d9bB02	3.40.50.1100	3.40.50.1100
2h3hB	2h3hB01_2h3hB02	3.40.50.2300	3.40.50.2300
1nr0A	1nr0A01_1nr0A02	2.130.10.10	2.130.10.10
2oayA	2oayA01_2oayA02	3.30.497.10	2.30.39.10
3pfgA	3pfgA01_3pfgA02	3.40.50.150	2.20.130.10
3dzoA	3dzoA01_3dzoA02	1.10.510.10	3.30.200.20
3c2eA	3c2eA01_3c2eA02	3.90.1170.20	3.20.20.70
2gb3C	2gb3C01_2gb3C02	3.90.1150.10	3.40.640.10
4fqdA	4fqdA01_4fqdA02	3.65.10.10	3.65.10.10
3p5pA	3p5pA01_3p5pA03	1.50.10.130	1.10.600.10
3qguB	3qguB01_3qguB02	3.90.1150.10	3.40.640.10
4dngA	4dngA01_4dngA02	3.40.605.10	3.40.309.10
2epjA	2epjA01_2epjA02	3.90.1150.10	3.40.640.10
4lv8A	4lv8A01_4lv8A02	1.10.510.10	3.30.200.20
2zc0D	2zc0D01_2zc0D02	3.90.1150.10	3.40.640.10
4nogB	4nogB01_4nogB02	3.90.1150.10	3.40.640.10
2xe4A	2xe4A01_2xe4A02	3.40.50.1820	2.130.10.120
1x7dA	1x7dA01_1x7dA02	3.30.1780.10	3.40.50.720
3go2A	3go2A01_3go2A02	3.30.390.10	3.20.20.120
1moqA	1moqA01_1moqA02	3.40.50.10490	3.40.50.10490
2ac1A	2ac1A01_2ac1A02	2.115.10.20	2.60.120.560
1itxA	1itxA01_1itxA02	3.20.20.80	3.10.50.10
3tj4A	3tj4A01_3tj4A02	3.30.390.10	3.20.20.120
3kd3B	3kd3B01_3kd3B02	3.40.50.1000	1.10.150.210
1r1hA	1r1hA01_1r1hA02	3.40.390.10	1.10.1380.10
2p3eA	2p3eA01_2p3eA02	2.40.37.10	3.20.20.10
4dccA	4dccA01_4dccA02	3.40.50.1000	1.10.150.240
3rotA	3rotA01_3rotA02	3.40.50.2300	3.40.50.2300
1qr0A	1qr0A01_1qr0A02	3.90.470.20	3.90.470.20
3bc9A	3bc9A02_3bc9A04	3.20.20.80	2.60.40.1180
3qm2B	3qm2B01_3qm2B02	3.90.1150.10	3.40.640.10
4l3tB	4l3tB01_4l3tB03	3.30.830.10	3.30.830.10
4itbB	4itbB01_4itbB02	3.40.605.10	3.40.309.10
2ps2A	2ps2A01_2ps2A02	3.30.390.10	3.20.20.120
1d2fA	1d2fA01_1d2fA02	3.90.1150.10	3.40.640.10
2ixaA	2ixaA01_2ixaA02	3.40.50.720	3.30.360.10
1xruA	1xruA01_1xruA02	2.60.120.520	2.60.120.10
3dwgA	3dwgA01_3dwgA02	3.40.50.1100	3.40.50.1100
2gl5B	2gl5B01_2gl5B02	3.30.390.10	3.20.20.120
1gqgC	1gqgC01_1gqgC02	2.60.120.10	2.60.120.10
3mfyA	3mfyA01_3mfyA02	3.40.50.300	2.40.50.100
4h27A	4h27A01_4h27A02	3.40.50.1100	3.40.50.1100
3b46B	3b46B01_3b46B02	3.90.1150.10	3.40.640.10
3q2iA	3q2iA01_3q2iA02	3.40.50.720	3.30.360.10
1m5sC	1m5sC01_1m5sC02	3.30.70.520	3.30.70.520
4ao9B	4ao9B01_4ao9B02	3.90.1150.10	3.40.640.10
3fm3A	3fm3A01_3fm3A02	3.90.230.10	1.10.10.10
3ozyA	3ozyA01_3ozyA02	3.30.390.10	3.20.20.120
1j58A	1j58A01_1j58A02	2.60.120.10	2.60.120.10
2j5zA	2j5zA01_2j5zA02	3.90.215.10	4.10.530.10
2b9lA	2b9lA01_2b9lA03	2.40.10.10	2.40.10.10
4k8lA	4k8lA01_4k8lA02	3.10.310.10	3.10.310.10
3ednB	3ednB01_3ednB02	3.10.310.10	3.10.310.10
1xgsA	1xgsA01_1xgsA02	3.90.230.10	1.10.10.10
2q49B	2q49B01_2q49B02	3.40.50.720	3.30.360.10
4h7nC	4h7nC01_4h7nC02	3.40.605.10	3.40.309.10
3bnjA	3bnjA01_3bnjA02	1.10.1130.10	1.20.140.10

4hvkA	4hvkA01_4hvkA02	3.90.1150.10	3.40.640.10
1wuuA	1wuuA01_1wuuA02	3.30.230.10	3.30.70.890
3uykA	3uykA01_3uykA02	3.40.50.2000	3.40.50.2000
4wlhB	4wlhB01_4wlhB02	3.90.1150.10	3.40.640.10
1v7cA	1v7cA01_1v7cA02	3.40.50.1100	3.40.50.1100
1hbnB	1hbnB01_1hbnB02	3.30.70.470	1.20.840.10
4qtoB	4qtoB01_4qtoB02	3.40.605.10	3.40.309.10
2qjvB	2qjvB01_2qjvB02	2.60.120.10	2.60.120.10
3kgwA	3kgwA01_3kgwA02	3.90.1150.10	3.40.640.10
2bklA	2bklA01_2bklA02	3.40.50.1820	2.130.10.120
1a59A	1a59A01_1a59A02	1.10.580.10	1.10.230.10
1sjdD	1sjdD01_1sjdD02	3.30.390.10	3.20.20.120
3e1iE	3e1iE01_3e1iE02	3.90.215.10	4.10.530.10
1o04G	1o04G01_1o04G02	3.40.605.10	3.40.309.10
4k2mA	4k2mA01_4k2mA02	3.40.640.10	3.90.1150.10
3n2oB	3n2oB01_3n2oB02	2.40.37.10	3.20.20.10
3ek1A	3ek1A01_3ek1A02	3.40.605.10	3.40.309.10
1ve5A	1ve5A01_1ve5A02	3.40.50.1100	3.40.50.1100
4npiD	4npiD01_4npiD02	3.40.605.10	3.40.309.10
3oenA	3oenA01_3oenA02	3.40.190.10	3.40.190.10
3rbtD	3rbtD01_3rbtD02	3.40.30.10	1.20.1050.10
3i4jB	3i4jB01_3i4jB02	3.90.1150.10	3.40.640.10
2j3hB	2j3hB01_2j3hB02	3.90.180.10	3.40.50.720
2igtA	2igtA01_2igtA02	3.40.50.150	2.60.40.1180
2higA	2higA02_2higA03	3.40.50.450	3.40.50.460
2vrqC	2vrqC01_2vrqC02	2.60.40.1180	3.20.20.80
3g7qA	3g7qA01_3g7qA02	3.90.1150.10	3.40.640.10
4a27A	4a27A01_4a27A02	3.90.180.10	3.40.50.720
3v2uA	3v2uA01_3v2uA02	3.40.50.720	3.30.360.10
2dm6B	2dm6B01_2dm6B02	3.90.180.10	3.40.50.720
4lw2A	4lw2A01_4lw2A02	3.90.1150.10	3.40.640.10
4aw2A	4aw2A01_4aw2A02	3.30.200.20	1.10.510.10
1qq5A	1qq5A01_1qq5A02	3.40.50.1000	1.10.150.240
4nesA	4nesA01_4nesA02	3.40.50.2000	3.40.50.2000
1pieA	1pieA01_1pieA02	3.30.230.10	3.30.70.890
3m5qA	3m5qA01_3m5qA02	1.10.520.10	1.10.420.10
3f9mA	3f9mA01_3f9mA02	3.40.367.20	3.30.420.40
1y3tA	1y3tA01_1y3tA02	2.60.120.10	2.60.120.10
4ofxA	4ofxA01_4ofxA02	3.40.50.1100	3.40.50.1100
3pplA	3pplA01_3pplA02	3.90.1150.10	3.40.640.10
1yi8C	1yi8C01_1yi8C02	3.40.50.620	1.10.240.10
3ingA	3ingA01_3ingA02	3.40.50.720	3.30.360.10
1rcqA	1rcqA01_1rcqA02	2.40.37.10	3.20.20.10
3warA	3warA01_3warA02	1.10.510.10	3.30.200.20
2f8mA	2f8mA01_2f8mA02	3.40.50.1360	3.30.70.260
4iymC	4iymC01_4iymC02	3.40.605.10	3.40.309.10
3qagA	3qagA01_3qagA02	3.40.30.10	1.20.1050.10
3nytA	3nytA01_3nytA02	3.40.640.10	3.90.1150.10
4e77A	4e77A01_4e77A02	3.90.1150.10	3.40.640.10
3iauA	3iauA01_3iauA02	3.40.50.1100	3.40.50.1100
4eojB	4eojB01_4eojB02	1.10.472.10	1.10.472.10
4j9wB	4j9wB01_4j9wB02	3.10.310.10	3.10.310.10
4kv7A	4kv7A01_4kv7A02	3.40.50.2300	3.40.50.2300
3gh5A	3gh5A01_3gh5A02	3.20.20.80	3.30.379.10
1ebfB	1ebfB01_1ebfB02	3.40.50.720	3.30.360.10
4i3vH	4i3vH01_4i3vH02	3.40.605.10	3.40.309.10
1c7nE	1c7nE01_1c7nE02	3.90.1150.10	3.40.640.10

1g55A	1g55A01_1g55A02	3.90.120.10	3.40.50.150
4l22A	4l22A01_4l22A02	3.40.50.2000	3.40.50.2000
3g6mA	3g6mA01_3g6mA02	3.20.20.80	3.10.50.10
2ez2B	2ez2B01_2ez2B02	3.90.1150.10	3.40.640.10
1s7jA	1s7jA01_1s7jA02	3.10.310.10	3.10.310.10
4c3xD	4c3xD01_4c3xD02	3.50.50.60	3.90.700.10
3vsvB	3vsvB01_3vsvB02	2.160.20.10	2.60.40.1180
1eg5A	1eg5A01_1eg5A02	3.90.1150.10	3.40.640.10
3mc0D	3mc0D01_3mc0D02	3.10.20.120	2.40.50.110
3hdlA	3hdlA01_3hdlA02	1.10.520.10	1.10.420.10
3ju8A	3ju8A01_3ju8A02	3.40.605.10	3.40.309.10
3g7dA	3g7dA02_3g7dA03	2.60.120.10	2.60.120.10
2gz1B	2gz1B01_2gz1B02	3.40.50.720	3.30.360.10
3gjuA	3gjuA01_3gjuA02	3.90.1150.10	3.40.640.10
1ewfA	1ewfA01_1ewfA02	3.15.10.10	3.15.20.10
2ovlA	2ovlA01_2ovlA02	3.30.390.10	3.20.20.120
3crmA	3crmA01_3crmA02	3.40.50.300	1.10.287.890
3nx4A	3nx4A01_3nx4A02	3.90.180.10	3.40.50.720
3eafA	3eafA01_3eafA02	3.40.50.2300	3.40.50.2300
4kctB	4kctB01_4kctB02	3.40.1380.20	3.20.20.60
1hxpA	1hxpA01_1hxpA02	3.30.428.10	3.30.428.10
3cq4A	3cq4A01_3cq4A02	3.90.1150.10	3.40.640.10
4f3xA	4f3xA01_4f3xA02	3.40.605.10	3.40.309.10
1jmkO	1jmkO01_1jmkO02	3.40.50.1820	1.10.287.490
4hacA	4hacA01_4hacA02	3.30.230.10	3.30.70.890
3islA	3islA01_3islA02	3.90.1150.10	3.40.640.10
2podB	2podB01_2podB02	3.30.390.10	3.20.20.120
3fdbA	3fdbA01_3fdbA02	3.90.1150.10	3.40.640.10
2dr1A	2dr1A01_2dr1A02	3.90.1150.10	3.40.640.10
3b4wA	3b4wA01_3b4wA02	3.40.605.10	3.40.309.10
3rplB	3rplB01_3rplB02	3.30.540.10	3.40.190.90
4ht3B	4ht3B01_4ht3B02	3.40.50.1100	3.40.50.1100
2ov9C	2ov9C01_2ov9C02	3.10.129.10	1.20.58.350
2hp0B	2hp0B01_2hp0B02	1.10.4100.10	3.30.1330.120
3i45A	3i45A01_3i45A02	3.40.50.2300	3.40.50.2300
2w8nA	2w8nA01_2w8nA02	3.40.605.10	3.40.309.10
1wkvB	1wkvB02_1wkvB03	3.40.50.1100	3.40.50.1100
3l7oB	3l7oB01_3l7oB02	3.40.50.1360	3.30.70.260
1x1oA	1x1oA01_1x1oA02	3.90.1170.20	3.20.20.70
3rivA	3rivA01_3rivA02	1.10.520.10	1.10.420.10
4ap9A	4ap9A01_4ap9A02	3.40.50.1000	1.10.150.210
4wf7C	4wf7C01_4wf7C02	3.20.20.80	3.90.400.10
4nzcA	4nzcA01_4nzcA02	3.20.20.80	3.10.50.10
1fonA	1fonA01_1fonA02	2.40.10.10	2.40.10.10
3jtxA	3jtxA01_3jtxA02	3.90.1150.10	3.40.640.10
2o0tA	2o0tA01_2o0tA02	2.40.37.10	3.20.20.10
3ondA	3ondA01_3ondA02	3.40.50.1480	3.40.50.720
4k8gA	4k8gA01_4k8gA02	3.30.390.10	3.20.20.120
1js1X	1js1X01_1js1X02	3.40.50.1370	3.40.50.1370
4fdiB	4fdiB01_4fdiB02	3.40.720.10	3.30.1120.10
2og9B	2og9B01_2og9B02	3.30.390.10	3.20.20.120
2v3uA	2v3uA01_2v3uA02	3.40.190.10	3.40.190.10
2vn8A	2vn8A01_2vn8A02	3.90.180.10	3.40.50.720
3mkcA	3mkcA01_3mkcA02	3.30.390.10	3.20.20.120
3gd6A	3gd6A01_3gd6A02	3.30.390.10	3.20.20.120
4lb0B	4lb0B01_4lb0B02	3.10.310.10	3.10.310.10
1v33A	1v33A01_1v33A02	3.90.920.10	1.10.8.160

4thiA	4thiA01_4thiA02	3.40.190.10	3.40.190.10
1j0aA	1j0aA01_1j0aA02	3.40.50.1100	3.40.50.1100
2gn0C	2gn0C01_2gn0C02	3.40.50.1100	3.40.50.1100
4dyeA	4dyeA01_4dyeA02	3.30.390.10	3.20.20.120
1w6kA	1w6kA02_1w6kA03	1.50.10.20	1.50.10.20
1u2kA	1u2kA01_1u2kA02	1.10.520.10	1.10.420.10
2xdwA	2xdwA01_2xdwA02	3.40.50.1820	2.130.10.120
4amuC	4amuC01_4amuC02	3.40.50.1370	3.40.50.1370
3pqaB	3pqaB01_3pqaB02	3.40.605.10	3.40.309.10
1qzyA	1qzyA01_1qzyA02	3.90.230.10	1.10.10.10
1ml4A	1ml4A01_1ml4A02	3.40.50.1370	3.40.50.1370
3s46B	3s46B01_3s46B02	2.40.37.10	3.20.20.10
1w61B	1w61B01_1w61B02	3.10.310.10	3.10.310.10
4i35A	4i35A01_4i35A02	2.150.10.10	3.40.390.10
1vf8A	1vf8A01_1vf8A02	3.20.20.80	3.10.50.10
2bhtA	2bhtA01_2bhtA02	3.40.50.1100	3.40.50.1100
2c0cA	2c0cA01_2c0cA02	3.90.180.10	3.40.50.720
2x5dC	2x5dC01_2x5dC02	3.90.1150.10	3.40.640.10
3gniB	3gniB01_3gniB02	3.30.200.20	1.10.510.10
3msuA	3msuA02_3msuA03	1.10.580.10	1.10.230.10
1wueA	1wueA01_1wueA02	3.30.390.10	3.20.20.120
3dwbA	3dwbA01_3dwbA02	3.40.390.10	1.10.1380.10
4aq4A	4aq4A01_4aq4A02	3.40.190.10	3.40.190.10
3szyA	3szyA01_3szyA02	3.40.720.10	3.30.1360.110
2gj4A	2gj4A01_2gj4A02	3.40.50.2000	3.40.50.2000
3t32A	3t32A01_3t32A02	3.90.1150.10	3.40.640.10
1u08B	1u08B01_1u08B02	3.90.1150.10	3.40.640.10
4dplD	4dplD01_4dplD02	3.40.50.720	3.30.360.10
1uxtA	1uxtA01_1uxtA02	3.40.605.10	3.40.309.10
3a9zB	3a9zB01_3a9zB03	3.90.1150.10	3.40.640.10
6prcC	6prcC01_6prcC02	1.10.468.10	1.10.468.10
4n8dB	4n8dB01_4n8dB02	3.40.50.1820	2.140.10.30
3caiA	3caiA01_3caiA02	3.90.1150.10	3.40.640.10
3cyjC	3cyjC01_3cyjC02	3.30.390.10	3.20.20.120
1w98B	1w98B01_1w98B02	1.10.472.10	1.10.472.10
2c1dA	2c1dA01_2c1dA02	1.10.760.10	1.10.760.10
1sqhA	1sqhA01_1sqhA02	3.40.630.30	3.40.630.30
3f0nA	3f0nA01_3f0nA02	3.30.230.10	3.30.70.890
4e3xB	4e3xB01_4e3xB02	3.40.605.10	3.40.309.10
2bjkA	2bjkA01_2bjkA02	3.40.605.10	3.40.309.10
1ytuA	1ytuA01_1ytuA02	3.30.420.10	3.40.50.2300
2y2wE	2y2wE01_2y2wE02	2.60.40.1180	3.20.20.80
2qdeE	2qdeE01_2qdeE02	3.30.390.10	3.20.20.120
4jf1A	4jf1A01_4jf1A02	3.90.1150.10	3.40.640.10
2y5dA	2y5dA01_2y5dA02	3.40.605.10	3.40.309.10
3piuA	3piuA01_3piuA02	3.90.1150.10	3.40.640.10
2as9A	2as9A01_2as9A02	2.40.10.10	2.40.10.10
4jb3A	4jb3A01_4jb3A02	3.40.50.1000	1.10.150.240
3etfA	3etfA01_3etfA02	3.40.605.10	3.40.309.10
3gazA	3gazA01_3gazA02	3.90.180.10	3.40.50.720
3zveA	3zveA01_3zveA02	2.40.10.10	2.40.10.10
3ekgA	3ekgA01_3ekgA02	3.30.390.10	3.20.20.120
2rc8B	2rc8B01_2rc8B02	3.40.190.10	3.40.190.10
2zj3A	2zj3A01_2zj3A02	3.40.50.10490	3.40.50.10490
1euhD	1euhD01_1euhD02	3.40.605.10	3.40.309.10
3o5cB	3o5cB01_3o5cB02	1.10.760.10	1.10.760.10
1oweA	1oweA01_1oweA02	2.40.10.10	2.40.10.10

4beuA	4beuA01_4beuA02	2.40.37.10	3.20.20.10
2o8lA	2o8lA01_2o8lA02	2.40.10.10	2.40.10.10
1qz9A	1qz9A01_1qz9A02	3.90.1150.10	3.40.640.10
1w1oA	1w1oA02_1w1oA03	3.30.465.10	3.40.462.10
1qw9A	1qw9A01_1qw9A02	2.60.40.1180	3.20.20.80
1uhvA	1uhvA01_1uhvA02	2.60.40.1500	3.20.20.80
1v0wA	1v0wA01_1v0wA02	3.30.870.10	3.30.870.10
3u8oH	3u8oH01_3u8oH02	2.40.10.10	2.40.10.10
4i6vA	4i6vA01_4i6vA02	3.20.20.140	1.10.2020.10
2q4hA	2q4hA01_2q4hA02	3.30.428.10	3.30.428.10
1h32A	1h32A01_1h32A02	1.10.760.10	1.10.760.10
4a8tA	4a8tA01_4a8tA02	3.40.50.1370	3.40.50.1370
3r7fC	3r7fC01_3r7fC02	3.40.50.1370	3.40.50.1370
4aanA	4aanA01_4aanA02	1.10.760.10	1.10.760.10
1fsuA	1fsuA01_1fsuA02	3.40.720.10	3.30.1120.10
1wz9A	1wz9A01_1wz9A02	3.30.497.10	2.30.39.10
3goqA	3goqA01_3goqA02	3.40.50.1400	3.40.50.1400
3iacC	3iacC01_3iacC02	3.20.20.140	1.10.2020.10
3szaB	3szaB01_3szaB02	3.40.605.10	3.40.309.10
2zl7B	2zl7B01_2zl7B02	2.40.510.10	2.40.30.120
4em6A	4em6A01_4em6A02	3.40.50.10490	3.40.50.10490
1yqdA	1yqdA01_1yqdA02	3.90.180.10	3.40.50.720
3cuzA	3cuzA02_3cuzA03	3.20.20.360	1.20.1220.12
1h6dJ	1h6dJ01_1h6dJ02	3.40.50.720	3.30.360.10
2nqlA	2nqlA01_2nqlA02	3.30.390.10	3.20.20.120
3hulA	3hulA01_3hulA02	3.30.230.10	3.30.70.890
1pg2A	1pg2A01_1pg2A02	3.40.50.620	1.10.730.10
4ip7D	4ip7D01_4ip7D02	3.40.1380.20	3.20.20.60
3ug3C	3ug3C01_3ug3C02	2.60.40.1180	3.20.20.80
3frkA	3frkA01_3frkA02	3.40.640.10	3.90.1150.10
2yxxA	2yxxA01_2yxxA02	2.40.37.10	3.20.20.10
2pifA	2pifA01_2pifA02	3.40.1640.10	3.30.2040.10
4dd5A	4dd5A01_4dd5A02	3.40.47.10	3.40.47.10
2vcnA	2vcnA01_2vcnA02	1.10.520.10	1.10.420.10
1qyaB	1qyaB01_1qyaB02	3.10.310.10	3.10.310.10
2q01C	2q01C01_2q01C02	3.20.20.140	1.10.2020.10
1oahA	1oahA01_1oahA02	1.10.1130.10	1.20.140.10
3to7A	3to7A01_3to7A02	3.30.60.60	3.40.630.30
2w70B	2w70B01_2w70B02	3.40.50.20	3.30.470.20
3c7fA	3c7fA01_3c7fA02	2.60.120.260	2.115.10.20
3hheB	3hheB01_3hheB02	3.40.50.1360	3.30.70.260
3n28A	3n28A01_3n28A03	3.40.50.1000	1.10.150.210
4j1oA	4j1oA01_4j1oA02	3.30.390.10	3.20.20.120
3gd5A	3gd5A01_3gd5A02	3.40.50.1370	3.40.50.1370
1jndA	1jndA01_1jndA02	3.20.20.80	3.10.50.10
3ty7A	3ty7A01_3ty7A02	3.40.605.10	3.40.309.10
1zzgB	1zzgB01_1zzgB02	3.40.50.10490	3.40.50.10490
4fp1B	4fp1B01_4fp1B02	3.30.390.10	3.20.20.120
1jixA	1jixA01_1jixA02	3.40.50.2000	3.40.50.2000
2pgwA	2pgwA01_2pgwA02	3.30.390.10	3.20.20.120
2xllB	2xllB01_2xllB03	2.60.40.420	2.60.40.420
2ibpB	2ibpB01_2ibpB02	1.10.580.10	1.10.230.10
2wdcA	2wdcA01_2wdcA03	3.60.21.10	3.90.780.10
3tqgB	3tqgB01_3tqgB02	1.10.580.10	1.10.230.10
1wdkD	1wdkD01_1wdkD02	3.40.47.10	3.40.47.10
1dleA	1dleA01_1dleA02	2.40.10.10	2.40.10.10
3oajB	3oajB01_3oajB02	3.10.180.10	3.10.180.10

3w8zA	3w8zA01_3w8zA02	3.30.43.10	3.30.465.10
2sqcA	2sqcA01_2sqcA02	1.50.10.20	1.50.10.20
3ntlB	3ntlB01_3ntlB02	3.40.50.1240	3.40.50.1240
2pqcA	2pqcA01_2pqcA02	3.65.10.10	3.65.10.10
2qghA	2qghA01_2qghA02	2.40.37.10	3.20.20.10
1mg7B	1mg7B01_1mg7B02	3.30.70.1000	3.30.230.10
2c7rA	2c7rA01_2c7rA02	3.40.50.150	3.90.120.10
4mx2H	4mx2H01_4mx2H02	1.10.275.10	1.20.200.10
3sz3A	3sz3A01_3sz3A02	3.40.50.620	1.10.240.10
3qu5A	3qu5A01_3qu5A02	3.40.50.1000	1.10.150.240
3lg0C	3lg0C01_3lg0C02	3.90.1150.10	3.40.640.10
3dtyE	3dtyE01_3dtyE02	3.40.50.720	3.30.360.10
1vlvA	1vlvA01_1vlvA02	3.40.50.1370	3.40.50.1370
1p1kB	1p1kB01_1p1kB02	3.40.50.720	3.30.360.10
2o0rA	2o0rA01_2o0rA02	3.90.1150.10	3.40.640.10
1tzjB	1tzjB01_1tzjB02	3.40.50.1100	3.40.50.1100
3le2A	3le2A01_3le2A02	3.30.497.10	2.30.39.10
4c4rA	4c4rA01_4c4rA02	3.40.50.1000	1.10.150.240
4my5C	4my5C01_4my5C02	3.90.1150.10	3.40.640.10
3fvvB	3fvvB01_3fvvB02	3.40.50.1000	1.20.1440.100
2hhfA	2hhfA01_2hhfA02	3.30.470.10	3.20.10.10
2rkvA	2rkvA01_2rkvA02	3.30.559.10	3.30.559.10
4eweA	4eweA01_4eweA02	2.60.120.10	2.60.120.10
1dyqA	1dyqA01_1dyqA02	2.40.50.110	3.10.20.120
2vfrA	2vfrA01_2vfrA02	3.30.43.10	3.30.465.10
4nmkC	4nmkC01_4nmkC02	3.40.605.10	3.40.309.10
3dgbA	3dgbA01_3dgbA02	3.30.390.10	3.20.20.120
2qltA	2qltA01_2qltA02	3.40.50.1000	1.10.150.240
4io1A	4io1A01_4io1A02	3.40.50.1360	3.30.70.260
3myxA	3myxA01_3myxA02	2.60.120.10	2.60.120.10
1uqtA	1uqtA01_1uqtA02	3.40.50.2000	3.40.50.2000
4jzcA	4jzcA01_4jzcA02	3.90.215.10	4.10.530.10
3gzxA	3gzxA01_3gzxA02	3.90.380.10	2.102.10.10
4io2A	4io2A01_4io2A02	3.40.190.10	3.40.190.10
2gbwE	2gbwE01_2gbwE02	3.90.380.10	2.102.10.10
1sozC	1sozC01_1sozC02	2.40.10.10	2.40.10.10
1pb7A	1pb7A01_1pb7A02	3.40.190.10	3.40.190.10
1tolA	1tolA01_1tolA02	2.30.27.10	3.30.1150.10
3vglA	3vglA01_3vglA02	3.30.420.40	3.30.420.40
1sqjA	1sqjA01_1sqjA02	2.130.10.10	2.130.10.10
1aukA	1aukA01_1aukA02	3.40.720.10	3.30.1120.10
3ugvC	3ugvC01_3ugvC02	3.30.390.10	3.20.20.120
2p2wA	2p2wA01_2p2wA02	1.10.580.10	1.10.230.10
3mwcA	3mwcA01_3mwcA02	3.30.390.10	3.20.20.120
4ooxA	4ooxA01_4ooxA02	2.40.510.10	2.40.30.120
3exaD	3exaD01_3exaD03	3.40.50.300	1.10.287.890
3co8A	3co8A01_3co8A02	2.40.37.10	3.20.20.10
3dxvB	3dxvB01_3dxvB02	3.90.1150.10	3.40.640.10
2pozH	2pozH01_2pozH02	3.30.390.10	3.20.20.120
3alfA	3alfA01_3alfA02	3.20.20.80	3.10.50.10
3e2oA	3e2oA01_3e2oA02	1.10.520.10	1.10.420.10
2z9wB	2z9wB01_2z9wB02	3.90.1150.10	3.40.640.10
2exrA	2exrA02_2exrA03	3.30.465.10	3.40.462.10
3dydB	3dydB01_3dydB02	3.90.1150.10	3.40.640.10
4dq6B	4dq6B01_4dq6B02	3.90.1150.10	3.40.640.10
2ozpA	2ozpA01_2ozpA02	3.40.50.720	3.30.360.10
3hcpA	3hcpA01_3hcpA02	3.40.50.1400	3.40.50.1400

2ogaA	2ogaA01_2ogaA02	3.40.640.10	3.90.1150.10
1gwuA	1gwuA01_1gwuA02	1.10.520.10	1.10.420.10
3hdjA	3hdjA01_3hdjA02	3.30.1780.10	3.40.50.720
2b3fA	2b3fA01_2b3fA02	3.40.190.10	3.40.190.10
3hmuA	3hmuA01_3hmuA02	3.90.1150.10	3.40.640.10
2gkjA	2gkjA01_2gkjA02	3.10.310.10	3.10.310.10
2nqtA	2nqtA01_2nqtA02	3.40.50.720	3.30.360.10
1uj6A	1uj6A01_1uj6A02	3.40.50.1360	3.30.70.260
2hzgB	2hzgB01_2hzgB02	3.30.390.10	3.20.20.120
3grfA	3grfA01_3grfA02	3.40.50.1370	3.40.50.1370
1r0mB	1r0mB01_1r0mB02	3.30.390.10	3.20.20.120
4lgnA	4lgnA01_4lgnA02	2.130.10.10	2.130.10.10
3ed4D	3ed4D01_3ed4D02	3.40.720.10	3.30.1120.10
4f2gA	4f2gA01_4f2gA02	3.40.50.1370	3.40.50.1370
1iomA	1iomA01_1iomA02	1.10.580.10	1.10.230.10
3igzB	3igzB01_3igzB02	3.40.720.10	3.40.1450.10
4q6rB	4q6rB02_4q6rB03	3.90.1150.10	3.40.640.10
1omoB	1omoB01_1omoB02	3.30.1780.10	3.40.50.720
4lrsB	4lrsB01_4lrsB02	3.40.50.720	3.30.360.10
1iynA	1iynA01_1iynA02	1.10.520.10	1.10.420.10
1xubA	1xubA01_1xubA02	3.10.310.10	3.10.310.10
4d9iA	4d9iA01_4d9iA02	3.40.50.1100	3.40.50.1100
2o0bA	2o0bA01_2o0bA02	3.65.10.10	3.65.10.10
1e9rA	1e9rA01_1e9rA02	3.40.50.300	1.10.8.80
4g8tD	4g8tD01_4g8tD02	3.30.390.10	3.20.20.120
2huiB	2huiB01_2huiB02	3.90.1150.10	3.40.640.10
4fkzA	4fkzA01_4fkzA02	3.40.50.2000	3.40.50.2000
2oz8A	2oz8A01_2oz8A02	3.30.390.10	3.20.20.120
3aieD	3aieD02_3aieD03	3.20.20.80	3.20.20.470
4h83F	4h83F01_4h83F02	3.30.390.10	3.20.20.120
2e7uA	2e7uA01_2e7uA02	3.90.1150.10	3.40.640.10
2hkeA	2hkeA01_2hkeA02	3.30.230.10	3.30.70.890
2bwnE	2bwnE01_2bwnE02	3.90.1150.10	3.40.640.10
2nyfA	2nyfA01_2nyfA02	1.10.275.10	1.20.200.10
3ff1A	3ff1A01_3ff1A02	3.40.50.10490	3.40.50.10490
2ep5B	2ep5B01_2ep5B02	3.40.50.720	3.30.360.10
2qddA	2qddA01_2qddA02	3.30.390.10	3.20.20.120
3dodA	3dodA01_3dodA02	3.90.1150.10	3.40.640.10
3iujA	3iujA01_3iujA02	3.40.50.1820	2.130.10.120
3tjmA	3tjmA01_3tjmA02	3.40.50.1820	1.10.1470.20
2zgyB	2zgyB01_2zgyB02	3.30.420.40	3.30.420.40
3nieA	3nieA01_3nieA02	3.30.200.20	1.10.510.10
4f6uB	4f6uB01_4f6uB02	1.10.472.10	1.10.472.10
3iqiX	3iqiX01_3iqiX02	3.40.50.1100	3.40.50.1100
3ihjA	3ihjA01_3ihjA03	3.90.1150.10	3.40.640.10
1tq5A	1tq5A01_1tq5A02	2.60.120.10	2.60.120.10
4j27A	4j27A01_4j27A02	3.20.20.80	2.60.40.1180
4ixoB	4ixoB01_4ixoB02	3.90.1150.10	3.40.640.10
4qgrA	4qgrA01_4qgrA02	3.40.640.10	3.90.1150.10
2ymmB	2ymmB01_2ymmB02	3.40.50.1000	1.10.150.240
3rr1A	3rr1A01_3rr1A02	3.30.390.10	3.20.20.120
2b1xC	2b1xC01_2b1xC02	3.90.380.10	2.102.10.10
4oh7B	4oh7B01_4oh7B02	3.40.50.1370	3.40.50.1370
2hqyB	2hqyB01_2hqyB02	3.40.630.30	3.40.630.30
3hurA	3hurA01_3hurA02	2.40.37.10	3.20.20.10
4cvqB	4cvqB01_4cvqB02	3.90.1150.10	3.40.640.10
1rrvB	1rrvB01_1rrvB02	3.40.50.2000	3.40.50.2000

3rcyE	3rcyE01_3rcyE02	3.30.390.10	3.20.20.120
3uwcA	3uwcA01_3uwcA02	3.40.640.10	3.90.1150.10
2w3pB	2w3pB01_2w3pB02	3.90.226.10	3.90.226.10
3qv2A	3qv2A01_3qv2A02	3.40.50.150	3.90.120.10
1l7mA	1l7mA01_1l7mA02	3.40.50.1000	1.10.150.210
4m29A	4m29A01_4m29A02	2.60.40.1500	3.20.20.80
3i5tA	3i5tA01_3i5tA02	3.90.1150.10	3.40.640.10
4clqA	4clqA01_4clqA02	3.65.10.20	3.30.360.20
2cunA	2cunA01_2cunA02	3.40.50.1260	3.40.50.1260
2ifcD	2ifcD01_2ifcD02	1.10.580.10	1.10.230.10
3bqwA	3bqwA01_3bqwA02	3.15.30.10	3.30.1930.10
4ogdA	4ogdA01_4ogdA02	3.40.605.10	3.40.309.10
4jbgB	4jbgB01_4jbgB02	3.90.180.10	3.40.50.720
1lk5C	1lk5C01_1lk5C02	3.40.50.1360	3.30.70.260
1wpcA	1wpcA01_1wpcA03	3.20.20.80	2.60.40.1180
1pfzA	1pfzA01_1pfzA02	2.40.70.10	2.40.70.10
3kq7A	3kq7A01_3kq7A02	3.30.200.20	1.10.510.10
3m00A	3m00A01_3m00A02	1.10.600.10	1.50.10.130
1mdoA	1mdoA01_1mdoA02	3.40.640.10	3.90.1150.10
3ugfB	3ugfB01_3ugfB02	2.115.10.20	2.60.120.560
3eoeD	3eoeD01_3eoeD02	3.40.1380.20	3.20.20.60
4at0A	4at0A01_4at0A02	3.50.50.60	3.90.700.10
2b7nA	2b7nA01_2b7nA02	3.90.1170.20	3.20.20.70
1wb0A	1wb0A01_1wb0A02	3.20.20.80	3.10.50.10
4a0gA	4a0gA02_4a0gA03	3.90.1150.10	3.40.640.10
2rdxB	2rdxB01_2rdxB02	3.30.390.10	3.20.20.120
3hskA	3hskA01_3hskA02	3.40.50.720	3.30.360.10
1kfwA	1kfwA01_1kfwA02	3.20.20.80	3.10.50.10
2q8nC	2q8nC01_2q8nC02	3.40.50.10490	3.40.50.10490
2dx7A	2dx7A01_2dx7A02	3.40.50.1860	3.40.50.1860
1sffC	1sffC01_1sffC02	3.90.1150.10	3.40.640.10
4h31A	4h31A01_4h31A02	3.40.50.1370	3.40.50.1370
3h7jA	3h7jA01_3h7jA02	2.60.120.10	2.60.120.10
3u4fA	3u4fA01_3u4fA02	3.30.390.10	3.20.20.120
3rq1B	3rq1B01_3rq1B02	3.90.1150.10	3.40.640.10
3kznA	3kznA01_3kznA02	3.40.50.1370	3.40.50.1370
4q75A	4q75A01_4q75A02	3.90.1150.10	3.40.640.10
3nraA	3nraA01_3nraA02	3.90.1150.10	3.40.640.10
2zpuA	2zpuA01_2zpuA02	3.40.50.1100	3.40.50.1100
1xe4A	1xe4A01_1xe4A02	3.40.630.30	3.40.630.30
3k2wB	3k2wB01_3k2wB02	3.40.605.10	3.40.309.10
1wyuH	1wyuH02_1wyuH03	3.90.1150.10	3.40.640.10
1u0kA	1u0kA01_1u0kA02	3.10.310.10	3.10.310.10
2yyyA	2yyyA01_2yyyA02	3.40.50.720	3.30.360.10
3pijB	3pijB01_3pijB02	2.115.10.20	2.60.120.560
1gdeA	1gdeA01_1gdeA02	3.90.1150.10	3.40.640.10
3kqjA	3kqjA01_3kqjA02	3.65.10.10	3.65.10.10
2ejwB	2ejwB01_2ejwB02	3.40.50.720	3.30.360.10
1ym5A	1ym5A01_1ym5A02	3.10.310.10	3.10.310.10
3k17B	3k17B01_3k17B02	3.30.230.10	3.30.70.890
2qa9E	2qa9E01_2qa9E02	2.40.10.10	2.40.10.10
3ri6A	3ri6A01_3ri6A02	3.40.640.10	3.90.1150.10
2oztA	2oztA01_2oztA02	3.30.390.10	3.20.20.120
3dr4A	3dr4A01_3dr4A02	3.40.640.10	3.90.1150.10
4e3rA	4e3rA01_4e3rA02	3.90.1150.10	3.40.640.10
4ix8A	4ix8A01_4ix8A02	3.90.1150.10	3.40.640.10
4a6rB	4a6rB01_4a6rB02	3.90.1150.10	3.40.640.10

2oktA	2oktA01_2oktA02	3.30.390.10	3.20.20.120
3cvmB	3cvmB01_3cvmB02	3.30.497.10	2.30.39.10

1464 protein-protein interaction dataset (inter-chain domai			
**PDB ID**	**Domain pair**	**Domain 1 CATH classification**	**Domain 2 CATH classification**
1a2kB	1a2kB01_1a2kC01	3.10.450.50	3.40.50.300
1a2kB	1a2kB01_1a2kE01	3.10.450.50	3.40.50.300
1a4yA	1a4yA01_1a4yB01	3.80.10.10	3.10.130.10
1abrA	1abrA02_1abrB01	4.10.470.10	2.80.10.50
1abrA	1abrA02_1abrB02	4.10.470.10	2.80.10.50
1aksA	1aksA01_1aksB01	2.40.10.10	2.40.10.10
1aokA	1aokA01_1aokB01	1.20.90.10	1.20.90.10
1avaB	1avaB01_1avaD01	3.20.20.80	2.80.10.50
1avaC	1avaC01_1avaA01	2.80.10.50	3.20.20.80
1ay7A	1ay7A01_1ay7B01	3.10.450.30	3.30.370.10
1b2uD	1b2uD01_1b2uA01	3.30.370.10	3.10.450.30
1b33H	1b33H01_1b33I01	1.10.490.20	1.10.490.20
1b33H	1b33H01_1b33M01	1.10.490.20	1.10.490.20
1b33I	1b33I01_1b33J01	1.10.490.20	1.10.490.20
1b4uD	1b4uD01_1b4uC01	3.40.830.10	1.10.700.10
1b5fA	1b5fA01_1b5fB01	2.40.70.10	2.40.70.10
1bd2A	1bd2A01_1bd2D01	3.30.500.10	2.60.40.10
1blxA	1blxA01_1blxB01	3.30.200.20	1.25.40.20
1bndA	1bndA01_1bndB01	2.10.90.10	2.10.90.10
1bouA	1bouA01_1bouB01	1.10.700.10	3.40.830.10
1bvyA	1bvyA01_1bvyB01	1.10.630.10	1.10.630.10
1bz1C	1bz1C01_1bz1B01	1.10.490.10	1.10.490.10
1bz1C	1bz1C01_1bz1D01	1.10.490.10	1.10.490.10
1bz7B	1bz7B01_1bz7A01	2.60.40.10	2.60.40.10
1bz7B	1bz7B02_1bz7A02	2.60.40.10	2.60.40.10
1c1yB	1c1yB01_1c1yA01	3.10.20.90	3.40.50.300
1c3aA	1c3aA01_1c3aB01	3.10.100.10	3.10.100.10
1c7dA	1c7dA01_1c7dB01	1.10.490.10	1.10.490.10
1c7dA	1c7dA02_1c7dB01	1.10.490.10	1.10.490.10
1ca0C	1ca0C01_1ca0B01	2.40.10.10	2.40.10.10
1ca0C	1ca0C01_1ca0D01	2.40.10.10	4.10.410.10
1cauA	1cauA01_1cauB01	2.60.120.10	2.60.120.10
1cb7B	1cb7B01_1cb7A01	3.20.20.240	3.40.50.280
1ccwC	1ccwC01_1ccwD01	3.40.50.280	3.20.20.240
1cg5A	1cg5A01_1cg5B01	1.10.490.10	1.10.490.10
1cicB	1cicB01_1cicA01	2.60.40.10	2.60.40.10
1cicB	1cicB02_1cicA02	2.60.40.10	2.60.40.10
1cmxA	1cmxA01_1cmxB01	3.40.532.10	3.10.20.90
1cs4B	1cs4B01_1cs4A01	3.30.70.1230	3.30.70.1230
1ct8A	1ct8A01_1ct8B01	2.60.40.10	2.60.40.10
1ct8A	1ct8A02_1ct8B02	2.60.40.10	2.60.40.10
1cxzA	1cxzA01_1cxzB01	3.40.50.300	1.10.287.160
1d0gB	1d0gB01_1d0gD01	2.60.120.40	2.60.120.40
1d2zB	1d2zB01_1d2zC01	1.10.533.10	1.10.533.10
1d2zC	1d2zC01_1d2zD01	1.10.533.10	1.10.533.10
1d3bI	1d3bI01_1d3bH01	2.30.30.100	2.30.30.100

1d3bL	1d3bL01_1d3bG01	2.30.30.100	2.30.30.100
1d3bL	1d3bL01_1d3bK01	2.30.30.100	2.30.30.100
1d4vA	1d4vA01_1d4vB01	2.10.50.10	2.60.120.40
1dhkA	1dhkA01_1dhkB01	3.20.20.80	2.60.120.200
1dn0B	1dn0B01_1dn0A01	2.60.40.10	2.60.40.10
1dn0B	1dn0B02_1dn0A02	2.60.40.10	2.60.40.10
1dqjA	1dqjA01_1dqjC01	2.60.40.10	1.10.530.10
1ds6A	1ds6A01_1ds6B01	3.40.50.300	2.70.50.30
1dtdA	1dtdA01_1dtdB01	3.40.630.10	3.30.1040.10
1dvfB	1dvfB01_1dvfD01	2.60.40.10	2.60.40.10
1dvfC	1dvfC01_1dvfD01	2.60.40.10	2.60.40.10
1dxtB	1dxtB01_1dxtA01	1.10.490.10	1.10.490.10
1dxtB	1dxtB01_1dxtC01	1.10.490.10	1.10.490.10
1e1hA	1e1hA01_1e1hB01	3.90.1240.10	4.10.1280.10
1e1hA	1e1hA01_1e1hB02	3.90.1240.10	4.10.1280.10
1e3dA	1e3dA01_1e3dB01	3.40.50.700	1.10.645.10
1e3dA	1e3dA02_1e3dB01	4.10.480.10	1.10.645.10
1e6yC	1e6yC01_1e6yA01	3.90.320.20	3.90.390.10
1e6yC	1e6yC01_1e6yA03	3.90.320.20	1.20.840.10
1e6yC	1e6yC01_1e6yD02	3.90.320.20	3.30.70.470
1e6yD	1e6yD01_1e6yF01	3.90.390.10	3.90.320.20
1e6yD	1e6yD03_1e6yF01	1.20.840.10	3.90.320.20
1e96A	1e96A01_1e96B01	3.40.50.300	1.25.40.10
1eapA	1eapA01_1eapB01	2.60.40.10	2.60.40.10
1eapA	1eapA02_1eapB02	2.60.40.10	2.60.40.10
1eerA	1eerA01_1eerB01	1.20.1250.10	2.60.40.10
1eerA	1eerA01_1eerC01	1.20.1250.10	2.60.40.10
1efnB	1efnB01_1efnA01	3.30.62.10	2.30.30.40
1efvB	1efvB01_1efvA01	3.40.50.620	3.40.50.1220
1efvB	1efvB01_1efvA02	3.40.50.620	3.40.50.620
1em8C	1em8C01_1em8D01	3.40.50.10110	3.40.50.10220
1ep3A	1ep3A01_1ep3B01	3.20.20.70	2.40.30.10
1ep3A	1ep3A01_1ep3B03	3.20.20.70	2.10.240.10
1eptB	1eptB01_1eptC01	2.40.10.10	2.40.10.10
1ewyA	1ewyA02_1ewyC01	3.40.50.80	3.10.20.30
1f2uA	1f2uA01_1f2uD01	3.40.50.300	3.40.50.300
1f2uB	1f2uB01_1f2uC01	3.40.50.300	3.40.50.300
1f3mA	1f3mA01_1f3mB01	3.90.810.10	3.90.810.10
1f3vA	1f3vA01_1f3vB01	3.30.70.680	2.60.210.10
1f6fA	1f6fA01_1f6fB01	1.20.1250.10	2.60.40.10
1f6fA	1f6fA01_1f6fB02	1.20.1250.10	2.60.40.10
1f6fA	1f6fA01_1f6fC01	1.20.1250.10	2.60.40.10
1f80A	1f80A01_1f80E01	3.90.470.20	1.10.1200.10
1f99A	1f99A01_1f99B01	1.10.490.20	1.10.490.20
1ffuF	1ffuF01_1ffuD01	3.30.43.10	3.10.20.30
1ffvD	1ffvD01_1ffvF01	3.10.20.30	3.30.43.10
1fi8D	1fi8D01_1fi8E01	4.10.1230.10	2.60.40.550
1ft1B	1ft1B01_1ft1A01	1.50.10.20	1.25.40.120
1fxkA	1fxkA01_1fxkC01	1.10.287.370	1.10.287.370

1fxkB	1fxkB01_1fxkC01	1.10.287.370	1.10.287.370
1fyhA	1fyhA01_1fyhB01	1.20.1250.10	2.60.40.10
1fz0F	1fz0F01_1fz0B01	1.20.1280.10	1.10.620.20
1fz0F	1fz0F01_1fz0D01	1.20.1280.10	1.10.620.20
1fz0F	1fz0F02_1fz0B01	1.20.1280.30	1.10.620.20
1fz0F	1fz0F02_1fz0D01	1.20.1280.30	1.10.620.20
1fz2A	1fz2A01_1fz2D01	1.10.620.20	1.10.620.20
1g73A	1g73A01_1g73C01	1.20.58.70	1.10.1170.10
1g73D	1g73D01_1g73B01	1.10.1170.10	1.20.58.70
1g7cB	1g7cB01_1g7cA01	3.30.70.60	3.40.50.300
1g7cB	1g7cB01_1g7cA02	3.30.70.60	2.40.30.10
1gcqA	1gcqA01_1gcqC01	2.30.30.40	2.30.30.40
1gcqB	1gcqB01_1gcqC01	2.30.30.40	2.30.30.40
1gcvA	1gcvA01_1gcvB01	1.10.490.10	1.10.490.10
1gcvA	1gcvA01_1gcvD01	1.10.490.10	1.10.490.10
1gcvB	1gcvB01_1gcvC01	1.10.490.10	1.10.490.10
1gl2A	1gl2A01_1gl2B01	1.20.5.110	1.20.5.110
1gl2A	1gl2A01_1gl2D01	1.20.5.110	1.20.5.110
1gl2B	1gl2B01_1gl2C01	1.20.5.110	1.20.5.110
1gl2C	1gl2C01_1gl2D01	1.20.5.110	1.20.5.110
1gl4A	1gl4A01_1gl4B01	2.40.155.10	2.60.40.10
1gpqB	1gpqB01_1gpqC01	3.40.1420.10	1.10.530.10
1gpqD	1gpqD01_1gpqA01	1.10.530.10	3.40.1420.10
1gpwA	1gpwA01_1gpwB01	3.20.20.70	3.40.50.880
1grnA	1grnA01_1grnB01	3.40.50.300	1.10.555.10
1guxA	1guxA01_1guxB01	1.10.472.10	1.10.472.10
1gvnA	1gvnA01_1gvnB01	1.10.8.130	3.40.50.300
1gzsA	1gzsA01_1gzsB01	3.40.50.300	1.10.4120.10
1gzsB	1gzsB01_1gzsC01	1.10.4120.10	3.40.50.300
1h0dB	1h0dB01_1h0dC01	2.60.40.10	3.10.130.10
1h2sA	1h2sA01_1h2sB01	1.20.1070.10	1.10.287.470
1hbmA	1hbmA01_1hbmC01	3.90.390.10	3.90.320.20
1hbmA	1hbmA02_1hbmF01	3.30.70.470	3.90.320.20
1hbmA	1hbmA03_1hbmC01	1.20.840.10	3.90.320.20
1hbrA	1hbrA01_1hbrB01	1.10.490.10	1.10.490.10
1hbrD	1hbrD01_1hbrC01	1.10.490.10	1.10.490.10
1hdsA	1hdsA01_1hdsB01	1.10.490.10	1.10.490.10
1hdsA	1hdsA01_1hdsD01	1.10.490.10	1.10.490.10
1hdsD	1hdsD01_1hdsC01	1.10.490.10	1.10.490.10
1he1B	1he1B01_1he1D01	1.20.120.260	3.40.50.300
1he1C	1he1C01_1he1A01	3.40.50.300	1.20.120.260
1hl6A	1hl6A01_1hl6B01	3.30.70.330	3.30.1560.10
1hq3H	1hq3H01_1hq3F01	1.10.20.10	1.10.20.10
1hr6A	1hr6A01_1hr6B02	3.30.830.10	3.30.830.10
1hr6A	1hr6A02_1hr6B01	3.30.830.10	3.30.830.10
1hwgA	1hwgA01_1hwgB01	1.20.1250.10	2.60.40.10
1hwgA	1hwgA01_1hwgB02	1.20.1250.10	2.60.40.10
1hwgA	1hwgA01_1hwgC01	1.20.1250.10	2.60.40.10
1i1qA	1i1qA01_1i1qB01	3.60.120.10	3.40.50.880

1i2mA	1i2mA01_1i2mB01	3.40.50.300	2.130.10.30
1i2mB	1i2mB01_1i2mC01	2.130.10.30	3.40.50.300
1i2mD	1i2mD01_1i2mC01	2.130.10.30	3.40.50.300
1i3rH	1i3rH01_1i3rG01	3.10.320.10	3.10.320.10
1i3rH	1i3rH01_1i3rG02	3.10.320.10	2.60.40.10
1i79A	1i79A01_1i79B01	3.60.90.10	3.30.360.50
1i7cB	1i7cB01_1i7cA01	3.30.360.50	3.60.90.10
1i7qC	1i7qC01_1i7qD01	3.60.120.10	3.40.50.880
1i7sB	1i7sB01_1i7sA01	3.40.50.880	3.60.120.10
1i7yB	1i7yB01_1i7yA01	1.10.490.20	1.10.490.20
1i7zC	1i7zC01_1i7zD01	2.60.40.10	2.60.40.10
1i7zC	1i7zC02_1i7zD02	2.60.40.10	2.60.40.10
1i8iA	1i8iA01_1i8iB01	2.60.40.10	2.60.40.10
1iarA	1iarA01_1iarB01	1.20.1250.10	2.60.40.10
1ibrA	1ibrA01_1ibrB01	3.40.50.300	1.25.10.10
1ibrD	1ibrD01_1ibrC01	1.25.10.10	3.40.50.300
1iilG	1iilG01_1iilC01	2.60.40.10	2.80.10.50
1iilG	1iilG02_1iilC01	2.60.40.10	2.80.10.50
1iknA	1iknA02_1iknC01	2.60.40.10	2.60.40.10
1iodB	1iodB01_1iodA01	3.10.100.10	3.10.100.10
1iqdA	1iqdA01_1iqdB01	2.60.40.10	2.60.40.10
1iqdA	1iqdA01_1iqdC01	2.60.40.10	2.60.120.260
1iqdA	1iqdA02_1iqdB02	2.60.40.10	2.60.40.10
1iqdB	1iqdB01_1iqdC01	2.60.40.10	2.60.120.260
1itbA	1itbA01_1itbB02	2.80.10.50	2.60.40.10
1itbA	1itbA01_1itbB03	2.80.10.50	2.60.40.10
1ixxB	1ixxB01_1ixxA01	3.10.100.10	3.10.100.10
1j3iB	1j3iB01_1j3iC01	3.40.430.10	3.30.572.10
1j3iB	1j3iB01_1j3iD01	3.40.430.10	3.30.572.10
1j3iD	1j3iD01_1j3iA01	3.30.572.10	3.40.430.10
1j8hA	1j8hA01_1j8hE01	3.10.320.10	2.60.40.10
1j8hE	1j8hE01_1j8hD01	2.60.40.10	2.60.40.10
1j8hE	1j8hE02_1j8hD02	2.60.40.10	2.60.40.10
1jb0D	1jb0D01_1jb0A01	3.30.1470.10	1.20.1130.10
1jb0D	1jb0D01_1jb0B01	3.30.1470.10	1.20.1130.10
1jb0D	1jb0D01_1jb0C01	3.30.1470.10	3.30.70.20
1jb0E	1jb0E01_1jb0A01	2.30.30.50	1.20.1130.10
1jb0E	1jb0E01_1jb0C01	2.30.30.50	3.30.70.20
1jebB	1jebB01_1jebA01	1.10.490.10	1.10.490.10
1jebC	1jebC01_1jebD01	1.10.490.10	1.10.490.10
1jebD	1jebD01_1jebA01	1.10.490.10	1.10.490.10
1jjuA	1jjuA01_1jjuB01	2.60.40.10	2.130.10.10
1jjuA	1jjuA01_1jjuC01	2.60.40.10	4.10.940.10
1jjuA	1jjuA04_1jjuC01	2.60.40.10	4.10.940.10
1jjuB	1jjuB01_1jjuC01	2.130.10.10	4.10.940.10
1jkgA	1jkgA01_1jkgB01	3.10.450.50	3.10.450.50
1jmxB	1jmxB01_1jmxA01	2.130.10.10	2.60.40.10
1jmzA	1jmzA01_1jmzB01	2.60.40.10	2.130.10.10
1jsuA	1jsuA01_1jsuC01	3.30.200.20	4.10.365.10

1jtdA	1jtdA01_1jtdB01	3.40.710.10	2.130.10.30
1jthB	1jthB01_1jthA01	1.20.5.110	1.20.5.110
1jthC	1jthC01_1jthD01	1.20.5.110	1.20.5.110
1jv5A	1jv5A01_1jv5B01	2.60.40.10	2.60.40.10
1jwiB	1jwiB01_1jwiA01	3.10.100.10	3.10.100.10
1jyoB	1jyoB01_1jyoE01	3.30.1460.10	4.10.1330.10
1jyoB	1jyoB01_1jyoF01	3.30.1460.10	4.10.1330.10
1jyoD	1jyoD01_1jyoE01	3.30.1460.10	4.10.1330.10
1jyoF	1jyoF01_1jyoA01	4.10.1330.10	3.30.1460.10
1jyoF	1jyoF01_1jyoC01	4.10.1330.10	3.30.1460.10
1jzdB	1jzdB02_1jzdC01	3.40.30.10	2.60.40.1250
1k4dA	1k4dA01_1k4dC01	2.60.40.10	1.10.287.70
1k8kD	1k8kD01_1k8kF01	3.30.1460.20	3.30.1460.20
1kb5A	1kb5A01_1kb5B01	2.60.40.10	2.60.40.10
1kb9B	1kb9B01_1kb9A02	3.30.830.10	3.30.830.10
1kb9B	1kb9B02_1kb9A01	3.30.830.10	3.30.830.10
1ki1B	1ki1B01_1ki1A01	1.20.900.10	3.40.50.300
1ki1C	1ki1C01_1ki1D01	3.40.50.300	1.20.900.10
1kiqC	1kiqC01_1kiqB01	1.10.530.10	2.60.40.10
1kirB	1kirB01_1kirC01	2.60.40.10	1.10.530.10
1kjvA	1kjvA01_1kjvB01	3.30.500.10	2.60.40.10
1kjvA	1kjvA02_1kjvB01	2.60.40.10	2.60.40.10
1kn1B	1kn1B01_1kn1A01	1.10.490.20	1.10.490.20
1ksgB	1ksgB01_1ksgA01	2.70.50.40	3.40.50.300
1kshA	1kshA01_1kshB01	3.40.50.300	2.70.50.40
1kvdC	1kvdC01_1kvdD01	4.10.420.10	3.30.44.10
1kx5C	1kx5C01_1kx5D01	1.10.20.10	1.10.20.10
1kx5H	1kx5H01_1kx5G01	1.10.20.10	1.10.20.10
1kxqA	1kxqA01_1kxqH01	3.20.20.80	2.60.40.10
1kxqG	1kxqG01_1kxqB01	2.60.40.10	3.20.20.80
1kxtD	1kxtD01_1kxtC01	2.60.40.10	3.20.20.80
1kxtE	1kxtE01_1kxtF01	3.20.20.80	2.60.40.10
1kxvA	1kxvA01_1kxvC01	3.20.20.80	2.60.40.10
1kxvD	1kxvD01_1kxvB01	2.60.40.10	3.20.20.80
1kzyC	1kzyC01_1kzyA01	3.40.50.10190	2.60.40.720
1l0lF	1l0lF01_1l0lC01	1.10.1090.10	1.20.810.10
1l0lH	1l0lH01_1l0lD02	1.10.287.20	1.10.760.10
1lfdA	1lfdA01_1lfdB01	3.10.20.90	3.40.50.300
1lfdD	1lfdD01_1lfdC01	3.40.50.300	3.10.20.90
1lm8C	1lm8C01_1lm8B01	3.30.710.10	3.10.20.90
1lnuH	1lnuH01_1lnuG01	3.10.320.10	3.10.320.10
1lnuH	1lnuH01_1lnuG02	3.10.320.10	2.60.40.10
1lp1B	1lp1B01_1lp1A01	1.20.5.420	1.20.5.420
1lrwA	1lrwA01_1lrwB01	2.140.10.10	4.10.160.10
1lrwD	1lrwD01_1lrwC01	4.10.160.10	2.140.10.10
1lshA	1lshA04_1lshB01	2.20.80.10	2.20.90.10
1lujA	1lujA01_1lujB01	1.25.10.10	1.10.10.490
1lywB	1lywB01_1lywC01	2.40.70.10	2.40.70.10
1lzwB	1lzwB01_1lzwA01	1.10.1780.10	3.30.1390.10

1m1eA	1m1eA01_1m1eB01	1.25.10.10	1.10.10.490
1m9yE	1m9yE01_1m9yH01	2.40.100.10	1.10.375.10
1mg2B	1mg2B01_1mg2C01	2.60.30.10	2.60.40.420
1mh5A	1mh5A01_1mh5B01	2.60.40.10	2.60.40.10
1mh5A	1mh5A02_1mh5B02	2.60.40.10	2.60.40.10
1mhcA	1mhcA01_1mhcB01	3.30.500.10	2.60.40.10
1mhcA	1mhcA02_1mhcB01	2.60.40.10	2.60.40.10
1mhmA	1mhmA01_1mhmB01	3.60.90.10	3.30.360.50
1mlcB	1mlcB01_1mlcA01	2.60.40.10	2.60.40.10
1mlcB	1mlcB01_1mlcE01	2.60.40.10	1.10.530.10
1mlcB	1mlcB02_1mlcA02	2.60.40.10	2.60.40.10
1mlcF	1mlcF01_1mlcD01	1.10.530.10	2.60.40.10
1mt1E	1mt1E01_1mt1D01	3.30.60.30	3.50.20.10
1mt1E	1mt1E01_1mt1F01	3.30.60.30	3.50.20.10
1mz8B	1mz8B01_1mz8A01	3.90.540.10	1.10.1200.20
1n0lA	1n0lA01_1n0lB01	2.60.40.10	2.60.40.1090
1n13D	1n13D01_1n13E01	3.50.20.10	3.30.60.30
1n5wA	1n5wA01_1n5wC01	3.10.20.30	3.30.43.10
1n7sD	1n7sD01_1n7sC01	1.20.5.110	1.20.5.110
1nbzC	1nbzC01_1nbzA01	1.10.530.10	2.60.40.10
1ndgB	1ndgB01_1ndgA01	2.60.40.10	2.60.40.10
1ndgB	1ndgB01_1ndgC01	2.60.40.10	1.10.530.10
1ndgB	1ndgB02_1ndgA02	2.60.40.10	2.60.40.10
1nezA	1nezA01_1nezB01	3.30.500.10	2.60.40.10
1nezA	1nezA02_1nezB01	2.60.40.10	2.60.40.10
1nf3B	1nf3B01_1nf3D01	3.40.50.300	2.30.42.10
1nheB	1nheB01_1nheA01	3.90.550.10	1.10.530.10
1npeA	1npeA01_1npeB01	2.120.10.30	2.10.25.10
1npeA	1npeA01_1npeB02	2.120.10.30	2.10.25.10
1nrjA	1nrjA01_1nrjB01	3.30.450.60	3.40.50.300
1ntmI	1ntmI01_1ntmA02	2.10.210.10	3.30.830.10
1ntmI	1ntmI01_1ntmB01	2.10.210.10	3.30.830.10
1ntmI	1ntmI01_1ntmB02	2.10.210.10	3.30.830.10
1nw9A	1nw9A01_1nw9B01	1.10.1170.10	3.40.50.1460
1oaoC	1oaoC01_1oaoA02	1.10.8.190	3.40.50.2030
1oaoC	1oaoC01_1oaoB03	1.10.8.190	1.20.1270.30
1oaoC	1oaoC02_1oaoB01	3.40.50.2030	3.40.50.2030
1ocrE	1ocrE01_1ocrD01	1.25.40.40	1.10.442.10
1ocrE	1ocrE01_1ocrI01	1.25.40.40	4.10.93.10
1ogaD	1ogaD01_1ogaE01	2.60.40.10	2.60.40.10
1ogaD	1ogaD02_1ogaE02	2.60.40.10	2.60.40.10
1onqA	1onqA01_1onqB01	3.30.500.10	2.60.40.10
1onqA	1onqA02_1onqB01	2.60.40.10	2.60.40.10
1op9A	1op9A01_1op9B01	2.60.40.10	1.10.530.10
1oqmA	1oqmA01_1oqmB01	1.10.530.10	3.90.550.10
1oqsA	1oqsA01_1oqsF01	1.20.90.10	1.20.90.10
1oqsH	1oqsH01_1oqsG01	1.20.90.10	1.20.90.10
1or7A	1or7A01_1or7B01	1.10.1740.10	1.10.1740.10
1orsB	1orsB01_1orsC01	2.60.40.10	1.20.120.350

1outB	1outB01_1outA01	1.10.490.10	1.10.490.10
1ouuA	1ouuA01_1ouuB01	1.10.490.10	1.10.490.10
1owsA	1owsA01_1owsB01	1.20.90.10	1.20.90.10
1oxkE	1oxkE01_1oxkB01	1.20.120.160	3.40.50.2300
1oxkF	1oxkF01_1oxkC01	3.40.50.2300	1.20.120.160
1oy3B	1oy3B01_1oy3C01	2.60.40.10	2.60.40.10
1oz7A	1oz7A01_1oz7B01	3.10.100.10	3.10.100.10
1p27C	1p27C01_1p27D01	3.30.1560.10	3.30.70.330
1p5uB	1p5uB01_1p5uA01	2.60.40.1310	2.60.40.10
1p7kA	1p7kA01_1p7kB01	2.60.40.10	2.60.40.10
1p7kA	1p7kA02_1p7kB02	2.60.40.10	2.60.40.10
1p84E	1p84E01_1p84A01	1.20.5.270	3.30.830.10
1p84E	1p84E01_1p84C01	1.20.5.270	1.20.810.10
1p84E	1p84E01_1p84D01	1.20.5.270	1.20.5.100
1p84E	1p84E01_1p84H01	1.20.5.270	1.20.5.210
1p84E	1p84E01_1p84I01	1.20.5.270	1.20.5.260
1p84E	1p84E02_1p84J01	2.102.10.10	2.60.40.10
1pbyC	1pbyC01_1pbyA01	4.10.940.10	2.60.40.10
1pbyC	1pbyC01_1pbyA04	4.10.940.10	2.60.40.10
1pbyC	1pbyC01_1pbyB01	4.10.940.10	2.130.10.10
1pdkA	1pdkA01_1pdkB01	2.60.40.10	2.60.40.1090
1pk1A	1pk1A01_1pk1B01	1.10.150.50	1.10.150.50
1pqzA	1pqzA01_1pqzB01	3.30.500.10	2.60.40.10
1pqzA	1pqzA02_1pqzB01	2.60.40.10	2.60.40.10
1pvhB	1pvhB01_1pvhA01	1.20.1250.10	2.60.40.10
1pvhC	1pvhC01_1pvhD01	2.60.40.10	1.20.1250.10
1pxvB	1pxvB01_1pxvD01	3.90.70.10	2.40.310.10
1pxvC	1pxvC01_1pxvA01	2.40.310.10	3.90.70.10
1pyaE	1pyaE01_1pyaD01	4.10.510.10	3.50.20.10
1pyaE	1pyaE01_1pyaF01	4.10.510.10	3.50.20.10
1pytA	1pytA01_1pytB01	3.30.70.340	3.40.630.10
1pz5B	1pz5B01_1pz5A01	2.60.40.10	2.60.40.10
1pz5B	1pz5B02_1pz5A02	2.60.40.10	2.60.40.10
1q40A	1q40A01_1q40B01	3.10.450.50	3.10.450.50
1qavA	1qavA01_1qavB01	2.30.42.10	2.30.42.10
1qdlA	1qdlA01_1qdlB01	3.60.120.10	3.40.50.880
1qo3A	1qo3A01_1qo3C01	3.30.500.10	3.10.100.10
1r5vC	1r5vC01_1r5vD01	3.10.320.10	3.10.320.10
1r5vC	1r5vC02_1r5vD01	2.60.40.10	3.10.320.10
1r6oC	1r6oC01_1r6oA01	3.30.1390.10	1.10.1780.10
1re0A	1re0A01_1re0B02	3.40.50.300	1.10.1000.11
1rhhB	1rhhB01_1rhhC02	2.60.40.10	2.60.40.10
1ri8A	1ri8A01_1ri8B01	2.60.40.10	1.10.530.10
1riwC	1riwC01_1riwB01	2.40.10.10	2.40.10.10
1rjcA	1rjcA01_1rjcB01	2.60.40.10	1.10.530.10
1rk8B	1rk8B01_1rk8A01	3.30.1560.10	3.30.70.330
1rkeA	1rkeA01_1rkeB01	1.20.120.230	1.20.120.230
1rldA	1rldA01_1rldB02	3.30.70.150	3.20.20.110
1rldA	1rldA02_1rldB01	3.20.20.110	3.30.70.150

1rldA	1rldA02_1rldB02	3.20.20.110	3.20.20.110
1rm6B	1rm6B01_1rm6C01	3.30.390.50	3.10.20.30
1rp3B	1rp3B01_1rp3A01	1.10.287.1160	1.10.1740.10
1rp3B	1rp3B02_1rp3A02	1.10.287.1160	1.10.1740.10
1rscG	1rscG01_1rscH02	3.30.70.150	3.20.20.110
1rscG	1rscG02_1rscH01	3.20.20.110	3.30.70.150
1rscG	1rscG02_1rscH02	3.20.20.110	3.20.20.110
1rwxA	1rwxA01_1rwxB01	3.40.50.1460	3.30.70.1470
1s1qA	1s1qA01_1s1qB01	3.10.110.10	3.10.20.90
1s4yC	1s4yC01_1s4yD01	2.10.60.10	2.10.90.10
1s7wA	1s7wA01_1s7wK01	3.30.500.10	2.60.40.10
1sb3C	1sb3C01_1sb3B01	3.10.20.30	3.30.390.50
1sc3B	1sc3B01_1sc3A01	3.30.70.1470	3.40.50.1460
1scjA	1scjA01_1scjB01	3.40.50.200	3.30.70.80
1sfcB	1sfcB01_1sfcD01	1.20.5.110	1.20.5.110
1sfcG	1sfcG01_1sfcH01	1.20.5.110	1.20.5.110
1shwA	1shwA01_1shwB01	2.60.40.420	2.60.120.260
1spgA	1spgA01_1spgB01	1.10.490.10	1.10.490.10
1sppA	1sppA01_1sppB01	2.60.120.290	2.60.120.290
1sr4A	1sr4A01_1sr4B01	2.80.10.50	3.60.10.10
1sr4A	1sr4A01_1sr4C01	2.80.10.50	2.80.10.50
1sr4C	1sr4C01_1sr4B01	2.80.10.50	3.60.10.10
1sv0B	1sv0B01_1sv0D01	1.10.150.50	1.10.150.50
1sv0C	1sv0C01_1sv0A01	1.10.150.50	1.10.150.50
1sz6A	1sz6A02_1sz6B01	4.10.470.10	2.80.10.50
1sz6A	1sz6A02_1sz6B02	4.10.470.10	2.80.10.50
1t0jA	1t0jA01_1t0jB01	2.30.30.40	3.40.50.300
1t0pA	1t0pA01_1t0pB01	3.40.50.410	2.60.40.10
1t3fB	1t3fB01_1t3fA01	2.60.40.10	2.60.40.10
1t3fB	1t3fB02_1t3fA02	2.60.40.10	2.60.40.10
1t3qD	1t3qD01_1t3qF01	3.10.20.30	3.30.43.10
1t4kB	1t4kB01_1t4kA01	2.60.40.10	2.60.40.10
1t4kB	1t4kB02_1t4kA02	2.60.40.10	2.60.40.10
1t60C	1t60C01_1t60H01	2.170.240.10	2.170.240.10
1t60G	1t60G01_1t60I01	2.170.240.10	2.170.240.10
1t60I	1t60I01_1t60H01	2.170.240.10	2.170.240.10
1t60I	1t60I01_1t60K01	2.170.240.10	2.170.240.10
1t60N	1t60N01_1t60X01	2.170.240.10	2.170.240.10
1t60T	1t60T01_1t60U01	2.170.240.10	2.170.240.10
1t60T	1t60T01_1t60X01	2.170.240.10	2.170.240.10
1t6gB	1t6gB02_1t6gD01	2.40.70.10	2.60.120.180
1t6gC	1t6gC01_1t6gA02	2.60.120.180	2.40.70.10
1ta3A	1ta3A01_1ta3B01	3.20.20.80	3.20.20.80
1tafA	1tafA01_1tafB01	1.10.20.10	1.10.20.10
1tbgB	1tbgB01_1tbgA01	2.130.10.10	2.130.10.10
1tcoA	1tcoA01_1tcoC01	3.60.21.10	3.10.50.40
1tcoB	1tcoB01_1tcoA01	1.10.238.10	3.60.21.10
1te1A	1te1A01_1te1B01	3.20.20.80	2.60.120.180
1tejB	1tejB01_1tejA01	4.10.70.10	4.10.70.10

1tmqA	1tmqA01_1tmqB01	3.20.20.80	1.10.110.10
1tqyB	1tqyB01_1tqyA01	3.40.47.10	3.40.47.10
1tqyB	1tqyB01_1tqyA02	3.40.47.10	3.40.47.10
1tqyB	1tqyB02_1tqyA01	3.40.47.10	3.40.47.10
1tqyG	1tqyG01_1tqyH01	3.40.47.10	3.40.47.10
1tqyG	1tqyG01_1tqyH02	3.40.47.10	3.40.47.10
1tqyG	1tqyG02_1tqyH01	3.40.47.10	3.40.47.10
1tu3E	1tu3E01_1tu3J01	3.40.50.300	1.20.5.730
1u58B	1u58B01_1u58A01	2.60.40.10	3.30.500.10
1u58B	1u58B01_1u58A02	2.60.40.10	2.60.40.10
1uadA	1uadA01_1uadC01	3.40.50.300	2.60.40.10
1uadD	1uadD01_1uadB01	2.60.40.10	3.40.50.300
1ub4A	1ub4A01_1ub4C01	2.30.30.110	2.10.260.10
1ugsA	1ugsA01_1ugsB01	3.90.330.10	1.10.472.20
1ugsA	1ugsA01_1ugsB02	3.90.330.10	2.30.30.50
1uj3A	1uj3A01_1uj3C02	2.60.40.10	2.60.40.10
1uj3B	1uj3B01_1uj3C02	2.60.40.10	2.60.40.10
1umdA	1umdA01_1umdB01	3.40.50.970	3.40.50.970
1umdA	1umdA01_1umdD01	3.40.50.970	3.40.50.970
1umdA	1umdA01_1umdD02	3.40.50.970	3.40.50.920
1umdB	1umdB01_1umdC01	3.40.50.970	3.40.50.970
1umdB	1umdB02_1umdC01	3.40.50.920	3.40.50.970
1umrB	1umrB01_1umrD01	3.10.100.10	3.10.100.10
1umrC	1umrC01_1umrA01	3.10.100.10	3.10.100.10
1unnA	1unnA03_1unnD01	3.10.150.10	3.30.1490.100
1uptA	1uptA01_1uptB01	3.40.50.300	1.10.220.60
1urqA	1urqA01_1urqB01	1.20.5.110	1.20.5.110
1urqA	1urqA01_1urqD01	1.20.5.110	1.20.5.110
1uuzA	1uuzA01_1uuzD01	3.40.1420.10	1.10.530.10
1uuzC	1uuzC01_1uuzB01	1.10.530.10	3.40.1420.10
1uw4C	1uw4C01_1uw4D01	3.30.70.330	1.25.40.180
1uzxA	1uzxA01_1uzxB01	3.10.110.10	3.10.20.90
1v1mA	1v1mA01_1v1mB02	3.40.50.970	3.40.50.970
1v1rB	1v1rB02_1v1rA01	3.40.50.970	3.40.50.970
1v4xB	1v4xB01_1v4xA01	1.10.490.10	1.10.490.10
1v4xB	1v4xB01_1v4xC01	1.10.490.10	1.10.490.10
1v4xC	1v4xC01_1v4xD01	1.10.490.10	1.10.490.10
1v5iA	1v5iA01_1v5iB01	3.40.50.200	3.30.70.80
1v75A	1v75A01_1v75B01	1.10.490.10	1.10.490.10
1v7pA	1v7pA01_1v7pB01	3.10.100.10	3.10.100.10
1v7pB	1v7pB01_1v7pC01	3.10.100.10	3.40.50.410
1vraA	1vraA01_1vraB01	3.60.70.12	3.30.2330.10
1w6sB	1w6sB01_1w6sA01	4.10.160.10	2.140.10.10
1w6sC	1w6sC01_1w6sD01	2.140.10.10	4.10.160.10
1w85D	1w85D01_1w85A01	3.40.50.970	3.40.50.970
1w85D	1w85D01_1w85C01	3.40.50.970	3.40.50.970
1w85D	1w85D02_1w85A01	3.40.50.920	3.40.50.970
1wmhA	1wmhA01_1wmhB01	3.10.20.90	3.10.20.90
1wrdA	1wrdA01_1wrdB01	1.20.58.160	3.10.20.90

1wt5A	1wt5A01_1wt5C01	2.60.40.10	2.60.40.10
1wt5D	1wt5D01_1wt5B01	2.60.40.10	2.60.40.10
1wt9A	1wt9A01_1wt9B01	3.10.100.10	3.10.100.10
1wveA	1wveA02_1wveB02	3.30.465.10	3.30.465.10
1wveA	1wveA02_1wveB03	3.30.465.10	3.40.462.10
1wveA	1wveA02_1wveB04	3.30.465.10	1.10.45.10
1wveA	1wveA03_1wveB02	3.40.462.10	3.30.465.10
1wveA	1wveA04_1wveB02	1.10.45.10	3.30.465.10
1wywA	1wywA01_1wywB01	3.40.470.10	3.10.20.90
1x1wB	1x1wB01_1x1wE01	3.10.450.30	3.30.370.10
1x2tA	1x2tA01_1x2tB01	3.10.100.10	3.10.100.10
1xd3A	1xd3A01_1xd3B01	3.40.532.10	3.10.20.90
1xd3D	1xd3D01_1xd3C01	3.10.20.90	3.40.532.10
1xexA	1xexA01_1xexB01	3.40.50.300	3.40.50.300
1xf6A	1xf6A01_1xf6B01	3.90.510.10	3.90.510.10
1xf6A	1xf6A01_1xf6C01	3.90.510.10	1.10.490.20
1xf6A	1xf6A01_1xf6D01	3.90.510.10	1.10.490.20
1xf6B	1xf6B01_1xf6D01	3.90.510.10	1.10.490.20
1xg0C	1xg0C01_1xg0A01	1.10.490.20	3.90.510.10
1xg0D	1xg0D01_1xg0A01	1.10.490.20	3.90.510.10
1xg0D	1xg0D01_1xg0B01	1.10.490.20	3.90.510.10
1xg2A	1xg2A01_1xg2B01	2.160.20.10	1.20.140.40
1xgrB	1xgrB01_1xgrC01	2.60.40.10	1.10.530.10
1xiwA	1xiwA01_1xiwB01	2.60.40.10	2.60.40.10
1xiwA	1xiwA01_1xiwD01	2.60.40.10	2.60.40.10
1xiwB	1xiwB01_1xiwG01	2.60.40.10	2.60.40.10
1xiwD	1xiwD01_1xiwC01	2.60.40.10	2.60.40.10
1xiwG	1xiwG01_1xiwH01	2.60.40.10	2.60.40.10
1xkpB	1xkpB01_1xkpC01	3.30.1460.10	3.30.1460.10
1y0lD	1y0lD01_1y0lC01	2.60.40.10	2.60.40.10
1y0lD	1y0lD02_1y0lC02	2.60.40.10	2.60.40.10
1ycsA	1ycsA01_1ycsB02	2.60.40.720	2.30.30.40
1yq3B	1yq3B01_1yq3C01	3.10.20.30	1.20.5.540
1yq3B	1yq3B02_1yq3C01	1.10.1060.10	1.20.5.540
1yq3B	1yq3B02_1yq3C02	1.10.1060.10	1.20.1300.10
1yq3B	1yq3B02_1yq3D01	1.10.1060.10	1.20.1300.10
1yu6B	1yu6B01_1yu6D01	3.40.50.200	3.30.60.30
1z3eA	1z3eA01_1z3eB01	3.40.30.10	1.10.150.20
1z8uC	1z8uC01_1z8uD01	1.20.58.420	1.10.490.10
1zc4B	1zc4B01_1zc4C01	2.30.29.30	3.40.50.300
1zvyA	1zvyA01_1zvyB01	2.60.40.10	1.10.530.10
2a19A	2a19A01_2a19B02	2.40.50.140	1.10.510.10
2a1uA	2a1uA01_2a1uB01	3.40.50.620	3.40.50.620
2a1uA	2a1uA02_2a1uB01	3.40.50.1220	3.40.50.620
2a5dA	2a5dA01_2a5dB01	3.40.50.300	3.90.210.10
2a6qA	2a6qA02_2a6qE01	3.40.1620.10	3.30.2310.20
2a78A	2a78A01_2a78B01	3.40.50.300	3.90.176.10
2a9kB	2a9kB01_2a9kA01	3.90.176.10	3.40.50.300
2aa1A	2aa1A01_2aa1B01	1.10.490.10	1.10.490.10

2aa1A	2aa1A01_2aa1D01	1.10.490.10	1.10.490.10
2aa1B	2aa1B01_2aa1C01	1.10.490.10	1.10.490.10
2aaiA	2aaiA02_2aaiB01	4.10.470.10	2.80.10.50
2aaiA	2aaiA02_2aaiB02	4.10.470.10	2.80.10.50
2abzB	2abzB01_2abzE01	3.40.630.10	3.30.1040.10
2abzC	2abzC01_2abzA01	3.30.1040.10	3.40.630.10
2ad7B	2ad7B01_2ad7A01	4.10.160.10	2.140.10.10
2adgB	2adgB01_2adgA01	2.60.40.10	2.60.40.10
2adgB	2adgB02_2adgA02	2.60.40.10	2.60.40.10
2ak4I	2ak4I01_2ak4J01	2.60.40.10	2.60.40.10
2ak4I	2ak4I02_2ak4J02	2.60.40.10	2.60.40.10
2ap2B	2ap2B01_2ap2A01	2.60.40.10	2.60.40.10
2ap2C	2ap2C01_2ap2D01	2.60.40.10	2.60.40.10
2astB	2astB01_2astC01	3.80.10.10	3.30.170.10
2b4sA	2b4sA01_2b4sB02	3.90.190.10	1.10.510.10
2b4sD	2b4sD02_2b4sC01	1.10.510.10	3.90.190.10
2b5uA	2b5uA01_2b5uB01	3.10.380.10	3.10.50.20
2b5uA	2b5uA03_2b5uB01	3.10.380.10	3.10.50.20
2bc4C	2bc4C01_2bc4D01	3.10.320.10	3.10.320.10
2bc4C	2bc4C02_2bc4D02	2.60.40.10	2.60.40.10
2bexA	2bexA01_2bexC01	3.80.10.10	3.10.130.10
2bexD	2bexD01_2bexB01	3.10.130.10	3.80.10.10
2bffA	2bffA01_2bffB01	3.40.50.970	3.40.50.970
2bkkB	2bkkB01_2bkkA02	1.25.40.20	3.90.1200.10
2bkkC	2bkkC02_2bkkD01	3.90.1200.10	1.25.40.20
2bo9A	2bo9A01_2bo9B02	3.40.630.10	3.10.450.10
2bo9D	2bo9D02_2bo9C01	3.10.450.10	3.40.630.10
2bumA	2bumA01_2bumB01	2.60.130.10	2.60.130.10
2butB	2butB01_2butA01	2.60.130.10	2.60.130.10
2bykA	2bykA01_2bykB01	1.10.20.10	1.10.20.10
2bykD	2bykD01_2bykC01	1.10.20.10	1.10.20.10
2c0lA	2c0lA01_2c0lB01	1.25.40.10	3.30.1050.10
2cdfA	2cdfA01_2cdfB01	2.60.40.10	2.60.40.10
2cdfA	2cdfA02_2cdfB02	2.60.40.10	2.60.40.10
2cdrB	2cdrB01_2cdrA01	3.30.70.1470	3.40.50.1460
2cfhB	2cfhB01_2cfhD01	3.30.1380.20	3.30.1380.20
2cnnA	2cnnA01_2cnnB01	3.40.50.1460	3.30.70.1470
2d07A	2d07A01_2d07B01	3.40.470.10	3.10.20.90
2d0vA	2d0vA01_2d0vB01	2.140.10.10	4.10.160.10
2d1pB	2d1pB01_2d1pA01	3.40.1260.10	3.40.1260.10
2d1pB	2d1pB01_2d1pC01	3.40.1260.10	3.40.1260.10
2d1pG	2d1pG01_2d1pH01	3.40.1260.10	3.40.1260.10
2d1pG	2d1pG01_2d1pI01	3.40.1260.10	3.40.1260.10
2d1pH	2d1pH01_2d1pF01	3.40.1260.10	3.40.1260.10
2d1pI	2d1pI01_2d1pE01	3.40.1260.10	3.40.1260.10
2d1pI	2d1pI01_2d1pH01	3.40.1260.10	3.40.1260.10
2dd4I	2dd4I01_2dd4G01	3.90.330.10	2.30.30.50
2dfkA	2dfkA01_2dfkB01	1.20.900.10	3.40.50.300
2dfkB	2dfkB01_2dfkC02	3.40.50.300	2.30.29.30

2dfkD	2dfkD01_2dfkC01	3.40.50.300	1.20.900.10
2dvwA	2dvwA01_2dvwB01	1.25.40.20	1.10.8.60
2e31A	2e31A01_2e31B01	2.60.120.260	3.30.710.10
2e4mA	2e4mA01_2e4mC01	2.80.10.50	2.80.10.50
2e4mC	2e4mC01_2e4mB01	2.80.10.50	2.80.10.50
2e7lA	2e7lA01_2e7lD01	2.60.40.10	2.60.40.10
2eijU	2eijU01_2eijO02	1.10.10.140	2.60.40.420
2eizA	2eizA01_2eizB01	2.60.40.10	2.60.40.10
2eizA	2eizA01_2eizC01	2.60.40.10	1.10.530.10
2ejfA	2ejfA01_2ejfC01	3.30.930.10	2.40.50.100
2ejfD	2ejfD01_2ejfB01	2.40.50.100	3.30.930.10
2ekeA	2ekeA01_2ekeC01	3.10.110.10	3.10.20.90
2eksB	2eksB01_2eksC01	2.60.40.10	1.10.530.10
2f2fE	2f2fE01_2f2fD01	3.60.10.10	2.80.10.50
2f2fE	2f2fE01_2f2fF01	3.60.10.10	2.80.10.50
2fjuA	2fjuA01_2fjuB01	3.40.50.300	3.20.20.190
2fm8B	2fm8B01_2fm8C01	3.30.1460.10	1.10.4150.10
2fmmA	2fmmA01_2fmmC01	2.40.50.40	2.40.50.40
2fmmB	2fmmB01_2fmmA01	2.40.50.40	2.40.50.40
2fu5A	2fu5A01_2fu5D01	2.170.150.10	3.40.50.300
2fu5C	2fu5C01_2fu5B01	3.40.50.300	2.170.150.10
2fyuJ	2fyuJ01_2fyuK01	1.20.5.260	1.20.5.220
2g2uA	2g2uA01_2g2uB01	3.40.710.10	3.30.1450.10
2g2uA	2g2uA01_2g2uB02	3.40.710.10	3.30.1450.10
2g2wB	2g2wB01_2g2wA01	3.30.1450.10	3.40.710.10
2ga4A	2ga4A02_2ga4C01	4.10.470.10	2.40.50.70
2ga4A	2ga4A02_2ga4E01	4.10.470.10	2.40.50.70
2ga4A	2ga4A02_2ga4F01	4.10.470.10	2.40.50.70
2gc7C	2gc7C01_2gc7D01	2.60.40.420	1.10.760.10
2gc7P	2gc7P01_2gc7O01	1.10.760.10	2.60.40.420
2gmiA	2gmiA01_2gmiB01	3.10.110.10	3.10.110.10
2golA	2golA01_2golB01	1.10.150.90	1.10.375.10
2gooD	2gooD01_2gooE01	2.10.90.10	2.10.60.10
2gooD	2gooD01_2gooF01	2.10.90.10	2.10.60.10
2guzI	2guzI01_2guzJ01	1.10.287.110	1.10.287.110
2guzN	2guzN01_2guzM01	1.10.287.110	1.10.287.110
2h26B	2h26B01_2h26A01	2.60.40.10	3.30.500.10
2h26B	2h26B01_2h26A02	2.60.40.10	2.60.40.10
2h62A	2h62A01_2h62D01	2.10.90.10	2.10.60.10
2h64C	2h64C01_2h64A01	2.10.60.10	2.10.90.10
2h7zA	2h7zA01_2h7zB01	2.10.60.10	2.10.60.10
2h89D	2h89D01_2h89B02	1.20.1300.10	1.10.1060.10
2h89D	2h89D01_2h89C02	1.20.1300.10	1.20.1300.10
2hd5B	2hd5B01_2hd5A01	3.10.20.90	3.90.70.10
2hdiA	2hdiA02_2hdiB01	2.40.170.20	3.30.305.10
2hleA	2hleA01_2hleB01	2.60.120.260	2.60.40.420
2htmC	2htmC01_2htmG01	3.20.20.70	3.10.20.30
2htmH	2htmH01_2htmD01	3.10.20.30	3.20.20.70
2hueA	2hueA01_2hueB01	2.60.40.1490	1.10.20.10

2hueA	2hueA01_2hueC01	2.60.40.1490	1.10.20.10
2hybG	2hybG01_2hybH01	3.40.1260.10	3.40.1260.10
2hybG	2hybG01_2hybI01	3.40.1260.10	3.40.1260.10
2hybH	2hybH01_2hybI01	3.40.1260.10	3.40.1260.10
2hybH	2hybH01_2hybL01	3.40.1260.10	3.40.1260.10
2hybR	2hybR01_2hybN01	3.40.1260.10	3.40.1260.10
2hybR	2hybR01_2hybP01	3.40.1260.10	3.40.1260.10
2hybR	2hybR01_2hybQ01	3.40.1260.10	3.40.1260.10
2iadA	2iadA01_2iadB01	3.10.320.10	3.10.320.10
2iadA	2iadA02_2iadB01	2.60.40.10	3.10.320.10
2ialA	2ialA01_2ialB01	2.60.40.10	2.60.40.10
2ialA	2ialA02_2ialB02	2.60.40.10	2.60.40.10
2ibgA	2ibgA01_2ibgF01	2.60.40.10	3.30.1380.10
2ibgE	2ibgE01_2ibgB01	3.30.1380.10	2.60.40.10
2icwH	2icwH01_2icwL01	1.20.120.390	2.60.40.10
2icwH	2icwH02_2icwL01	1.10.10.530	2.60.40.10
2idoC	2idoC01_2idoD01	3.30.420.10	1.20.58.250
2inpD	2inpD01_2inpB01	1.10.620.20	1.10.620.20
2iwtA	2iwtA01_2iwtB01	3.40.30.10	2.80.10.50
2ix2A	2ix2A01_2ix2B01	3.70.10.10	3.70.10.10
2ix2A	2ix2A01_2ix2C01	3.70.10.10	3.70.10.10
2j12A	2j12A01_2j12B01	2.60.90.10	2.60.40.10
2j2zA	2j2zA01_2j2zB01	2.60.40.10	2.60.40.1090
2j3tA	2j3tA01_2j3tB01	3.30.1380.20	3.30.1380.20
2j3tA	2j3tA01_2j3tC01	3.30.1380.20	3.30.450.70
2j3tB	2j3tB01_2j3tC01	3.30.1380.20	3.30.450.70
2j3wA	2j3wA01_2j3wB01	3.30.450.70	3.30.1380.20
2j3wB	2j3wB01_2j3wE01	3.30.1380.20	3.30.1380.20
2j8xA	2j8xA01_2j8xB01	3.40.470.10	3.10.450.20
2j8xD	2j8xD01_2j8xC01	3.10.450.20	3.40.470.10
2j9fB	2j9fB01_2j9fC01	3.40.50.970	3.40.50.970
2j9fB	2j9fB02_2j9fC01	3.40.50.920	3.40.50.970
2jbgC	2jbgC01_2jbgD01	1.10.1200.20	3.90.540.10
2jccE	2jccE01_2jccA01	2.60.40.10	3.30.500.10
2jeaA	2jeaA01_2jeaB01	3.30.230.70	3.30.230.70
2jg8B	2jg8B01_2jg8A01	2.60.120.40	2.60.120.40
2jg8B	2jg8B01_2jg8C01	2.60.120.40	2.60.120.40
2jg8C	2jg8C01_2jg8A01	2.60.120.40	2.60.120.40
2jg9A	2jg9A01_2jg9B01	2.60.120.40	2.60.120.40
2jg9A	2jg9A01_2jg9C01	2.60.120.40	2.60.120.40
2jjsB	2jjsB01_2jjsD01	2.60.40.10	2.60.40.10
2npsA	2npsA01_2npsB01	1.20.5.110	1.20.5.110
2npsA	2npsA01_2npsD01	1.20.5.110	1.20.5.110
2nptA	2nptA01_2nptD01	3.10.20.90	3.10.20.90
2nq2A	2nq2A01_2nq2B01	1.10.3470.10	1.10.3470.10
2ns1B	2ns1B01_2ns1A01	3.30.70.120	1.10.3430.10
2ntiE	2ntiE01_2ntiF01	3.70.10.10	3.70.10.10
2ntiF	2ntiF01_2ntiD01	3.70.10.10	3.70.10.10
2nuuD	2nuuD01_2nuuJ01	1.10.3430.10	3.30.70.120

2nuuD	2nuuD01_2nuuL01	1.10.3430.10	3.30.70.120
2nxnB	2nxnB01_2nxnA01	3.30.1550.10	3.30.70.1170
2nxnB	2nxnB02_2nxnA01	1.10.10.250	3.30.70.1170
2nxyA	2nxyA01_2nxyD01	2.170.40.20	2.60.40.10
2ny4D	2ny4D01_2ny4A01	2.60.40.10	2.170.40.20
2nz1A	2nz1A02_2nz1D01	2.60.40.1340	2.40.50.40
2nz8A	2nz8A01_2nz8B01	3.40.50.300	1.20.900.10
2o2vA	2o2vA01_2o2vB01	3.10.20.90	3.10.20.90
2omzA	2omzA01_2omzB01	3.80.10.10	2.60.40.60
2otuA	2otuA01_2otuB01	2.60.40.10	2.60.40.10
2ozlB	2ozlB01_2ozlA01	3.40.50.970	3.40.50.970
2ozlB	2ozlB01_2ozlC01	3.40.50.970	3.40.50.970
2ozlB	2ozlB02_2ozlC01	3.40.50.920	3.40.50.970
2oznA	2oznA01_2oznB02	2.60.40.680	1.20.1270.90
2p1oA	2p1oA01_2p1oB01	3.30.710.10	1.20.1280.50
2p1oA	2p1oA01_2p1oB02	3.30.710.10	1.20.1280.50
2p1qA	2p1qA01_2p1qB01	3.30.710.10	1.20.1280.50
2p1qA	2p1qA01_2p1qB02	3.30.710.10	1.20.1280.50
2p5eA	2p5eA01_2p5eE01	3.30.500.10	2.60.40.10
2p5eE	2p5eE01_2p5eD01	2.60.40.10	2.60.40.10
2p5eE	2p5eE02_2p5eD02	2.60.40.10	2.60.40.10
2pbiA	2pbiA01_2pbiB01	1.10.10.10	2.130.10.10
2pbiA	2pbiA02_2pbiB01	1.10.10.10	2.130.10.10
2pbiA	2pbiA03_2pbiB01	1.10.10.10	2.130.10.10
2pbiA	2pbiA04_2pbiB01	1.10.10.10	2.130.10.10
2pbiD	2pbiD01_2pbiC01	2.130.10.10	1.10.10.10
2pbiD	2pbiD01_2pbiC02	2.130.10.10	1.10.10.10
2pbiD	2pbiD01_2pbiC03	2.130.10.10	1.10.10.10
2pbiD	2pbiD01_2pbiC04	2.130.10.10	1.10.10.10
2pe6A	2pe6A01_2pe6B01	3.10.110.10	3.10.20.90
2pi2A	2pi2A01_2pi2E01	2.40.50.140	2.40.50.140
2pi2F	2pi2F01_2pi2B01	2.40.50.140	2.40.50.140
2pkaB	2pkaB01_2pkaA01	2.40.10.10	2.40.10.10
2po0B	2po0B01_2po0A01	3.30.230.70	3.30.230.70
2pttA	2pttA01_2pttB01	2.60.40.10	2.60.40.10
2pu9A	2pu9A01_2pu9B01	3.90.460.10	2.30.30.50
2pu9A	2pu9A01_2pu9C01	3.90.460.10	3.40.30.10
2pvgA	2pvgA01_2pvgC01	3.90.460.10	3.10.20.30
2pxyA	2pxyA01_2pxyB01	2.60.40.10	2.60.40.10
2q0oB	2q0oB02_2q0oD01	1.10.10.10	1.10.287.160
2q86C	2q86C01_2q86D01	2.60.40.10	2.60.40.10
2q86C	2q86C02_2q86D02	2.60.40.10	2.60.40.10
2qieH	2qieH01_2qieG01	3.90.1170.40	3.10.20.30
2qjbA	2qjbA01_2qjbD01	2.10.90.10	2.10.60.10
2qk7A	2qk7A01_2qk7B01	2.70.240.10	2.70.240.10
2ql2A	2ql2A01_2ql2B01	4.10.280.10	4.10.280.10
2qqdE	2qqdE01_2qqdD01	3.50.20.10	3.30.60.30
2r1hC	2r1hC01_2r1hB01	1.10.490.10	1.10.490.10
2r1hC	2r1hC01_2r1hD01	1.10.490.10	1.10.490.10

2r1hD	2r1hD01_2r1hA01	1.10.490.10	1.10.490.10
2r25A	2r25A01_2r25B01	1.20.120.160	3.40.50.2300
2rexC	2rexC01_2rexD01	3.10.20.90	3.40.50.300
2uyzB	2uyzB01_2uyzA01	3.10.20.90	3.10.110.10
2v4iE	2v4iE01_2v4iH02	3.60.70.12	3.30.2330.10
2v9tA	2v9tA01_2v9tB01	2.60.40.10	3.80.10.10
2vjhA	2vjhA01_2vjhB01	1.10.490.20	1.10.490.20
2vjtA	2vjtA01_2vjtB01	1.10.490.20	1.10.490.20
2vloA	2vloA01_2vloB01	1.10.1200.20	3.90.540.10
2vlrF	2vlrF01_2vlrJ01	3.30.500.10	2.60.40.10
2vmlA	2vmlA01_2vmlB01	1.10.490.20	1.10.490.20
2vmlA	2vmlA01_2vmlF01	1.10.490.20	1.10.490.20
2vmlD	2vmlD01_2vmlC01	1.10.490.20	1.10.490.20
2vmlD	2vmlD01_2vmlE01	1.10.490.20	1.10.490.20
2vq1A	2vq1A01_2vq1B01	2.60.40.10	2.60.40.10
2vq1A	2vq1A02_2vq1B02	2.60.40.10	2.60.40.10
2vyrA	2vyrA01_2vyrE01	1.10.245.10	2.60.40.10
2vyrH	2vyrH01_2vyrD01	2.60.40.10	1.10.245.10
2vzkB	2vzkB01_2vzkA01	3.30.2330.10	3.60.70.12
2w07A	2w07A01_2w07B01	2.60.40.10	2.60.40.1090
2w2xB	2w2xB01_2w2xC01	3.40.50.300	2.30.29.30
2w83B	2w83B01_2w83C01	3.40.50.300	1.20.5.1000
2wbwA	2wbwA01_2wbwB01	2.60.90.10	2.60.40.10
2wo3A	2wo3A01_2wo3B01	2.60.120.260	2.60.40.420
2wptA	2wptA01_2wptB01	1.10.1200.20	3.90.540.10
2wqyC	2wqyC01_2wqyB01	1.20.5.540	3.10.20.30
2wqyC	2wqyC01_2wqyB02	1.20.5.540	1.10.1060.10
2wqyC	2wqyC02_2wqyB02	1.20.1300.10	1.10.1060.10
2wqyC	2wqyC02_2wqyD01	1.20.1300.10	1.20.1300.10
2x89B	2x89B01_2x89D01	2.60.40.10	2.60.40.10
2x89F	2x89F01_2x89B01	2.60.40.10	2.60.40.10
2xcmD	2xcmD01_2xcmA01	2.60.40.790	3.30.565.10
2xfgA	2xfgA01_2xfgB01	1.50.10.10	2.60.40.710
2xfxA	2xfxA01_2xfxB01	3.30.500.10	2.60.40.10
2xfxA	2xfxA02_2xfxB01	2.60.40.10	2.60.40.10
2xn9B	2xn9B01_2xn9A01	2.60.40.10	2.60.40.10
2xn9B	2xn9B02_2xn9A02	2.60.40.10	2.60.40.10
2xqwB	2xqwB01_2xqwC02	1.50.10.20	2.10.70.10
2xwtA	2xwtA01_2xwtB01	2.60.40.10	2.60.40.10
2xwtA	2xwtA01_2xwtC01	2.60.40.10	3.80.10.10
2xwtA	2xwtA02_2xwtB02	2.60.40.10	2.60.40.10
2xwtB	2xwtB01_2xwtC01	2.60.40.10	3.80.10.10
2y1lA	2y1lA01_2y1lF01	3.40.50.1460	1.25.40.20
2y69B	2y69B01_2y69A01	1.10.287.90	1.20.210.10
2y69B	2y69B01_2y69I01	1.10.287.90	4.10.93.10
2y69B	2y69B02_2y69A01	2.60.40.420	1.20.210.10
2y69B	2y69B02_2y69D01	2.60.40.420	1.10.442.10
2y69B	2y69B02_2y69H01	2.60.40.420	1.10.10.140
2y69B	2y69B02_2y69I01	2.60.40.420	4.10.93.10

2ybvE	2ybvE02_2ybvD01	3.20.20.110	3.30.190.10
2ybvE	2ybvE02_2ybvF01	3.20.20.110	3.30.190.10
2ybvF	2ybvF01_2ybvG02	3.30.190.10	3.20.20.110
2yc1D	2yc1D01_2yc1E01	2.60.40.10	2.60.40.10
2yc1D	2yc1D01_2yc1F01	2.60.40.10	3.30.30.10
2yclA	2yclA01_2yclB01	3.20.20.20	3.20.20.20
2yevB	2yevB01_2yevA01	1.10.287.90	1.20.210.10
2yevB	2yevB02_2yevA01	1.10.287.90	1.20.210.10
2yevD	2yevD01_2yevE01	1.20.210.10	1.10.287.90
2yevD	2yevD01_2yevE02	1.20.210.10	1.10.287.90
2yhoB	2yhoB01_2yhoA01	3.10.110.10	3.30.40.10
2yhoG	2yhoG01_2yhoH01	3.30.40.10	3.10.110.10
2ynmA	2ynmA01_2ynmD01	3.40.50.300	3.40.50.1980
2ynmB	2ynmB01_2ynmD01	3.40.50.300	3.40.50.1980
2yplD	2yplD01_2yplE01	2.60.40.10	2.60.40.10
2yplD	2yplD02_2yplE02	2.60.40.10	2.60.40.10
2yssC	2yssC01_2yssB01	1.10.530.10	2.60.40.10
2z58B	2z58B01_2z58A01	3.30.70.80	3.40.50.200
2z6nB	2z6nB01_2z6nA01	1.10.490.10	1.10.490.10
2z93C	2z93C01_2z93D01	2.60.40.10	2.60.40.10
2z93C	2z93C02_2z93D02	2.60.40.10	2.60.40.10
2zfdA	2zfdA01_2zfdB01	1.10.238.10	3.30.310.80
2zfoA	2zfoA01_2zfoC01	1.10.490.10	1.10.490.10
2zfoA	2zfoA01_2zfoD01	1.10.490.10	1.10.490.10
2zs0C	2zs0C01_2zs0A01	1.10.490.10	1.10.490.10
2zs0C	2zs0C01_2zs0B01	1.10.490.10	1.10.490.10
2zs0D	2zs0D01_2zs0B01	1.10.490.10	1.10.490.10
2zyzC	2zyzC01_2zyzB01	3.40.1350.10	3.40.1170.20
2zyzC	2zyzC01_2zyzD02	3.40.1350.10	3.40.1350.10
2zyzD	2zyzD01_2zyzA01	3.40.1170.20	3.40.1350.10
3a1pA	3a1pA02_3a1pB01	2.30.30.240	3.30.860.10
3a33B	3a33B01_3a33A01	3.10.20.90	3.10.110.10
3a5zC	3a5zC01_3a5zD01	3.30.930.10	2.30.30.30
3aazA	3aazA01_3aazB01	2.60.40.10	2.60.40.10
3aazA	3aazA02_3aazB02	2.60.40.10	2.60.40.10
3abkS	3abkS01_3abkN01	2.60.11.10	1.20.210.10
3abkS	3abkS01_3abkP02	2.60.11.10	1.20.120.80
3ajiB	3ajiB01_3ajiA01	1.10.8.60	1.25.40.20
3at5B	3at5B01_3at5A01	1.10.490.10	1.10.490.10
3at6A	3at6A01_3at6B01	1.10.490.10	1.10.490.10
3aujA	3aujA01_3aujB01	3.20.20.350	3.40.50.10150
3axjA	3axjA01_3axjB01	1.20.58.190	1.20.58.190
3axjA	3axjA01_3axjB02	1.20.58.190	1.20.58.200
3axjA	3axjA02_3axjB01	1.20.58.200	1.20.58.190
3axyC	3axyC01_3axyA01	1.20.190.20	3.90.280.10
3axyH	3axyH01_3axyJ01	3.90.280.10	1.20.190.20
3ayzB	3ayzB01_3ayzA01	3.40.50.700	1.10.645.10
3ayzB	3ayzB01_3ayzC01	3.40.50.700	1.10.645.10
3ayzB	3ayzB02_3ayzA01	4.10.480.10	1.10.645.10

3ayzC	3ayzC01_3ayzD01	1.10.645.10	3.40.50.700
3ayzC	3ayzC01_3ayzD02	1.10.645.10	4.10.480.10
3b4vD	3b4vD01_3b4vB01	3.30.60.30	2.10.90.10
3b4vD	3b4vD03_3b4vA01	3.30.60.30	2.10.90.10
3b4vE	3b4vE01_3b4vG01	2.10.90.10	3.30.60.30
3b4vE	3b4vE01_3b4vH03	2.10.90.10	3.30.60.30
3b5nA	3b5nA01_3b5nB01	1.20.5.110	1.20.5.110
3b5nA	3b5nA01_3b5nD01	1.20.5.110	1.20.5.110
3b5nB	3b5nB01_3b5nC01	1.20.5.110	1.20.5.110
3b5nC	3b5nC01_3b5nD01	1.20.5.110	1.20.5.110
3bcqB	3bcqB01_3bcqA01	1.10.490.10	1.10.490.10
3bcqB	3bcqB01_3bcqC01	1.10.490.10	1.10.490.10
3bcqC	3bcqC01_3bcqD01	1.10.490.10	1.10.490.10
3bj2D	3bj2D01_3bj2A01	1.10.490.10	1.10.490.10
3bj2D	3bj2D01_3bj2C01	1.10.490.10	1.10.490.10
3bj3C	3bj3C01_3bj3B01	1.10.490.10	1.10.490.10
3bj3C	3bj3C01_3bj3D01	1.10.490.10	1.10.490.10
3bn3A	3bn3A01_3bn3B01	3.40.50.410	2.60.40.10
3bp5B	3bp5B01_3bp5A01	2.60.40.10	2.60.40.10
3bs5A	3bs5A01_3bs5B01	1.10.150.50	1.10.150.50
3bx1B	3bx1B01_3bx1C01	3.40.50.200	2.80.10.50
3bx1B	3bx1B01_3bx1D01	3.40.50.200	2.80.10.50
3bx1D	3bx1D01_3bx1A01	2.80.10.50	3.40.50.200
3bx4C	3bx4C01_3bx4D01	3.10.100.10	3.10.100.10
3bzeD	3bzeD01_3bzeC01	2.60.40.10	3.30.500.10
3bzeD	3bzeD01_3bzeC02	2.60.40.10	2.60.40.10
3bzfA	3bzfA01_3bzfB01	3.30.500.10	2.60.40.10
3bzfA	3bzfA02_3bzfB01	2.60.40.10	2.60.40.10
3c05A	3c05A01_3c05B01	4.10.70.10	4.10.70.10
3c5sB	3c5sB01_3c5sA01	2.60.40.10	2.60.40.10
3c5sB	3c5sB02_3c5sA02	2.60.40.10	2.60.40.10
3c6sG	3c6sG01_3c6sH01	2.60.40.10	2.60.40.10
3c6sG	3c6sG02_3c6sH02	2.60.40.10	2.60.40.10
3c7vD	3c7vD01_3c7vC01	3.30.1450.10	3.40.710.10
3c7vD	3c7vD02_3c7vC01	3.30.1450.10	3.40.710.10
3cjiA	3cjiA01_3cjiB01	2.60.120.20	2.60.120.20
3cjiA	3cjiA01_3cjiC01	2.60.120.20	2.60.120.20
3cjiB	3cjiB01_3cjiC01	2.60.120.20	2.60.120.20
3cjkA	3cjkA01_3cjkB01	3.30.70.100	3.30.70.100
3cjtA	3cjtA01_3cjtB01	3.30.70.1170	3.30.1550.10
3cjtA	3cjtA02_3cjtB01	3.40.50.150	3.30.1550.10
3cjtC	3cjtC02_3cjtJ02	3.40.50.150	3.30.1550.10
3ckiA	3ckiA01_3ckiB01	3.40.390.10	2.40.50.120
3cx5A	3cx5A01_3cx5B02	3.30.830.10	3.30.830.10
3cx5A	3cx5A02_3cx5B01	3.30.830.10	3.30.830.10
3cx5A	3cx5A02_3cx5C01	3.30.830.10	1.20.810.10
3cx5A	3cx5A02_3cx5H01	3.30.830.10	1.20.5.210
3cx5C	3cx5C01_3cx5D01	1.20.810.10	1.10.760.10
3cx5C	3cx5C01_3cx5D02	1.20.810.10	1.20.5.100

3cx5C	3cx5C01_3cx5E01	1.20.810.10	1.20.5.270
3cx5C	3cx5C01_3cx5G01	1.20.810.10	1.10.1090.10
3cx5C	3cx5C01_3cx5H01	1.20.810.10	1.20.5.210
3cx5C	3cx5C01_3cx5P02	1.20.810.10	2.102.10.10
3cx5T	3cx5T01_3cx5O01	1.20.5.260	1.10.760.10
3cx5T	3cx5T01_3cx5P01	1.20.5.260	1.20.5.270
3cxhE	3cxhE02_3cxhN01	2.102.10.10	1.20.810.10
3cxhJ	3cxhJ01_3cxhE02	2.60.40.10	2.102.10.10
3cxhJ	3cxhJ01_3cxhK01	2.60.40.10	2.60.40.10
3cxhO	3cxhO01_3cxhW01	1.10.760.10	1.10.760.10
3cxhS	3cxhS01_3cxhL02	1.20.5.210	3.30.830.10
3cxhS	3cxhS01_3cxhN01	1.20.5.210	1.20.810.10
3cxhS	3cxhS01_3cxhO02	1.20.5.210	1.20.5.100
3cxhS	3cxhS01_3cxhP01	1.20.5.210	1.20.5.270
3cxhS	3cxhS01_3cxhR01	1.20.5.210	1.10.1090.10
3d2uA	3d2uA01_3d2uB01	3.30.500.10	2.60.40.10
3d2uA	3d2uA02_3d2uB01	2.60.40.10	2.60.40.10
3d5rA	3d5rA01_3d5rB01	1.50.10.20	1.50.10.20
3dbxA	3dbxA01_3dbxB01	3.30.500.10	2.60.40.10
3dbxA	3dbxA02_3dbxB01	2.60.40.10	2.60.40.10
3dggA	3dggA01_3dggD01	2.60.40.10	2.60.40.10
3dhiA	3dhiA01_3dhiB01	1.10.620.20	1.10.620.20
3dhiA	3dhiA01_3dhiC01	1.10.620.20	3.10.20.270
3dhrB	3dhrB01_3dhrC01	1.10.490.10	1.10.490.10
3draA	3draA01_3draB01	1.25.40.120	1.50.10.20
3dsnB	3dsnB01_3dsnD01	2.60.40.1310	2.60.40.10
3dufF	3dufF01_3dufG01	3.40.50.970	3.40.50.970
3dufF	3dufF02_3dufG01	3.40.50.920	3.40.50.970
3duuA	3duuA01_3duuB01	2.60.40.10	2.60.40.10
3dxrA	3dxrA01_3dxrB01	1.10.287.810	1.10.287.810
3e2kA	3e2kA01_3e2kD01	3.40.710.10	3.30.1450.10
3e2kA	3e2kA01_3e2kD02	3.40.710.10	3.30.1450.10
3e2kC	3e2kC01_3e2kB01	3.30.1450.10	3.40.710.10
3e2kC	3e2kC02_3e2kB01	3.30.1450.10	3.40.710.10
3e32A	3e32A01_3e32B01	1.25.40.120	1.50.10.20
3e95A	3e95A01_3e95C01	3.10.110.10	3.10.110.10
3e95B	3e95B01_3e95C01	3.10.110.10	3.10.110.10
3ebaA	3ebaA01_3ebaB01	2.60.40.10	1.10.530.10
3ej3B	3ej3B01_3ej3E01	3.30.429.10	3.30.429.10
3ej9E	3ej9E01_3ej9B01	3.30.429.10	3.30.429.10
3ex7B	3ex7B01_3ex7A01	3.30.70.330	3.30.1560.10
3ex7E	3ex7E01_3ex7G01	3.30.1560.10	3.30.70.330
3exeA	3exeA01_3exeB01	3.40.50.970	3.40.50.970
3exeA	3exeA01_3exeD01	3.40.50.970	3.40.50.970
3exeA	3exeA01_3exeD02	3.40.50.970	3.40.50.920
3eyoC	3eyoC01_3eyoD01	2.60.40.10	2.60.40.10
3eyoC	3eyoC02_3eyoD02	2.60.40.10	2.60.40.10
3f6qB	3f6qB01_3f6qA01	2.10.110.10	1.25.40.20
3fapA	3fapA01_3fapB01	3.10.50.40	1.20.120.150

3feyA	3feyA01_3feyC01	1.25.40.180	1.25.10.10
3feyA	3feyA02_3feyB01	1.25.40.180	3.30.70.330
3ff7A	3ff7A01_3ff7C01	2.60.40.60	3.10.100.10
3ff8A	3ff8A01_3ff8C01	2.60.40.60	3.10.100.10
3ff8D	3ff8D01_3ff8B01	3.10.100.10	2.60.40.60
3fgcA	3fgcA01_3fgcB01	3.20.20.30	3.20.20.30
3fgcA	3fgcA01_3fgcD01	3.20.20.30	3.20.20.30
3fgcD	3fgcD01_3fgcC01	3.20.20.30	3.20.20.30
3fm8A	3fm8A01_3fm8C02	2.60.200.20	2.30.29.30
3fo1A	3fo1A01_3fo1B01	2.60.40.10	2.60.40.10
3fo1A	3fo1A02_3fo1B02	2.60.40.10	2.60.40.10
3fo9B	3fo9B01_3fo9A01	2.60.40.10	2.60.40.10
3fo9B	3fo9B02_3fo9A02	2.60.40.10	2.60.40.10
3fruA	3fruA01_3fruB01	3.30.500.10	2.60.40.10
3fruA	3fruA02_3fruB01	2.60.40.10	2.60.40.10
3fruC	3fruC01_3fruF01	3.30.500.10	2.60.40.10
3g3bA	3g3bA01_3g3bB01	3.80.10.10	1.10.530.10
3g3bF	3g3bF01_3g3bE01	1.10.530.10	3.80.10.10
3g5oA	3g5oA02_3g5oB01	3.40.1620.10	3.30.2310.20
3g5oC	3g5oC01_3g5oD02	3.30.2310.20	3.40.1620.10
3g5xA	3g5xA01_3g5xD01	2.60.40.10	2.60.40.10
3g5xD	3g5xD01_3g5xC01	2.60.40.10	2.60.40.10
3g5xD	3g5xD02_3g5xC02	2.60.40.10	2.60.40.10
3g5yA	3g5yA01_3g5yB01	2.60.40.10	2.60.40.10
3g5yA	3g5yA02_3g5yB02	2.60.40.10	2.60.40.10
3gc3A	3gc3A01_3gc3B01	2.60.40.840	2.130.10.110
3gc3A	3gc3A02_3gc3B01	2.60.40.640	2.130.10.110
3gdjC	3gdjC01_3gdjB01	1.10.490.10	1.10.490.10
3gdjC	3gdjC01_3gdjD01	1.10.490.10	1.10.490.10
3gewC	3gewC01_3gewB01	2.60.40.10	2.60.40.10
3gfkB	3gfkB01_3gfkA01	1.10.150.20	3.40.30.10
3gjnC	3gjnC01_3gjnD01	3.90.540.10	1.10.1200.20
3gklD	3gklD01_3gklB01	1.10.1200.20	3.90.540.10
3gqiA	3gqiA02_3gqiB01	1.10.510.10	3.30.505.10
3h3bB	3h3bB01_3h3bC02	3.80.20.20	2.60.40.10
3h3bB	3h3bB01_3h3bD02	3.80.20.20	2.60.40.10
3h3bD	3h3bD02_3h3bA01	2.60.40.10	3.80.20.20
3h82B	3h82B01_3h82A01	3.30.450.20	3.30.450.20
3hb3C	3hb3C01_3hb3D01	2.60.40.10	2.60.40.10
3hctB	3hctB01_3hctA01	3.10.110.10	3.30.40.10
3hd8A	3hd8A02_3hd8B01	2.40.70.10	2.60.120.180
3hd8D	3hd8D01_3hd8C02	2.60.120.180	2.40.70.10
3heiI	3heiI01_3heiJ01	2.60.120.260	2.60.40.420
3heiN	3heiN01_3heiM01	2.60.40.420	2.60.120.260
3hhsA	3hhsA02_3hhsB02	1.10.1280.10	1.10.1280.10
3hrdD	3hrdD01_3hrdC01	3.10.20.30	3.30.390.50
3hrdG	3hrdG01_3hrdH01	3.30.390.50	3.10.20.30
3hrzB	3hrzB02_3hrzC02	2.60.40.10	2.60.40.690
3hujA	3hujA01_3hujG01	3.30.500.10	2.60.40.10

3hujH	3hujH01_3hujG01	2.60.40.10	2.60.40.10
3hujH	3hujH02_3hujG02	2.60.40.10	2.60.40.10
3hyuA	3hyuA01_3hyuB01	1.10.490.10	1.10.490.10
3hzhA	3hzhA01_3hzhB01	3.40.50.2300	3.40.1550.10
3hzvA	3hzvA01_3hzvB01	2.60.40.10	2.60.40.10
3hzvA	3hzvA02_3hzvB02	2.60.40.10	2.60.40.10
3i75A	3i75A01_3i75B01	2.60.40.10	2.60.40.10
3i75A	3i75A02_3i75B02	2.60.40.10	2.60.40.10
3imaA	3imaA01_3imaB01	3.90.70.10	3.10.450.10
3imaD	3imaD01_3imaC01	3.10.450.10	3.90.70.10
3ip4B	3ip4B01_3ip4A01	1.10.10.410	3.90.1300.10
3it4C	3it4C01_3it4B02	3.60.70.12	3.30.2330.10
3it4C	3it4C01_3it4D01	3.60.70.12	3.30.2330.10
3it6B	3it6B01_3it6A01	3.30.2330.10	3.60.70.12
3it6B	3it6B02_3it6C01	3.30.2330.10	3.60.70.12
3jvgB	3jvgB01_3jvgD01	3.30.500.10	2.60.40.10
3jvgB	3jvgB02_3jvgD01	2.60.40.10	2.60.40.10
3k1rA	3k1rA02_3k1rB01	2.30.42.10	1.10.150.50
3k2mA	3k2mA01_3k2mD01	3.30.505.10	2.60.40.10
3k74A	3k74A01_3k74B01	3.40.430.10	2.60.40.10
3k9oA	3k9oA02_3k9oB01	1.10.8.10	3.10.20.90
3kb3A	3kb3A01_3kb3B01	3.30.530.20	3.60.40.10
3kcpB	3kcpB01_3kcpA03	2.60.40.680	2.60.40.1120
3kdjA	3kdjA01_3kdjB01	3.30.530.20	3.60.40.10
3kdmA	3kdmA01_3kdmB01	2.60.40.10	2.60.40.10
3kdmA	3kdmA02_3kdmB02	2.60.40.10	2.60.40.10
3kfqB	3kfqB01_3kfqC01	3.90.70.10	3.10.450.10
3kfqD	3kfqD01_3kfqA01	3.10.450.10	3.90.70.10
3kldA	3kldA02_3kldB01	2.60.40.10	3.10.200.10
3kmwA	3kmwA02_3kmwB01	1.10.510.10	1.10.418.10
3kraC	3kraC01_3kraD01	1.10.600.10	1.10.600.10
3kseA	3kseA01_3kseF01	3.90.70.10	3.10.450.10
3kseC	3kseC01_3kseF01	3.90.70.10	3.10.450.10
3ktaD	3ktaD01_3ktaC01	3.40.50.300	3.40.50.300
3kucA	3kucA01_3kucB01	3.40.50.300	3.10.20.90
3kudA	3kudA01_3kudB01	3.40.50.300	3.10.20.90
3kyjA	3kyjA01_3kyjB01	1.20.120.160	3.40.50.2300
3l7eB	3l7eB01_3l7eA01	2.60.40.10	2.60.40.10
3l7eB	3l7eB02_3l7eA02	2.60.40.10	2.60.40.10
3l7zA	3l7zA01_3l7zB01	3.30.230.70	3.30.230.70
3l7zA	3l7zA01_3l7zE01	3.30.230.70	3.30.230.70
3l7zC	3l7zC01_3l7zB01	3.30.1370.10	3.30.230.70
3l7zC	3l7zC02_3l7zB01	2.40.50.140	3.30.230.70
3l7zE	3l7zE01_3l7zF01	3.30.230.70	3.30.1370.10
3l7zE	3l7zE01_3l7zF02	3.30.230.70	2.40.50.140
3l82A	3l82A01_3l82B01	1.25.40.210	3.40.50.300
3l9rE	3l9rE01_3l9rF01	3.30.500.10	2.60.40.10
3l9rE	3l9rE02_3l9rF01	2.60.40.10	2.60.40.10
3lcpB	3lcpB01_3lcpD01	2.60.120.200	1.10.238.10

3m18A	3m18A01_3m18B01	3.80.10.10	1.10.530.10
3m62A	3m62A01_3m62B01	3.30.40.10	3.10.20.90
3m63A	3m63A01_3m63B01	3.30.40.10	3.10.20.90
3m7fA	3m7fA01_3m7fB01	3.30.505.10	2.60.40.150
3m7nE	3m7nE01_3m7nB01	3.30.230.70	2.40.50.140
3m7nE	3m7nE01_3m7nG01	3.30.230.70	3.30.230.70
3m7nE	3m7nE01_3m7nH01	3.30.230.70	3.30.230.70
3mezA	3mezA01_3mezB01	2.90.10.10	2.90.10.10
3mffB	3mffB01_3mffA01	2.60.40.10	2.60.40.10
3mffB	3mffB02_3mffA02	2.60.40.10	2.60.40.10
3mffC	3mffC01_3mffD01	2.60.40.10	2.60.40.10
3mffC	3mffC02_3mffD02	2.60.40.10	2.60.40.10
3mhsA	3mhsA02_3mhsD01	3.90.70.10	3.10.20.90
3mi9A	3mi9A01_3mi9B01	3.30.200.20	1.10.472.10
3mkbA	3mkbA01_3mkbB01	1.10.490.10	1.10.490.10
3mkbA	3mkbA01_3mkbD01	1.10.490.10	1.10.490.10
3mkbB	3mkbB01_3mkbC01	1.10.490.10	1.10.490.10
3mnvB	3mnvB01_3mnvC01	2.60.40.10	2.60.40.10
3msxA	3msxA01_3msxB01	3.40.50.300	1.10.555.10
3mv8E	3mv8E01_3mv8D01	2.60.40.10	2.60.40.10
3mv8E	3mv8E02_3mv8D02	2.60.40.10	2.60.40.10
3myrC	3myrC01_3myrB01	3.40.50.700	1.10.645.10
3myrC	3myrC01_3myrD01	3.40.50.700	1.10.645.10
3myrC	3myrC02_3myrD01	4.10.480.10	1.10.645.10
3myrD	3myrD01_3myrA01	1.10.645.10	3.40.50.700
3n1fD	3n1fD01_3n1fA01	2.60.40.10	3.30.1380.10
3n1xB	3n1xB01_3n1xA01	1.10.620.20	1.10.620.20
3n3aB	3n3aB01_3n3aA01	1.10.620.20	1.10.620.20
3napA	3napA01_3napB01	2.60.120.20	2.60.120.20
3napA	3napA01_3napC01	2.60.120.20	2.60.120.20
3napB	3napB01_3napC01	2.60.120.20	2.60.120.20
3nceB	3nceB01_3nceA01	2.60.40.10	1.20.1250.10
3nceB	3nceB02_3nceA01	2.60.40.10	1.20.1250.10
3ndyF	3ndyF01_3ndyE01	2.60.40.290	2.60.40.290
3ng6C	3ng6C01_3ng6B01	1.10.490.10	1.10.490.10
3ng6C	3ng6C01_3ng6D01	1.10.490.10	1.10.490.10
3nheA	3nheA01_3nheB01	3.90.70.10	3.10.20.90
3nmnB	3nmnB01_3nmnA01	3.60.40.10	3.30.530.20
3nmvA	3nmvA01_3nmvB01	3.30.530.20	3.60.40.10
3npsB	3npsB01_3npsC01	2.60.40.10	2.60.40.10
3npsB	3npsB02_3npsC02	2.60.40.10	2.60.40.10
3nquB	3nquB01_3nquA01	1.10.20.10	1.10.20.10
3nv0A	3nv0A01_3nv0B01	3.10.450.50	3.10.450.50
3nvwB	3nvwB01_3nvwA01	3.30.465.10	3.10.20.30
3nvwB	3nvwB01_3nvwA02	3.30.465.10	1.10.150.120
3nvzA	3nvzA01_3nvzB01	3.10.20.30	3.30.43.10
3o2cA	3o2cA01_3o2cB01	1.10.490.20	1.10.490.20
3o5tB	3o5tB01_3o5tA01	3.30.70.120	1.10.4080.10
3o9lD	3o9lD01_3o9lC01	2.40.70.10	2.40.70.10

3oabD	3oabD01_3oabC01	1.10.600.10	1.10.600.10
3oagC	3oagC01_3oagD01	2.40.70.10	2.40.70.10
3oeiB	3oeiB02_3oeiD01	3.40.1620.10	3.30.2310.20
3oj2A	3oj2A01_3oj2C01	2.80.10.50	2.60.40.10
3oj2A	3oj2A01_3oj2C02	2.80.10.50	2.60.40.10
3ojdA	3ojdA01_3ojdB01	2.60.40.10	2.60.40.10
3ojdA	3ojdA02_3ojdB02	2.60.40.10	2.60.40.10
3okyA	3okyA01_3okyB01	2.130.10.10	2.130.10.10
3onlA	3onlA01_3onlC01	1.25.40.90	1.20.58.400
3oq3A	3oq3A01_3oq3B01	1.20.1250.10	2.60.40.10
3oq3A	3oq3A01_3oq3B02	1.20.1250.10	2.60.40.10
3oq3A	3oq3A01_3oq3B03	1.20.1250.10	2.60.40.10
3p73A	3p73A01_3p73B01	3.30.500.10	2.60.40.10
3p73A	3p73A02_3p73B01	2.60.40.10	2.60.40.10
3p9wB	3p9wB01_3p9wA01	2.60.40.10	2.10.90.10
3p9wE	3p9wE01_3p9wF01	2.10.90.10	2.60.40.10
3pabA	3pabA01_3pabB01	3.30.500.10	2.60.40.10
3pabA	3pabA02_3pabB01	2.60.40.10	2.60.40.10
3pdiC	3pdiC01_3pdiD01	3.40.50.1980	3.40.50.1980
3pdiH	3pdiH01_3pdiG01	3.40.50.1980	3.40.50.1980
3pnwO	3pnwO01_3pnwN01	2.30.30.140	2.60.40.10
3pnwT	3pnwT01_3pnwU01	2.60.40.10	2.30.30.140
3pnwW	3pnwW01_3pnwX01	2.60.40.10	2.30.30.140
3pt7A	3pt7A01_3pt7B01	1.10.490.10	1.10.490.10
3pt8B	3pt8B01_3pt8A01	1.10.490.10	1.10.490.10
3pv6A	3pv6A01_3pv6B01	2.60.40.10	2.60.40.10
3q3jA	3q3jA01_3q3jB01	3.10.20.90	3.40.50.300
3q3nC	3q3nC01_3q3nA01	3.10.20.270	1.10.620.20
3q73A	3q73A01_3q73B01	1.25.40.120	1.50.10.20
3q7fB	3q7fB01_3q7fA01	1.50.10.20	1.25.40.120
3q9nD	3q9nD01_3q9nB01	1.25.40.20	3.40.50.720
3q9uA	3q9uA01_3q9uC01	3.40.50.720	1.25.40.20
3qb4A	3qb4A01_3qb4D01	2.10.90.10	2.10.60.10
3qb4C	3qb4C01_3qb4D01	2.10.90.10	2.10.60.10
3qc8B	3qc8B01_3qc8A02	3.10.20.90	3.10.330.10
3qeuA	3qeuA01_3qeuB01	2.60.40.10	2.60.40.10
3qeuA	3qeuA02_3qeuB02	2.60.40.10	2.60.40.10
3qh3D	3qh3D02_3qh3A01	2.60.40.10	2.60.40.10
3qhtB	3qhtB01_3qhtC01	3.10.20.90	2.60.40.10
3qhtB	3qhtB01_3qhtD01	3.10.20.90	2.60.40.10
3qhyA	3qhyA01_3qhyB01	3.40.710.10	2.130.10.30
3qi9D	3qi9D01_3qi9C01	2.60.40.10	2.60.40.10
3qi9D	3qi9D02_3qi9C02	2.60.40.10	2.60.40.10
3qksA	3qksA01_3qksB01	3.40.50.300	3.40.50.300
3qksA	3qksA02_3qksB01	3.40.50.300	3.40.50.300
3qnyC	3qnyC01_3qnyD01	2.60.40.10	2.60.40.10
3qnyC	3qnyC02_3qnyD02	2.60.40.10	2.60.40.10
3qnzB	3qnzB01_3qnzA01	2.60.40.10	2.60.40.10
3qnzB	3qnzB02_3qnzA02	2.60.40.10	2.60.40.10

3qvgA	3qvgA01_3qvgB01	3.40.50.10190	3.40.50.10190
3qvgD	3qvgD01_3qvgC01	3.40.50.10190	3.40.50.10190
3qwzA	3qwzA01_3qwzB01	2.40.40.20	3.10.20.90
3qygC	3qygC01_3qygD01	3.90.330.10	1.10.472.20
3qygC	3qygC01_3qygD02	3.90.330.10	2.30.30.50
3qz5F	3qz5F01_3qz5E01	1.10.472.20	3.90.330.10
3qz5F	3qz5F02_3qz5E01	2.30.30.50	3.90.330.10
3rbbA	3rbbA01_3rbbB01	3.30.62.10	2.30.30.40
3rczA	3rczA01_3rczB01	3.10.20.90	3.10.110.10
3repB	3repB01_3repA02	1.10.418.10	1.10.510.10
3revA	3revA01_3revB01	2.60.40.10	2.60.40.10
3revA	3revA02_3revB02	2.60.40.10	2.60.40.10
3rneA	3rneA01_3rneB01	1.10.620.20	1.10.620.20
3rneA	3rneA01_3rneC01	1.10.620.20	3.10.20.270
3rnfC	3rnfC01_3rnfA01	3.10.20.270	1.10.620.20
3rnkA	3rnkA01_3rnkB01	2.60.40.10	2.60.40.10
3rpfB	3rpfB01_3rpfC01	3.90.1170.40	3.10.20.30
3rrlA	3rrlA01_3rrlB01	3.40.1080.10	3.40.1080.10
3rrlA	3rrlA01_3rrlD01	3.40.1080.10	3.40.1080.10
3rrlD	3rrlD01_3rrlC01	3.40.1080.10	3.40.1080.10
3ru4D	3ru4D01_3ru4B01	2.40.10.10	2.10.69.10
3ru4E	3ru4E01_3ru4B01	2.40.10.10	2.10.69.10
3rugC	3rugC01_3rugG01	3.30.500.10	2.60.40.10
3rugE	3rugE01_3rugF01	2.60.40.10	2.60.40.10
3rugE	3rugE02_3rugF02	2.60.40.10	2.60.40.10
3rzwA	3rzwA01_3rzwD01	2.60.40.10	3.10.20.90
3s96A	3s96A01_3s96B01	2.60.40.10	2.60.40.10
3s96A	3s96A02_3s96B02	2.60.40.10	2.60.40.10
3s97A	3s97A01_3s97C01	3.10.200.10	2.60.40.10
3s97A	3s97A01_3s97C02	3.10.200.10	2.60.40.10
3s97D	3s97D01_3s97B01	2.60.40.10	3.10.200.10
3s97D	3s97D02_3s97B01	2.60.40.10	3.10.200.10
3sdeA	3sdeA01_3sdeB01	3.30.70.330	3.30.70.330
3sdeA	3sdeA02_3sdeB02	3.30.70.330	3.30.70.330
3sdeA	3sdeA02_3sdeB03	3.30.70.330	3.30.70.330
3sdeA	3sdeA03_3sdeB02	3.30.70.330	3.30.70.330
3sdeA	3sdeA03_3sdeB03	3.30.70.330	3.30.70.330
3sdlB	3sdlB01_3sdlD01	1.10.287.10	3.10.20.90
3sdlC	3sdlC01_3sdlA01	3.10.20.90	1.10.287.10
3sqgC	3sqgC01_3sqgD02	3.90.320.20	3.30.70.470
3sqgD	3sqgD01_3sqgF01	3.90.390.10	3.90.320.20
3sqgD	3sqgD03_3sqgF01	1.20.840.10	3.90.320.20
3sqgI	3sqgI01_3sqgG01	3.90.320.20	3.90.390.10
3sqgI	3sqgI01_3sqgG03	3.90.320.20	1.20.840.10
3stbA	3stbA01_3stbD01	2.60.40.10	2.40.50.140
3stbB	3stbB01_3stbC01	2.60.40.10	2.40.50.140
3stbC	3stbC01_3stbD01	2.40.50.140	2.40.50.140
3svwC	3svwC01_3svwD01	2.60.30.10	2.130.10.10
3svwC	3svwC01_3svwF01	2.60.30.10	2.130.10.10

3swnB	3swnB01_3swnA01	2.30.30.100	2.30.30.100
3swnD	3swnD01_3swnE01	2.30.30.100	2.30.30.100
3t5gA	3t5gA01_3t5gB01	3.40.50.300	2.70.50.40
3tacA	3tacA02_3tacB01	1.10.510.10	1.10.150.50
3tclB	3tclB01_3tclA01	2.60.40.10	2.60.40.10
3tclB	3tclB02_3tclA02	2.60.40.10	2.60.40.10
3tdhA	3tdhA01_3tdhC01	3.30.310.80	3.10.580.10
3tsrA	3tsrA01_3tsrE01	3.10.130.10	3.80.10.10
3tsrH	3tsrH01_3tsrD01	3.80.10.10	3.10.130.10
3u0tB	3u0tB01_3u0tA01	2.60.40.10	2.60.40.10
3u0tB	3u0tB02_3u0tA02	2.60.40.10	2.60.40.10
3u52A	3u52A01_3u52C01	1.10.620.20	1.10.620.20
3ubuA	3ubuA01_3ubuB01	3.10.100.10	3.10.100.10
3uipA	3uipA01_3uipB01	3.10.110.10	3.10.20.90
3ulrA	3ulrA01_3ulrB01	1.10.530.10	2.30.30.40
3utzB	3utzB01_3utzA01	2.60.40.10	2.60.40.10
3utzB	3utzB02_3utzA02	2.60.40.10	2.60.40.10
3utzD	3utzD01_3utzC01	2.60.40.10	2.60.40.10
3utzD	3utzD02_3utzC02	2.60.40.10	2.60.40.10
3utzF	3utzF01_3utzE01	2.60.40.10	2.60.40.10
3utzF	3utzF02_3utzE02	2.60.40.10	2.60.40.10
3v6oB	3v6oB01_3v6oD01	2.60.40.10	2.60.40.10
3v6oC	3v6oC01_3v6oA01	2.60.40.10	2.60.40.10
3v7dB	3v7dB01_3v7dA01	1.20.1280.50	3.30.710.10
3vmfA	3vmfA02_3vmfB02	2.40.30.10	3.30.420.60
3vmfA	3vmfA03_3vmfB03	2.40.30.10	3.30.1330.30
3vreB	3vreB01_3vreC01	1.10.490.10	1.10.490.10
3vrfB	3vrfB01_3vrfA01	1.10.490.10	1.10.490.10
3vrgA	3vrgA01_3vrgB01	1.10.490.10	1.10.490.10
3vsgA	3vsgA01_3vsgB01	3.40.830.10	3.40.830.10
3vsiB	3vsiB01_3vsiA01	3.40.830.10	3.40.830.10
3vv2A	3vv2A01_3vv2B01	3.40.50.200	3.30.70.80
3vxmA	3vxmA01_3vxmD01	3.30.500.10	2.60.40.10
3vxmD	3vxmD01_3vxmE01	2.60.40.10	2.60.40.10
3vxmD	3vxmD02_3vxmE02	2.60.40.10	2.60.40.10
3vxqB	3vxqB01_3vxqA01	2.60.40.10	2.60.40.10
3vxqB	3vxqB02_3vxqA02	2.60.40.10	2.60.40.10
3vygG	3vygG01_3vygI01	2.30.30.50	3.90.330.10
3w4uC	3w4uC01_3w4uB01	1.10.490.10	1.10.490.10
3w4uD	3w4uD01_3w4uA01	1.10.490.10	1.10.490.10
3w4uF	3w4uF01_3w4uE01	1.10.490.10	1.10.490.10
3w9eA	3w9eA01_3w9eB01	2.60.40.10	2.60.40.10
3w9eA	3w9eA02_3w9eB02	2.60.40.10	2.60.40.10
3wcwC	3wcwC01_3wcwA01	1.10.490.10	1.10.490.10
3wcwC	3wcwC01_3wcwB01	1.10.490.10	1.10.490.10
3wg7D	3wg7D01_3wg7A01	1.10.442.10	1.20.210.10
3wg7D	3wg7D01_3wg7B02	1.10.442.10	2.60.40.420
3wg7D	3wg7D01_3wg7E01	1.10.442.10	1.25.40.40
3zkxA	3zkxA02_3zkxC01	2.40.70.10	2.60.40.10

3zniE	3zniE01_3zniO01	1.20.930.20	3.10.110.10
3zniE	3zniE04_3zniG01	3.30.40.10	3.10.110.10
3zniK	3zniK01_3zniA01	3.10.110.10	1.20.930.20
3zr4B	3zr4B01_3zr4A01	3.40.50.880	3.20.20.70
43c9C	43c9C01_43c9D01	2.60.40.10	2.60.40.10
4a63B	4a63B02_4a63A01	2.30.30.40	2.60.40.720
4a63G	4a63G01_4a63H02	2.60.40.720	2.30.30.40
4a69A	4a69A01_4a69C01	3.40.800.20	1.10.10.60
4a69A	4a69A01_4a69D01	3.40.800.20	1.10.10.60
4aahC	4aahC01_4aahD01	2.140.10.10	4.10.160.10
4aezA	4aezA01_4aezB01	2.130.10.10	3.30.900.10
4aezH	4aezH01_4aezG01	3.30.900.10	2.130.10.10
4atzA	4atzA01_4atzD01	2.60.90.10	1.25.40.20
4auqA	4auqA01_4auqB01	3.10.110.10	3.30.40.10
4b93A	4b93A01_4b93B01	3.30.450.50	1.25.40.20
4bd9A	4bd9A01_4bd9B01	3.40.630.10	4.10.410.10
4bemA	4bemA01_4bemJ01	1.20.20.10	1.20.120.610
4bemI	4bemI01_4bemJ01	1.20.20.10	1.20.120.610
4bfbA	4bfbA01_4bfbD01	2.40.70.10	2.60.40.10
4bfbD	4bfbD01_4bfbB02	2.60.40.10	2.40.70.10
4bmoB	4bmoB01_4bmoA01	3.40.50.360	1.10.620.20
4bmpA	4bmpA01_4bmpB01	1.10.620.20	3.40.50.360
4bq6B	4bq6B02_4bq6F01	2.60.40.10	3.40.1000.10
4bwsA	4bwsA01_4bwsC01	3.40.30.10	3.30.1490.40
4bwsF	4bwsF01_4bwsD01	3.30.1490.40	3.40.30.10
4bxoA	4bxoA01_4bxoB01	3.40.50.10130	1.10.150.20
4bxoA	4bxoA02_4bxoB02	1.10.150.20	1.10.150.20
4c58A	4c58A02_4c58B01	1.10.510.10	2.60.40.10
4c92D	4c92D01_4c92G01	2.30.30.100	2.30.30.100
4c92E	4c92E01_4c92F01	2.30.30.100	2.30.30.100
4c92E	4c92E01_4c92G01	2.30.30.100	2.30.30.100
4c9bA	4c9bA02_4c9bB01	3.40.50.300	1.25.40.180
4cadH	4cadH01_4cadG01	2.60.40.10	2.60.40.10
4cadH	4cadH02_4cadG02	2.60.40.10	2.60.40.10
4cayA	4cayA01_4cayB01	1.10.20.10	1.10.20.10
4cdcA	4cdcA01_4cdcB01	2.40.128.80	3.90.70.10
4cdcH	4cdcH01_4cdcG01	3.90.70.10	2.40.128.80
4cj0B	4cj0B01_4cj0A02	2.40.50.40	1.50.10.10
4cj1A	4cj1A02_4cj1B01	1.50.10.10	2.40.50.40
4cj2B	4cj2B01_4cj2C01	1.10.530.10	2.40.50.40
4cj2D	4cj2D01_4cj2A01	2.40.50.40	1.10.530.10
4cmmA	4cmmA01_4cmmB01	2.60.40.10	2.60.40.10
4cvnA	4cvnA01_4cvnF01	3.40.50.300	3.30.420.80
4cvnE	4cvnE01_4cvnB01	3.30.420.80	3.40.50.300
4cw7A	4cw7A01_4cw7B01	3.40.50.300	3.30.420.80
4d0nB	4d0nB01_4d0nA01	1.20.900.10	3.40.50.300
4d9rD	4d9rD01_4d9rE01	2.60.40.10	2.60.40.10
4d9rD	4d9rD02_4d9rE02	2.60.40.10	2.60.40.10
4didA	4didA01_4didB01	3.40.50.300	1.20.58.450

4djdE	4djdE01_4djdF01	3.20.20.20	3.20.20.20
4djdF	4djdF01_4djdB01	3.20.20.20	3.20.20.20
4drxE	4drxE01_4drxD03	1.25.40.20	1.10.287.600
4ehmA	4ehmA01_4ehmB01	1.25.40.120	1.50.10.20
4ei6B	4ei6B01_4ei6A01	2.60.40.10	2.60.40.10
4ei6B	4ei6B02_4ei6A02	2.60.40.10	2.60.40.10
4ei6D	4ei6D01_4ei6C01	2.60.40.10	2.60.40.10
4ei6D	4ei6D02_4ei6C02	2.60.40.10	2.60.40.10
4eigA	4eigA01_4eigB01	3.40.430.10	2.60.40.10
4eizD	4eizD01_4eizA01	2.60.40.10	3.40.430.10
4eizD	4eizD01_4eizB01	2.60.40.10	3.40.430.10
4ej1A	4ej1A01_4ej1C01	3.40.430.10	2.60.40.10
4ej1A	4ej1A01_4ej1D01	3.40.430.10	2.60.40.10
4elkC	4elkC01_4elkD01	2.60.40.10	2.60.40.10
4elkC	4elkC02_4elkD02	2.60.40.10	2.60.40.10
4eneC	4eneC01_4eneD01	2.60.40.10	2.60.40.10
4eneC	4eneC02_4eneD02	2.60.40.10	2.60.40.10
4eo5B	4eo5B01_4eo5A01	1.10.20.10	2.60.40.1490
4eo5C	4eo5C01_4eo5A01	1.10.20.10	2.60.40.1490
4eozC	4eozC01_4eozA01	3.30.710.10	3.30.710.10
4esaC	4esaC01_4esaB01	1.10.490.10	1.10.490.10
4esaC	4esaC01_4esaD01	1.10.490.10	1.10.490.10
4eukA	4eukA01_4eukB01	3.40.50.2300	1.20.120.160
4f0tA	4f0tA01_4f0tB01	1.10.490.20	1.10.490.20
4f0uA	4f0uA01_4f0uB01	1.10.490.20	1.10.490.20
4f0uA	4f0uA01_4f0uF01	1.10.490.20	1.10.490.20
4f0uD	4f0uD01_4f0uE01	1.10.490.20	1.10.490.20
4f33C	4f33C01_4f33H01	2.60.40.10	2.60.40.10
4f3lA	4f3lA01_4f3lB01	4.10.280.10	4.10.280.10
4f3lA	4f3lA01_4f3lB02	4.10.280.10	3.30.450.20
4f3lA	4f3lA02_4f3lB02	3.30.450.20	3.30.450.20
4f3lA	4f3lA03_4f3lB02	3.30.450.20	3.30.450.20
4f3lA	4f3lA03_4f3lB03	3.30.450.20	3.30.450.20
4f6tA	4f6tA01_4f6tB01	3.40.1160.10	3.40.1160.10
4ffvD	4ffvD01_4ffvC01	2.60.40.10	2.60.40.10
4ffvD	4ffvD02_4ffvC02	2.60.40.10	2.60.40.10
4fhrB	4fhrB01_4fhrA01	1.10.220.30	3.40.1550.10
4fm4E	4fm4E01_4fm4F01	3.90.330.10	1.10.472.20
4fm4E	4fm4E01_4fm4F02	3.90.330.10	2.30.30.50
4g0nB	4g0nB01_4g0nA01	3.10.20.90	3.40.50.300
4g4sF	4g4sF01_4g4sE01	3.60.20.10	3.60.20.10
4g4sF	4g4sF01_4g4sG01	3.60.20.10	3.60.20.10
4g4sF	4g4sF01_4g4sN01	3.60.20.10	3.60.20.10
4g4sG	4g4sG01_4g4sA01	3.60.20.10	3.60.20.10
4g51D	4g51D01_4g51A01	1.10.490.10	1.10.490.10
4g51D	4g51D01_4g51C01	1.10.490.10	1.10.490.10
4g6aC	4g6aC01_4g6aD01	2.60.40.10	2.60.40.10
4g6aC	4g6aC02_4g6aD02	2.60.40.10	2.60.40.10
4g8eA	4g8eA01_4g8eB01	2.60.40.10	2.60.40.10

4g8eA	4g8eA02_4g8eB02	2.60.40.10	2.60.40.10
4g8gD	4g8gD01_4g8gE01	2.60.40.10	2.60.40.10
4g8gD	4g8gD02_4g8gE02	2.60.40.10	2.60.40.10
4g9fE	4g9fE01_4g9fD01	2.60.40.10	2.60.40.10
4g9fE	4g9fE02_4g9fD02	2.60.40.10	2.60.40.10
4g9sA	4g9sA01_4g9sB01	1.10.530.10	2.60.120.380
4gafA	4gafA01_4gafB01	2.80.10.50	2.60.40.10
4gafA	4gafA01_4gafB02	2.80.10.50	2.60.40.10
4gafA	4gafA01_4gafB03	2.80.10.50	2.60.40.10
4gftA	4gftA01_4gftB01	1.10.238.10	2.60.40.10
4ghiB	4ghiB01_4ghiA01	3.30.450.20	3.30.450.20
4gipF	4gipF01_4gipE01	2.60.40.1690	2.60.40.1690
4gipF	4gipF01_4gipE03	2.60.40.1690	2.60.40.1690
4gipF	4gipF04_4gipE04	2.60.40.1690	2.60.40.1690
4gkzA	4gkzA01_4gkzB01	2.60.40.10	2.60.40.10
4gkzA	4gkzA02_4gkzB02	2.60.40.10	2.60.40.10
4gn3E	4gn3E01_4gn3F01	1.10.530.10	2.40.50.140
4gn5A	4gn5A01_4gn5D01	2.40.50.140	1.10.530.10
4gojB	4gojB01_4gojD01	3.40.50.300	2.70.50.40
4gojC	4gojC01_4gojA01	2.70.50.40	3.40.50.300
4grmC	4grmC01_4grmB02	2.60.40.10	2.60.40.10
4h0mB	4h0mB01_4h0mA01	1.10.490.20	1.10.490.20
4h0mB	4h0mB01_4h0mE01	1.10.490.20	1.10.490.20
4h0mW	4h0mW01_4h0mT01	1.10.490.20	1.10.490.20
4h0mW	4h0mW01_4h0mX01	1.10.490.20	1.10.490.20
4h10A	4h10A01_4h10B01	4.10.280.10	4.10.280.10
4h2lA	4h2lA01_4h2lB01	1.10.490.10	1.10.490.10
4h2uB	4h2uB01_4h2uD01	3.30.930.10	1.10.1200.10
4h2wC	4h2wC01_4h2wB01	1.10.1200.10	3.30.930.10
4h2xA	4h2xA01_4h2xD01	3.30.930.10	1.10.1200.10
4h5sA	4h5sA01_4h5sB01	2.60.40.10	2.60.40.10
4h6jA	4h6jA01_4h6jB01	3.30.450.20	3.30.450.20
4hawB	4hawB01_4hawA01	2.30.29.30	3.40.50.300
4hb4A	4hb4A01_4hb4B01	3.40.50.300	2.30.29.30
4hdiB	4hdiB01_4hdiA01	2.60.40.10	2.60.40.10
4hdiB	4hdiB02_4hdiA02	2.60.40.10	2.60.40.10
4hdqA	4hdqA01_4hdqB01	2.30.29.30	3.40.50.300
4hgmA	4hgmA01_4hgmB02	2.60.40.10	1.10.246.10
4hi8A	4hi8A01_4hi8B01	1.25.40.20	2.10.110.10
4hkxA	4hkxA01_4hkxB01	2.60.40.10	2.60.40.10
4hkxA	4hkxA02_4hkxB02	2.60.40.10	2.60.40.10
4hopD	4hopD01_4hopC01	2.30.42.10	2.30.42.10
4hr6B	4hr6B02_4hr6C01	4.10.470.10	2.80.10.50
4hr6B	4hr6B02_4hr6C02	4.10.470.10	2.80.10.50
4hrtD	4hrtD01_4hrtA01	1.10.490.10	1.10.490.10
4hrtD	4hrtD01_4hrtC01	1.10.490.10	1.10.490.10
4hrtE	4hrtE01_4hrtF01	1.10.490.10	1.10.490.10
4hrtE	4hrtE01_4hrtH01	1.10.490.10	1.10.490.10
4hx2A	4hx2A01_4hx2B01	3.40.50.200	3.30.350.10

4i0cB	4i0cB01_4i0cC01	1.10.530.10	2.60.40.10
4i0cD	4i0cD01_4i0cA01	2.60.40.10	1.10.530.10
4i0xF	4i0xF01_4i0xE01	1.10.287.1060	1.10.287.1060
4i0xK	4i0xK01_4i0xL01	1.10.287.1060	1.10.287.1060
4i13A	4i13A01_4i13B01	3.40.430.10	2.60.40.10
4i2xB	4i2xB01_4i2xA01	2.60.40.10	2.60.40.10
4i2xB	4i2xB01_4i2xE01	2.60.40.10	2.60.40.10
4i2xB	4i2xB02_4i2xA02	2.60.40.10	2.60.40.10
4i4tA	4i4tA01_4i4tB01	3.30.1330.20	3.40.50.1440
4i5bB	4i5bB02_4i5bD02	2.60.40.10	2.60.40.10
4i5bD	4i5bD01_4i5bE01	3.10.320.10	3.10.320.10
4i5bD	4i5bD02_4i5bE01	2.60.40.10	3.10.320.10
4i99B	4i99B01_4i99D01	3.40.50.300	1.10.10.580
4imkC	4imkC01_4imkB01	2.60.40.10	2.60.40.10
4imkC	4imkC02_4imkB02	2.60.40.10	2.60.40.10
4iv1A	4iv1A01_4iv1B01	2.60.120.20	2.60.120.20
4iv1A	4iv1A01_4iv1C01	2.60.120.20	2.60.120.20
4jamA	4jamA01_4jamB01	2.60.40.10	2.60.40.10
4jamA	4jamA02_4jamB02	2.60.40.10	2.60.40.10
4je4A	4je4A01_4je4B01	3.30.505.10	2.60.40.10
4jegA	4jegA01_4jegB01	3.30.505.10	2.60.40.10
4jj5A	4jj5A01_4jj5B01	2.60.40.10	2.60.40.10
4jj5A	4jj5A02_4jj5B02	2.60.40.10	2.60.40.10
4jkxB	4jkxB01_4jkxA02	2.80.10.50	4.10.470.10
4jkxB	4jkxB02_4jkxA02	2.80.10.50	4.10.470.10
4jn2B	4jn2B01_4jn2A01	2.60.40.10	2.60.40.10
4jn2B	4jn2B02_4jn2A02	2.60.40.10	2.60.40.10
4jx1A	4jx1A01_4jx1C01	1.10.630.10	3.10.20.30
4jx1G	4jx1G01_4jx1E01	3.10.20.30	1.10.630.10
4jznC	4jznC01_4jznD01	2.60.40.10	2.60.40.10
4jznC	4jznC02_4jznD02	2.60.40.10	2.60.40.10
4jzoB	4jzoB01_4jzoA01	2.60.40.10	2.60.40.10
4jzoB	4jzoB02_4jzoA02	2.60.40.10	2.60.40.10
4k6lA	4k6lA01_4k6lG01	2.40.50.110	3.90.210.10
4k6lB	4k6lB01_4k6lG01	2.40.50.110	3.90.210.10
4k6lF	4k6lF01_4k6lG01	3.60.10.10	3.90.210.10
4k81A	4k81A01_4k81B01	3.10.20.90	3.40.50.300
4k81G	4k81G02_4k81D01	3.10.20.90	3.40.50.300
4k81H	4k81H01_4k81G01	3.40.50.300	3.10.20.90
4kaxA	4kaxA01_4kaxB01	3.40.50.300	2.30.29.30
4kgqA	4kgqA01_4kgqD02	2.60.120.40	2.10.50.10
4khaA	4khaA03_4khaB01	1.10.20.10	1.10.20.10
4ki5C	4ki5C01_4ki5D01	2.60.40.10	2.60.40.10
4ki5C	4ki5C02_4ki5D02	2.60.40.10	2.60.40.10
4kpuA	4kpuA01_4kpuB01	3.40.50.620	3.40.50.620
4kpuA	4kpuA02_4kpuB01	3.40.50.1220	3.40.50.620
4kt3A	4kt3A01_4kt3B01	1.10.530.10	3.10.450.170
4kvgA	4kvgA01_4kvgB01	3.40.50.300	3.10.20.90
4l0pA	4l0pA01_4l0pB01	2.60.120.260	2.60.40.420

4l2iB	4l2iB01_4l2iA01	3.40.50.620	3.40.50.620
4l2iB	4l2iB01_4l2iA02	3.40.50.620	3.40.50.1220
4l9pB	4l9pB01_4l9pA01	1.50.10.20	1.25.40.120
4lfmB	4lfmB01_4lfmA01	3.40.1400.10	3.40.1400.10
4lfnA	4lfnA01_4lfnB01	3.40.1400.10	3.40.1400.10
4lgpB	4lgpB01_4lgpA01	2.60.40.10	3.40.420.10
4lgpC	4lgpC01_4lgpD01	3.40.420.10	2.60.40.10
4lgrA	4lgrA01_4lgrB01	3.40.420.10	2.60.40.10
4lhqC	4lhqC01_4lhqD01	3.40.420.10	2.60.40.10
4lluB	4lluB01_4lluA01	2.60.40.10	2.60.40.10
4lluB	4lluB02_4lluA02	2.60.40.10	2.60.40.10
4lluC	4lluC01_4lluD01	2.60.40.10	2.60.40.10
4lluC	4lluC02_4lluD02	2.60.40.10	2.60.40.10
4llvB	4llvB01_4llvA01	2.60.40.10	2.60.40.10
4llvD	4llvD01_4llvC01	2.60.40.10	2.60.40.10
4llyC	4llyC01_4llyD01	2.60.40.10	2.60.40.10
4llyC	4llyC02_4llyD02	2.60.40.10	2.60.40.10
4lmsA	4lmsA01_4lmsB01	3.90.510.10	1.10.490.20
4lmsA	4lmsA01_4lmsC01	3.90.510.10	3.90.510.10
4lmsA	4lmsA01_4lmsD01	3.90.510.10	1.10.490.20
4lmsC	4lmsC01_4lmsD01	3.90.510.10	1.10.490.20
4lnbA	4lnbA01_4lnbB01	1.25.40.120	1.50.10.20
4lo3B	4lo3B01_4lo3A01	2.80.10.50	2.80.10.50
4lo3B	4lo3B01_4lo3C01	2.80.10.50	2.80.10.50
4lszC	4lszC01_4lszF01	3.40.50.1460	1.25.40.20
4lszE	4lszE01_4lszA01	1.25.40.20	3.40.50.1460
4lvnC	4lvnC01_4lvnA01	2.60.40.10	3.40.50.200
4lvoA	4lvoA01_4lvoC01	3.40.50.200	2.60.40.10
4lvoB	4lvoB01_4lvoC01	2.60.40.10	2.60.40.10
4lvoB	4lvoB02_4lvoC02	2.60.40.10	2.60.40.10
4lylE	4lylE01_4lylF01	3.40.470.10	3.10.450.20
4lylJ	4lylJ01_4lylI01	3.10.450.20	3.40.470.10
4m1uF	4m1uF01_4m1uA02	2.40.50.70	4.10.470.10
4m3kA	4m3kA01_4m3kB01	3.40.710.10	2.60.40.10
4m61A	4m61A01_4m61B01	2.60.40.10	2.60.40.10
4m61A	4m61A02_4m61B02	2.60.40.10	2.60.40.10
4m93B	4m93B01_4m93C01	2.60.40.10	2.60.40.10
4m93B	4m93B02_4m93C02	2.60.40.10	2.60.40.10
4mayC	4mayC01_4mayD01	2.60.40.10	2.60.40.10
4mayC	4mayC02_4mayD02	2.60.40.10	2.60.40.10
4mcxA	4mcxA01_4mcxB01	1.10.260.40	3.30.2310.20
4mjsE	4mjsE01_4mjsD01	3.10.20.90	3.10.20.90
4mjsG	4mjsG01_4mjsH01	3.10.20.90	3.10.20.90
4mjsX	4mjsX01_4mjsO01	3.10.20.90	3.10.20.90
4mjsX	4mjsX01_4mjsW01	3.10.20.90	3.10.20.90
4mqjD	4mqjD01_4mqjC01	1.10.490.10	1.10.490.10
4mqkG	4mqkG01_4mqkH01	1.10.490.10	1.10.490.10
4n1eJ	4n1eJ01_4n1eC01	1.10.530.10	2.60.40.10
4n1eJ	4n1eJ01_4n1eD01	1.10.530.10	2.60.40.10

4ndrB	4ndrB01_4ndrA01	3.40.1160.10	3.40.1160.10
4ni2A	4ni2A01_4ni2B01	3.30.70.1230	3.30.70.1230
4nikA	4nikA01_4nikB01	1.25.40.20	2.60.40.10
4ntwC	4ntwC01_4ntwB01	1.20.90.10	4.10.410.10
4ntxB	4ntxB01_4ntxC01	4.10.410.10	1.20.90.10
4nujB	4nujB01_4nujA01	2.60.40.10	2.60.40.10
4nujB	4nujB02_4nujA02	2.60.40.10	2.60.40.10
4nwpD	4nwpD01_4nwpG01	1.20.1200.10	3.30.429.10
4ob3B	4ob3B01_4ob3A01	1.10.472.20	3.90.330.10
4ob3B	4ob3B02_4ob3A01	2.30.30.50	3.90.330.10
4ontC	4ontC01_4ontD01	1.50.10.20	2.10.70.10
4orzB	4orzB01_4orzC01	3.30.62.10	2.60.40.10
4p1xB	4p1xB01_4p1xA01	2.70.240.10	2.70.240.10
4p1xH	4p1xH01_4p1xG01	2.70.240.10	2.70.240.10
4peqA	4peqA01_4peqB01	3.10.130.10	3.80.10.10
4peqD	4peqD01_4peqC01	3.80.10.10	3.10.130.10
4perA	4perA01_4perB01	3.80.10.10	3.10.130.10
4pjeC	4pjeC02_4pjeE01	2.60.40.10	2.60.40.10
4pjhE	4pjhE01_4pjhC02	2.60.40.10	2.60.40.10
4pjiH	4pjiH01_4pjiA01	2.60.40.10	3.30.500.10
4pjiH	4pjiH01_4pjiG01	2.60.40.10	2.60.40.10
4pjiH	4pjiH02_4pjiG02	2.60.40.10	2.60.40.10
4po5A	4po5A01_4po5B01	1.10.490.20	1.10.490.20
4po5A	4po5A01_4po5F01	1.10.490.20	1.10.490.20
4po5D	4po5D01_4po5C01	1.10.490.20	1.10.490.20
4po5D	4po5D01_4po5E01	1.10.490.20	1.10.490.20
4prhA	4prhA01_4prhD01	3.30.500.10	2.60.40.10
4priA	4priA01_4priD01	3.30.500.10	2.60.40.10
4priD	4priD01_4priE01	2.60.40.10	2.60.40.10
4priD	4priD02_4priE02	2.60.40.10	2.60.40.10
4qd2D	4qd2D01_4qd2B01	2.80.10.50	2.80.10.50
4qjfA	4qjfA01_4qjfB01	3.30.1490.120	1.10.287.420
4qjfA	4qjfA02_4qjfB02	2.40.50.140	1.10.150.80
4qo1A	4qo1A01_4qo1B01	2.60.40.10	2.60.40.720
4r40D	4r40D01_4r40C02	3.30.1330.60	2.120.10.30
4rhwA	4rhwA01_4rhwE01	1.10.533.10	1.10.533.10
4rhwA	4rhwA01_4rhwF01	1.10.533.10	1.10.533.10
4rhwE	4rhwE01_4rhwC01	1.10.533.10	1.10.533.10
4txoB	4txoB01_4txoA01	3.40.30.10	3.40.30.10
4txoE	4txoE01_4txoF01	3.40.30.10	3.40.30.10
4txvA	4txvA01_4txvB01	3.40.30.10	2.60.40.420
4uujC	4uujC01_4uujA01	1.10.287.70	2.60.40.10
6reqA	6reqA01_6reqB01	3.20.20.240	3.20.20.240
7reqD	7reqD01_7reqC01	3.20.20.240	3.20.20.240
8fabD	8fabD01_8fabC01	2.60.40.10	2.60.40.10
8fabD	8fabD02_8fabC02	2.60.40.10	2.60.40.10
