# Supplementary material for: Unraveling the structural landscape of intra-chain domain interfaces: Implication in the evolution of domain-domain interactions
Source: PLoS One. 2019 Aug 2;14(8):e0220336. doi: 10.1371/journal.pone.0220336 (PMC6677297; doi:10.1371/journal.pone.0220336)
Supplement: S1 File — Figure A: Overview of non-redundant dataset construction. A Flow chart showing steps of non-redundant domain pairs dataset generation with an example. Figure B: Distribution of scores for the best structural interface matches of domain-CC-M. Scatter plot of interfacial RMSD versus (1) fraction of aligned residues (fres) and (2) fraction of aligned contacts (fcon) for the closest match of 759 consecutive continuous domain-domain interfaces extracted from proteins with >2 CATH structural domains. Each point is colored based on IS-score. Distribution of IS-score is shown as histogram. The same scheme is used in Figure D. Figure C: Comparison of interface planarity and IS-score. Scatter plot showing relationship between planarity of domain-domain interface to the best IS-score of interface obtained for each representative 2270 consecutive domains. Planarity is measured using PRINCIP program in SURFNET [Laskowski, 1995] suit of programs that is a root-mean square deviation between interface Cα-atoms and the best fit of plane through the interface Cα-atoms. Figure D: Distribution of scores for the best structural interface matches of domain-CU-2. Scatter plot of interfacial RMSD versus (1) fraction of aligned residues (fres) and (2) fraction of aligned contacts (fcon) for the closest match of 512 consecutive and non-continuous domains extracted from proteins with only two CATH structural domains. Each point is colored based on IS-score. Distribution of IS-score is shown as histogram. Figure E: Network connectivity of DDI in structural space. The fraction of directed pairs of nodes (interfaces), which are connected with at most kth neighbor are plotted as a function of IS-score. This is shown for DDI and a combined DDI+PPI interfaces in panels (1) and (3) respectively. Here, fraction is computed as nk/(N x (N-1)), where nk is the number of kth neighbor pairs and N is total number of interfaces in a graph. The relative size of LSCC at various k for graphs generated at a giv [file pone.0220336.s002.pdf]

Supporting information of the manuscript titled "Unraveling the structural landscape of intra-chain domain interfaces: implication in the evolution of domain-domain interactions"

Rivi Verma and Shashi Bhushan Pandit*

Department of Biological Sciences,

Indian Institute of Science Education and Research, Mohali,

Knowledge City, Sector 81, SAS Nagar, Manuali PO 140306, India,

*To whom correspondence should be addressed.

Shashi Bhushan Pandit
Assistant Professor
Department of Biological Sciences
Indian Institute of Science Education and Research Mohali
Email: shashibp@iisermohali.ac.in
Phone no: +91 172 229 2321

Appendix A
Procedure to generate non-redundant domain pair dataset

In order to generate a list of representative domain-domain interfaces, we filtered the redundant interactions using CD-Hit. As illustrated in Figure A, all individual domain sequences were clustered hierarchically at 40% sequence identity. First, all domains pairs in a given protein are selected such that two domains of a given domain pair belongs to different clusters. This excludes domain pairs belonging to the same cluster such as P1D1-P1D2 in Figure A. Thus, making sure that at least one domain has sequence identity < 40% with other corresponding domains in domain pairs.

Next, we make all cluster combinations to find representative for non-redundant dataset. For each such combination of cluster, first the common pdb entries having different domain numbers between two clusters are identified. Then, depending on the number of structures (zero, one or more) identified in previous step following is performed: a) if there are zero common cluster members (structures), then no structure is selected, b) if only one common entry exists, then this is taken as representative structure, and c) if there are more than one common pdb entry, then a representative non-redundant structure is selected that has highest resolution with longest length (P5D1-P5D2 in cluster_0). There are some domains, which are clustered as single unit (P4D3 in cluster_2) given the CD-Hit algorithm. We specifically checked for these cases and calculated the sequence identity of this lone domain with its corresponding domains in different proteins (P6D3, P6D1). If the sequence identity is > 40%, we merge this domain in the other cluster (cluster_3/cluster_4), otherwise it will be included in the final dataset. We also made sure that we conserve the domain order in a given protein. In cases, where domain order is reversed we selected both the domain pairs (P6D3-P6D1 and P7D1-P7D3).

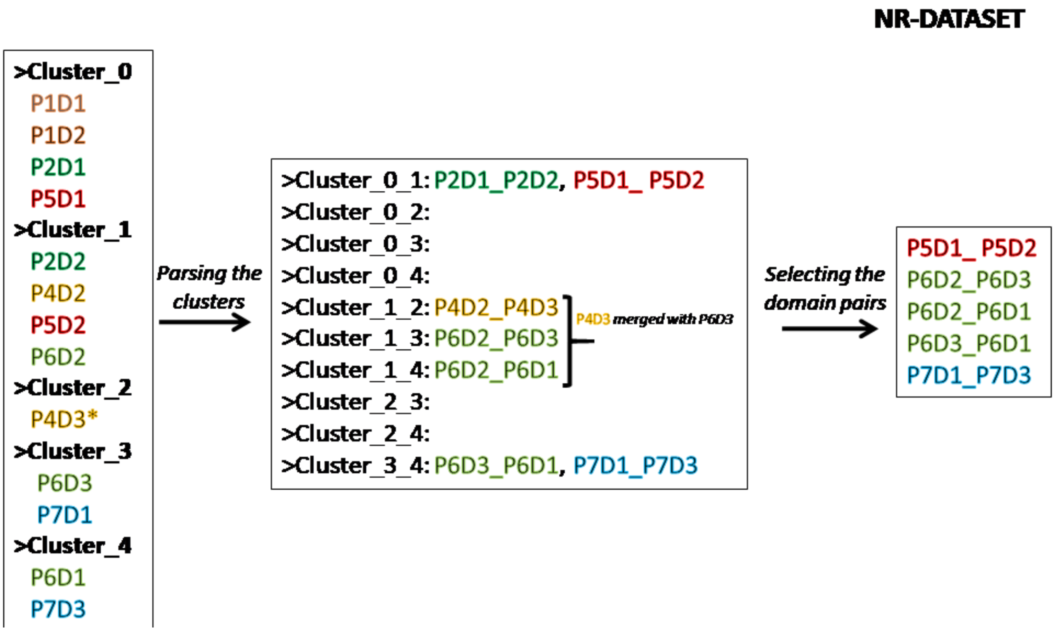
NR-DATASET
>Cluster_0
P1D1
P1D2
P2D1
P5D1
>Cluster_1
P2D2
P4D2
P5D2
P6D2
>Cluster_2
P4D3*
>Cluster_3
P6D3
P7D1
>Cluster_4
P6D1
P7D3
Parsing the clusters
>Cluster_0_1: P2D1_P2D2, P5D1_ P5D2
>Cluster_0_2:
>Cluster_0_3:
>Cluster_0_4:
>Cluster_1_2: P4D2_P4D3
>Cluster_1_3: P6D2_P6D3
>Cluster_1_4: P6D2_P6D1
>Cluster_2_3:
>Cluster_2_4:
>Cluster_3_4: P6D3_P6D1, P7D1_P7D3
P4D3 merged with P6D3
Selecting the domain pairs
P5D1_ P5D2
P6D2_P6D3
P6D2_P6D1
P6D3_P6D1
P7D1_P7D3

Figure A

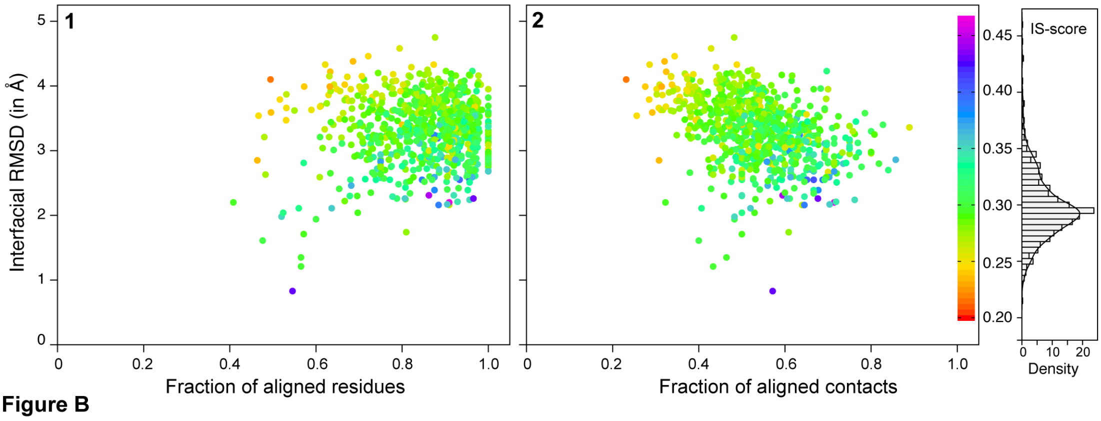
1
2
Interfacial RMSD (in Å)
Fraction of aligned residues
Fraction of aligned contacts
IS-score
Density
0
0.2
0.4
0.6
0.8
1.0
0.20
0.25
0.30
0.35
0.40
0.45
10
20

Figure B

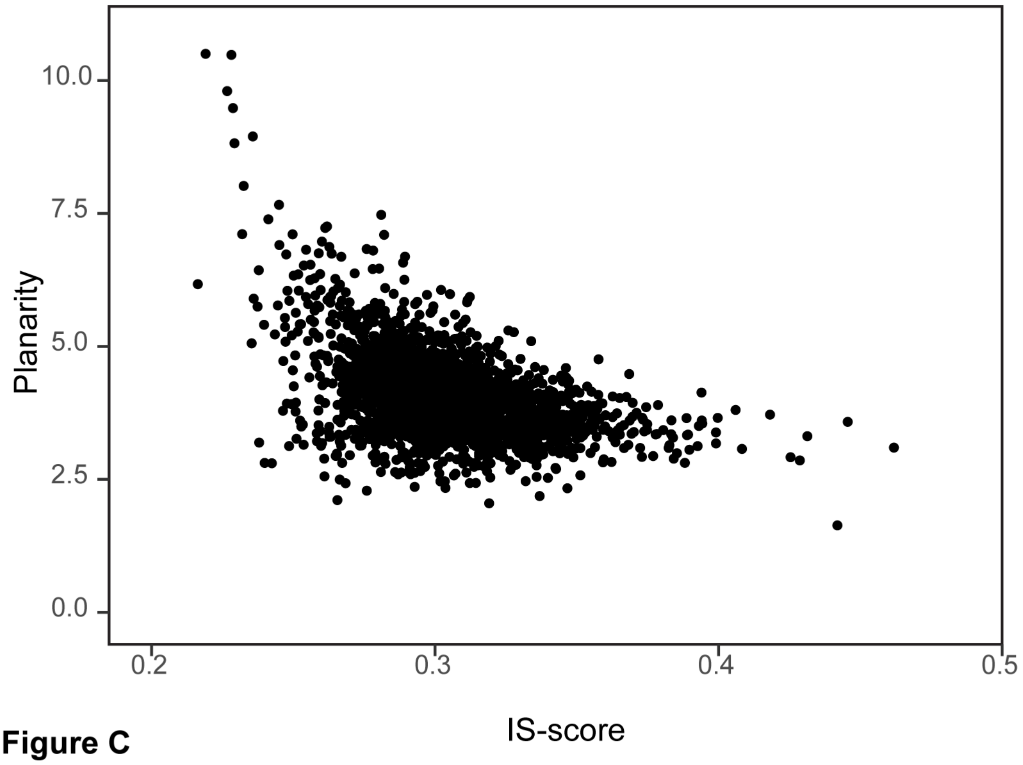

Figure C

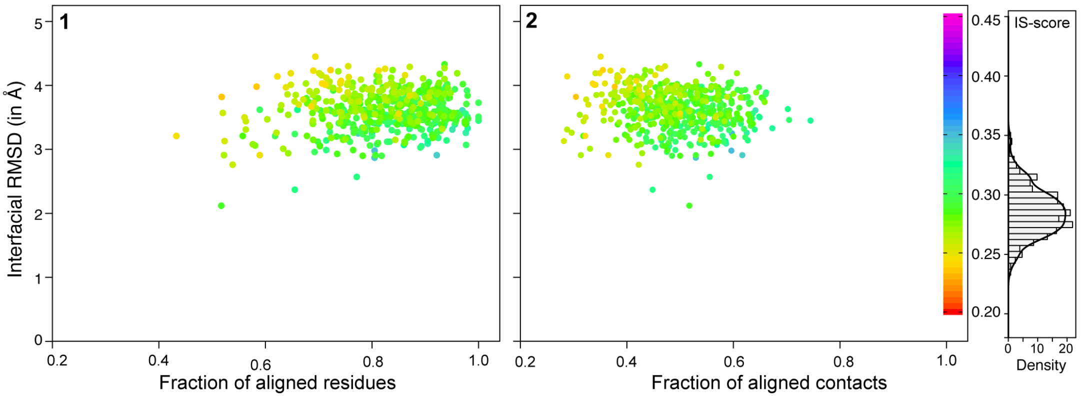
1
2
Interfacial RMSD (in Å)
Fraction of aligned residues
Fraction of aligned contacts
IS-score
Density

Figure D

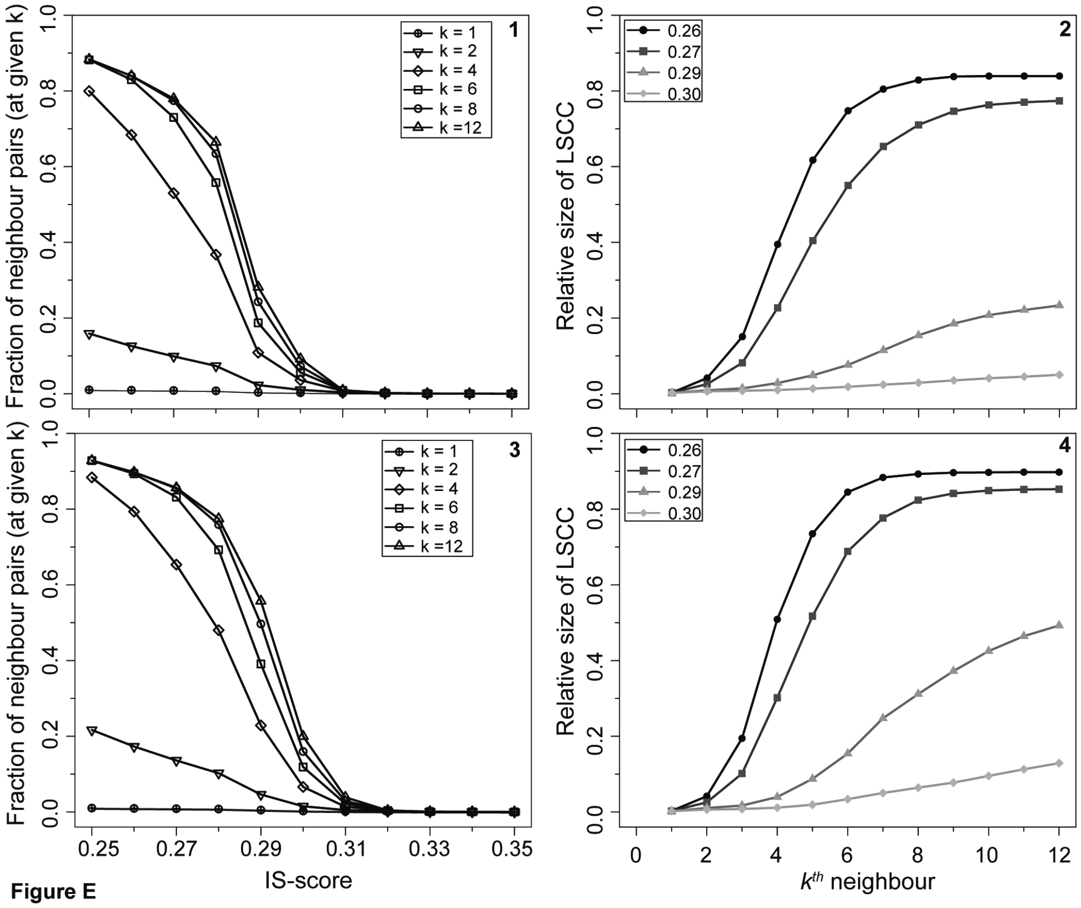

Figure E

References:

Laskowski RA: SURFNET: a program for visualizing molecular surfaces, cavities, and intermolecular interactions. Journal of molecular graphics 1995, 13(5): 323-330, 307-328
